# Equity-specific effects of interventions to promote physical activity among middle-aged and older adults: results from applying a novel equity-specific re-analysis strategy

**DOI:** 10.1186/s12966-021-01131-w

**Published:** 2021-05-17

**Authors:** Gesa Czwikla, Filip Boen, Derek G. Cook, Johan de Jong, Tess Harris, Lisa K. Hilz, Steve Iliffe, Lilian Lechner, Richard W. Morris, Saskia Muellmann, Denise A. Peels, Claudia R. Pischke, Benjamin Schüz, Martin Stevens, Klaus Telkmann, Frank J. van Lenthe, Julie Vanderlinden, Gabriele Bolte

**Affiliations:** 1 Department of Social Epidemiology, Institute of Public Health and Nursing Research, University of Bremen, Bremen, Germany; 2 Health Sciences Bremen, University of Bremen, Bremen, Germany; 3 Physical Activity, Sports & Health Research Group, Department of Movement Sciences, KU Leuven, Leuven, Belgium; 4 Population Health Research Institute, St George’s University of London, London, UK; 5 School of Sports Studies, Hanze University of Applied Sciences, Groningen, The Netherlands; 6 Research Department of Primary Care & Population Health, University College London, London, UK; 7 Faculty of Psychology, Open University, Heerlen, The Netherlands; 8 Department of Population Health Sciences, Bristol Medical School, University of Bristol, Bristol, UK; 9 Leibniz Institute for Prevention Research and Epidemiology – BIPS, Bremen, Germany; 10 Institute of Medical Sociology, Centre for Health and Society, Medical Faculty, Heinrich Heine UniversityDuesseldorf, Duesseldorf, Germany; 11 Department of Prevention and Health Promotion, Institute of Public Health and Nursing Research, University of Bremen, Bremen, Germany; 12 Department of Orthopedics, University of Groningen, University Medical Center Groningen, Groningen, The Netherlands; 13 Department of Public Health, Erasmus University Medical Center Rotterdam, Rotterdam, The Netherlands

**Keywords:** Physical activity, Social inequalities, Interventions, Intervention-generated inequalities, Equity impact assessment, Re-analysis, Middle-aged adults, Older adults

## Abstract

**Background:**

Reducing inequalities in physical activity (PA) and PA-associated health outcomes is a priority for public health. Interventions to promote PA may reduce inequalities, but may also unintentionally increase them. Thus, there is a need to analyze equity-specific intervention effects. However, the potential for analyzing equity-specific effects of PA interventions has not yet been sufficiently exploited. The aim of this study was to set out a novel equity-specific re-analysis strategy tried out in an international interdisciplinary collaboration.

**Methods:**

The re-analysis strategy comprised harmonizing choice and definition of outcomes, exposures, socio-demographic indicators, and statistical analysis strategies across studies, as well as synthesizing results. It was applied in a collaboration of a convenience sample of eight European PA intervention studies in adults aged ≥45 years. Weekly minutes of moderate-to-vigorous PA was harmonized as outcome. Any versus no intervention was harmonized as exposure. Gender, education, income, area deprivation, and marital status were harmonized as socio-demographic indicators. Interactions between the intervention and socio-demographic indicators on moderate-to-vigorous PA were analyzed using multivariable linear regression and random-effects meta-analysis.

**Results:**

The collaborative experience shows that the novel re-analysis strategy can be applied to investigate equity-specific effects of existing PA interventions. Across our convenience sample of studies, no consistent pattern of equity-specific intervention effects was found. Pooled estimates suggested that intervention effects did not differ by gender, education, income, area deprivation, and marital status.

**Conclusions:**

To exploit the potential for equity-specific effect analysis, we encourage future studies to apply the strategy to representative samples of existing study data. Ensuring sufficient representation of ‘hard to reach’ groups such as the most disadvantaged in study samples is of particular importance. This will help to extend the limited evidence required for the design and prioritization of future interventions that are most likely to reduce health inequalities.

**Supplementary Information:**

The online version contains supplementary material available at 10.1186/s12966-021-01131-w.

## Background

Reducing health inequalities - defined as socio-demographic differences in life-expectancy, morbidity, and mortality - has become an important public health priority [1]. Socio-demographic differences have also been shown in health behaviors, including physical activity (PA), an important determinant of healthy ageing [2–4]. The proportion of individuals with sufficient PA levels, however, declines with age, with particularly low levels of PA among middle-aged and older adults [5, 6]. Furthermore, lower leisure-time PA levels have been associated with low socio-economic position (SEP), being female, belonging to an ethnic minority group, living in a deprived neighborhood, and not having a spouse [7–11]. Because being physically active regularly has numerous beneficial effects on physical and mental wellbeing [12, 13], it is likely that inequalities in PA are an important contributor to health inequalities [14].

Public health interventions have the potential to reduce existing health inequalities, but in particular interventions that aim at changing individual behavior (‘downstream interventions’) may also unintentionally increase them (‘intervention-generated inequalities’; [15, 16]). One major reason for this is that downstream interventions in contrast to policy-change (‘upstream’) interventions usually require relatively more individual psychological, temporal, and material resources (‘individual agency’ [17];) to succeed. Such resources are unequally distributed between different population groups, favoring predominantly those at the upper end of the socio-economic spectrum [17–19]. In this regard, it has also been found that the links between psychosocial determinants of health behavior, such as attitudes and intentions, and health behavior are more pronounced and have stronger effects on behavior among high- than among low-SEP individuals [20, 21]. Thus, interventions based on these psychosocial determinants may unintentionally increase inequalities by benefiting high-SEP individuals disproportionally more. The relevance of individual agency for equity-specific effects of behavioral interventions is empirically supported by systematic reviews of interventions in different areas, including tobacco control [22], obesity prevention [23], and healthy eating [24]. Divergent perceptions between low-SEP individuals and health promoters regarding lifestyle, lifestyle change, and support for lifestyle change are a further possible explanation for interventions being less beneficial in low-SEP population groups [25].

The effects of PA interventions may not only differ by SEP but also by gender and other relevant socio-demographic indicators associated with inequalities and PA, such as ethnicity and marital status [26–28]. With regard to gender, there are differences between males and females in preferred PA domains and contexts, as well as in motivational factors and barriers to PA [29–31]. Compared with males, females appear to be more motivated by the social aspects of PA (e.g., spending time with others and meeting friends), by losing or managing weight, and by improving appearance. They tend to be less motivated than men to participate in physical activities that are vigorous, require skill and practice, involve some kind of competition, and are done outdoors [30]. Moreover, compared with males, females more often take over domestic and care responsibilities, not infrequently carried out in addition to paid work, leaving little time for leisure activities such as PA [31]. With regard to ethnicity, minority ethnic groups may face additional barriers to PA engagement, for example due to differing perceptions about and attitudes towards PA as well as cultural expectations [32].

Results of an equity-focused systematic review by Attwood and colleagues [27] indicate that the effects of primary-care-based PA interventions may differ by gender, but there was no consistent pattern regarding the direction of these differences. This is in line with the results of another equity-focused systematic review of interventions to promote PA among adults aged ≥50 years by Lehne & Bolte [28]. As reported by Humphreys & Ogilvie [26], the effects of environmental and policy interventions to promote PA may differ by ethnicity and gender, whereby members of the majority population seemed to benefit more from the interventions than members of ethnic minority populations. Like Attwood et al. [27] and Lehne & Bolte [28], this review also found no consistent pattern regarding the direction of gender-specific intervention effects. All three reviews concluded that, because of the paucity of studies that actually report equity-specific effect analyses, it is difficult to draw implications for the design of future interventions that could effectively reduce PA inequalities according to SEP, gender, and other relevant socio-demographic indicators (e.g., ethnicity, marital status) [26–28]. Such indicators are frequently measured in studies, but only a minority of studies explicitly analyze equity-specific intervention effects. The potential for assessing intervention effects on inequalities in PA has not yet been fully exploited [26–28].

Analyzing equity-specific intervention effects requires interaction or subgroup analyses that compare intervention effects across different population subgroups defined by socio-demographic characteristics [33]. A criticism of this approach is that few studies are designed with adequate sample sizes to run such interaction or subgroup analyses, so that many of the current findings are based on potentially underpowered post-hoc analyses with limited credibility [33, 34]. However, given the importance of better understanding whether, how, and why interventions affect health inequalities, and the plausibility of differential intervention effects, equity-specific re-analyses of data of existing intervention studies are arguably a valuable approach [35–41]. One particular reason is that the consistent conduct and reporting of such analyses allows for pooling effect estimates across studies, which increases statistical power and improves the credibility of the findings [42]. As re-analyses require access to complete primary data (including individual participant data) and detailed knowledge of the individual studies going beyond the information usually given in publications, a collaborative approach involving researchers from the primary studies seems necessary.

The aim of this study was to set out a novel strategy for re-analyzing equity-specific intervention effects and to try out its application in an international interdisciplinary collaboration between existing individual-level PA intervention studies in middle-aged and older adults.

## Methods

This study was conducted as part of the project “EQUAL - Equity impacts of interventions to increase physical activity”, a subproject within the prevention research network “AEQUIPA - Physical activity and health equity: primary prevention for healthy ageing” [43]. EQUAL aimed to develop and try out a strategy for re-analyzing equity-specific effects of PA interventions in an international interdisciplinary collaboration [44]. The collaboration was initiated based on researchers representing eight published European PA intervention studies in middle-aged and older adults [45–52] (a convenience sample of 20 eligible studies), as well as experts on equity-specific data analysis. In accordance with previous studies [53, 54], middle-aged and older adults were defined as individuals aged 45 years and older. As well as using the AEQUIPA intervention study PROMOTE [52], studies were identified through a literature search [44]. Inclusion criteria were: studies reporting the effects of individual-level PA interventions; targeted at community-dwelling adults aged ≥45 years; with a randomized or non-randomized controlled longitudinal study design in which the control group received no intervention; and reporting on participants’ age, gender, as well as on at least one measure of SEP (i.e., education, income, occupation, composite SEP). The collaborating researchers represent various disciplines, including (social) epidemiology, biostatistics, health psychology, primary care research, sport and human movement sciences.

Plenary and bilateral meetings (face-to-face and online) and e-mail correspondence were used to develop the re-analysis strategy and to define common criteria for adopting it to the sample of studies included in the collaboration. First, the EQUAL study team outlined ideas for the strategy to be developed, informed by: 1) available evidence about equity-specific effects of PA interventions; 2) concepts and theories of how interventions may affect health inequalities; and 3) existing approaches to equity-specific re-analysis. The outline was sent to the collaborating researchers via e-mail with a request for feedback and subsequently revised by the EQUAL study team according to the feedback received. In a next step, the collaborating researchers were invited to a one-day face-to-face workshop in Bremen, Germany, to find consensus about the individual steps of the strategy based on the revised outline as well as to discuss common criteria for adapting the strategy to the eight included studies. Based on the results of the discussion, the EQUAL study team developed draft criteria for re-analyzing equity-specific effects of the individual studies, which were revised after two rounds of iterative discussion by e-mail. These criteria were applied by members of the research group to their own data (i.e., there was no pooling of the studies’ individual participant data) with or without assistance from the EQUAL study team. Finally, criteria for combining the results from the individual studies were added. These criteria were developed by statistician colleagues of the collaboration working with the EQUAL study team and were agreed at an online meeting.

### Characteristics of studies included in the collaboration

Details of the eight intervention studies are presented in Additional file 1. Three studies were conducted in the United Kingdom, three in the Netherlands, one in Belgium, and one in Germany. Seven studies were (cluster-)randomized controlled trials, and one was a controlled before and after study. Baseline sample sizes varied between 298 and 2140 participants. Two studies (GALM, PACE-UP) recruited exclusively physically inactive participants. Study participants were either recruited via the community (Active Plus I, Active Plus II, Every Step Counts!, GALM, PROMOTE) or through primary care (PACE-Lift, PACE-UP, ProAct65+). While all eight studies aimed to increase PA, three (PACE-Lift, PACE-UP, Every Step Counts!) had a particular focus on promoting walking, and one (GALM) on promoting recreational sports activities. Three studies (Every Step Counts!, PACE-Lift, PACE-UP) delivered individual-level pedometer-based walking programs, three personalized PA advices without (Active Plus I, Active Plus II) or with community-based group meetings (PROMOTE), one (GALM) group-based PA sessions in a gymnasium in the neighborhood, and one (ProAct65+) a home- or class-based exercise program. Intervention length ranged between 10 and 26 weeks.

## Results

### Equity-specific re-analysis strategy

The equity-specific re-analysis strategy comprises harmonizing the choice and definitions of outcomes (step 1), exposures (step 2), socio-demographic indicators (step 3), and statistical analysis strategies (step 4) across studies by defining common criteria; as well as synthesizing the results (step 5). The following sections provide detailed descriptions of the individual steps of the strategy and how to adopt them to existing study data. To do so, we present the criteria for harmonization and synthesizing results as defined for our convenience sample of PA intervention studies.

#### Step 1: harmonizing the choice and definition of outcome measures across studies

The first step includes choosing an outcome measure which adequately measures the objectives of the kind of intervention under study and which can be defined across studies as similar as possible. Health promoting behaviors such as PA need to be maintained for long-term health benefits [55, 56]. Moreover, it has been shown that inequalities may initially increase after implementation of new interventions before decreasing again as time passes [57]. Therefore, in order to make conclusions about inequalities in long-term health benefits, where data permit, both short-term and long-term outcomes of the interventions should be considered.

For our sample of PA intervention studies, we identified weekly minutes of moderate-to-vigorous PA (MVPA) at the post-intervention follow-up time point closest to the intervention end point (T1) as primary outcome because it could be defined in a similar manner across the studies and the beneficial effects of MVPA on health are well documented [58]. Considering the data of five studies, weekly minutes of MVPA at the next follow-up assessment (T2) was chosen as secondary outcome to investigate potential changes in equity-specific intervention effects over time. This was 8 months post-intervention for Active Plus I and Active Plus II, 9 months post-intervention for PACE-Lift and PACE-UP, and 6 months post-intervention for ProAct65+. Due to better precision and accuracy [59], we decided to prefer objective PA measures over subjective measures, when both were available in a study. In Active Plus I, Active Plus II, Every Step Counts!, GALM, and ProAct65+ that measured PA exclusively subjectively, physical activities of at least three metabolic equivalents (MET) were defined as MVPA, following recommendations by guidelines [60]. In PACE-Lift, PACE-UP, and PROMOTE that measured PA objectively, the standard Freedson cut-point of 1952 counts per minute [61], equivalent to three METs, was used to define MVPA. In addition to the main outcome total weekly minutes of MVPA, sensitivity analyses were conducted for PACE-Lift and PACE-UP using weekly minutes of MVPA in bouts of at least 10 min.

#### Step 2: harmonizing the choice and definition of exposure measures across studies

Studies of interventions may differ with regard to the number of intervention and control groups. Step two includes choosing an exposure measure which can be defined across studies as similar as possible.

For our sample of PA intervention studies, any versus no intervention was defined as exposure. In Active Plus I, Active Plus II, PACE-UP, ProAct65+, and PROMOTE which included several intervention groups, intervention groups were combined to create a single pair-wise comparison in order to avoid double-counting. The Cochrane Handbook for Systematic Reviews of Interventions recommends this approach for including studies with several intervention groups in a meta-analysis [62].

#### Step 3: harmonizing the choice and definition of socio-demographic indicators across studies

Step three includes harmonizing the choice and definition of socio-demographic indicators which should be based on existing theories and evidence of equity-specific intervention effects. There are several different socio-demographic indicators that might be relevant to consider. The PROGRESS-Plus framework [63], proposed by the Campbell and Cochrane Equity Methods Group, may help researchers in identifying socio-demographic indicators relevant for their specific research question. SEP should be considered a multidimensional construct comprising diverse socio-economic indicators at the individual, household, or contextual level [64–67]. Because different indicators of SEP operate through different causal pathways and may have different relevance among individuals of varying age and gender [64–67], the choice of SEP indicator may affect findings about the presence and extent of equity-specific intervention effects. It is therefore important to consider, and clearly differentiate between, various relevant SEP indicators instead of focusing on one indicator only or using several SEP indicators interchangeably. Moreover, potential intersections between several socio-demographic indicators [68, 69], such as gender and SEP, should be considered. Putting such an intersectionality lens to the re-analysis of data of intervention studies, where sample size and diversity permit, could yield even more comprehensive insights on the impact of these interventions on health inequalities.

For our sample of PA intervention studies, education as a measure of SEP [64–67] and gender (only defined as female versus male) as a social construct [70, 71] were selected as main socio-demographic indicators because both characteristics have previously been shown to moderate the effects of PA interventions [26–28], information on both were available in all collaborating studies, and both can be operationalized in a similar manner across studies from different countries. Education was defined according to the International Standard Classification of Education (ISCED) 2011 [72]. Based on the highest level of educational qualification or age at leaving full time education, individuals were grouped into the categories “Low” (at most lower secondary education (ISCED 0–2) or leaving full time education at ≤16 years), “Medium” (upper secondary and post-secondary non-tertiary education (ISCED 3–4) or leaving full time education at 17–18 years), or “High” (tertiary education (ISCED 5–8) or leaving full time education at ≥19 years).

In a secondary analysis, income and area deprivation as measures of SEP [64–67] were considered. Information on household income was available in two (ProAct65+, PROMOTE) and information on area deprivation (index of multiple deprivation [IMD] score [73]) was available in three studies (PACE-Lift, PACE-UP, ProAct65+). For both of these indicators, in each study, tertiles were defined in terms of the distribution in the study’s specific data set. This resulted in two variables with the categories “Low”, “Medium”, and “High” each for household income and area deprivation (see Additional file 2 for details). Additionally, marital status (defined as having versus not having a partner) was considered as a socio-demographic indicator because the presence or absence of a spouse has been shown to be associated with health inequalities and PA [10, 74].

Although the effects of PA interventions my also differ between individuals of different ethnic backgrounds, we did not consider ethnicity as a socio-demographic indicator due to differing ethnic compositions in the study populations and data availability. Potential intersections between several socio-demographic indicators were also not considered because of small sample size and insufficient diversity.

#### Step 4: harmonizing the choice and definition of statistical analysis strategies across studies

Step four comprises to specify the statistical methods and modeling strategies for the equity-specific effect analyses. Not only intervention effects, but also intervention reach, adherence, and dropout may also differ by socio-demographic characteristics and therefore should be considered for a comprehensive assessment of equity-specific intervention benefits [15, 75].

##### Equity-specific intervention reach

In our sample of PA intervention studies, the majority lacked information on socio-demographic indicators for non-participants. This precluded the calculation of socio-demographic group-specific response rates [76, 77], so it was not possible to investigate equity-specific intervention reach. We originally aimed to consult census data and to compare the study population with the targeted population of each study, considering the studies’ specific eligibility criteria. However, as no suitable census data could be identified, we decided to calculate an overall response percentage, defined as the number of persons who completed the baseline (T0) questionnaire and were assigned to the intervention conditions, divided by the number of persons invited to participate. For Every Step Counts! and PROMOTE, only estimations of response percentages could be made because the recruitment strategies comprised advertising. For each study, the distribution of gender, education, income, area deprivation, and marital status groups as well as the mean age in the intervention and control groups at T0 were calculated.

##### Equity-specific intervention adherence and dropout

We calculated percentages and means to describe adherence and dropout stratified by socio-demographic indicators. Information on intervention adherence was available in Active Plus II, GALM, PACE-UP, and PROMOTE, relating to the use of intervention materials and/or attendance at group meetings. We defined *dropouts* as individuals with valid information on MVPA at T0 but without valid information at T1. Additionally, we calculated mean values and corresponding standard deviations (SD) of weekly minutes of MVPA at T0 for each subgroup of interest, stratified by intervention and control group, as well as by completers and dropouts.

##### General and equity-specific intervention effects

The general intervention effect was defined as the difference between the intervention and control groups in minutes of MVPA per week at T1 (main analysis) or T2 (secondary analysis). For this purpose, post-intervention values of weekly minutes of MVPA were regressed on intervention versus control group and minutes of MVPA per week at T0 without (minimally adjusted model) and with adjustment for age in years, gender, and education (fully adjusted model). Due to the nature of the data, in four studies, the models were additionally (multilevel-)adjusted for practice (PACE-Lift, PACE-UP, ProAct65+); household (PACE-Lift, PACE-UP); or community, valid wear-time, and season (PROMOTE). All analyses were conducted by intention-to-treat, analyzing participants according to the group to which they were originally assigned, restricting the models to individuals with complete data on all variables included (i.e., complete case intention-to-treat analysis).

Equity-specific intervention effects were investigated by adding intervention*socio-demographic indicator interaction terms to the regression models. For analyzing equity-specific intervention effects by gender, for example, post-intervention values of weekly minutes of MVPA were regressed on intervention versus control group, MVPA per week at T0, age in years, gender, and the intervention*gender interaction without (minimally adjusted model) and with adjustment for education and the intervention*education interaction (fully adjusted model). Because age is associated with most of the socio-demographic indicators and with PA levels, we decided to include it as a covariate in all models. For each model, the *p*-values for the interaction terms and effect estimates with corresponding 95% confidence interval (CI) for each subgroup of interest were computed. Following Greenland et al. [78], precise *p*-values were reported.

#### Step 5: synthesizing the results

The last step includes synthesizing the results from the individual studies. Meta-analysis is the preferable method because it can increase the power for detecting equity-specific intervention effects which is often limited in post-hoc analysis [33, 34]. If the number of studies permit, meta-regression [79] should be used to investigate possible sources of heterogeneity (e.g. study quality, study design). If the sample of studies is highly heterogeneous and data can hardly be harmonized to enable meta-analysis, there are alternative approaches to synthesize and visualize the equity-specific results of individual studies, such as the harvest plot [80].

In our homogeneous sample of PA intervention studies, after data had been harmonized, the estimates for the regression coefficients of the intervention*socio-demographic indicator interactions from the individual studies were pooled using random-effects meta-analysis. To be able to assess the direction of these interaction effects, in particular for any disadvantage experienced by the most disadvantaged groups, regression models were slightly modified. Education, income, and area deprivation were considered as variables with two (low versus medium/high education and income, high versus medium/low deprivation) instead of three categories resulting in one regression coefficient for each intervention*socio-demographic indicator interaction. This means that for all studies, the socio-demographic indicators were comparable in measurement and levels.

Analyses were conducted in R using the metafor package [81]. As effect size, we chose the point estimates of the intervention*socio-demographic indicator interactions in minutes. A random effects model was fitted using the DerSimonian and Laird method. The extent of heterogeneity was measured by the I^2^ index. Following Higgins et al. [82], I^2^-values of 25, 50, and 75% were considered low, moderate, and high heterogeneity, respectively. The intervention*socio-demographic indicator interaction effect estimates and their corresponding 95% CI were presented in forest plots. Since some studies used different numbers of predictors, a sensitivity analysis was conducted estimating partial correlation coefficients [83]. Meta-regression was deemed inappropriate due to the low number of studies.

#### Risk of bias assessment

Whichever method to synthesize the results is chosen, a risk of bias assessment should be conducted. There is no specific tool for assessing the risk of bias in a result from equity-specific effect analysis. For our sample of studies, we therefore decided to assess the risk of bias regarding the general intervention effects, using the revised Cochrane risk-of-bias tool for randomized trials (RoB 2.0) [84] and the ROBINS-I risk-of-bias tool for non-randomized studies of interventions [85]. The assessment of each study was performed by at least one researcher from the contributing study (FB, TH, SI, RM, SM, DP, MS, JV) and one researcher from the EQUAL project team (GC) independently. Journal article(s), the published re-analysis strategy [44], and internal knowledge about the study were used to help inform the assessment. Any discrepancies were resolved through discussion and, where necessary, consulting the last author (GB).

### Application of the equity-specific re-analysis strategy

The following sections illustrate the application of the equity-specific re-analysis strategy. To do so, we present the results from applying the criteria for adapting the strategy set out above to our convenience sample of PA intervention studies.

#### Risk of bias within studies

Regarding the general intervention effects, the randomized studies PACE-Lift and PACE-UP were judged to be at low risk of bias, and Active Plus I, Active Plus II, GALM, ProAct65+, and PROMOTE at high risk (Table 1). The non-randomized study Every Step Counts! was judged to be at serious risk (Table 2). The high/serious risks resulted from non-concealed randomization sequences, differing proportions of missing outcome data in the intervention and control groups, and/or participant-reported outcome measures. Further details are available in Additional file 3.

**Table 1:** Risk of bias assessment using the revised Cochrane risk-of-bias tool for randomized trials (RoB 2.0)

Study	Risk of bias domain					
	Randomisation process	Deviations from intended interventions	Missing outcome data	Measurement of the outcome	Selection of the reported result	Overall risk of bias^**a**^
Active Plus I	High	Low	High	High	Low	High
Active Plus II	High	Low	Low	High	Low	High
GALM	High	Low	High	High	Low	High
PACE-Lift	Low	Low	Low	Low	Low	Low
PACE-UP	Low	Low	Low	Low	Low	Low
ProAct65+	Low	Low	Low	High	Low	High
PROMOTE	Low	Low	High	Low	Low	High

^a^Low risk of bias: The study is judged to be at low risk of bias for all domains; Some concerns: The study is judged to raise some concerns in at least one domain, but not to be at high risk of bias for any domain; High risk of bias: The study is judged to be at high risk of bias in at least one domain

**Table 2:** Risk of bias assessment using the ROBINS-I risk-of-bias tool for non-randomized studies of interventions

Study	Risk of bias domain							
	Confounding	Selection of participants into the study	Classification of interventions	Deviations from intended interventions	Missing data	Measurement of outcomes	Selection of the reported result	Overall risk of bias^**a**^
Every Step Counts!	Moderate	Low	Low	Low	Moderate	Serious	Low	Serious

^a^Low risk of bias: The study is judged to be at low risk of bias for all domains; Moderate risk of bias: The study is judged to be at low or moderate risk of bias for all domains; Serious risk of bias: The study is judged to be at serious risk of bias in at least one domain, but not at critical risk of bias in any domain; Critical risk of bias: The study is judged to be at critical risk of bias in at least one domain

#### Response percentages and baseline socio-demographic characteristics

Calculated response percentages ranged from 6% in ProAct65+, over 10% in PACE-UP, 12% in GALM, 16% in Active Plus II, 23% in Active Plus I, to 30% in PACE-Lift. Response percentages of PROMOTE and Every Step Counts! were estimated to be 7 and 80%, respectively. Some differences existed between the studies regarding the socio-demographic composition of their baseline samples (Table 3). Most studies had slightly higher percentages of females, ranging from 51% in Active Plus I to 68% in Every Step Counts! (mean = 58%). There was a great variation in the proportion of low-educated participants, ranging from 2% in PROMOTE to 56% in Every Step Counts! (mean = 38%). The percentages of participants without a partner ranged from 18% in Active Plus II to 42% in ProAct65+ (mean = 26%).

**Table 3:** Baseline socio-demographic characteristics

	Active Plus I		Active Plus II		Every Step Counts!		GALM		PACE-Lift		PACE-UP		ProAct65+		PROMOTE	
	IG (*n* = 1384)	CG (*n* = 582)	IG (*n* = 1710)	CG (*n* = 409)	IG (*n* = 468)	CG (*n* = 154)	IG (*n* = 163)	CG (*n* = 152)	IG (*n* = 150)	CG (*n* = 148)	IG (*n* = 685)	CG (*n* = 338)	IG (*n* = 704)	CG (*n* = 400)	IG (*n* = 376)	CG (*n* = 164)
	n (%)	n (%)	n (%)	n (%)	n (%)	n (%)	n (%)	n (%)	n (%)	n (%)	n (%)	n (%)	n (%)	n (%)	n (%)	n (%)
**Gender**																
Males	601 (44)	251 (43)	828 (49)	204 (50)	141 (30)	58 (38)	72 (44)	73 (48)	69 (46)	69 (47)	252 (37)	115 (34)	261 (37)	149 (37)	165 (44)	70 (43)
Females	780 (56)	329 (57)	873 (51)	205 (50)	327 (70)	96 (62)	91 (56)	79 (52)	81 (54)	79 (53)	433 (63)	223 (66)	443 (63)	251 (63)	211 (56)	94 (57)
**Education**																
Low	634 (47)	293 (52)	785 (46)	199 (50)	256 (55)	90 (59)	64 (39)	47 (31)	67 (46)	54 (32)	177 (26)	85 (26)	330 (48)	158 (40)	6 (2)	6 (4)
Medium	267 (20)	103 (18)	451 (27)	107 (27)	152 (33)	41 (27)	53 (33)	70 (46)	25 (17)	20 (14)	142 (21)	83 (25)	218 (32)	135 (34)	187 (50)	93 (57)
High	462 (34)	170 (30)	465 (27)	90 (23)	56 (12)	22 (14)	46 (28)	35 (23)	55 (37)	71 (48)	351 (52)	165 (50)	143 (21)	104 (26)	183 (49)	65 (40)
**Income**																
Low	NA	NA	NA	NA	NA	NA	NA	NA	NA	NA	NA	NA	200 (33)	92 (26)	103 (30)	57 (36)
Medium	NA	NA	NA	NA	NA	NA	NA	NA	NA	NA	NA	NA	158 (26)	114 (33)	111 (32)	43 (27)
High	NA	NA	NA	NA	NA	NA	NA	NA	NA	NA	NA	NA	244 (41)	141 (41)	131 (38)	57 (36)
**Area deprivation**^**a**^																
High	NA	NA	NA	NA	NA	NA	NA	NA	50 (33)	57 (39)	224 (34)	108 (33)	295 (42)	105 (26)	NA	NA
Medium	NA	NA	NA	NA	NA	NA	NA	NA	50 (33)	45 (30)	223 (34)	108 (33)	165 (23)	193 (48)	NA	NA
Low	NA	NA	NA	NA	NA	NA	NA	NA	50 (33)	46 (31)	214 (32)	111 (34)	244 (35)	102 (26)	NA	NA
**Marital status**																
No partner	272 (20)	99 (17)	288 (17)	82 (20)	166 (36)	36 (23)	29 (18)	27 (18)	27 (18)	30 (20)	227 (34)	119 (36)	294 (42)	167 (42)	94 (25)	50 (31)
With partner	1089 (80)	467 (83)	1412 (83)	325 (80)	301 (64)	118 (77)	134 (82)	125 (82)	123 (82)	117 (80)	445 (66)	213 (64)	407 (58)	233 (58)	275 (75)	113 (69)
**Age in years**																
Mean (SD)	63 (±9)	64 (±8)	62 (±8)	64 (±9)	69 (±7)	70 (±6)	60 (±3)	59 (±3)	67 (±4)	66 (±4)	59 (±8)	59 (±8)	73 (±6)	73 (±6)	70 (±3)	70 (±3)

IG Intervention group, *CG* Control group, *NA* Not applicable

^a^Area deprivation based on index for the clusters, not individual participants in ProAct65 +

#### Equity-specific intervention adherence

Results of Active Plus II, GALM, PACE-UP, and PROMOTE with information on intervention adherence indicated no or only slight differences across gender and education subgroups, with no consistent pattern regarding the direction of differences (Table 4). For example, in GALM, slightly higher mean attendance rates of the 15 intervention sessions were observed among low educated participants. In PACE-UP, PA diary return and pedometer use were slightly higher among medium educated individuals. In PROMOTE, females attended the group meetings more often than males. We also found only marginal differences across income, area deprivation, and marital status subgroups. Further details are available in Additional file 4.

**Table 4:** Gender- and education-specific intervention adherence

Study	Measure of adherence	Gender				Education					
		Males		Females		Low education		Medium education		High education	
		n(/N)	%	n(/N)	%	n(/N)	%	n(/N)	%	n(/N)	%
Active Plus II	Tailored advice 1 completely read	405/442	92	452/477	95	395/425	93	212/229	93	250/267	94
	Tailored advice 2 completely read	334/440	76	368/473	78	326/422	77	177/228	78	200/265	76
	Tailored advice 3 completely read	281/332	85	328/369	89	274/314	87	150/175	86	184/211	87
GALM	Mean attendance rate of 15 intervention sessions	36	83	43	77	34	85	23	76	22	77
PACE-UP	PA diary returned after 12-week intervention	201/236	85	339/400	85	137/165	83	121/132	92	271/327	83
	Pedometer used at every day or most days	191/214	89	312/364	86	125/150	83	116/125	93	254/295	86
PROMOTE	Web-based PA diary used	84/97	87	101/121	83	2/2	100	86/99	87	97/117	83
	Group meetings attended	67/98	68	101/125	81	2/2	100	79/102	77	87/119	73

#### Equity-specific intervention dropout

Dropout rates from T0 to T1 varied considerably between the studies, ranging from 6% in PACE-Lift to 45% in Active Plus II. In half of the studies (Active Plus I, Active Plus II, GALM, PROMOTE), intervention group participants were more likely to drop out of the study (Table 5). This bias was mainly the same across gender and education subgroups. In the other half of the studies (Every Step Counts!, PACE-Lift, PACE-UP, ProAct65+), dropout rates were comparable between intervention and control groups, for the total sample, as well as for the gender and education subgroups. Moreover, dropout rates in the intervention and control groups were generally comparable or differed only slightly across gender and education subgroups. For example, in GALM and PROMOTE, dropout rates in the control group slightly differed by gender, with a higher dropout among males (GALM) and females (PROMOTE), respectively.

**Table 5:** General, gender-, and education-specific dropout at T1

**Study**	**Intervention group**											
	**Total sample**		**Gender**				**Education**					
			**Males**		**Females**		**Low education**		**Medium education**		**High education**	
	**Completers**^**a**^ **n (%)**	**Dropouts**^**b**^ **n (%)**	**Completers** **n (%)**	**Dropouts** **n (%)**	**Completers** **n (%)**	**Dropouts** **n (%)**	**Completers** **n (%)**	**Dropouts** **n (%)**	**Completers** **n (%)**	**Dropouts** **n (%)**	**Completers** **n (%)**	**Dropouts** **n (%)**
Active Plus I	925 (67)	459 (33)	410 (68)	191 (32)	514 (66)	266 (34)	425 (67)	209 (33)	170 (64)	97 (36)	313 (68)	149 (32)
Active Plus II	860 (50)	850 (50)	414 (50)	414 (50)	444 (51)	429 (49)	386 (49)	399 (51)	229 (51)	222 (49)	240 (52)	225 (48)
Every Step Counts!	300 (64)	168 (36)	94 (67)	47 (33)	206 (63)	121 (37)	167 (65)	89 (35)	102 (67)	50 (33)	28 (50)	28 (50)
GALM	79 (48)	84 (52)	36 (50)	36 (50)	43 (47)	48 (53)	34 (53)	30 (47)	23 (43)	30 (57)	22 (48)	24 (52)
PACE-Lift	142 (95)	8 (5)	64 (93)	5 (7)	78 (96)	3 (4)	61 (91)	6 (9)	25 (100)	0 (0)	53 (95)	2 (4)
PACE-UP	636 (93)	49 (7)	236 (94)	16 (6)	400 (92)	33 (8)	165 (93)	12 (7)	132 (93)	10 (7)	327 (93)	24 (7)
ProAct65+	422 (60)	282 (40)	154 (59)	107 (41)	268 (60)	175 (40)	177 (54)	153 (46)	148 (68)	70 (32)	89 (63)	54 (38)
PROMOTE	226 (60)	150 (40)	100 (61)	65 (39)	126 (60)	85 (40)	2 (33)	4 (67)	102 (55)	85 (45)	122 (67)	61 (33)
**Study**	**Control group**											
	**Total sample**		**Gender**				**Education**					
			**Males**		**Females**		**Low education**		**Medium education**		**High education**	
	**Completers** **n (%)**	**Dropouts** **n (%)**	**Completers** **n (%)**	**Dropouts** **n (%)**	**Completers** **n (%)**	**Dropouts** **n (%)**	**Completers** **n (%)**	**Dropouts** **n (%)**	**Completers** **n (%)**	**Dropouts** **n (%)**	**Completers** **n (%)**	**Dropouts** **n (%)**
Active Plus I	484 (83)	98 (17)	208 (83)	43 (17)	275 (84)	54 (16)	244 (83)	49 (17)	87 (84)	16 (16)	139 (82)	31 (18)
Active Plus II	305 (75)	104 (25)	144 (71)	60 (29)	161 (79)	44 (21)	148 (74)	51 (26)	82 (77)	25 (23)	68 (76)	22 (24)
Every Step Counts!	95 (62)	59 (38)	35 (60)	23 (40)	57 (59)	39 (41)	54 (60)	36 (40)	24 (59)	17 (41)	13 (59)	9 (41)
GALM	102 (67)	50 (33)	44 (60)	29 (40)	58 (73)	21 (27)	34 (72)	13 (28)	46 (66)	24 (34)	22 (63)	13 (37)
PACE-Lift	138 (93)	10 (7)	65 (94)	4 (6)	73 (92)	6 (8)	49 (91)	5 (9)	19 (95)	1 (5)	68 (96)	3 (4)
PACE-UP	318 (94)	20 (6)	109 (95)	6 (5)	209 (94)	14 (6)	82 (96)	3 (4)	78 (94)	5 (6)	155 (94)	10 (6)
ProAct65+	255 (64)	145 (36)	95 (64)	54 (36)	160 (64)	91 (36)	98 (62)	60 (38)	87 (64)	48 (36)	68 (65)	36 (35)
PROMOTE	124 (76)	40 (24)	60 (86)	10 (14)	64 (68)	30 (32)	4 (67)	2 (33)	66 (71)	27 (29)	54 (83)	11 (17)

^a^Individuals with information on MVPA at T0 (baseline) and T1 (post-intervention follow-up time-point closest to intervention end point)

^b^Individuals with information on MVPA at T0 only

Patterns of dropout in intervention and control groups were also similar across income, area deprivation, and marital status subgroups. Only slight differences in dropout rates in the intervention and control groups were found across these subgroups (Additional file 5).

Information on equity-specific dropout at T2 and baseline MVPA levels can be found in Additional files 5 and 6.

#### General and equity-specific intervention effects

The general intervention effects as well as the gender- and education-specific intervention effects at T1 derived from the fully adjusted models are shown in Table 6. Results of the minimally adjusted models are available in Additional file 7. In Active Plus II, Every Step Counts!, PACE-Lift, PACE-UP, and PROMOTE, the intervention groups did more weekly minutes of MVPA at T1 than the control groups. In Active Plus I, GALM, and ProAct65+, no differences between the groups were found.

**Table 6:** General, gender-, and education-specific intervention effects at T1 (fully adjusted models)

**Study**	**General intervention effect**^**a**^		**Gender-specific intervention effects**^**b**^				
			**Males**		**Females**		***P*-value** intervention*gender interaction
	**n**	**Estimate (95% CI)**	**n**	**Estimate (95% CI)**	**n**	**Estimate (95% CI)**	
Active Plus I	1370	5.3 (− 53.6; 64.3)	603	− 113.5 (− 203.7; −23.3)	767	104.8 (19.9; 189.7)	< 0.001
Active Plus II	1150	196.3 (113.1; 279.4)	554	151.7 (30.7; 272.7)	596	215.3 (92.5; 338.2)	0.465
Every Step Counts!	389	17.4 (6.1; 28.8)	128	24.2 (4.0; 44.4)	261	18.2 (1.7; 34.7)	0.624
GALM	181	28.3 (−43.9; 100.4)	80	71.7 (−37.2; 180.5)	101	−22.3 (− 122.5; 77.9)	0.213
PACE-Lift^d^	275	74.4 (43.7; 105.1)	125	93.7 (50.6; 136.9)	150	58.6 (19.2; 98.0)	0.195
PACE-UP^d^	939	48.0 (30.5; 65.4)	341	45.7 (17.4; 74.0)	598	48.0 (26.9; 69.1)	0.958
ProAct65 + ^e^	667	−4.8 (−48.9; 39.2)	245	−38.6 (−102.5; 25.4)	422	14.1 (−36.9; 65.1)	0.142
PROMOTE^f^	350	7.6 (2.6; 12.6)	160	14.7 (−0.2; 29.6)	190	8.6 (−4.9; 22.2)	0.245
**Study**	**Education-specific intervention effects**^**c**^						
	**Low education**		**Medium education**		**High education**		***P*-value** intervention*education interaction
	**n**	**Estimate (95% CI)**	**n**	**Estimate (95% CI)**	**n**	**Estimate (95% CI)**	
Active Plus I	666	−19.6 (− 104.5; 65.2)	254	5.6 (− 130.8; 142.0)	450	1.0 (− 103.7; 105.8)	0.933
Active Plus II	533	225.4 (103.5; 347.2)	309	213.1 (53.0; 373.1)	308	112.1 (−57.5; 281.6)	0.546
Every Step Counts!	222	17.6 (2.0; 33.3)	126	12.9 (−8.6; 34.4)	41	33.1 (1.2; 65.0)	0.581
GALM	68	87.6 (−28.4; 203.5)	69	29.2 (−92.6; 150.9)	44	−42.7 (− 186.9; 101.6)	0.378
PACE-Lift^d^	110	105.6 (58.4; 152.8)	44	30.1 (−43.0; 103.2)	121	62.5 (17.9; 107.0)	0.164
PACE-UP^d^	247	14.2 (−19.9; 48.3)	210	87.5 (52.5; 122.6)	482	46.5 (22.2; 70.8)	0.012
ProAct65 + ^e^	275	−35.8 (−97.4; 25.7)	235	21.4 (−43.8; 86.6)	157	8.3 (−68.2; 84.9)	0.339
PROMOTE^f^	6	19.7 (−18.7; 58.2)	168	5.8 (−1.4; 13.0)	176	9.5 (2.3; 16.8)	0.633

^a^Models adjusted for minutes of MVPA per week at T0, age in years, gender, and education

^b^Models adjusted for minutes of MVPA per week at T0, age in years, education, and the intervention*education interaction

^c^Models adjusted for minutes of MVPA per week at T0, age in years, gender, and the intervention*gender interaction

^d^Models additionally adjusted for practice, and multi-level adjusted for household as a random effect

^e^Models additionally multi-level adjusted for practice as a random effect

^f^Models additionally adjusted for community, valid wear-time, and season

Overall, we found no consistent pattern of differential intervention effects across the studies. For Active Plus I, an intervention*gender interaction was found, suggesting that the intervention was more effective in increasing weekly minutes of MVPA in females than in males. For PACE-UP, an intervention*education interaction was found, suggesting that the intervention was more effective among medium than high or low educated individuals.

There was no evidence of differential intervention effects by household income, area deprivation, and marital status (Additional file 7). For Active Plus II, at 8 months post-intervention, as well as for PACE-Lift and PACE-UP, at 9 months post-intervention, the intervention groups continued to have higher MVPA levels compared to the control groups, although the differences between the groups were less pronounced when compared to the main analysis (Additional file 7). For Active Plus I, at 8 months post-intervention, and ProAct65+, at 6 months post-intervention, the intervention groups tended to engage in more MVPA than the control groups. There was no evidence of differential intervention effects by any of the socio-demographic indicators examined. For PACE-Lift and PACE-UP, sensitivity analyses of MVPA in bouts of at least 10 min had little impact on the effect estimates and did not change the interpretation (Additional file 7).

#### Meta-analyses

**Fig. 1:**
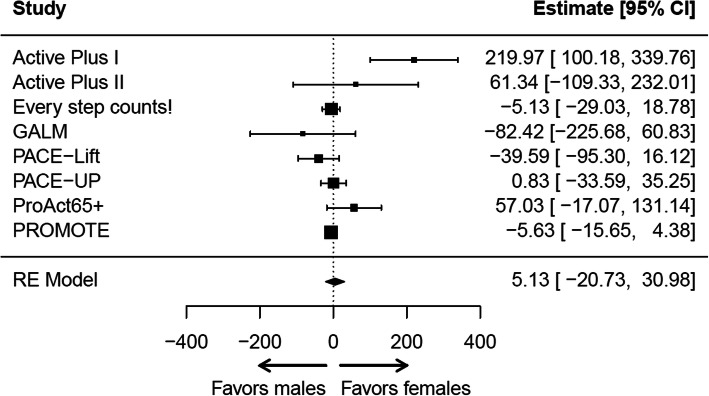
Forest plot of moderated intervention effects through gender at T1

**Fig. 2:**
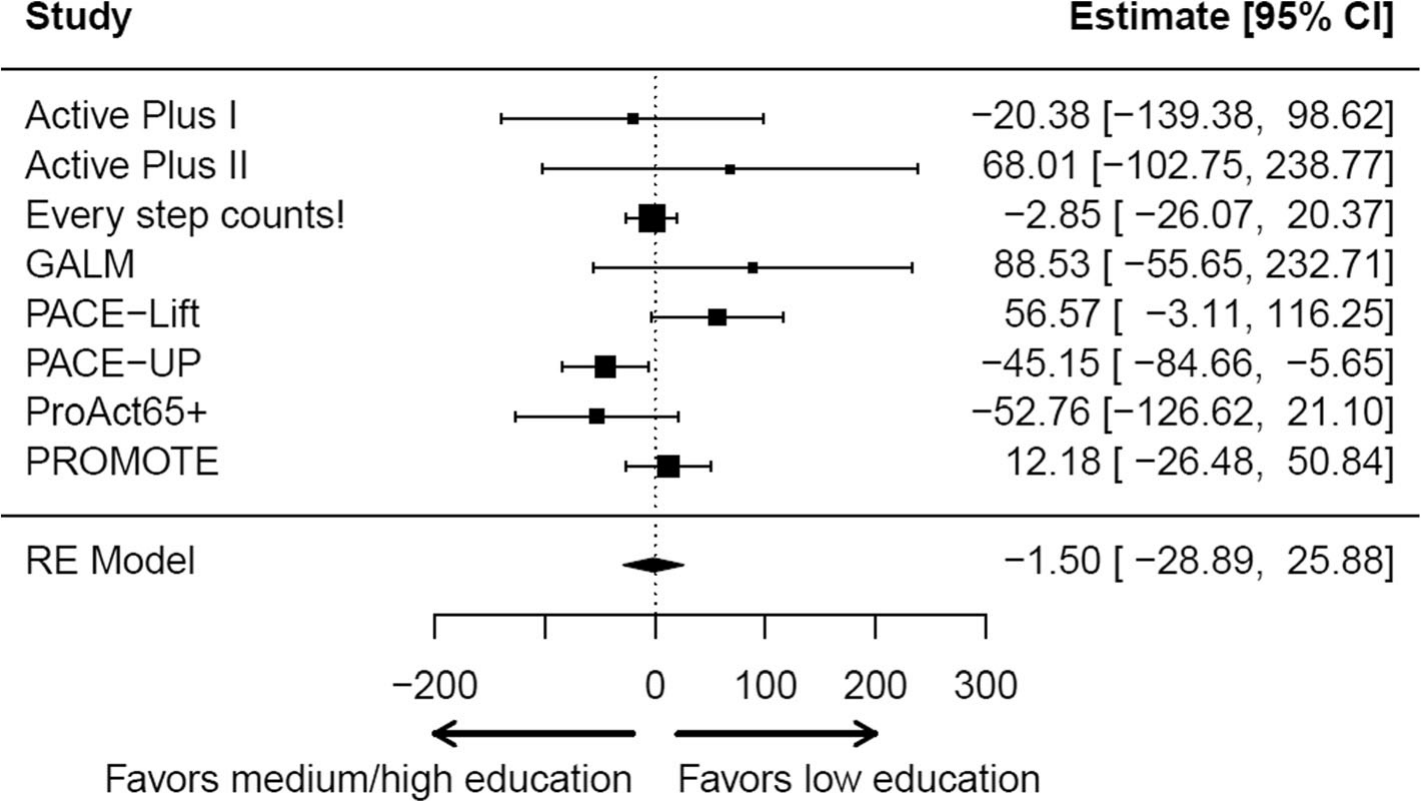
Forest plot of moderated intervention effects through education at T1

Figures 1 and 2 show the estimates for the moderated effects of the interventions through gender and education at T1 for each study (fully adjusted models). The detailed results of the meta-analyses can be found in Additional file 8. The pooled estimates indicated no differences in intervention effects either by gender (5.1 (95% CI: − 20.7 to 31.0), 5321 participants, 8 studies) or by education (− 1.5 (95% CI: − 28.9 to 25.9), 5321 participants, 8 studies). Between study heterogeneity was moderate to high (I^2^ = 64%) for the moderated intervention effects through gender and low to moderate (45%) for the moderated intervention effects through education.

The pooled estimates for the moderated intervention effects through income, area deprivation, and marital status at T1 indicated no differences in intervention effects by these indicators (income: 0.5 (95% CI: − 10.6 to 11.6), I^2^ = 0%, 933 participants, 2 studies); area deprivation: -27.9 (95% CI: − 58.5 to 2.7), I^2^ = 0%, 1802 participants, 3 studies); marital status: 6.9 (95% CI: − 3.3 to 17.1), I^2^ = 0%, 5341 participants, 8 studies).

At T2, the pooled estimates indicated no differences in intervention effects by gender (17.2 (95% CI: − 14.6 to 49.1); I^2^ = 18%; 4348 participants; 5 studies), education (− 13.4 (95% CI: − 54.3 to 27.5); I^2^ = 38%; 4348 participants; 5 studies), area deprivation (− 21.8 (95% CI: − 50.4 to 6.9); I^2^ = 0%; 1887 participants; 3 studies), and marital status (− 1.7 (95% CI: − 36.8 to 33.5), I^2^ = 15%; 4366 participants; 5 studies) (Additional file 8). The sensitivity analysis using partial correlation coefficients lead to comparable results (Additional file 9).

## Discussion

This study sets out a novel equity-specific re-analysis strategy tried out in an international interdisciplinary collaboration. The collaborative experience shows that the novel strategy can be applied to investigate equity-specific effects of existing PA intervention studies in community-dwelling middle-aged and older adults. Across our convenience sample of eight studies, we found no consistent pattern of differential intervention adherence, dropout, and efficacy by gender, education, income, area deprivation, and marital status.

### Strengths and limitations

By applying an equity lens to the analysis of data from PA intervention studies, our strategy offers an approach to filling the gap in knowledge about the impact of these interventions on health inequalities. In contrast to other approaches of equity-specific re-analysis, our strategy proposes the consideration of several SEP indicators instead of focusing on education only [40, 41] or using several SEP indicators interchangeably [38, 39]. Moreover, besides equity-specific intervention effects, the novel strategy includes investigating equity-specific intervention reach, adherence, and dropout, allowing for a comprehensive assessment of equity-specific intervention benefits. The strategy comprises harmonizing the choice and definition of outcomes, exposures, socio-demographic indicators, and statistical analysis strategies across studies as much as possible. Similar to an individual participant data meta-analysis with harmonized data [86], harmonizing each study’s individual participant data according to jointly developed criteria allows to examine interaction and subgroup effects in a setting that goes far beyond conventional meta-analyses of published data. Our experience shows that a collaborative approach bringing together researchers from primary studies and regular exchange within the collaboration is important as it allows discussing methodological issues and re-analysis findings in-depth. In this regard, the internal knowledge about the studies contributed by the responsible researchers is of particular importance as this far exceeds the information which can be extracted from publications.

A limitation of our study is that we applied the equity-specific re-analysis strategy to a convenience sample of studies. Therefore, our re-analysis results cannot be considered generalizable. To provide a comprehensive summary of the current evidence on equity-specific effects of individual-level PA interventions among middle-aged and older adults, it would be relevant to apply our strategy to a larger, representative sample of studies identified in a systematic literature search. The small sample of eight studies also prevented us from conducting meta-regression [79] which we would recommend to take into consideration when applying our strategy to a larger group of studies.

Our experience shows that there are certain limitations and challenges to using our strategy. First, data harmonization may result in a loss of data detail. For instance, in studies with several intervention groups, these groups were combined to create a single pair-wise comparison. Moreover, weekly minutes of MVPA was used as the outcome, without differentiating between different intensities, domains, or types of PA, and data transformations carried out in some studies’ original analysis were not used here. As a result, for some studies, the general intervention effects observed in the re-analysis diverged from the original study results. However, without data harmonization, no formal meta-analysis would be possible, thus losing the opportunity to gain precision in estimating effects of interest. It will be important for future studies to weigh the advantages against the disadvantages of data harmonization from a public health perspective.

A second issue relates to the fact that, because information on socio-demographic indicators for non-participants is often not available in studies of health promotion interventions, assessing inequalities in intervention reach is not straightforward. Instead, census data could be consulted and the study population could be compared with the targeted population of each study, considering the studies’ specific eligibility criteria. Our experience shows, however, that finding suitable census data can be complicated. We would recommend at least calculation (or estimation) of overall response rates and investigation of the socio-demographic characteristics of the study sample. In our convenience sample of eight intervention studies, most included rather equal numbers of females and males, with some studies reaching slightly more women than men. The percentage of individuals with low education, however, varied considerably between the studies, partly as a result of different recruitment procedures. In one study, the percentage was particularly low (2%), suggesting that the intervention reached predominantly those at the upper end of the socio-economic spectrum.

A third aspect involves the comprehensiveness with which equity-specific intervention effects can be analyzed. This depends particularly on the availability of information on relevant socio-demographic indicators, the comparability of socio-demographic indicators across studies, as well as the size and diversity of study samples. In our sample of PA intervention studies, information on gender, education, and marital status were available in all studies and could be defined in a similar manner, but information on income and area deprivation were available in only two and three studies, respectively. Ethnicity, which was assessed in three studies, was not considered as a socio-demographic indicator due to differing ethnic compositions in the study populations. The fact that not all studies were heterogeneous in terms of education might have limited the ability to identify education-specific intervention effects. Moreover, gender could be defined only as female versus male without further operationalizing gender according to gender theoretical concepts [71]. We were also only able to consider differential intervention effects with regard to a single dimension of inequalities, such as SEP, whereas potential differential intervention effects across intersections of multiple dimensions [68, 69], such as SEP and gender, were not considered.

A fourth issue concerns the handling of missing data. For our sample of PA intervention studies, we did not address the risk of attrition bias through sensitivity analyses using multiple imputation (MI) methods which future studies applying the strategy may consider. Because MI methods would have varied between the studies posing problems for interpretation, we decided to not impute missing outcome data. Moreover, in half the studies, MI sensitivity analyses were conducted in their original analyses providing evidence that their results were not biased by missing outcome data and dropout rates were found to be comparable across socio-demographic subgroups for most of the studies. In such cases, the risk of having under- or overestimated differential intervention effects due to differential dropout can be considered rather low. Fifth, in this regard, it also becomes clear that the high risk and serious risk of bias judgements of general intervention effect estimates, for example, due to differing proportions of missing outcome data in the intervention and control groups, must not necessarily apply to equity-specific intervention effect estimates. Existing risk of bias tools, such as the RoB 2.0 and the ROBINS-I, are designed to assess the risk of bias in estimates of general intervention effects, whereas estimates of equity-specific intervention effects are not considered. There is a need for tools that enable adequate assessments of the risk of bias in estimates of equity-specific intervention effects.

A sixth point is that the ability to investigate potential changes in equity-specific intervention effects over time may be limited because few studies of PA interventions have evaluated long-term intervention effects [56]. For our sample of PA intervention studies, we identified PA at the post-intervention follow-up time point closest to the intervention end point as the primary outcome as this criterion was met by all studies. Six, eight, or nine months post intervention, respectively, were used as a secondary outcome, considering the data of five studies. We strongly recommend, where sufficient data is available, to investigate equity-specific differences in intervention effects over a longer time period.

Finally, a collaborative procedure such as ours requires temporal, personnel, and financial resources. Future studies that aim to apply the strategy to existing study data must take these resources into account and should rate the costs against the expected benefit from a public health perspective.

Equity-specific re-analysis can help build the needed evidence base on the effects of public health interventions on health inequalities in the short term. However, there are some limitations of post-hoc analyses [33]. As discussed above, the comprehensiveness with which equity-specific intervention effects can be analyzed may be limited. Moreover, the probability of false-negative results (i.e., failing to detect a true differential intervention effect) may be increased due to insufficient statistical power [87]. Therefore, planning equity-specific effect analysis a-priori should be the long-term objective. Future studies should ideally consider inequalities already in the planning of data collection tools and sample size calculations. Particularly the latter is an ambitious goal which may not always be feasible because the increase in sample size required to detect differential intervention effects may be considerable [87].

## Conclusions

The collaborative experience shows that the novel re-analysis strategy can be applied to investigate equity-specific effects of existing PA interventions. We encourage future studies to exploit the potential for equity-specific effect analysis by applying the strategy to representative samples of existing study data ensuring sufficient representation of ‘hard to reach’ groups. Ability to share individual participant data in line with open science principles and willingness to share detailed knowledge of study characteristics among primary study authors is of particular relevance. This will help extend the limited evidence required for the design and prioritization of future interventions that will be most likely to reduce health inequalities.

## Supplementary Information

**Additional file 1.** Characteristics of intervention studies included in the collaboration. This file contains a table in which characteristics of the included intervention studies are summarized.

**Additional file 2.** Definition of variables on income and area deprivation. This file contains details of how the variables on income and area deprivation were defined.

**Additional file 3.** Results of Risk of Bias assessment. This file contains the detailed results of the risk of bias assessment.

**Additional file 4.** Equity-specific intervention adherence. This file contains the detailed results of the equity-specific intervention adherence analyses (main and secondary analysis).

**Additional file 5.** Equity-specific dropout. This file contains the results of the secondary analysis on equity-specific dropout.

**Additional file 6.** Equity-specific baseline MVPA levels. This file contains information on equity-specific baseline MVPA levels (main and secondary analysis).

**Additional file 7.** General and equity-specific intervention effects. This file contains the results of the general, gender-, and education-specific intervention effect analysis at T1 derived from the minimally adjusted models as well as the results of the secondary and sensitivity analyses on general and equity-specific intervention effects.

**Additional file 8.** Detailed results of the meta-analyses using raw coefficients. This file contains the complete output of the random-effects meta-analysis (main and secondary analysis) using raw coefficients.

**Additional file 9.** Detailed results of the meta-analyses using partial correlation coefficients. This file contains the complete output of the random-effects meta-analysis (main and secondary analysis) using partial correlation coefficients.

## Data Availability

Aggregated data supporting the conclusions of this article are included within the article and additional files. Individual participant data remain under ownership of the researchers from the contributing studies.

## Abbreviations

CI: Confidence interval
IMD: Index of multiple deprivation
ISCED: International Standard Classification of Education
MI: Multiple imputation
MVPA: Moderate-to-vigorous physical activity
PA: Physical activity
RoB 2.0: Revised Cochrane risk-of-bias tool for randomized trials
ROBINS-I: Risk-of-bias tool for non-randomized studies of interventions
SD: Standard deviation
SEP: Socio-economic position
