# Supplementary material for: Equity-specific effects of interventions to promote physical activity among middle-aged and older adults: results from applying a novel equity-specific re-analysis strategy
Source: Int J Behav Nutr Phys Act. 2021 May 17;18:65. doi: 10.1186/s12966-021-01131-w (PMC8130354; doi:10.1186/s12966-021-01131-w)
Supplement: Supplementary file 1 — Additional file 1. Characteristics of intervention studies included in the collaboration. This file contains a table in which characteristics of the included intervention studies are summarized. [file 12966_2021_1131_MOESM1_ESM.docx]

Additional file 1: Characteristics of intervention studies included in the collaboration

| **Study name (country)** | **Study design (*n* at baseline, participants’ characteristics)*,* follow-up** | **Intervention characteristics** | **Main PA outcome measure** | **Socio-demographic indicators** |
| --- | --- | --- | --- | --- |
| Active Plus I  (The Netherlands) | Cluster RCT (*n* = 1971, ≥50 years), 2 and 8 months post-intervention | IG1: Three tailored letters; personalized PA advice targeting psychosocial determinants during 4 months.  IG2: Intervention of IG1 plus tailored environmental information. CG: Wait-listed | Self-report (Dutch Squash): leisure walking and cycling, transport walking and cycling, sports, gardening, housework, doing odd jobs performed with moderate- and vigorous-intensity | Gender, education, age, marital status |
| Active Plus II  (The Netherlands) | Cluster RCT (*n* = 2140, ≥50 years), 2 and 8 months post-intervention | IG1: Three tailored letters; personalized PA advice targeting psychosocial determinants during 4 months. IG2: Web-based version of intervention of IG1. IG3: Intervention of IG1 plus tailored environmental information. IG4: Web-based version of intervention of IG3. CG: Wait-listed | Self-report (Dutch Squash): leisure walking and cycling, transport walking and cycling, sports, gardening, housework, doing odd jobs performed with moderate- and vigorous-intensity | Occupation, gender, education, age, marital status |
| Every Step Counts!  (Belgium) | Controlled before and after study (*n* = 623, ≥55 years), immediately post-intervention | IG: 10-week pedometer-defined walks in weekly walking schedules (fitness tailored and structured in walking load). CG: Wait-listed | Self-report (adapted version GLTEQ): low, moderate, and vigorous intensity PA | Gender, education, social capital*, age, marital status |
| GALM  (The Netherlands) | Cluster-randomized trial (*n* = 315, ≥55 years, physically inactive), immediately post-intervention | IG: Weekly sessions emphasizing tailored moderate-intensity recreational sports activities over 15 weeks. CG: Wait-listed | Self-report (Voorrips PA questionnaire; compendium of physical activities by Ainsworth et al.): recreational sports activities, leisure-time physical activities | Gender, education, age  marital status, living situation |
| PACE-Lift  (UK) | Cluster RCT (*n* = 298, 60-75 years); immediately, 9, and 45 months post-intervention | IG: Four tailored primary care nurse-delivered PA consultations over 3 months, pedometer and accelerometer feedback, individual PA diary and plan. CG: Usual care | Objective (accelerometer) | Area deprivation, race/ethnicity, occupation, gender, education, social capital*, age, marital status, living situation |
| PACE-UP  (UK) | Cluster RCT (*n* = 1023, 45-75 years, physically inactive); immediately, 9, and 33 months post-intervention | IG1: Pedometers, patient handbook, PA diary including individual walking plan over 3 months. IG2: Intervention of IG1 plus 3 tailored practice nurse PA consultations. CG: Usual care | Objective (accelerometer) | Area deprivation, race/ethnicity, occupation, gender, education, social capital*, age, marital status, living situation |
| ProAct65+  (UK) | Cluster RCT (*n* = 1254, ≥65 years); immediately, 6, 12, 18, and 24 months post-intervention | IG1: Home-based exercise program over 6 months comprising exercises, walking plan, visits of trained peer mentors. IG2: Community-based exercise program over 6 months comprising instructor-delivered group exercise class, home exercise, advice to walk. CG: Usual care | Self-report (CHAMPS): dancing; golfing, tennis, skating, housework, gardening, jogging/running, walking/hiking; cycling; aerobic machines; exercising in water; swimming; aerobics; strength training; basketball, soccer, or racquetball | Area deprivation, race/ethnicity, occupation, gender, education, income, age, marital status, living situation |
| PROMOTE (Germany) | RCT (*n* = 589, 65-79 years), immediately post-intervention | IG1: Tailored exercise plan; website with PA diary, online-forum, social features; weekly group meetings over 10 weeks. IG2: Intervention of IG1 plus PA tracker. CG: Wait-listed | Objective (accelerometer) | Race/ethnicity, occupation, gender, education, income, social capital*, age, marital status, living situation |

IG = intervention group; CG = control group; PA = physical activity; SQUASH = Dutch Short Questionnaire to Assess Health Enhancing Physical Activity; GLTEQ = Godin Leisure-Time Questionnaire; CHAMPS = Community Healthy Activities Model Program for Seniors. * Social capital is considered a generic term covering various operationalizations.
