# Supplementary material for: Equity-specific effects of interventions to promote physical activity among middle-aged and older adults: results from applying a novel equity-specific re-analysis strategy
Source: Int J Behav Nutr Phys Act. 2021 May 17;18:65. doi: 10.1186/s12966-021-01131-w (PMC8130354; doi:10.1186/s12966-021-01131-w)
Supplement: Supplementary file 2 — Additional file 2. Definition of variables on income and area deprivation. This file contains details of how the variables on income and area deprivation were defined. [file 12966_2021_1131_MOESM2_ESM.docx]

**Additional file 2: Definition of variables on income and area deprivation**

For the variables on income and area deprivation, in each study with available information (PACE-Lift, PACE-UP, ProAct65+, PROMOTE), tertiles were defined in terms of the distribution in the study’s specific data set. This resulted in two variables with the categories “Low”, “Medium”, and “High” each for income and area deprivation. The detailed variable definitions are provided in the following table.

|  | **PACE-Lift** | **PACE-UP** | **ProAct65+** | **PROMOTE** |
| --- | --- | --- | --- | --- |
| **Income** | | | | |
| Low | NA | NA | <=£12000 pa+ | <1500€ per month** |
| Medium | NA | NA | >£12000 & <=£20000 | 1500€ <= 2150€ |
| High | NA | NA | >£20000 | >2150€ |
| **Area deprivation** | | | | |
| High | Range 5682 - 26440 | Range 2656 - 14369 | Practice deprivation score*>22 | NA |
| Medium | Range 26455 - 30547 | Range 14439 - 22142 | Score >15.5 & <=22 | NA |
| Low | Range 30618 - 32239 | Range 22216 - 32408 | Score <=15.5 | NA |

NA = not applicable. + The questionnaire was administered at recruitment, from 2009-2011. * The Index for Multiple Deprivation for 2007 was used, as applied to the “lower super output areas” in which the family practice was located. This may not reflect the living circumstances of all individual patients belonging to the practice (Noble M, Wright G, Dibben C, Smith GAN, McLennan D, Anttila C, et al. The English Indices of Deprivation. London: Office of the Deputy Prime Minister; 2004. p. 180.). ** Need-weighted household income per capita was derived from the number of household members and the monthly household income according to the German Microcensus (Boehle M. Armutsmessung mit dem Mikrozensus: methodische Aspekte und Umsetzung für Querschnitts- und Trendanalysen. (GESIS Papers 2015/16). Köln: GESIS - Leibniz-Institut für Sozialwissenschaften; 2015).
