# Supplementary material for: Equity-specific effects of interventions to promote physical activity among middle-aged and older adults: results from applying a novel equity-specific re-analysis strategy
Source: Int J Behav Nutr Phys Act. 2021 May 17;18:65. doi: 10.1186/s12966-021-01131-w (PMC8130354; doi:10.1186/s12966-021-01131-w)
Supplement: Supplementary file 3 — Additional file 3. Results of Risk of Bias assessment. This file contains the detailed results of the risk of bias assessment. [file 12966_2021_1131_MOESM3_ESM.docx]

**Additional file 3: Results of Risk of Bias assessment**

Of the seven randomized studies, four were judged to be at low risk of bias arising from the randomization process. Active Plus I, Active Plus II, and GALM were judged to be at high risk as the allocation sequence was not concealed. All seven studies were judged to be at low risk of bias due to deviations from interventions. Four studies were judged to be at low risk of bias due to missing outcome data because sensitivity analyses using multiple imputation (MI) methods were conducted in the original analyses providing evidence that the re-analysis result was not biased by missing outcome data. For Active Plus I, GALM, and PROMOTE, there was no such evidence available, and higher rates of dropout were observed in the intervention groups, which could indicate that missingness in the outcome depends on its true value. Because they measured PA outcomes objectively, PACE-Lift, PACE-UP, and PROMOTE were judged to be at low risk of bias in measurement of the outcome. Active Plus I, Active Plus II, GALM, and ProAct65+ were considered to be at high risk, as knowledge of intervention status was likely to influence the outcome assessment (self-reported PA outcome).

The non-randomized study Every Step Counts! was judged to be at moderate risk of bias due to confounding because confounding is to be expected, but important confounding domains were reliably and validly measured and appropriately controlled for. It was judged to be at low risk of bias in selection of participants into the study, in classification of interventions, and due to deviations from intended interventions. Because rates of dropout were similar for intervention and control groups but reasons for missing participants were mainly unknown, the study was judged to be at moderate risk of bias due to missing outcome data. Moreover, it was judged to be at serious risk of bias in measurement of the outcome because knowledge of intervention status could have influenced the outcome assessment (self-reported PA outcome). All eight studies were judged to be at low risk of bias in selection of the reported result because the re-analysis was conducted and reported in accordance with pre-specified criteria. Answers to all signaling questions are presented in the following two tables.

**Risk of** **bias assessment using the revised Cochrane risk-of-bias tool for randomized trials (RoB 2.0)**

| **Study** | **Risk of bias domain** | | | | | **Overall risk of bias*** |
| --- | --- | --- | --- | --- | --- | --- |
|  | **Randomisation process** | **Deviations from intended interventions** | **Missing**  **outcome data** | **Measurement of the outcome** | **Selection of the reported**  **result** |  |
| Active Plus I | High risk | Low risk | High risk | High risk | Low risk | High risk |
|  | **Question 1.1** The allocation sequence was random (use of computer-generated numbers)  **Question 1.2** The allocation sequence was not concealed  **Question 1.3** There were no baseline imbalances to suggest a problem with the randomization | **Question 2.1** Participants were aware of intervention  **Question 2.2** Intervention deliverers were not aware of intervention  **Question 2.3** Probably no deviations from intended interventions  **Questions 2.4-2.5** NA  **Question 2.6** Complete case intention-to-treat analysis | **Question 3.1** Outcome data were not available for all participants  **Question 3.2** Probably no evidence that the results are robust to the presence of missing outcome data  **Question 3.3** Missingness in the outcome could depend on its true value  **Question 3.4** Missingness in the outcome is likely to depend on its true value because there are differences between intervention groups in the proportions of missing outcome data. | **Question 4.1** The method of measuring the outcome was not inappropriate  **Question 4.2** Methods of outcome measurement were comparable across groups  **Question 4.3** Outcome assessors were aware of the intervention (participant-reported outcome)  **Question 4.4-4.5** Assessment of the outcome is likely to have been influenced by knowledge of intervention received. | **Question 5.1** The re-analysis was conducted and reported in accordance with a pre-specified re-analysis protocol  **Questions 5.2-5.3** Analyses and reports are consistent with the pre-specified re-analysis plan |  |
| Active Plus II | High risk | Low risk | Low risk | High risk | Low risk | High risk |
|  | **Question 1.1** The allocation sequence was random (use of computer-generated numbers)  **Question 1.2** The allocation sequence was not concealed  **Question 1.3** There were no baseline imbalances to suggest a problem with the randomization | **Question 2.1** Participants were aware of intervention  **Question 2.2** Intervention deliverers were not aware of intervention  **Question 2.3** Probably no deviations from intended interventions  **Questions 2.4-2.5** NA  **Question 2.6** Complete case intention-to-treat analysis | **Question 3.1** Outcome data were not available for all participants  **Question 3.2** There is evidence that the results are robust to the presence of missing data  **Questions 3.3-3.4** NA | **Question 4.1** The method of measuring the outcome was not inappropriate  **Question 4.2** Methods of outcome measurement were comparable across groups  **Question 4.3** Outcome assessors were aware of the intervention (participant-reported outcome)  **Question 4.4-4.5** Assessment of the outcome is likely to have been influenced by knowledge of intervention received. | **Question 5.1** The re-analysis was conducted and reported in accordance with a pre-specified re-analysis protocol  **Questions 5.2-5.3** Analyses and reports are consistent with the pre-specified re-analysis plan |  |
| GALM | High risk | Low risk | High risk | High risk | Low risk | High risk |
|  | **Question 1.1** The allocation sequence was random (group randomized design)  **Question 1.2** The allocation sequence was not concealed  **Question 1.3** There were no baseline imbalances to suggest a problem with the randomization | **Question 2.1** Participants were aware of intervention  **Question 2.2** Intervention deliverers were aware of intervention  **Question 2.3** No deviations from intended interventions  **Question 2.4**- **2.5** NA  **Question 2.6** Complete case intention-to-treat analysis | **Question 3.1** Outcome data were not available for all participants  **Question 3.2** Probably no evidence that the results are robust to the presence of missing outcome data  **Question 3.3** Missingness in the outcome could depend on its true value  **Question 3.4** Missingness in the outcome is likely to depend on its true value because there are differences between intervention groups in the proportions of missing outcome data. | **Question 4.1** The method of measuring the outcome was not inappropriate  **Question 4.2** Methods of outcome measurement were comparable across groups  **Question 4.3** Outcome assessors were aware of the intervention (participant-reported outcome)  **Question 4.4-4.5** Assessment of the outcome is likely to have been influenced by knowledge of intervention received | **Question 5.1** The re-analysis was conducted and reported in accordance with a pre-specified re-analysis protocol  **Questions 5.2-5.3** Analyses and reports are consistent with the pre-specified re-analysis plan |  |
| PACE-Lift | Low risk | Low risk | Low risk | Low risk | Low risk | Low risk |
|  | **Question 1.1** The allocation sequence was random (use of randomization software)  **Question 1.2** The allocation sequence was concealed (use of Nottingham Clinical Trials Unit internet randomisation service)  **Question 1.3** There were no baseline imbalances to suggest a problem with the randomization | **Question 2.1** Participants were aware of intervention  **Question 2.2** Intervention deliverers were aware of intervention  **Question 2.3** No deviations from intended interventions  **Questions 2.4-2.5** NA  **Question 2.6** Complete case intention-to-treat analysis | **Question 3.1** Outcome data were not available for all participants  **Question 3.2** There is evidence that the results are robust to the presence of missing data  **Questions 3.3-3.4** NA | **Question 4.1** The method of measuring the outcome was not inappropriate  **Question 4.2** Methods of outcome measurement were comparable across groups  **Question 4.3** Outcome assessors were aware of the intervention  **Questions 4.4-4.5** Assessment of the outcome was most likely not influenced by knowledge of intervention received (objectively measured outcome not relying on assessor interpretation). | **Question 5.1** The re-analysis was conducted and reported in accordance with a pre-specified re-analysis protocol  **Questions 5.2-5.3** Analyses and reports are consistent with the pre-specified re-analysis plan |  |
| PACE-UP | Low risk | Low risk | Low risk | Low risk | Low risk | Low risk |
|  | **Question 1.1** The allocation sequence was random (use of Stata's random number generator and Kings College Clinical Trials Unit internet service)  **Question 1.2** The allocation sequence was concealed (Kings College Clinical Trials Unit internet service)  **Question 1.3** There were no baseline imbalances to suggest a problem with the randomization | **Question 2.1** Participants were aware of intervention  **Question 2.2** Intervention deliverers were aware of intervention  **Question 2.3** No deviations from intended interventions  **Questions 2.4-2.5** NA  **Question 2.6** Complete case intention-to-treat analysis | **Question 3.1** Outcome data were not available for all participants  **Question 3.2** There is evidence that the results are robust to the presence of missing data  **Questions 3.3-3.4** NA | **Question 4.1** The method of measuring the outcome was not inappropriate  **Question 4.2** Methods of outcome measurement were comparable across groups  **Question 4.3** Outcome assessors were aware of the intervention  **Questions 4.4-4.5** Assessment of the outcome was most likely not influenced by knowledge of intervention received (objectively measured outcome not relying on assessor interpretation). | **Question 5.1** The re-analysis was conducted and reported in accordance with a pre-specified re-analysis protocol  **Questions 5.2-5.3** Analyses and reports are consistent with the pre-specified re-analysis plan |  |
| ProAct65+ | Low risk | Low risk | Low risk | High risk | Low risk | High risk |
|  | **Question 1.1** The allocation sequence was random (use of computer-generated random number tables, embedded in a computer program for minimization)  **Question 1.2** The allocation sequence was concealed  **Question 1.3** There were no baseline imbalances to suggest a problem with the randomization | **Question 2.1** Participants were aware of intervention  **Question 2.2** Intervention deliverers were aware of intervention  **Question 2.3** Probably no deviations from intended interventions  **Questions 2.4-2.5** NA  balanced between groups  **Question 2.6** Complete case intention-to-treat analysis | **Question 3.1** Outcome data were not available for all participants  **Question 3.2** There is evidence that the results are robust to the presence of missing data  **Questions 3.3-3.4** NA | **Question 4.1** The method of measuring the outcome was not inappropriate  **Question 4.2** Methods of outcome measurement were comparable across groups  **Question 4.3** Outcome assessors were aware of the intervention (participant-reported outcome)  **Question 4.4-4.5** Assessment of the outcome is likely to have been influenced by knowledge of intervention received. | **Question 5.1** The re-analysis was conducted and reported in accordance with a pre-specified re-analysis protocol  **Questions 5.2-5.3** Analyses and reports are consistent with the pre-specified re-analysis plan |  |
| PROMOTE | Low risk | Low risk | High risk | Low risk | Low risk | High risk |
|  | **Question 1.1** The allocation sequence was random (participants’ choice of date of baseline assessment determined group assignment)  **Question 1.2** The allocation sequence was concealed  **Question 1.3** There were no baseline imbalances to suggest a problem with the randomization | **Question 2.1** Participants were aware of intervention  **Question 2.2** Intervention deliverers were aware of intervention  **Question 2.3** No deviations from intended interventions  **Questions 2.4-2.5** NA  **Question 2.6** Complete case intention-to-treat analysis | **Question 3.1** Outcome data were not available for all participants  **Question 3.2** Probably no evidence that the results are robust to the presence of missing outcome data  **Question 3.3** Missingness in the outcome could depend on its true value  **Question 3.4** Missingness in the outcome is likely to depend on its true value because there are differences between intervention groups in the proportions of missing outcome data. | **Question 4.1** The method of measuring the outcome was not inappropriate  **Question 4.2** Methods of outcome measurement were comparable across groups  **Question 4.3** Outcome assessors were aware of the intervention  **Questions 4.4-4.5** Assessment of the outcome was most likely not influenced by knowledge of intervention received (objectively measured outcome not relying on assessor interpretation). | **Question 5.1** The re-analysis was conducted and reported in accordance with a pre-specified re-analysis protocol  **Questions 5.2-5.3** Analyses and reports are consistent with the pre-specified re-analysis plan |  |

NA = not applicable. * Low risk of bias: The study is judged to be at low risk of bias for all domains; Some concerns: The study is judged to raise some concerns in at least one domain, but not to be at high risk of bias for any domain; High risk of bias: The study is judged to be at high risk of bias in at least one domain.

**Risk of** **bias assessment using the ROBINS-I risk-of-bias tool for non-randomized studies of interventions**

| **Study** | **Risk of bias domain** | | | | | | | **Overall risk of bias*** |
| --- | --- | --- | --- | --- | --- | --- | --- | --- |
|  | **Confounding** | **Selection of participants into the study** | **Classification of interventions** | **Deviations from intended interventions** | **Missing data** | **Measurement of**  **outcomes** | **Selection of the reported**  **result** |  |
| Every Step Counts! | Moderate risk | Low risk | Low risk | Low risk | Moderate risk | Serious | Low risk | Serious |
|  | **Question 1.1** Confounding expected  **Question 1.2** The analysis was not based on splitting participants’ follow up time according to intervention received  **Question 1.3** NA  **Question 1.4** All known important confounding domains controlled for  **Question 1.5** The variables adjusted for are valid and reliable measures of the confounding domains  **Question 1.6** No postintervention variables that could have been affected by the intervention were controlled for  **Questions 1.7-1.8** NA | **Question 2.1** Selection of participants was not based on participant characteristics observed after the start of intervention  **Questions 2.2-2.3** NA  **Question 2.4** Start of follow-up and start of intervention coincide for all participants  **Question 2.5** NA | **Question 3.1** Intervention groups were clearly defined  **Question 3.2** The information used to define intervention groups was recorded at the start of the intervention  **Question 3.3** Classification of intervention status has most likely not been affected by knowledge of the outcome or risk of the outcome | **Question 4.1** No deviations from intended interventions  **Question 4.2** NA | **Question 5.1** Outcome data were not available for all participants  **Question 5.2** Participants were not excluded due to  missing data on intervention status  **Question 5.3** Participants were excluded due to missing data on other variables needed for the analysis  **Question 5.4** Balanced missings in intervention and control group, but reasons for missing data were mostly unknown  **Question 5.5** Probably no evidence that the results are robust to the presence of missing outcome data | **Question 6.1** Outcome measure could have been influenced by knowledge of intervention received (participant-reported outcome)  **Question 6.2** Outcome assessors were aware of the intervention (participant-reported outcome)  **Question 6.3** Methods of outcome measurement were comparable across groups  **Question 6.4** No information regarding possible systematic errors in measurements of the outcome available | **Questions 7.1-7.3** Analyses and reports are consistent with the pre-specified re-analysis plan |  |

NA = not applicable. * Low risk of bias: The study is judged to be at low risk of bias for all domains; Moderate risk of bias: The study is judged to be at low or moderate risk of bias for all domains; Serious risk of bias: The study is judged to be at serious risk of bias in at least one domain, but not at critical risk of bias in any domain; Critical risk of bias: The study is judged to be at critical risk of bias in at least one domain.
