# Supplementary material for: Equity-specific effects of interventions to promote physical activity among middle-aged and older adults: results from applying a novel equity-specific re-analysis strategy
Source: Int J Behav Nutr Phys Act. 2021 May 17;18:65. doi: 10.1186/s12966-021-01131-w (PMC8130354; doi:10.1186/s12966-021-01131-w)
Supplement: Supplementary file 4 — Additional file 4. Equity-specific intervention adherence. This file contains the detailed results of the equity-specific intervention adherence analyses (main and secondary analysis). [file 12966_2021_1131_MOESM4_ESM.docx]

**Additional file 4: Equity-specific intervention adherence**

| **Study** | **Measure of adherence** | **Gender** | | | | **Education** | | | | | |
| --- | --- | --- | --- | --- | --- | --- | --- | --- | --- | --- | --- |
|  |  | **Males** | | **Females** | | **Low education** | | **Medium education** | | **High education** | |
|  |  | **n(/N)** | **%** | **n(/N)** | **%** | **n(/N)** | **%** | **n(/N)** | **%** | **n(/N)** | **%** |
| Active Plus II | Tailored advice 1 completely read | 405/442 | 92 | 452/477 | 95 | 395/425 | 93 | 212/229 | 93 | 250/267 | 94 |
|  | Tailored advice 2 completely read | 334/440 | 76 | 368/473 | 78 | 326/422 | 77 | 177/228 | 78 | 200/265 | 76 |
|  | Tailored advice 3 completely read | 281/332 | 85 | 328/369 | 89 | 274/314 | 87 | 150/175 | 86 | 184/211 | 87 |
|  | Role model stories read/watched | 213/433 | 49 | 262/469 | 56 | 215/416 | 52 | 121/226 | 54 | 142/262 | 54 |
|  | Intrinsic motivation assignment used | 131/436 | 30 | 146/470 | 31 | 130/419 | 31 | 69/225 | 31 | 77/263 | 29 |
|  | Formulate action plans used | 27/440 | 6 | 37/463 | 8 | 36/416 | 9 | 11/225 | 5 | 18/263 | 7 |
|  | Formulate coping plans used | 23/322 | 7 | 35/356 | 10 | 40/302 | 13 | 8/173 | 5 | 10/202 | 5 |
|  | Logbook used | 22/325 | 7 | 29/361 | 8 | 24/307 | 8 | 12/172 | 7 | 15/207 | 7 |
|  | Additional information chronic limitation read | 49/226 | 22 | 75/233 | 32 | 70/220 | 32 | 28/112 | 25 | 25/128 | 20 |
|  | Walk and cycling routes used | 45/203 | 22 | 47/232 | 20 | 49/221 | 22 | 18/108 | 17 | 25/108 | 23 |
|  | Extra walk and cycling route used | 32/154 | 21 | 41/177 | 23 | 42/164 | 26 | 14/80 | 18 | 17/89 | 19 |
|  | Sport information used | 11/204 | 5 | 16/231 | 7 | 17/219 | 8 | 2/109 | 2 | 8/110 | 7 |
|  | Explanation how to plan a cycling route used | 53/153 | 35 | 62/177 | 35 | 57/164 | 35 | 30/79 | 38 | 28/89 | 32 |
|  | PA home exercises used | 63/192 | 33 | 89/219 | 41 | 80/209 | 38 | 39/103 | 38 | 32/101 | 32 |
|  | Additional exercises used | 49/153 | 32 | 68/177 | 38 | 62/164 | 38 | 27/79 | 34 | 28/89 | 32 |
|  | Neighborhood map used | 51/154 | 33 | 46/177 | 26 | 54/164 | 33 | 19/80 | 24 | 24/89 | 27 |
|  | Post cards / e-cards used | 2/203 | 1 | 9/232 | 4 | 7/219 | 3 | 2/109 | 2 | 2/109 | 2 |
| GALM | Mean attendance rate of 15 intervention sessions | 36 | 83 | 43 | 77 | 34 | 85 | 23 | 76 | 22 | 77 |
| PACE-UP | PA diary returned after 12-week intervention | 201/236 | 85 | 339/400 | 85 | 137/165 | 83 | 121/132 | 92 | 271/327 | 83 |
|  | Pedometer used at every day or most days | 191/214 | 89 | 312/364 | 86 | 125/150 | 83 | 116/125 | 93 | 254/295 | 86 |
| PROMOTE | Web-based PA diary used | 84/97 | 87 | 101/121 | 83 | 2/2 | 100 | 86/99 | 87 | 97/117 | 83 |
|  | Group meetings attended | 67/98 | 68 | 101/125 | 81 | 2/2 | 100 | 79/102 | 77 | 87/119 | 73 |
|  | Website used (FitBit) | 35/38 | 92 | 54/60 | 90 | 2/2 | 100 | 43/46 | 93 | 44/50 | 88 |

| **Study** | **Measure of adherence** | **Income** | | | | | | **Area deprivation** | | | | | | | **Marital status** | | | |
| --- | --- | --- | --- | --- | --- | --- | --- | --- | --- | --- | --- | --- | --- | --- | --- | --- | --- | --- |
|  |  | **Low**  **income** | | **Medium income** | | **High**  **income** | | **High deprivation** | | **Medium deprivation** | | **Low deprivation** | | | **No partner** | | **With partner** | |
|  |  | **n(/N)** | **%** | **n(/N)** | **%** | **n(/N)** | **%** | **n(/N)** | **%** | **n(/N)** | **%** | | **n(/N)** | **%** | **n(/N)** | **%** | **n(/N)** | **%** |
| Active Plus II | Tailored advice 1 completely read | NA | NA | NA | NA | NA | NA | NA | NA | NA | NA | | NA | NA | 144/154 | 94 | 712/766 | 93 |
|  | Tailored advice 2 completely read | NA | NA | NA | NA | NA | NA | NA | NA | NA | NA | | NA | NA | 118/152 | 78 | 584/762 | 77 |
|  | Tailored advice 3 completely read | NA | NA | NA | NA | NA | NA | NA | NA | NA | NA | | NA | NA | 108/121 | 89 | 499/578 | 86 |
|  | Role model stories read/watched | NA | NA | NA | NA | NA | NA | NA | NA | NA | NA | | NA | NA | 77/153 | 50 | 400/750 | 53 |
|  | Intrinsic motivation assignment used | NA | NA | NA | NA | NA | NA | NA | NA | NA | NA | | NA | NA | 46/152 | 30 | 230/754 | 31 |
|  | Formulate action plans used | NA | NA | NA | NA | NA | NA | NA | NA | NA | NA | | NA | NA | 11/150 | 7 | 54/753 | 7 |
|  | Formulate coping plans used | NA | NA | NA | NA | NA | NA | NA | NA | NA | NA | | NA | NA | 15/114 | 13 | 43/562 | 8 |
|  | Logbook used | NA | NA | NA | NA | NA | NA | NA | NA | NA | NA | | NA | NA | 7/116 | 6 | 44/507 | 8 |
|  | Additional information chronic limitation read | NA | NA | NA | NA | NA | NA | NA | NA | NA | NA | | NA | NA | 21/73 | 29 | 103/387 | 27 |
|  | Walk and cycling routes used | NA | NA | NA | NA | NA | NA | NA | NA | NA | NA | | NA | NA | 16/70 | 23 | 74/365 | 20 |
|  | Extra walk and cycling route used | NA | NA | NA | NA | NA | NA | NA | NA | NA | NA | | NA | NA | 9/56 | 16 | 62/275 | 23 |
|  | Sport information used | NA | NA | NA | NA | NA | NA | NA | NA | NA | NA | | NA | NA | 5/70 | 7 | 22/366 | 6 |
|  | Explanation how to plan a cycling route used | NA | NA | NA | NA | NA | NA | NA | NA | NA | NA | | NA | NA | 14/55 | 26 | 101/275 | 37 |
|  | PA home exercises used | NA | NA | NA | NA | NA | NA | NA | NA | NA | NA | | NA | NA | 25/65 | 39 | 125/346 | 36 |
|  | Additional exercises used | NA | NA | NA | NA | NA | NA | NA | NA | NA | NA | | NA | NA | 21/56 | 38 | 95/274 | 35 |
|  | Neighborhood map used | NA | NA | NA | NA | NA | NA | NA | NA | NA | NA | | NA | NA | 13/55 | 24 | 83/276 | 30 |
|  | Post cards / e-cards used | NA | NA | NA | NA | NA | NA | NA | NA | NA | NA | | NA | NA | 3/70 | 4 | 8/365 | 2 |
| GALM | Mean attendance rate of 15 intervention sessions | NA | NA | NA | NA | NA | NA | NA | NA | NA | NA | | NA | NA | 14 | 73 | 65 | 81 |
| PACE-UP | PA diary returned after 12-week intervention | NA | NA | NA | NA | NA | NA | 163/204 | 80 | 182/208 | 88 | | 177/206 | 86 | 180/209 | 86 | 351/417 | 84 |
|  | Pedometer used at every day or most days | NA | NA | NA | NA | NA | NA | 150/180 | 83 | 163/187 | 87 | | 174/194 | 90 | 164/191 | 86 | 333/381 | 87 |
| PROMOTE | Web-based PA diary used | 47/55 | 85 | 60/67 | 90 | 71/84 | 85 | NA | NA | NA | NA | | NA | NA | 42/50 | 84 | 143/167 | 86 |
|  | Group meetings attended | 42/56 | 75 | 54/68 | 79 | 60/85 | 71 | NA | NA | NA | NA | | NA | NA | 39/51 | 76 | 128/171 | 75 |
|  | Website used (FitBit) | 27/31 | 87 | 19/21 | 90 | 36/39 | 92 | NA | NA | NA | NA | | NA | NA | 16/19 | 84 | 73/79 | 92 |

NA = not applicable.
