# Supplementary material for: Equity-specific effects of interventions to promote physical activity among middle-aged and older adults: results from applying a novel equity-specific re-analysis strategy
Source: Int J Behav Nutr Phys Act. 2021 May 17;18:65. doi: 10.1186/s12966-021-01131-w (PMC8130354; doi:10.1186/s12966-021-01131-w)
Supplement: Supplementary file 5 — Additional file 5. Equity-specific dropout. This file contains the results of the secondary analysis on equity-specific dropout. [file 12966_2021_1131_MOESM5_ESM.docx]

**Additional file 5: Equity-specific dropout**

**Income-, area deprivation-, and marital status-specific dropout at T1**

| **Study** | **Intervention group** | | | | | | | | | | | |
| --- | --- | --- | --- | --- | --- | --- | --- | --- | --- | --- | --- | --- |
|  | **Income** | | | | | | **Area deprivation** | | | | | |
|  | **Low income** | | **Medium income** | | **High income** | | **High deprivation** | | **Medium deprivation** | | **Low deprivation** | |
|  | **Completers***  **n (%)** | **Dropouts****  **n (%)** | **Completers**  **n (%)** | **Dropouts**  **n (%)** | **Completers**  **n (%)** | **Dropouts**  **n (%)** | **Completers**  **n (%)** | **Dropouts**  **n (%)** | **Completers**  **n (%)** | **Dropouts**  **n (%)** | **Completers**  **n (%)** | **Dropouts**  **n (%)** |
| PACE-Lift | NA | NA | NA | NA | NA | NA | 47 (94) | 3 (6) | 49 (98) | 1 (2) | 46 (92) | 4 (8) |
| PACE-UP | NA | NA | NA | NA | NA | NA | 204 (91) | 20 (9) | 208 (93) | 15 (7) | 206 (96) | 8 (4) |
| ProAct65+ | 111 (56) | 89 (45) | 103 (65) | 55 (35) | 160 (66) | 84 (34) | 180 (61) | 115 (39) | 95 (58) | 70 (42) | 147 (60) | 97 (40) |
| PROMOTE | 57 (55) | 46 (45) | 68 (61) | 43 (39) | 88 (67) | 43 (33) | NA | NA | NA | NA | NA | NA |

| **Study** | **Control group** | | | | | | | | | | | |
| --- | --- | --- | --- | --- | --- | --- | --- | --- | --- | --- | --- | --- |
|  | **Income** | | | | | | **Area deprivation** | | | | | |
|  | **Low income** | | **Medium income** | | **High income** | | **High deprivation** | | **Medium deprivation** | | **Low deprivation** | |
|  | **Completers**  **n (%)** | **Dropouts**  **n (%)** | **Completers**  **n (%)** | **Dropouts**  **n (%)** | **Completers**  **n (%)** | **Dropouts**  **n (%)** | **Completers**  **n (%)** | **Dropouts**  **n (%)** | **Completers**  **n (%)** | **Dropouts**  **n (%)** | **Completers**  **n (%)** | **Dropouts**  **n (%)** |
| PACE-Lift | NA | NA | NA | NA | NA | NA | 55 (96) | 2 (4) | 41 (41) | 4 (9) | 42 (91) | 4 (9) |
| PACE-UP | NA | NA | NA | NA | NA | NA | 101 (94) | 7 (6) | 104 (96) | 4 (4) | 107 (96) | 4 (4) |
| ProAct65+ | 62 (67) | 30 (33) | 74 (65) | 40 (35) | 91 (65) | 50 (35) | 64 (61) | 41 (39) | 126 (65) | 67 (35) | 65 (64) | 37 (36) |
| PROMOTE | 40 (70) | 17 (30) | 36 (84) | 7 (16) | 43 (75) | 14 (25) | NA | NA | NA | NA | NA | NA |

| **Study** | **Intervention group** | | | | **Control group** | | | |
| --- | --- | --- | --- | --- | --- | --- | --- | --- |
|  | **No Partner** | | **With partner** | | **No Partner** | | **With partner** | |
|  | **Completers**  **n (%)** | **Dropouts**  **n (%)** | **Completers**  **n (%)** | **Dropouts**  **n (%)** | **Completers**  **n (%)** | **Dropouts**  **n (%)** | **Completers**  **n (%)** | **Dropouts**  **n (%)** |
| Active Plus I | 167 (61) | 105 (39) | 741 (68) | 348 (32) | 76 (77) | 23 (23) | 395 (85) | 72 (15) |
| Active Plus II | 154 (53) | 134 (47) | 700 (50) | 712 (50) | 63 (77) | 19 (23) | 241 (74) | 84 (26) |
| Every Step Counts! | 98 (59) | 68 (41) | 202 (67) | 99 (33) | 23 (64) | 13 (36) | 69 (58) | 49 (42) |
| GALM | 14 (48) | 15 (52) | 65 (49) | 69 (51) | 20 (74) | 7 (26) | 82 (66) | 43 (34) |
| PACE-Lift | 26 (96) | 1 (4) | 116 (94) | 7 (6) | 28 (93) | 2 (7) | 109 (93) | 8 (7) |
| PACE-UP | 209 (92) | 18 (8) | 417 (94) | 28 (6) | 112 (94) | 7 (6) | 202 (95) | 11 (5) |
| ProAct65+ | 179 (61) | 115 (39) | 241 (59) | 166 (41) | 109 (65) | 58 (35) | 146 (63) | 87 (37) |
| PROMOTE | 50 (53) | 44 (47) | 174 (63) | 101 (37) | 37 (74) | 13 (26) | 86 (76) | 27 (24) |

* individuals with information on MVPA at T0 (baseline) and T1 (post-intervention follow-up time-point closest to intervention end point). ** individuals with information on MVPA at T0 only. NA = not applicable.

**Equity-specific dropout at T2**

| **Study** | **Intervention group** | | | | | | | | | | | |
| --- | --- | --- | --- | --- | --- | --- | --- | --- | --- | --- | --- | --- |
|  | **Total sample** | | **Gender** | | | | **Education** | | | | | |
|  |  |  | **Males** | | **Females** | | **Low education** | | **Medium education** | | **High education** | |
|  | **Completers***  **n (%)** | **Dropouts****  **n (%)** | **Completers**  **n (%)** | **Dropouts**  **n (%)** | **Completers**  **n (%)** | **Dropouts**  **n (%)** | **Completers**  **n (%)** | **Dropouts**  **n (%)** | **Completers**  **n (%)** | **Dropouts**  **n (%)** | **Completers**  **n (%)** | **Dropouts**  **n (%)** |
| Active Plus I | 883 (64) | 501 (36) | 394 (66) | 207 (34) | 488 (63) | 292 (37) | 406 (64) | 228 (36) | 160 (60) | 107 (40) | 303 (66) | 159 (34) |
| Active Plus II | 940 (55) | 770 (45) | 457 (55) | 371 (45) | 480 (55) | 393 (45) | 424 (54) | 361 (46) | 239 (53) | 212 (47) | 273 (59) | 192 (41) |
| PACE-Lift | 137 (91) | 13 (9) | 61 (88) | 8 (12) | 76 (94) | 5 (6) | 57 (85) | 10 (15) | 24 (96) | 1 (4) | 53 (96) | 2 (4) |
| PACE-UP | 633 (92) | 52 (8) | 234 (93) | 18 (7) | 399 (92) | 34 (8) | 162 (92) | 15 (8) | 131 (92) | 11 (8) | 329 (94) | 22 (6) |
| ProAct65+ | 372 (53) | 332 (47) | 134 (51) | 127 (49) | 238 (54) | 205 (46) | 155 (47) | 175 (53) | 130 (60) | 88 (40) | 80 (56) | 63 (44) |

| **Study** | **Control group** | | | | | | | | | | | |
| --- | --- | --- | --- | --- | --- | --- | --- | --- | --- | --- | --- | --- |
|  | **Total sample** | | **Gender** | | | | **Education** | | | | | |
|  |  | | **Males** | | **Females** | | **Low education** | | **Medium education** | | **High education** | |
|  | **Completers**  **n (%)** | **Dropouts**  **n (%)** | **Completers**  **n (%)** | **Dropouts**  **n (%)** | **Completers**  **n (%)** | **Dropouts**  **n (%)** | **Completers**  **n (%)** | **Dropouts**  **n (%)** | **Completers**  **n (%)** | **Dropouts**  **n (%)** | **Completers**  **n (%)** | **Dropouts**  **n (%)** |
| Active Plus I | 463 (80) | 119 (20) | 195 (78) | 56 (22) | 267 (81) | 62 (19) | 226 (77) | 67 (23) | 85 (83) | 18 (17) | 140 (82) | 30 (18) |
| Active Plus II | 310 (76) | 99 (24) | 152 (75) | 52 (25) | 158 (77) | 47 (23) | 149 (75) | 50 (25) | 84 (79) | 23 (21) | 68 (76) | 22 (24) |
| PACE-Lift | 136 (92) | 12 (8) | 64 (93) | 5 (7) | 72 (91) | 7 (9) | 46 (85) | 8 (15) | 19 (95) | 1 (5) | 68 (96) | 3 (4) |
| PACE-UP | 323 (96) | 15 (4) | 112 (97) | 3 (3) | 211 (95) | 12 (5) | 80 (94) | 5 (6) | 81 (98) | 2 (2) | 158 (96) | 7 (4) |
| ProAct65+ | 231 (58) | 169 (42) | 86 (58) | 63 (42) | 145 (58) | 106 (42) | 88 (57) | 70 (44) | 82 (61) | 53 (39) | 59 (57) | 45 (43) |

| **Study** | **Intervention group** | | | | | | | | | | | |
| --- | --- | --- | --- | --- | --- | --- | --- | --- | --- | --- | --- | --- |
|  | **Income** | | | | | | **Area deprivation** | | | | | |
|  | **Low income** | | **Medium income** | | **High income** | | **High deprivation** | | **Medium deprivation** | | **Low deprivation** | |
|  | **Completers**  **n (%)** | **Dropouts**  **n (%)** | **Completers**  **n (%)** | **Dropouts**  **n (%)** | **Completers**  **n (%)** | **Dropouts**  **n (%)** | **Completers**  **n (%)** | **Dropouts**  **n (%)** | **Completers**  **n (%)** | **Dropouts**  **n (%)** | **Completers**  **n (%)** | **Dropouts**  **n (%)** |
| PACE-Lift | NA | NA | NA | NA | NA | NA | 45 (90) | 5 (10) | 46 (92) | 4 (8) | 46 (92) | 4 (8) |
| PACE-UP | NA | NA | NA | NA | NA | NA | 204 (91) | 20 (9) | 208 (93) | 15 (7) | 201 (94) | 13 (6) |
| ProAct65+ | 93 (47) | 107 (54) | 90 (57) | 68 (43) | 141 (58) | 103 (42) | 156 (53) | 139 (47) | 78 (47) | 87 (53) | 138 (57) | 106 (43) |

| **Study** | **Control group** | | | | | | | | | | | |  |
| --- | --- | --- | --- | --- | --- | --- | --- | --- | --- | --- | --- | --- | --- |
|  | **Income** | | | | | | **Area deprivation** | | | | | |  |
|  | **Low income** | | **Medium income** | | **High income** | | **High deprivation** | | **Medium deprivation** | | **Low deprivation** | | |
|  | **Completers**  **n (%)** | **Dropouts**  **n (%)** | **Completers**  **n (%)** | **Dropouts**  **n (%)** | **Completers**  **n (%)** | **Dropouts**  **n (%)** | **Completers**  **n (%)** | **Dropouts**  **n (%)** | **Completers**  **n (%)** | **Dropouts**  **n (%)** | **Completers**  **n (%)** | **Dropouts**  **n (%)** |  |
| PACE-Lift | NA | NA | NA | NA | NA | NA | 53 (93) | 4 (7) | 41 (91) | 4 (9) | 42 (91) | 4 (9) |  |
| PACE-UP | NA | NA | NA | NA | NA | NA | 99 (92) | 9 (8) | 105 (97) | 3 (3) | 109 (98) | 2 (2) |  |
| ProAct65+ | 52 (57) | 40 (43) | 69 (61) | 45 (39) | 85 (60) | 56 (40) | 60 (57) | 45 (43) | 114 (59) | 79 (41) | 57 (56) | 45 (44) |  |

| **Study** | **Intervention group** | | | | **Control group** | | | |
| --- | --- | --- | --- | --- | --- | --- | --- | --- |
|  | **No Partner** | | **With partner** | | **No Partner** | | **With partner** | |
|  | **Completers**  **n (%)** | **Dropouts**  **n (%)** | **Completers**  **n (%)** | **Dropouts**  **n (%)** | **Completers**  **n (%)** | **Dropouts**  **n (%)** | **Completers**  **n (%)** | **Dropouts**  **n (%)** |
| Active Plus I | 159 (58) | 113 (42) | 709 (65) | 380 (35) | 78 (79) | 21 (21) | 373 (80) | 94 (20) |
| Active Plus II | 161 (56) | 127 (44) | 773 (55) | 639 (45) | 57 (70) | 25 (30) | 251 (77) | 74 (23) |
| PACE-Lift | 26 (96) | 1 (4) | 111 (90) | 12 (10) | 27 (90) | 3 (10) | 108 (92) | 9 (8) |
| PACE-UP | 208 (92) | 19 (8) | 416 (93) | 29 (7) | 113 (95) | 6 (5) | 205 (96) | 8 (4) |
| ProAct65+ | 160 (54) | 134 (46) | 211 (52) | 196 (48) | 96 (57) | 71 (43) | 135 (58) | 98 (42) |

* individuals with information on MVPA at T0 (baseline) and T2 (12 months post baseline) (information on MVPA at T1 not necessary). ** individuals with information on MVPA at T0 only. NA = not applicable.
