# Supplementary material for: Equity-specific effects of interventions to promote physical activity among middle-aged and older adults: results from applying a novel equity-specific re-analysis strategy
Source: Int J Behav Nutr Phys Act. 2021 May 17;18:65. doi: 10.1186/s12966-021-01131-w (PMC8130354; doi:10.1186/s12966-021-01131-w)
Supplement: Supplementary file 6 — Additional file 6. Equity-specific baseline MVPA levels. This file contains information on equity-specific baseline MVPA levels (main and secondary analysis). [file 12966_2021_1131_MOESM6_ESM.docx]

**Additional file 6: Equity-specific** **baseline MVPA levels**

Equity-specific baseline MVPA levels among completers and dropouts at T1

| **Study** | **Intervention group** | | | | | | | | | | | | | | | | | | | | | | | |  |
| --- | --- | --- | --- | --- | --- | --- | --- | --- | --- | --- | --- | --- | --- | --- | --- | --- | --- | --- | --- | --- | --- | --- | --- | --- | --- |
|  | **Total sample** | | | | **Gender** | | | | | | | | **Education** | | | | | | | | | | | |  |
|  |  |  |  |  | **Males** | | | | **Females** | | | | **Low education** | | | | **Medium education** | | | | **High education** | | | | |
|  | **Completers*** | | **Dropouts**** | | **Completers** | | **Dropouts** | | **Completers** | | **Dropouts** | | **Completers** | | **Dropouts** | | **Completers** | | **Dropouts** | | **Completers** | | **Dropouts** | |  |
|  | **n** | **Mean (SD)** | **n** | **Mean (SD)** | **n** | **Mean (SD)** | **n** | **Mean (SD)** | **n** | **Mean (SD)** | **n** | **Mean (SD)** | **n** | **Mean (SD)** | **n** | **Mean (SD)** | **n** | **Mean (SD)** | **n** | **Mean (SD)** | **n** | **Mean (SD)** | **n** | **Mean (SD)** |  |
| Active Plus I | 925 | 761 (665) | 459 | 643 (715) | 410 | 841 (740) | 191 | 697 (805) | 514 | 698 (592) | 266 | 605 (643) | 425 | 824 (700) | 209 | 704 (736) | 170 | 837 (734) | 97 | 638 (765) | 313 | 647 (554) | 149 | 574 (649) |  |
| Active Plus II | 860 | 727 (661) | 850 | 709 (752) | 414 | 739 (656) | 414 | 765 (812) | 444 | 714 (664) | 429 | 652 (673) | 386 | 735 (665) | 399 | 666 (704) | 229 | 832 (742) | 222 | 827 (918) | 240 | 621 (553) | 225 | 673 (633) |  |
| Every Step Counts! | 300 | 61 (56) | 168 | 58 (64) | 94 | 79  (68) | 47 | 74 (82) | 206 | 73 (66) | 121 | 53 (54) | 167 | 66 (60) | 89 | 54 (63) | 102 | 55 (54) | 50 | 56 (62) | 28 | 53 (43) | 28 | 79 (66) |  |
| GALM | 79 | 455 (403) | 84 | 560 (476) | 36 | 601 (494) | 36 | 633 (539) | 43 | 333 (256) | 48 | 505 (420) | 34 | 429 (380) | 30 | 573 (516) | 23 | 393 (290) | 30 | 606 (507) | 22 | 562 (521) | 24 | 484 (386) |  |
| PACE-Lift | 142 | 297 (153) | 8 | 269 (191) | 64 | 345 (162) | 5 | 267 (236) | 78 | 258 (133) | 3 | 273 (128) | 61 | 271 (140) | 6 | 187 (125) | 25 | 329 (174) | 2 | 515 (130) | 53 | 314 (155) | 8 | 269 (191) |  |
| PACE-UP | 636 | 318 (152) | 49 | 277 (134) | 236 | 347 (158) | 16 | 264 (131) | 400 | 301 (147) | 33 | 284 (137) | 165 | 284 (147) | 12 | 300 (136) | 132 | 313 (150) | 10 | 244 (125) | 327 | 336 (153) | 24 | 287 (130) |  |
| ProAct65+ | 422 | 188 (228) | 282 | 174 (257) | 154 | 249 (277) | 107 | 201 (246) | 268 | 153 (187) | 175 | 158 (263) | 177 | 182 (245) | 153 | 171 (268) | 148 | 216 (225) | 70 | 183 (279) | 89 | 150 (171) | 54 | 175 (191) |  |
| PROMOTE | 226 | 47 (23) | 148 | 41 (22) | 100 | 48 (25) | 65 | 45 (25) | 126 | 46 (21) | 83 | 39 (19) | 2 | 34 (38) | 4 | 39 (13) | 102 | 46 (23) | 83 | 40 (22) | 122 | 48 (23) | 61 | 44 (23) |  |

| **Study** | **Control group** | | | | | | | | | | | | | | | | | | | | | | | | | | | | | | | | | | | | | | | | | | | | | |  |  |
| --- | --- | --- | --- | --- | --- | --- | --- | --- | --- | --- | --- | --- | --- | --- | --- | --- | --- | --- | --- | --- | --- | --- | --- | --- | --- | --- | --- | --- | --- | --- | --- | --- | --- | --- | --- | --- | --- | --- | --- | --- | --- | --- | --- | --- | --- | --- | --- | --- |
|  | **Total sample** | | | | | | | **Gender** | | | | | | | | | | | | | | | | **Education** | | | | | | | | | | | | | | | | | | | | | | |  |  |
|  |  |  |  |  |  |  |  | **Males** | | | | | | | | **Females** | | | | | | | | **Low education** | | | | | | | | **Medium education** | | | | | | | | **High education** | | | | | | |  |  |
|  | **Completers** | | | **Dropouts** | | | | **Completers** | | | | **Dropouts** | | | | **Completers** | | | | **Dropouts** | | | | **Completers** | | | | **Dropouts** | | | | **Completers** | | | | **Dropouts** | | | | **Completers** | | | | **Dropouts** | | |  |  |
|  | **n** | **Mean (SD)** | | **n** | | **Mean (SD)** | | **n** | | **Mean (SD)** | | **n** | | **Mean (SD)** | | **n** | | **Mean (SD)** | | **n** | | **Mean (SD)** | | **n** | | **Mean (SD)** | | **n** | | **Mean (SD)** | | **n** | **Mean (SD)** | | | **n** | **Mean (SD)** | | | **n** | **Mean (SD)** | | | **n** | **Mean (SD)** | |  |  |
| Active Plus I | 484 | 663 (623) | | 98 | | 560 (620) | | 208 | | 714 (683) | | 43 | | 678 (752) | | 275 | | 616 (555) | | 54 | | 476 (481) | | 244 | | 742 (705) | | 49 | | 511 (630) | | 87 | 650 (577) | | | 16 | 816 (755) | | | 139 | 556 (484) | | | 31 | 521 (520) | |  |  |
| Active Plus II | 305 | 774 (695) | | 104 | | 902 (1007) | | 144 | | 826 (739) | | 60 | | 943 (1062) | | 161 | | 728 (652) | | 44 | | 846 (938) | | 148 | | 759 (706) | | 51 | | 899 (1087) | | 82 | 784 (651) | | | 25 | 864 (863) | | | 68 | 731 (684) | | | 22 | 737 (732) | |  |  |
| Every Step Counts! | 95 | 55 (51) | | 59 | | 54 (48) | | 35 | | 58 (61) | | 23 | | 54 (32) | | 57 | | 53 (44) | | 39 | | 54 (56) | | 54 | | 53 (52) | | 36 | | 54 (53) | | 24 | 61 (54) | | | 17 | 54 (40) | | | 13 | 49 (42) | | | 9 | 53 (47) | |  |  |
| GALM | 102 | 402 (332) | | 50 | | 388 (291) | | 44 | | 420 (370) | | 29 | | 411 (311) | | 58 | | 389 (302) | | 21 | | 356 (265) | | 34 | | 355 (319) | | 13 | | 481 (271) | | 46 | 440 (369) | | | 24 | 325 (268) | | | 22 | 395 (268) | | | 13 | 413 (343) | |  |  |
| PACE-Lift | 138 | 310 (169) | | 10 | | 178 (122) | | 65 | | 302 (171) | | 4 | | 220 (155) | | 73 | | 316 (169) | | 6 | | 150 (100) | | 49 | | 275 (162) | | 5 | | 115 (61) | | 19 | 341 (136) | | | 1 | 278 (0) | | | 68 | 325 (182) | | | 3 | 230 (197) | |  |  |
| PACE-UP | 318 | 305 (148) | | 20 | | 300 (196) | | 109 | | 350 (167) | | 6 | | 248 (154) | | 209 | | 282 (132) | | 14 | | 323 (212) | | 82 | | 287 (157) | | 3 | | 171 (87) | | 78 | 296 (146) | | | 5 | 257 (186) | | | 155 | 322 (143) | | | 10 | 387 (208) | |  |  |
| ProAct65+ | 255 | 193 (227) | | 145 | | 154 (259) | | 95 | | 264 (277) | | 54 | | 245 (349) | | 160 | | 151 (180) | | 91 | | 101 (166) | | 98 | | 204 (243) | | 60 | | 135 (218) | | 87 | 188 (227) | | | 48 | 162 (313) | | | 68 | 186 (209) | | | 36 | 181 (249) | |  |  |
| PROMOTE | 124 | 46 (23) | | 40 | | 38 (24) | | 60 | | 49 (27) | | 10 | | 42 (33) | | 64 | | 44 (20) | | 30 | | 37 (21) | | 4 | | 39 (22) | | 2 | | 44 (23) | | 66 | 44 (20) | | | 27 | 33 (22) | | | 54 | 50 (27) | | | 11 | 50 (26) | |  |  |
| **Study** | **Intervention group** | | | | | | | | | | | | | | | | | | | | | | | | | | | | | | | | | | | | | | | | | | | | | | | |
|  | **Income** | | | | | | | | | | | | | | | | | | | | | | | | **Area deprivation** | | | | | | | | | | | | | | | | | | | | | | | |
|  | **Low income** | | | | | | | | **Medium income** | | | | | | | | **High income** | | | | | | | | **High deprivation** | | | | | | | | | **Medium deprivation** | | | | | | | | **Low deprivation** | | | | | | |
|  | **Completers*** | | | | **Dropouts**** | | | | **Completers** | | | | **Dropouts** | | | | **Completers** | | | | **Dropouts** | | | | **Completers** | | | | **Dropouts** | | | | | **Completers** | | | | **Dropouts** | | | | **Completers** | | | | **Dropouts** | | |
|  | **n** | | **Mean (SD)** | | **n** | | **Mean (SD)** | | **n** | | **Mean (SD)** | | **n** | | **Mean (SD)** | | **n** | | **Mean (SD)** | | **n** | | **Mean (SD)** | | **n** | | **Mean (SD)** | | **n** | | **Mean (SD)** | | | **n** | **Mean (SD)** | | | **n** | **Mean (SD)** | | | **n** | **Mean (SD)** | | | **n** | | **Mean (SD)** |
| PACE-Lift | NA | | NA | | NA | | NA | | NA | | NA | | NA | | NA | | NA | | NA | | NA | | NA | | 47 | | 301 (173) | | 3 | | 110 (33) | | | 49 | 284 (139) | | | 1 | 423 (0) | | | 46 | 307 (147) | | | 4 | | 350 (207) |
| PACE-UP | NA | | NA | | NA | | NA | | NA | | NA | | NA | | NA | | NA | | NA | | NA | | NA | | 204 | | 298 (135) | | 20 | | 304 (131) | | | 208 | 313 (168) | | | 15 | 207 (109) | | | 206 | 341 (152) | | | 8 | | 312 (164) |
| ProAct65+ | 111 | | 163 (188) | | 89 | | 126 (223) | | 103 | | 171 (201) | | 55 | | 187 (273) | | 160 | | 230 (265) | | 84 | | 235 (295) | | 180 | | 179 (205) | | 115 | | 186 (311) | | | 95 | 184 (250) | | | 70 | 152 (182) | | | 147 | 202 (240) | | | 97 | | 177 (233) |
| PROMOTE | 57 | | 46 (24) | | 46 | | 39 (22) | | 68 | | 48 (20) | | 43 | | 44 (22) | | 88 | | 49 (25) | | 42 | | 44 (26) | | NA | | NA | | NA | | NA | | | NA | NA | | | NA | NA | | | NA | NA | | | NA | | NA |

| **Study** | **Control group** | | | | | | | | | | | | | | | | | | | | | | | |
| --- | --- | --- | --- | --- | --- | --- | --- | --- | --- | --- | --- | --- | --- | --- | --- | --- | --- | --- | --- | --- | --- | --- | --- | --- |
|  | **Income** | | | | | | | | | | | | **Area deprivation** | | | | | | | | | | | |
|  | **Low income** | | | | **Medium income** | | | | **High income** | | | | **High deprivation** | | | | **Medium deprivation** | | | | **Low deprivation** | | | |
|  | **Completers** | | **Dropouts** | | **Completers** | | **Dropouts** | | **Completers** | | **Dropouts** | | **Completers** | | **Dropouts** | | **Completers** | | **Dropouts** | | **Completers** | | **Dropouts** | |
|  | **n** | **Mean (SD)** | **n** | **Mean (SD)** | **n** | **Mean (SD)** | **n** | **Mean (SD)** | **n** | **Mean (SD)** | **n** | **Mean (SD)** | **n** | **Mean (SD)** | **n** | **Mean (SD)** | **n** | **Mean (SD)** | **n** | **Mean (SD)** | **n** | **Mean (SD)** | **n** | **Mean (SD)** |
| PACE-Lift | NA | NA | NA | NA | NA | NA | NA | NA | NA | NA | NA | NA | 55 | 299 (163) | 2 | 256 (31) | 41 | 297 (172) | 4 | 180 (174) | 42 | 336 (175) | 4 | 137 (87) |
| PACE-UP | NA | NA | NA | NA | NA | NA | NA | NA | NA | NA | NA | NA | 101 | 290 (149) | 7 | 245 (163) | 104 | 297 (149) | 4 | 343 (290) | 107 | 324 (144) | 4 | 385 (233) |
| ProAct65+ | 62 | 199 (244) | 30 | 92 (178) | 74 | 177 (208) | 40 | 164 (246) | 91 | 207 (219) | 50 | 212 (333) | 64 | 179 (196) | 41 | 182 (258) | 126 | 194 (199) | 67 | 169 (301) | 65 | 205 (299) | 37 | 96 (149) |
| PROMOTE | 40 | 49 (25) | 17 | 39 (22) | 36 | 42 (17) | 7 | 34 (20) | 43 | 49 (26) | 14 | 41 (30) | NA | NA | NA | NA | NA | NA | NA | NA | NA | NA | NA | NA |

| **Study** | **Intervention group** | | | | | | | | | **Control group** | | | | | | | | | | | |  |
| --- | --- | --- | --- | --- | --- | --- | --- | --- | --- | --- | --- | --- | --- | --- | --- | --- | --- | --- | --- | --- | --- | --- |
|  | **No Partner** | | | | | **With partner** | | | | | **No Partner** | | | | | **With partner** | | | | | |  |
|  | **Completers** | | **Dropouts** | | | **Completers** | | **Dropouts** | | | **Completers** | | | **Dropouts** | | **Completers** | | | **Dropouts** | | |  |
|  | **n** | **Mean (SD)** | | **n** | **Mean (SD)** | **n** | **Mean (SD)** | **n** | **Mean (SD)** | | **n** | **Mean (SD)** | **n** | | **Mean (SD)** | | **n** | **Mean (SD)** | | **n** | **Mean (SD)** | |
| Active Plus I | 167 | 724 (628) | | 105 | 706 (889) | 741 | 773 (667) | 348 | 631 (657) | | 76 | 734 (718) | 23 | | 636 (622) | | 395 | 656 (610) | | 72 | 548 (629) | |
| Active Plus II | 154 | 739 (654) | | 134 | 596 (716) | 700 | 725 (661) | 712 | 726 (755) | | 63 | 757 (595) | 19 | | 963 (1224) | | 241 | 776 (720) | | 84 | 896 (963) | |
| Every Step Counts! | 98 | 53 (44) | | 68 | 43 (44) | 202 | 65 (61) | 99 | 70 (73) | | 23 | 65 (48) | 13 | | 32 (28) | | 69 | 51 (52) | | 49 | 60 (51) | |
| GALM | 14 | 465 (307) | | 15 | 724 (443) | 65 | 453 (423) | 69 | 524 (478) | | 20 | 450 (402) | 7 | | 531 (280) | | 82 | 390 (314) | | 43 | 365 (290) | |
| PACE-Lift | 26 | 282 (141) | | 1 | 127 (0) | 116 | 301 (155) | 7 | 290 (197) | | 28 | 316 (199) | 2 | | 152 (179) | | 109 | 307 (163) | | 8 | 184 (120) | |
| PACE-UP | 209 | 315 (152) | | 18 | 242 (127) | 417 | 317 (152) | 28 | 306 (127) | | 112 | 291 (149) | 7 | | 319 (201) | | 202 | 314 (147) | | 11 | 312 (208) | |
| ProAct65+ | 179 | 138 (162) | | 115 | 129 (215) | 241 | 222 (254) | 166 | 206 (279) | | 109 | 170 (210) | 58 | | 124 (198) | | 146 | 211 (238) | | 87 | 175 (291) | |
| PROMOTE | 50 | 49 (23) | | 42 | 43 (18) | 174 | 46 (23) | 101 | 42 (24) | | 37 | 46 (21) | 13 | | 30 (17) | | 86 | 47 (24) | | 27 | 42 (26) | |

* individuals with information on MVPA at T0 (baseline) and T1 (post-intervention follow-up time-point closest to intervention end point). ** individuals with information on MVPA at T0 only. NA = not applicable. SD = Standard deviation.

Equity-specific baseline MVPA levels among completers and dropouts at T2

| **Study** | **Intervention group** | | | | | | | | | | | | | | | | | | | | | | | | |
| --- | --- | --- | --- | --- | --- | --- | --- | --- | --- | --- | --- | --- | --- | --- | --- | --- | --- | --- | --- | --- | --- | --- | --- | --- | --- |
|  | **Total sample** | | | | **Gender** | | | | | | | | **Education** | | | | | | | | | | | | |
|  |  |  |  |  | **Males** | | | | **Females** | | | | **Low education** | | | | | **Medium education** | | | | **High education** | | | |
|  | **Completers*** | | **Dropouts**** | | **Completers** | | **Dropouts** | | **Completers** | | **Dropouts** | | **Completers** | | **Dropouts** | | | **Completers** | | **Dropouts** | | **Completers** | | **Dropouts** | |
|  | **n** | **Mean (SD)** | **n** | **Mean (SD)** | **n** | **Mean (SD)** | **n** | **Mean (SD)** | **n** | **Mean (SD)** | **n** | **Mean (SD)** | **n** | **Mean (SD)** | | **n** | **Mean (SD)** | **n** | **Mean (SD)** | **n** | **Mean (SD)** | **n** | **Mean (SD)** | **n** | **Mean (SD)** |
| Active Plus I | 883 | 775 (674) | 501 | 628 (691) | 394 | 852 (750) | 207 | 687 (780) | 488 | 712 (601) | 292 | 588 (621) | 406 | 839 (719) | | 228 | 688 (697) | 160 | 864 (739) | 107 | 616 (746) | 303 | 650 (550) | 159 | 574 (649) |
| Active Plus II | 940 | 739 (678) | 770 | 692 (741) | 457 | 774 (722) | 371 | 724 (757) | 480 | 708 (634) | 393 | 654 (709) | 424 | 731 (653) | | 361 | 663 (720) | 239 | 856 (807) | 212 | 800 (861) | 273 | 653 (574) | 192 | 637 (619) |
| PACE-Lift | 137 | 297 (152) | 13 | 287 (178) | 61 | 341 (164) | 8 | 329 (204) | 76 | 261 (133) | 5 | 221 (117) | 57 | 264 (139) | | 10 | 259 (157) | 24 | 338 (172) | 1 | 116 (0) | 53 | 314 (155) | 2 | 515 (130) |
| PACE-UP | 633 | 318 (151) | 52 | 270 (156) | 234 | 346 (156) | 18 | 288 (162) | 399 | 303 (145) | 34 | 260 (154) | 162 | 288 (147) | | 15 | 261 (131) | 131 | 316 (148) | 11 | 212 (134) | 329 | 335 (151) | 22 | 293 (155) |
| ProAct65+ | 372 | 192 (234) | 332 | 172 (246) | 134 | 259 (286) | 127 | 198 (238) | 238 | 155 (190) | 205 | 155 (251) | 155 | 199 (281) | | 175 | 156 (229) | 130 | 200 (189) | 88 | 214 (309) | 80 | 159 (176) | 63 | 159 (182) |

| **Study** | **Control group** | | | | | | | | | | | | | | | | | | | | | | | |
| --- | --- | --- | --- | --- | --- | --- | --- | --- | --- | --- | --- | --- | --- | --- | --- | --- | --- | --- | --- | --- | --- | --- | --- | --- |
|  | **Total sample** | | | | **Gender** | | | | | | | | **Education** | | | | | | | | | | | |
|  |  |  |  |  | **Males** | | | | **Females** | | | | **Low education** | | | | **Medium education** | | | | **High education** | | | |
|  | **Completers** | | **Dropouts** | | **Completers** | | **Dropouts** | | **Completers** | | **Dropouts** | | **Completers** | | **Dropouts** | | **Completers** | | **Dropouts** | | **Completers** | | **Dropouts** | |
|  | **n** | **Mean (SD)** | **n** | **Mean (SD)** | **n** | **Mean (SD)** | **n** | **Mean (SD)** | **n** | **Mean (SD)** | **n** | **Mean (SD)** | **n** | **Mean (SD)** | **n** | **Mean (SD)** | **n** | **Mean (SD)** | **n** | **Mean (SD)** | **n** | **Mean (SD)** | **n** | **Mean (SD)** |
| Active Plus I | 463 | 673 (640) | 119 | 542 (542) | 195 | 733 (713) | 56 | 620 (619) | 267 | 620 (562) | 62 | 479 (456) | 226 | 755 (723) | 67 | 529 (574) | 85 | 663 (612) | 18 | 736 (597) | 140 | 561 (502) | 30 | 498 (429) |
| Active Plus II | 310 | 788 (726) | 99 | 864 (955) | 152 | 858 (798) | 52 | 868 (981) | 158 | 722 (645) | 47 | 859 (936) | 149 | 771 (717) | 50 | 864 (1074) | 84 | 829 (688) | 23 | 705 (762) | 68 | 695 (667) | 22 | 847 (767) |
| PACE-Lift | 136 | 310 (169) | 12 | 191 (144) | 64 | 307 (170) | 5 | 170 (119) | 72 | 313 (168) | 7 | 206 (167) | 46 | 274 (161) | 8 | 179 (155) | 19 | 341 (136) | 1 | 278 (0) | 68 | 327 (182) | 3 | 195 (160) |
| PACE-UP | 323 | 303 (147) | 15 | 333 (224) | 112 | 342 (166) | 3 | 410 (225) | 211 | 282 (132) | 12 | 314 (230) | 80 | 281 (153) | 5 | 313 (216) | 81 | 295 (145) | 2 | 244 (313) | 158 | 323 (143) | 7 | 384 (251) |
| ProAct65+ | 231 | 209 (243) | 169 | 137 (228) | 86 | 298 (300) | 63 | 201 (302) | 145 | 157 (185) | 106 | 100 (159) | 88 | 220 (266) | 70 | 125 (178) | 82 | 197 (230) | 53 | 151 (300) | 59 | 215 (232) | 45 | 143 (203) |

| **Study** | **Intervention group** | | | | | | | | | | | | | | | | | | | | | | | |
| --- | --- | --- | --- | --- | --- | --- | --- | --- | --- | --- | --- | --- | --- | --- | --- | --- | --- | --- | --- | --- | --- | --- | --- | --- |
|  | **Income** | | | | | | | | | | | | **Area deprivation** | | | | | | | | | | | |
|  | **Low income** | | | | **Medium income** | | | | **High income** | | | | **High deprivation** | | | | **Medium deprivation** | | | | **Low deprivation** | | | |
|  | **Completers** | | **Dropouts** | | **Completers** | | **Dropouts** | | **Completers** | | **Dropouts** | | **Completers** | | **Dropouts** | | **Completers** | | **Dropouts** | | **Completers** | | **Dropouts** | |
|  | **n** | **Mean (SD)** | **n** | **Mean (SD)** | **n** | **Mean (SD)** | **n** | **Mean (SD)** | **n** | **Mean (SD)** | **n** | **Mean (SD)** | **n** | **Mean (SD)** | **n** | **Mean (SD)** | **n** | **Mean (SD)** | **n** | **Mean (SD)** | **n** | **Mean (SD)** | **n** | **Mean**  **(SD)** |
| PACE-Lift | NA | NA | NA | NA | NA | NA | NA | NA | NA | NA | NA | NA | 45 | 298 (176) | 5 | 217 (149) | 46 | 285 (135) | 4 | 312 (201) | 46 | 307 (147) | 4 | 350  (207) |
| PACE-UP | NA | NA | NA | NA | NA | NA | NA | NA | NA | NA | NA | NA | 204 | 298 (133) | 20 | 304 (153) | 208 | 310 (167) | 15 | 243 (146) | 201 | 344 (150) | 13 | 274  (179) |
| ProAct65+ | 93 | 161 (191) | 107 | 134 (216) | 90 | 195 (258) | 68 | 152 (180) | 141 | 223 (247) | 103 | 245 (311) | 156 | 197 (244) | 139 | 164 (258) | 78 | 158 (193) | 87 | 182 (249) | 138 | 206 (243) | 106 | 174  (229) |

| **Study** | **Control group** | | | | | | | | | | | | | | | | | | | | | | | |
| --- | --- | --- | --- | --- | --- | --- | --- | --- | --- | --- | --- | --- | --- | --- | --- | --- | --- | --- | --- | --- | --- | --- | --- | --- |
|  | **Income** | | | | | | | | | | | | **Area deprivation** | | | | | | | | | | | |
|  | **Low income** | | | | **Medium income** | | | | **High income** | | | | **High deprivation** | | | | **Medium deprivation** | | | | **Low deprivation** | | | |
|  | **Completers** | | **Dropouts** | | **Completers** | | **Dropouts** | | **Completers** | | **Dropouts** | | **Completers** | | **Dropouts** | | **Completers** | | **Dropouts** | | **Completers** | | **Dropouts** | |
|  | **n** | **Mean (SD)** | **n** | **Mean (SD)** | **n** | **Mean (SD)** | **n** | **Mean (SD)** | **n** | **Mean (SD)** | **n** | **Mean (SD)** | **n** | **Mean (SD)** | **n** | **Mean (SD)** | **n** | **Mean (SD)** | **n** | **Mean (SD)** | **n** | **Mean (SD)** | **n** | **Mean (SD)** |
| PACE-Lift | NA | NA | NA | NA | NA | NA | NA | NA | NA | NA | NA | NA | 53 | 294 (163) | 4 | 342 (136) | 41 | 301 (173) | 4 | 138 (90) | 42 | 340 (171) | 4 | 93 (48) |
| PACE-UP | NA | NA | NA | NA | NA | NA | NA | NA | NA | NA | NA | NA | 99 | 283 (148) | 9 | 326 (170) | 105 | 298 (146) | 3 | 322 (392) | 109 | 324 (143) | 2 | 399 (395) |
| ProAct65+ | 52 | 208 (259) | 40 | 106 (170) | 69 | 208 (227) | 45 | 117 (203) | 85 | 220 (239) | 56 | 192 (300) | 60 | 206 (239) | 45 | 146 (191) | 114 | 203 (206) | 79 | 160 (279) | 57 | 226 (312) | 45 | 90 (142) |

| **Study** | **Intervention group** | | | | | | | | **Control group** | | | | | | | |
| --- | --- | --- | --- | --- | --- | --- | --- | --- | --- | --- | --- | --- | --- | --- | --- | --- |
|  | **No Partner** | | | | **With partner** | | | | **No Partner** | | | | **With partner** | | | |
|  | **Completers** | | **Dropouts** | | **Completers** | | **Dropouts** | | **Completers** | | **Dropouts** | | **Completers** | | **Dropouts** | |
|  | **n** | **Mean**  **(SD)** | **n** | **Mean**  **(SD)** | **n** | **Mean (SD)** | **n** | **Mean**  **(SD)** | **n** | **Mean**  **(SD)** | **n** | **Mean**  **(SD)** | **n** | **Mean**  **(SD)** | **n** | **Mean**  **(SD)** |
| Active Plus I | 159 | 762 (636) | 113 | 654 (861) | 709 | 780 (677) | 380 | 631 (637) | 78 | 761 (737) | 21 | 529 (478) | 373 | 660 (625) | 94 | 560 (561) |
| Active Plus II | 161 | 719 (634) | 127 | 613 (746) | 773 | 742 (685) | 639 | 706 (738) | 57 | 757 (602) | 25 | 913 (1096) | 251 | 796 (753) | 74 | 847 (910) |
| PACE-Lift | 26 | 282 (141) | 1 | 127 (0) | 111 | 300 (155) | 12 | 301 (179) | 27 | 323 (198) | 3 | 138 (129) | 108 | 307 (162) | 9 | 209 (152) |
| PACE-UP | 208 | 318 (148) | 19 | 215 (153) | 416 | 318 (152) | 29 | 297 (129) | 113 | 291 (148) | 6 | 316 (224) | 205 | 313 (146) | 8 | 356 (251) |
| ProAct65+ | 160 | 137 (162) | 134 | 131 (208) | 211 | 229 (262) | 196 | 201 (267) | 96 | 185 (218) | 71 | 111 (183) | 135 | 227 (260) | 98 | 156 (255) |

* individuals with information on MVPA at T0 (baseline) and T2 (12 months post baseline) (information on MVPA at T1 not necessary). ** individuals with information on MVPA at T0 only. NA = not applicable. SD = Standard deviation.
