# Supplementary material for: Equity-specific effects of interventions to promote physical activity among middle-aged and older adults: results from applying a novel equity-specific re-analysis strategy
Source: Int J Behav Nutr Phys Act. 2021 May 17;18:65. doi: 10.1186/s12966-021-01131-w (PMC8130354; doi:10.1186/s12966-021-01131-w)
Supplement: Supplementary file 7 — Additional file 7. General and equity-specific intervention effects. This file contains the results of the general, gender-, and education-specific intervention effect analysis at T1 derived from the minimally adjusted models as well as the results of the secondary and sensitivity analyses on general and equity-specific intervention effects. [file 12966_2021_1131_MOESM7_ESM.docx]

**Additional file 7: General and equity-specific intervention effects**

**General and equity-specific intervention effects at T1** **(minimally adjusted models)**

| **Study** | **General intervention effect*** | | **Gender-specific intervention effects**** | | | | |
| --- | --- | --- | --- | --- | --- | --- | --- |
|  |  |  | **Males** | | **Females** | | **P-value**  **intervention*gender interaction** |
|  | **n** | **Estimate (95% CI)** | **n** | **Estimate (95% CI)** | **n** | **Estimate (95% CI)** |  |
| Active Plus I | 1409 | -2.2 (-60.1; 55.6) | 613 | -124.3 (-212.0; -36.6) | 783 | 86.2 (9.1; 163.4) | <0.001 |
| Active Plus II | 1165 | 179.0 (96.7; 261.4) | 557 | 152.2 (32.4; 272.0) | 603 | 218.6 (104.7; 332.6) | 0.430 |
| Every Step Counts! | 393 | 18.0 (6.7; 29.2) | 129 | 21.5 (2.7; 40.2) | 264 | 16.4 (2.4; 30.5) | 0.673 |
| GALM | 181 | 40.8 (-30.4; 112.0) | 80 | 77.9 (-29.3; 185.1) | 101 | 0.1 (-96.2; 96.4) | 0.290 |
| PACE-Lift^a^ | 280 | 61.4 (30.4; 92.3) | 129 | 81.7 (38.6; 124.8) | 151 | 56.5 (16.6; 96.4) | 0.377 |
| PACE-UP^a^ | 954 | 48.7 (31.2; 66.2) | 345 | 43.8 (15.5; 72.1) | 609 | 50.0 (29.0; 71.1) | 0.721 |
| ProAct65+^b^ | 677 | -2.4 (-46.8; 42.1) | 249 | -44.6 (-109.1; 19.8) | 428 | 11.5 (-40.2; 63.2) | 0.137 |
| PROMOTE^c^ | 350 | 7.7 (2.8; 12.7) | 160 | 10.1 (2.7; 17.4) | 190 | 5.2 (-1.6; 12.0) | 0.342 |

| **Study** | **Education-specific intervention effects**** | | | | | | |
| --- | --- | --- | --- | --- | --- | --- | --- |
|  | **Low education** | | **Medium education** | | **High education** | | **P-value**  **intervention*education interaction** |
|  | **n** | **Estimate (95% CI)** | **n** | **Estimate (95% CI)** | **n** | **Estimate (95% CI)** |  |
| Active Plus I | 666 | 14.2 (-69.3; 97.7) | 255 | 13.3 (-123.4; 150.0) | 451 | -1.1 (-106.5; 104.3) | 0.973 |
| Active Plus II | 533 | 234.5 (115.1; 353.9) | 311 | 201.3 (42.9; 359.7) | 308 | 110.2 (-59.0; 279.3) | 0.497 |
| Every Step Counts! | 222 | 16.1 (1.3; 31.0) | 126 | 12.0 (-9.4; 33.5) | 41 | 32.0 (0.3; 63.7) | 0.583 |
| GALM | 68 | 81.8 (-34.3; 197.9) | 69 | 7.4 (-113.8; 128.7) | 44 | -22.4 (-167.0; 122.1) | 0.486 |
| PACE-Lift^a^ | 110 | 103.8 (56.0; 151.6) | 44 | 32.8 (-41.0; 106.5) | 121 | 60.2 (15.2; 105.3) | 0.203 |
| PACE-UP^a^ | 247 | 14.8 (-19.4; 49.0) | 210 | 87.2 (52.1; 122.3) | 482 | 46.8 (22.4; 71.1) | 0.014 |
| ProAct65+^b^ | 275 | -33.7 (-95.6; 28.2) | 235 | 21.1 (-44.6; 86.7) | 157 | 8.5 (-68.5; 85.6) | 0.407 |
| PROMOTE^c^ | 6 | 16.8 (-21.3; 54.9) | 168 | 4.9 (-2.1; 11.9) | 176 | 9.9 (2.7; 17.1) | 0.549 |

| **Study** | **Income-specific intervention effects**** | | | | | | |
| --- | --- | --- | --- | --- | --- | --- | --- |
|  | **Low income** | | **Medium income** | | **High income** | | **P-value**  **intervention*income interaction** |
|  | **n** | **Estimate (95% CI)** | **n** | **Estimate (95% CI)** | **n** | **Estimate (95% CI)** |  |
| ProAct65+^b^ | 173 | -1.7 (-78.0; 74.5) | 177 | -38.2 (-112.3; 36.0) | 251 | 21.7 (-44.6; 88.0) | 0.399 |
| PROMOTE^c^ | 97 | 6.9 (-2.4; 16.1) | 104 | 3.6 (-5.6; 12.8) | 131 | 10.3 (1.9; 18.7) | 0.570 |

| **Study** | **Area deprivation-specific intervention effects**** | | | | | | |
| --- | --- | --- | --- | --- | --- | --- | --- |
|  | **High deprivation** | | **Medium deprivation** | | **Low deprivation** | | **P-value**  **intervention*area deprivation interaction** |
|  | **n** | **Estimate (95% CI)** | **n** | **Estimate (95% CI)** | **n** | **Estimate (95% CI)** |  |
| PACE-Lift^a^ | 102 | 48.6 (-3.0; 100.2) | 90 | 59.8 (6.0; 113.6) | 88 | 95.7 (41.6; 149.9) | 0.450 |
| PACE-UP^a^ | 305 | 27.9 (-2.9; 58.6) | 312 | 47.2 (16.7; 77.8) | 313 | 66.6 (36.3; 96.8) | 0.214 |
| ProAct65+^b^ | 244 | -22.6 (-99.8; 54.5) | 221 | -4.4 (-78.2; 69.3) | 212 | 24.9 (-57.6; 107.5) | 0.710 |

| **Study** | **Marital status-specific intervention effects**** | | | | |
| --- | --- | --- | --- | --- | --- |
|  | **No partner** | | **With partner** | | **P-value**  **intervention*marital status interaction** |
|  | **n** | **Estimate (95% CI)** | **n** | **Estimate (95% CI)** |  |
| Active Plus I | 241 | 29.5 (-113.1; 172.0) | 1132 | -2.4 (-67.0; 62.1) | 0.689 |
| Active Plus II | 217 | 186.5 (1.1; 371.9) | 940 | 188.0 (95.5; 280.5) | 0.989 |
| Every Step Counts! | 121 | 13.1 (-8.8; 34.9) | 272 | 19.5 (6.2;32.7) | 0.624 |
| GALM | 34 | 130.2 (-35.5; 295.9) | 147 | 15.7 (-64.1; 95.4) | 0.219 |
| PACE-Lift^a^ | 54 | 41.7 (-26.3; 109.8) | 225 | 73.3 (38.3; 108.2) | 0.417 |
| PACE-UP^a^ | 321 | 61.8 (32.7; 91.0) | 619 | 37.6 (15.6; 59.6) | 0.193 |
| ProAct65+^b^ | 288 | -18.8 (-79.0; 41.4) | 387 | 2.5 (-51.5; 56.5) | 0.566 |
| PROMOTE^c^ | 87 | 13.0 (3.4; 22.6) | 260 | 6.0 (0.1; 11.9) | 0.222 |

* models adjusted for minutes of MVPA per week at T0. ** models adjusted for minutes of MVPA per week at T0 and age in years. ^a^ models additionally adjusted for practice, and multi-level adjusted for household as a random effect. ^b^ models additionally multi-level adjusted for practice as a random effect. ^c^ models additionally adjusted for community, valid wear-time, and season.

**Income-, area deprivation-, and marital status-specific intervention effects at T1 (fully adjusted models)**

| **Study** | **Income-specific intervention effects*** | | | | | | |
| --- | --- | --- | --- | --- | --- | --- | --- |
|  | **Low income** | | **Medium income** | | **High income** | | **P-value**  **intervention*income interaction** |
|  | **n** | **Estimate (95% CI)** | **n** | **Estimate (95% CI)** | **n** | **Estimate (95% CI)** |  |
| ProAct65+^b^ | 173 | -0.7 (-76.7; 75.4) | 177 | -40.3 (-114.2; 33.6) | 251 | 20.0 (-46.2; 86.1) | 0.341 |
| PROMOTE^c^ | 97 | 7.6 (-1.7; 17.0) | 104 | 3.6 (-5.6; 12.8) | 131 | 10.0 (1.5; 18.4) | 0.605 |

| **Study** | **Area deprivation-specific intervention effects*** | | | | | | |
| --- | --- | --- | --- | --- | --- | --- | --- |
|  | **High deprivation** | | **Medium deprivation** | | **Low deprivation** | | **P-value**  **intervention*area deprivation interaction** |
|  | **n** | **Estimate (95% CI)** | **n** | **Estimate (95% CI)** | **n** | **Estimate (95% CI)** |  |
| PACE-Lift^a^ | 102 | 51.9 (0.8; 102.9) | 90 | 62.6 (9.3; 115.9) | 88 | 92.9 (39.3; 146.5) | 0.549 |
| PACE-UP^a^ | 305 | 27.8 (-2.9; 58.5) | 312 | 46.0 (15.4; 76.5) | 313 | 66.2 (36.0; 96.5) | 0.212 |
| ProAct65+^b^ | 244 | -27.8 (-104.8; 49.2) | 221 | -2.4 (-75.9; 71.2) | 212 | 25.9 (-56.5; 108.2) | 0.658 |

| **Study** | **Marital status-specific intervention effects*** | | | | |
| --- | --- | --- | --- | --- | --- |
|  | **No partner** | | **With partner** | | **P-value**  **intervention*marital status interaction** |
|  | **n** | **Estimate (95% CI)** | **n** | **Estimate (95% CI)** |  |
| Active Plus I | 240 | -26.2 (-170.6; 118.2) | 1131 | -13.3 (-77.5; 50.9) | 0.873 |
| Active Plus II | 217 | 173.1 (-15.1; 361.4) | 938 | 190.0 (97.3; 282.7) | 0.875 |
| Every Step Counts! | 272 | 13.8 (-10.2; 37.9) | 121 | 20.3 (7.0; 33.7) | 0.640 |
| GALM | 34 | 169.3 (2.7; 335.8) | 147 | 11.5 (-67.6; 90.6) | 0.092 |
| PACE-Lift^a^ | 54 | 42.9 (-24.4; 110.2) | 225 | 74.8 (40.4; 109.3) | 0.465 |
| PACE-UP^a^ | 321 | 61.4 (32.3; 90.5) | 619 | 37.4 (15.5; 59.4) | 0.211 |
| ProAct65+^b^ | 288 | -18.7 (-78.8; 41.4) | 387 | 1.5 (-52.3; 55.4) | 0.362 |
| PROMOTE^c^ | 87 | 14.9 (4.8; 25.0) | 260 | 5.7 (-0.2; 11.7) | 0.130 |

* models adjusted for minutes of MVPA per week at T0, age in years, gender, and the intervention*gender interaction. ^a^ models additionally adjusted for practice and multi-level adjusted for household as a random effect. ^b^ models additionally multi-level adjusted for practice as a random effect. ^c^ models additionally adjusted for community, valid wear-time, and season.

**General and equity-specific intervention effects at T2 (minimally adjusted models)**

| **Study** | **General intervention effect*** | | **Gender-specific intervention effects**** | | | | |
| --- | --- | --- | --- | --- | --- | --- | --- |
|  |  |  | **Males** | | **Females** | | **P-value**  **intervention*gender interaction** |
|  | **n** | **Estimate (95% CI)** | **n** | **Estimate (95% CI)** | **n** | **Estimate (95% CI)** |  |
| Active Plus I | 1346 | 46.0 (-13.1; 105.2) | 585 | 1.7 (-88.9; 92.4) | 751 | 80.0 (1.1; 158.8) | 0.201 |
| Active Plus II | 1250 | 97.5 (26.8; 168.1) | 609 | 129.6 (28.3; 230.9) | 636 | 69.8 (-29.4; 168.9) | 0.407 |
| PACE-Lift^a^ | 273 | 42.9 (12.1; 73.6) | 125 | 23.9 (-20.5; 68.3) | 148 | 66.2 (25.2; 107.2) | 0.157 |
| PACE-UP^a^ | 956 | 34.3 (17.8; 50.7) | 346 | 36.2 (9.7; 62.6) | 610 | 31.5 (11.7; 51.3) | 0.774 |
| ProAct65+^b^ | 603 | 28.1 (-6.6; 62.8) | 220 | -13.0 (-69.3; 43.3) | 383 | 44.1 (1.4; 86.8) | 0.113 |

| **Study** | **Education-specific intervention effects**** | | | | | | |
| --- | --- | --- | --- | --- | --- | --- | --- |
|  | **Low education** | | **Medium education** | | **High education** | | **P-value**  **intervention*education interaction** |
|  | **n** | **Estimate (95% CI)** | **n** | **Estimate (95% CI)** | **n** | **Estimate (95% CI)** |  |
| Active Plus I | 630 | 87.2 (1.7; 172.7) | 243 | 27.5 (-110.8; 165.7) | 442 | 12.7 (-92.2; 117.6) | 0.519 |
| Active Plus II | 572 | 140.0 (37.1; 242.9) | 323 | -5.7 (-142.3; 130.9) | 341 | 136.2 (-9.8; 282.2) | 0.210 |
| PACE-Lift^a^ | 103 | 49.5 (-0.3; 99.2) | 43 | 54.6 (-21.6; 130.7) | 121 | 50.0 (4.6; 95.4) | 0.993 |
| PACE-UP^a^ | 242 | 13.7 (-18.9; 46.2) | 212 | 48.0 (15.2; 80.7) | 487 | 34.0 (11.1; 56.8) | 0.335 |
| ProAct65+^b^ | 243 | -13.8 (-67.9; 40.4) | 212 | 60.7 (3.5; 117.9) | 139 | 49.2 (-20.5; 118.9) | 0.142 |

| **Study** | **Income-specific intervention effects**** | | | | | | |
| --- | --- | --- | --- | --- | --- | --- | --- |
|  | **Low income** | | **Medium income** | | **High income** | | **P-value**  **intervention*income interaction** |
|  | **n** | **Estimate (95% CI)** | **n** | **Estimate (95% CI)** | **n** | **Estimate (95% CI)** |  |
| ProAct65+^a^ | 145 | 22.8 (-44.4; 90.1) | 159 | 15.7 (-46.6; 78.0) | 226 | 40.3 (-13.8; 94.4) | 0.820 |
| **Study** | **Area deprivation-specific intervention effects**** | | | | | | |
|  | **High deprivation** | | **Medium deprivation** | | **Low deprivation** | | **P-value**  **intervention*area deprivation interaction** |
|  | **n** | **Estimate (95% CI)** | **n** | **Estimate (95% CI)** | **n** | **Estimate (95% CI)** |  |
| PACE-Lift^c^ | 98 | 10.9 (-41.4; 63.2) | 87 | 58.2 (3.6; 112.8) | 88 | 72.1 (18.1; 126.1) | 0.243 |
| PACE-UP^c^ | 303 | 21.4 (-7.7; 50.4) | 313 | 20.4 (-8.1; 48.9) | 310 | 53.5 (25.1; 81.8) | 0.187 |
| ProAct65+^b^ | 216 | 16.2 (-45.8; 78.2) | 192 | 12.1 (-47.9; 72.1) | 195 | 31.2 (-33.0; 95.4) | 0.906 |

| **Study** | **Marital status-specific intervention effects**** | | | | |
| --- | --- | --- | --- | --- | --- |
|  | **No partner** | | **With partner** | | **P-value**  **intervention*marital status interaction** |
|  | **n** | **Estimate (95% CI)** | **n** | **Estimate (95% CI)** |  |
| Active Plus I | 235 | 60.3 (-81.0; 201.6) | 1079 | 52.3 (-13.3; 117.9) | 0.920 |
| Active Plus II | 218 | 35.5 (-131.5; 202.5) | 1023 | 114.0 (35.3; 192.7) | 0.404 |
| PACE-Lift^a^ | 53 | 18.2 (-50.8; 87.2) | 219 | 52.7 (17.9; 87.4) | 0.380 |
| PACE-UP^a^ | 321 | 48.6 (21.2; 76.0) | 621 | 22.5 (1.8; 43.1) | 0.135 |
| ProAct65+^b^ | 256 | 13.5 (-39.2; 66.2) | 346 | 33.0 (-12.0; 78.0) | 0.580 |

* models adjusted for minutes of MVPA per week at T0. ** models adjusted for minutes of MVPA per week at T0 and age in years. ^a^ models additionally adjusted for practice and multi-level adjusted for household as a random effect. ^b^ models additionally multi-level adjusted for practice as a random effect.

General and equity-specific intervention effects at T2 (fully adjusted models)

| **Study** | **General intervention effect*** | | **Gender-specific intervention effects**** | | | | |
| --- | --- | --- | --- | --- | --- | --- | --- |
|  |  |  | **Males** | | **Females** | | **P-value**  **intervention*gender interaction** |
|  | **n** | **Estimate (95% CI)** | **n** | **Estimate (95% CI)** | **n** | **Estimate (95% CI)** |  |
| Active Plus I | 1313 | 50.4 (-9.6; 110.4) | 577 | 4.6 (-87.8; 97.1) | 736 | 70.7 (-15.5; 156.8) | 0.291 |
| Active Plus II | 1233 | 98.2 (26.6; 169.9) | 604 | 121.1 (18.1; 224.0) | 629 | 54.3 (-52.6; 161.2) | 0.372 |
| PACE-Lift^a^ | 267 | 51.1 (20.0; 82.2) | 121 | 32.6 (-12.4; 77.7) | 146 | 65.7 (24.7; 106.8) | 0.259 |
| PACE-UP^a^ | 941 | 32.2 (15.7; 48.6) | 341 | 33.9 (7.3; 60.5) | 600 | 30.7 (10.8; 50.7) | 0.801 |
| ProAct65+^b^ | 594 | 27.8 (-6.5; 62.2) | 216 | -7.7 (-64.2; 48.7) | 378 | 47.2 (4.5; 89.9) | 0.125 |

| **Study** | **Education-specific intervention effects***** | | | | | | |
| --- | --- | --- | --- | --- | --- | --- | --- |
|  | **Low education** | | **Medium education** | | **High education** | | **P-value**  **intervention*education interaction** |
|  | **n** | **Estimate (95% CI)** | **n** | **Estimate (95% CI)** | **n** | **Estimate (95% CI)** |  |
| Active Plus I | 630 | 76.5 (-11.3; 164.2) | 242 | 24.2 (-114.9; 163.2) | 441 | 12.3 (-92.7; 117.4) | 0.622 |
| Active Plus II | 572 | 148.7 (44.2; 253.2) | 321 | -17.0 (-155.1; 121.1) | 340 | 131.3 (-14.9; 277.5) | 0.156 |
| PACE-Lift^a^ | 103 | 49.9 (0.3; 99.5) | 43 | 50.2 (-25.6; 126.0) | 121 | 51.7 (6.4; 96.9) | 0.964 |
| PACE-UP^a^ | 242 | 13.6 (-19.0; 46.1) | 212 | 47.9 (15.1; 80.7) | 487 | 34.0 (11.2; 56.8) | 0.329 |
| ProAct65+^b^ | 243 | -13.7 (-67.6; 40.1) | 212 | 60.0 (3.2; 116.9) | 139 | 48.9 (-20.5; 118.2) | 0.107 |

| **Study** | **Income-specific intervention effects***** | | | | | | |
| --- | --- | --- | --- | --- | --- | --- | --- |
|  | **Low income** | | **Medium income** | | **High income** | | **P-value**  **intervention*income interaction** |
|  | **n** | **Estimate (95% CI)** | **n** | **Estimate (95% CI)** | **n** | **Estimate (95% CI)** |  |
| ProAct65+^a^ | 145 | 23.2 (-43.8; 90.1) | 159 | 14.3 (-47.8; 76.4) | 226 | 38.5 (-15.4; 92.4) | 0.766 |

| **Study** | **Area deprivation-specific intervention effects***** | | | | | | |
| --- | --- | --- | --- | --- | --- | --- | --- |
|  | **High deprivation** | | **Medium deprivation** | | **Low deprivation** | | **P-value**  **intervention*area deprivation interaction** |
|  | **n** | **Estimate (95% CI)** | **n** | **Estimate (95% CI)** | **n** | **Estimate (95% CI)** |  |
| PACE-Lift^c^ | 98 | 12.6 (-39.6; 64.8) | 87 | 59.0 (4.6; 113.4) | 88 | 70.5 (16.6; 124.4) | 0.292 |
| PACE-UP^c^ | 303 | 21.4 (-7.7; 50.4) | 313 | 20.0 (-8.5; 48.5) | 310 | 53.4 (25.1; 81.7) | 0.184 |
| ProAct65+^b^ | 216 | 12.9 (-48.7; 74.5) | 192 | 13.7 (-46.0; 73.3) | 195 | 29.7 (-34.1; 93.5) | 0.933 |

| **Study** | **Marital status-specific intervention effects***** | | | | |
| --- | --- | --- | --- | --- | --- |
|  | **No partner** | | **With partner** | | **P-value**  **intervention*marital status interaction** |
|  | **n** | **Estimate (95% CI)** | **n** | **Estimate (95% CI)** |  |
| Active Plus I | 234 | 41.6 (-103.2; 186.4) | 1078 | 48.6 (-17.2; 114.4) | 0.931 |
| Active Plus II | 218 | 43.3 (-126.1; 212.8) | 1020 | 111.9 (32.9; 190.8) | 0.473 |
| PACE-Lift^a^ | 53 | 20.3 (-48.2; 88.8) | 219 | 53.2 (18.5; 87.8) | 0.313 |
| PACE-UP^a^ | 321 | 48.6 (21.2; 76.0) | 621 | 22.5 (1.9; 43.1) | 0.119 |
| ProAct65+^b^ | 256 | 13.4 (-39.0; 65.7) | 346 | 32.7 (-12.0;77.4) | 0.344 |

* models adjusted for minutes of MVPA per week at T0, age in years, gender, and education. ** models adjusted for minutes of MVPA per week at T0, age in years, education, and the intervention*education interaction. *** models adjusted for minutes of MVPA per week at T0, age in years, gender, and the intervention*gender interaction. ^a^ models additionally adjusted for practice and multi-level adjusted for household as a random effect. ^b^ models additionally multi-level adjusted for practice as a random effect.

**Sensitivity analysis: General and equity-specific intervention effects at T1** **using weekly minutes of MVPA in bouts of at least 10 min as the outcome (minimally adjusted models)**

| **Study** | **General intervention effect*** | | **Gender-specific intervention effects**** | | | | |
| --- | --- | --- | --- | --- | --- | --- | --- |
|  |  |  | **Males** | | **Females** | | **P-value**  **intervention*gender interaction** |
|  | **n** | **Estimate (95% CI)** | **n** | **Estimate (95% CI)** | **n** | **Estimate (95% CI)** |  |
| PACE-Lift | 280 | 58.6 (34.7; 82.5) | 129 | 72.3 (38.6; 106.0) | 151 | 56.3 (25.1; 87.4) | 0.476 |
| PACE-UP | 954 | 51.9 (37.0; 66.7) | 345 | 53.7 (30.1; 77.4) | 609 | 50.0 (32.3; 67.7) | 0.793 |

| **Study** | **Education-specific intervention effects**** | | | | | | |
| --- | --- | --- | --- | --- | --- | --- | --- |
|  | **Low education** | | **Medium education** | | **High education** | | **P-value**  **intervention*education interaction** |
|  | **n** | **Estimate (95% CI)** | **n** | **Estimate (95% CI)** | **n** | **Estimate (95% CI)** |  |
| PACE-Lift | 110 | 81.1 (43.7; 118.4) | 44 | 19.6 (-38.6; 77.7) | 121 | 70.1 (34.9; 105.4) | 0.198 |
| PACE-UP | 247 | 33.7 (4.6; 62.7) | 210 | 77.8 (48.3; 107.3) | 482 | 49.7 (29.1; 70.4) | 0.097 |

| **Study** | **Area deprivation-specific intervention effects**** | | | | | | |
| --- | --- | --- | --- | --- | --- | --- | --- |
|  | **High deprivation** | | **Medium deprivation** | | **Low deprivation** | | **P-value**  **intervention*area deprivation interaction** |
|  | **n** | **Estimate (95% CI)** | **n** | **Estimate (95% CI)** | **n** | **Estimate (95% CI)** |  |
| PACE-Lift | 102 | 32.8 (-6.8; 72.3) | 90 | 59.0 (17.6; 100.3) | 88 | 98.9 (57.4; 140.3) | 0.079 |
| PACE-UP | 305 | 42.4 (16.2; 68.5) | 312 | 38.2 (12.2; 64.2) | 313 | 74.8 (49.0; 100.5) | 0.100 |

| **Study** | **Marital status-specific intervention effects**** | | | | |
| --- | --- | --- | --- | --- | --- |
|  | **No partner** | | **With partner** | | **P-value**  **intervention*marital status interaction** |
|  | **n** | **Estimate (95% CI)** | **n** | **Estimate (95% CI)** |  |
| PACE-Lift | 54 | 45.9 (-7.1; 99.0) | 225 | 66.6 (39.6; 93.6) | 0.495 |
| PACE-UP | 321 | 56.0 (31.3; 80.7) | 619 | 48.0 (29.1; 66.9) | 0.615 |

* models adjusted for minutes of MVPA per week at T0, practice, and multi-level adjusted for household as a random effect. ** models adjusted for minutes of MVPA per week at T0, age in years, practice, and multi-level adjusted for household as a random effect.

Sensitivity analysis: General and equity-specific intervention effects at T1 using weekly minutes of MVPA in bouts of at least 10 min as the outcome (fully adjusted models)

| **Study** | **General intervention effect*** | | **Gender-specific intervention effects**** | | | | |
| --- | --- | --- | --- | --- | --- | --- | --- |
|  |  |  | **Males** | | **Females** | | **P-value**  **intervention*gender interaction** |
|  | **n** | **Estimate (95% CI)** | **n** | **Estimate (95% CI)** | **n** | **Estimate (95% CI)** |  |
| PACE-Lift | 275 | 67.5 (43.7; 91.4) | 125 | 80.9 (47.0; 114.7) | 150 | 56.0 (25.1; 86.8) | 0.340 |
| PACE-UP | 939 | 52.1 (37.2; 67.0) | 341 | 54.8 (31.2; 78.5) | 598 | 50.0 (32.2; 67.8) | 0.625 |

| **Study** | **Education-specific intervention effects***** | | | | | | |
| --- | --- | --- | --- | --- | --- | --- | --- |
|  | **Low education** | | **Medium education** | | **High education** | | **P-value**  **intervention*education interaction** |
|  | **n** | **Estimate (95% CI)** | **n** | **Estimate (95% CI)** | **n** | **Estimate (95% CI)** |  |
| PACE-Lift | 110 | 82.3 (45.4; 119.2) | 44 | 17.3 (-40.3; 74.9) | 121 | 71.9 (37.1; 106.7) | 0.182 |
| PACE-UP | 247 | 33.5 (4.5; 62.5) | 210 | 78.3 (48.9; 107.8) | 482 | 49.5 (28.9; 70.1) | 0.083 |

| **Study** | **Area deprivation-specific intervention effects***** | | | | | | |
| --- | --- | --- | --- | --- | --- | --- | --- |
|  | **High deprivation** | | **Medium deprivation** | | **Low deprivation** | | **P-value**  **intervention*area deprivation interaction** |
|  | **n** | **Estimate (95% CI)** | **n** | **Estimate (95% CI)** | **n** | **Estimate (95% CI)** |  |
| PACE-Lift | 102 | 34.8 (-4.4; 74.0) | 90 | 60.7 (19.8; 101.7) | 88 | 97.3 (56.2; 138.3) | 0.100 |
| PACE-UP | 305 | 42.4 (16.3; 68.5) | 312 | 37.3 (11.4; 63.3) | 313 | 74.6 (49.0; 100.3) | 0.094 |

| **Study** | **Marital status-specific intervention effects***** | | | | |
| --- | --- | --- | --- | --- | --- |
|  | **No partner** | | **With partner** | | **P-value**  **intervention*marital status interaction** |
|  | **n** | **Estimate (95% CI)** | **n** | **Estimate (95% CI)** |  |
| PACE-Lift | 54 | 47.0 (-5.5; 99.6) | 225 | 67.6 (41.0; 94.3) | 0.546 |
| PACE-UP | 321 | 55.5 (30.8; 80.1) | 619 | 48.1 (29.2; 66.9) | 0.605 |

* models adjusted for minutes of MVPA per week at T0, age in years, gender, education, practice, and multi-level adjusted for household as a random effect. ** models adjusted for minutes of MVPA per week at T0, age in years, education, the intervention*education interaction, practice, and multi-level adjusted for household as a random effect. *** models adjusted for minutes of MVPA per week at T0, age in years, gender, the intervention*gender interaction, practice, and multi-level adjusted for household as a random effect.

Sensitivity analysis: General and equity-specific intervention effects at T2 using weekly minutes of MVPA in bouts of at least 10 min as the outcome (minimally adjusted models)

| **Study** | **General intervention effect*** | | **Gender-specific intervention effects**** | | | | |
| --- | --- | --- | --- | --- | --- | --- | --- |
|  |  |  | **Males** | | **Females** | | **P-value**  **intervention*gender interaction** |
|  | **n** | **Estimate (95% CI)** | **n** | **Estimate (95% CI)** | **n** | **Estimate (95% CI)** |  |
| PACE-Lift | 273 | 38.0 (14.4; 61.6) | 125 | 52.1 (22.2; 82.0) | 148 | 33.8 (5.7; 61.8) | 0.277 |
| PACE-UP | 956 | 33.8 (20.1; 47.6) | 346 | 48.4 (26.4; 70.5) | 610 | 25.2 (8.6; 41.7) | 0.085 |

| **Study** | **Education-specific intervention effects**** | | | | | | |
| --- | --- | --- | --- | --- | --- | --- | --- |
|  | **Low education** | | **Medium education** | | **High education** | | **P-value**  **intervention*education interaction** |
|  | **n** | **Estimate (95% CI)** | **n** | **Estimate (95% CI)** | **n** | **Estimate (95% CI)** |  |
| PACE-Lift | 103 | 41.1 (5.0; 77.1) | 43 | 50.5 (-1.0; 102.0) | 121 | 44.8 (11.7; 77.9) | 0.951 |
| PACE-UP | 242 | 14.6 (-12.6; 41.7) | 212 | 57.1 (29.8; 84.3) | 487 | 31.4 (12.4; 50.4) | 0.086 |

| **Study** | **Area deprivation-specific intervention effects**** | | | | | | |
| --- | --- | --- | --- | --- | --- | --- | --- |
|  | **High deprivation** | | **Medium deprivation** | | **Low deprivation** | | **P-value**  **intervention*area deprivation interaction** |
|  | **n** | **Estimate (95% CI)** | **n** | **Estimate (95% CI)** | **n** | **Estimate (95% CI)** |  |
| PACE-Lift | 98 | 22.7 (-17.4; 62.9) | 87 | 61.2 (19.6; 102.8) | 88 | 42.7 (1.4; 84.1) | 0.416 |
| PACE-UP | 303 | 26.5 (2.0; 50.9) | 313 | 18.3 (-5.7; 42.3) | 310 | 53.7 (29.8; 77.5) | 0.100 |

| **Study** | **Marital status-specific intervention effects**** | | | | |
| --- | --- | --- | --- | --- | --- |
|  | **No partner** | | **With partner** | | **P-value**  **intervention*marital status interaction** |
|  | **n** | **Estimate (95% CI)** | **n** | **Estimate (95% CI)** |  |
| PACE-Lift | 53 | 30.4 (-20.3; 81.1) | 219 | 44.0 (16.9; 71.1) | 0.641 |
| PACE-UP | 321 | 41.5 (18.6; 64.4) | 621 | 27.9 (10.6; 45.3) | 0.356 |

* models adjusted for minutes of MVPA per week at T0, practice, and multi-level adjusted for household as a random effect. ** models adjusted for minutes of MVPA per week at T0, age in years, practice, and multi-level adjusted for household as a random effect.

Sensitivity analysis: General and equity-specific intervention effects at T2 using weekly minutes of MVPA in bouts of at least 10 min as the outcome (fully adjusted models)

| **Study** | **General intervention effect*** | | **Gender-specific intervention effects**** | | | | |
| --- | --- | --- | --- | --- | --- | --- | --- |
|  |  |  | **Males** | | **Females** | | **P-value**  **intervention*gender interaction** |
|  | **n** | **Estimate (95% CI)** | **n** | **Estimate (95% CI)** | **n** | **Estimate (95% CI)** |  |
| PACE-Lift | 267 | 44.9 (20.9; 69.0) | 121 | 58.2 (27.8; 88.7) | 146 | 34.4 (6.1; 62.7) | 0.159 |
| PACE-UP | 941 | 33.2 (19.5; 47.0) | 341 | 46.6 (24.6; 68.6) | 600 | 25.4 (8.9; 42.0) | 0.090 |

| **Study** | **Education-specific intervention effects***** | | | | | | |
| --- | --- | --- | --- | --- | --- | --- | --- |
|  | **Low education** | | **Medium education** | | **High education** | | **P-value**  **intervention*education interaction** |
|  | **n** | **Estimate (95% CI)** | **n** | **Estimate (95% CI)** | **n** | **Estimate (95% CI)** |  |
| PACE-Lift | 103 | 43.1 (7.2; 79.0) | 43 | 48.3 (-3.2; 99.9) | 121 | 45.9 (12.9; 78.9) | 0.962 |
| PACE-UP | 242 | 14.9 (-12.2; 42.0) | 212 | 57.3 (30.0; 84.5) | 487 | 31.6 (12.7; 50.6) | 0.070 |

| **Study** | **Area deprivation-specific intervention effects***** | | | | | | |
| --- | --- | --- | --- | --- | --- | --- | --- |
|  | **High deprivation** | | **Medium deprivation** | | **Low deprivation** | | **P-value**  **intervention*area deprivation interaction** |
|  | **n** | **Estimate (95% CI)** | **n** | **Estimate (95% CI)** | **n** | **Estimate (95% CI)** |  |
| PACE-Lift | 98 | 24.5 (-15.5; 64.5) | 87 | 62.9 (21.5; 104.3) | 88 | 41.8 (0.7; 82.8) | 0.375 |
| PACE-UP | 303 | 26.5 (2.1; 51.0) | 313 | 18.6 (-5.4; 42.6) | 310 | 53.9 (30.1; 77.7) | 0.104 |

| **Study** | **Marital status-specific intervention effects***** | | | | |
| --- | --- | --- | --- | --- | --- |
|  | **No partner** | | **With partner** | | **P-value**  **intervention*marital status interaction** |
|  | **n** | **Estimate (95% CI)** | **n** | **Estimate (95% CI)** |  |
| PACE-Lift | 53 | 31.2 (-19.2; 81.6) | 219 | 44.7 (17.8; 71.7) | 0.710 |
| PACE-UP | 321 | 41.6 (18.7; 64.5) | 621 | 28.2 (10.9; 45.5) | 0.215 |

* models adjusted for minutes of MVPA per week at T0, age in years, gender, education, practice, and multi-level adjusted for household as a random effect. ** models adjusted for minutes of MVPA per week at T0, age in years, education, the intervention*education interaction, practice, and multi-level adjusted for household as a random effect. *** models adjusted for minutes of MVPA per week at T0, age in years, gender, the intervention*gender interaction, practice, and multi-level adjusted for household as a random effect.
