# Supplementary material for: Equity-specific effects of interventions to promote physical activity among middle-aged and older adults: results from applying a novel equity-specific re-analysis strategy
Source: Int J Behav Nutr Phys Act. 2021 May 17;18:65. doi: 10.1186/s12966-021-01131-w (PMC8130354; doi:10.1186/s12966-021-01131-w)
Supplement: Supplementary file 8 — Additional file 8. Detailed results of the meta-analyses using raw coefficients. This file contains the complete output of the random-effects meta-analysis (main and secondary analysis) using raw coefficients. [file 12966_2021_1131_MOESM8_ESM.pdf]

Random-effects meta-analysis

```
library(readxl)
library(metafor)
```

```
## Loading required package: Matrix

## Loading 'metafor' package (version 2.4-0). For an overview
## and introduction to the package please type: help(metafor).
```

```
data_EQUAL_meta = read_excel("data_EQUAL_meta.xlsx")
attach(data_EQUAL_meta)
```

Gender-specific intervention effects

Model B0T1 (data from 8 studies; 7 randomized, 1 non-randomized; 3 with objective physical activity measure, 5 with subjective physical activity measure): The moderated effect of the intervention through gender (reference: male) at T1 (follow-up time-point closest to intervention end point), adjusted for physical activity level at T0 (baseline) and age:

```
mod_gnd_con_B0T1 = rma(yi=data_EQUAL_meta$X_GND_CON_B0T1,
                       sei=data_EQUAL_meta$X_GND_CON_B0T1/data_EQUAL_meta$t_X_GND_CON_B0T1,
                       method='DL',slab=Study)
summary(mod_gnd_con_B0T1)
```

```
##
## Random-Effects Model (k = 8; tau^2 estimator: DL)
##
##   logLik  deviance       AIC       BIC      AICc
## -44.6488   21.0861   93.2976   93.4565   95.6976
##
## tau^2 (estimated amount of total heterogeneity): 521.1622 (SE = 618.9129)
## tau (square root of estimated tau^2 value):      22.8290
## I^2 (total heterogeneity / total variability):   61.34%
## H^2 (total variability / sampling variability):  2.59
##
## Test for Heterogeneity:
## Q(df = 7) = 18.1067, p-val = 0.0115
##
## Model Results:
##
## estimate       se    zval    pval     ci.lb    ci.ub
##   7.6388  12.5412  0.6091  0.5425  -16.9414  32.2190
##
## ---
## Signif. codes:  0 '***' 0.001 '**' 0.01 '*' 0.05 '.' 0.1 ' ' 1
```

```
forest(mod_gnd_con_B0T1,main="Forest plot",header='Study',xlab = '')
mtext(c("Favors males", "Favors females"), side=1, line=3, at=c(-20,20),adj=c(1,0))
arrows(x0=c(-20,20), y0=-4.4, x1=c(-200, 200), y1=-4.4, length=0.1, lwd=2, xpd=TRUE)
```

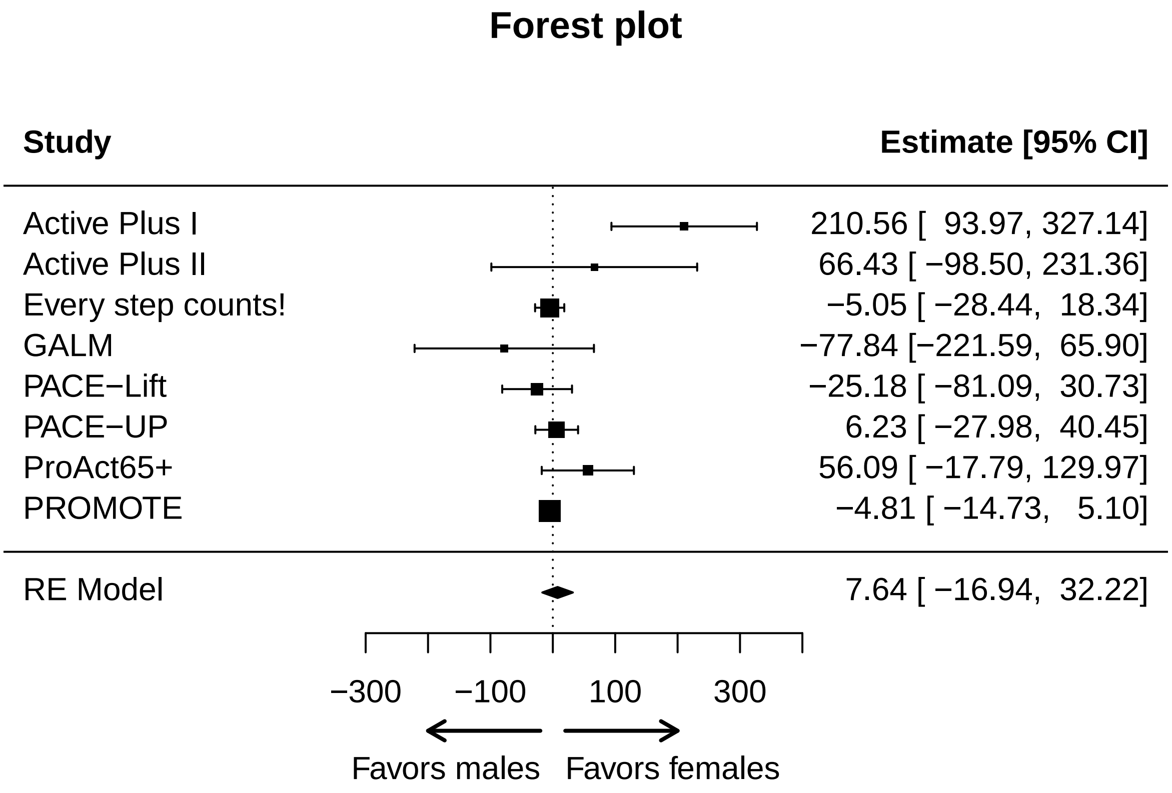


```
funnel(mod_gnd_con_BOT1,legend=TRUE,main='Funnel plot')
```

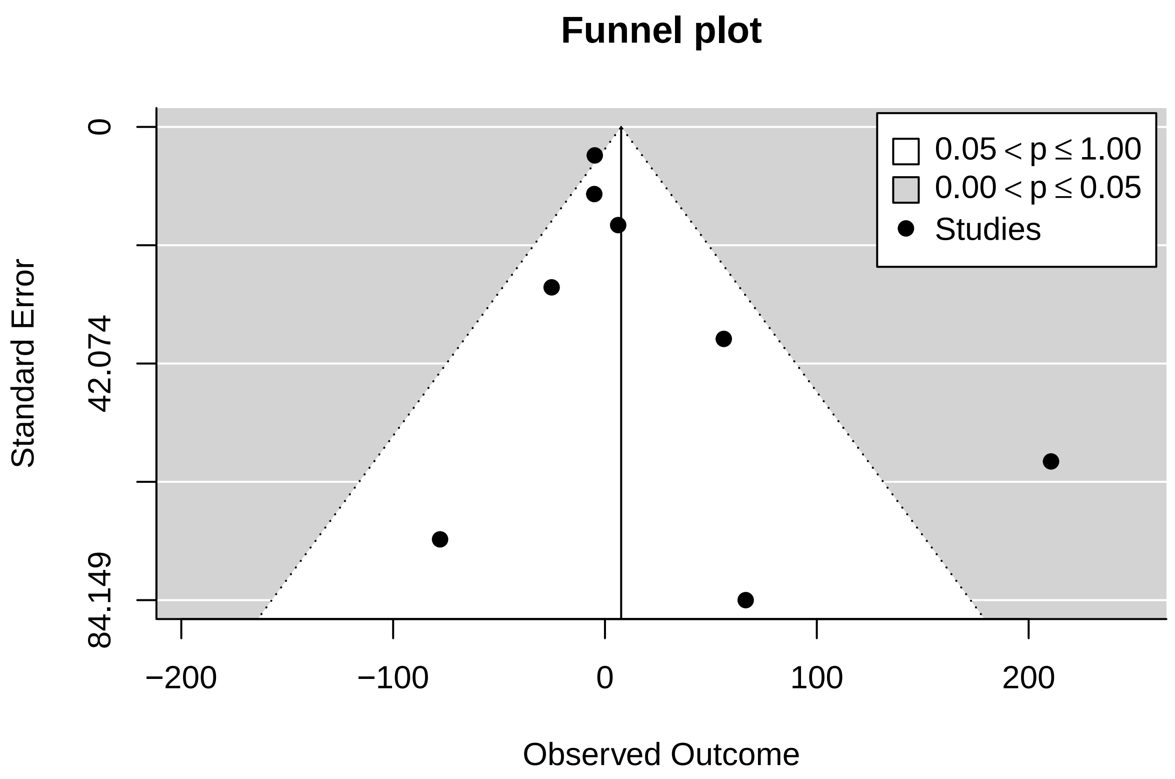

Model B2T1 (data from 8 studies; 7 randomized, 1 non-randomized; 3 with objective physical activity measure, 5 with subjective physical activity measure): The moderated effect of the intervention through gender (reference: male) at T1, adjusted for physical activity level at T0, age, education, and the condition x education interaction:

```
mod_gnd_con_B2T1 = rma(yi=data_EQUAL_meta$X_GND_CON_B2T1,
                       sei=data_EQUAL_meta$X_GND_CON_B2T1/data_EQUAL_meta$t_X_GND_CON_B2T1,
                       method='DL',slab=Study)
summary(mod_gnd_con_B2T1)
```

```
##
## Random-Effects Model (k = 8; tau^2 estimator: DL)
##
##   logLik  deviance       AIC       BIC      AICc
## -45.4653   22.5301   94.9305   95.0894   97.3305
##
## tau^2 (estimated amount of total heterogeneity): 601.9298 (SE = 696.3036)
## tau (square root of estimated tau^2 value):      24.5343
## I^2 (total heterogeneity / total variability):   64.11%
## H^2 (total variability / sampling variability):  2.79
##
## Test for Heterogeneity:
## Q(df = 7) = 19.5037, p-val = 0.0067
##
## Model Results:
##
## estimate       se     zval     pval      ci.lb     ci.ub
```

```
##   5.1265  13.1928  0.3886  0.6976  -20.7309  30.9839
##
## ---
## Signif. codes:  0 '***' 0.001 '**' 0.01 '*' 0.05 '.' 0.1 ' ' 1
```

```
forest(mod_gnd_con_B2T1,main="Forest plot",header='Study',xlab='')
mtext(c("Favors males", "Favors females"), side=1, line=3, at=c(-20,20),adj=c(1,0))
arrows(x0=c(-20,20), y0=-4.4, x1=c(-200, 200), y1=-4.4, length=0.1, lwd=2, xpd=TRUE)
```

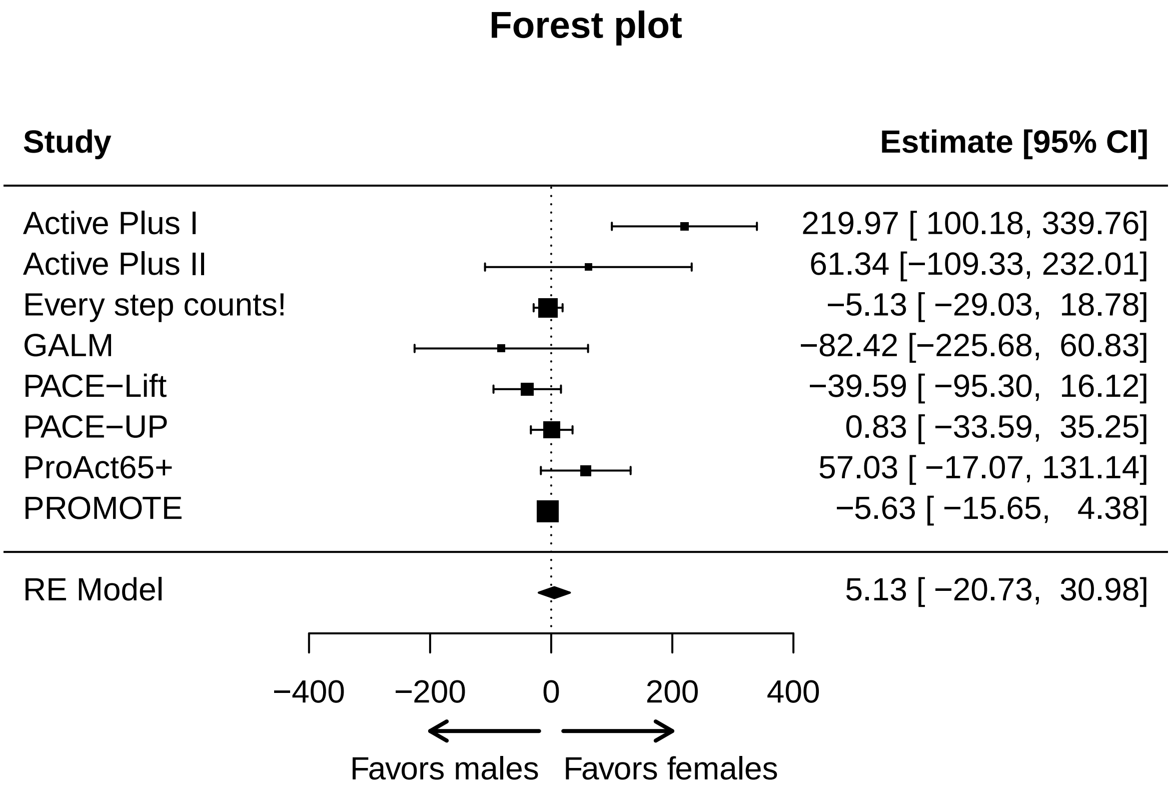


```
funnel(mod_gnd_con_B2T1,legend=TRUE,main='Funnel plot')
```

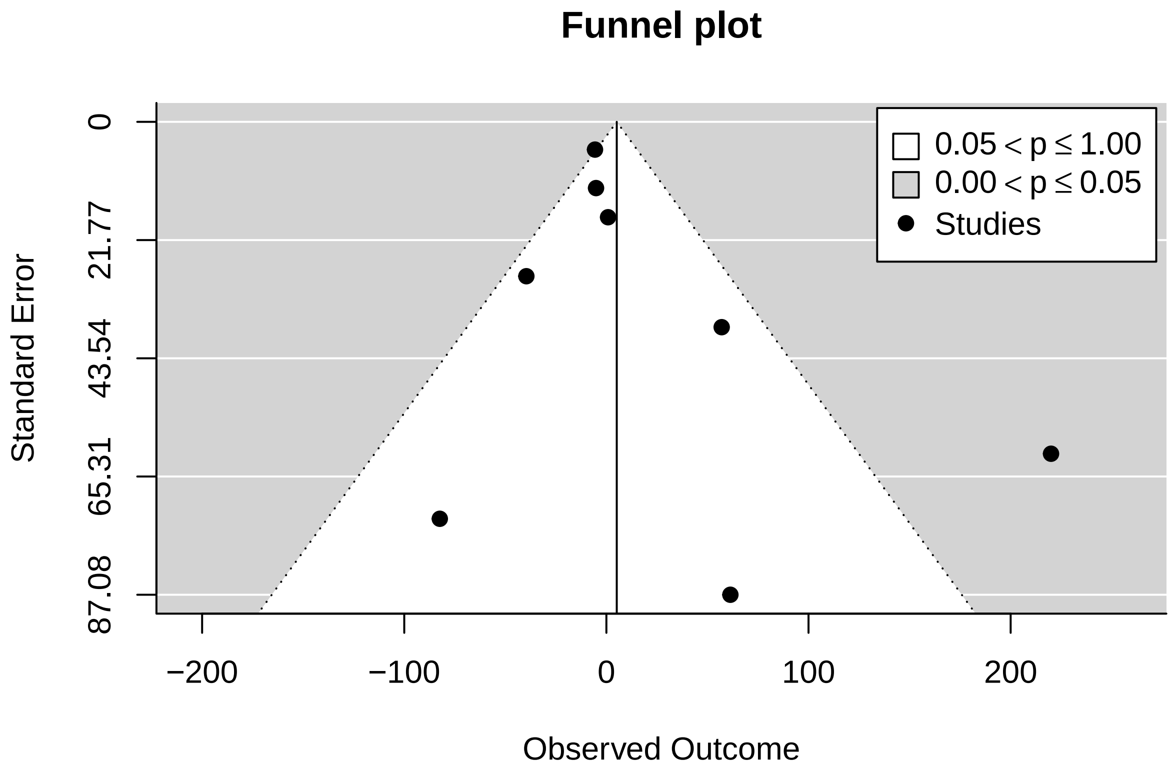

Model B0T2 (data from 5 studies; 5 randomized, 0 non-randomized; 2 with objective physical activity measure, 3 with subjective physical activity measure): The moderated effect of the intervention through gender (reference: male) at T2 (next follow-up assessment), adjusted for physical activity level at T0 and age

```
mod_gnd_con_B0T2 = rma(yi=data_EQUAL_meta$X_GND_CON_B0T2,
                       sei=data_EQUAL_meta$X_GND_CON_B0T2/data_EQUAL_meta$t_X_GND_CON_B0T2,
                       method='DL',slab=Study)
```

```
## Warning in rma(yi = data_EQUAL_meta$X_GND_CON_B0T2, sei =
## data_EQUAL_meta$X_GND_CON_B0T2/data_EQUAL_meta$t_X_GND_CON_B0T2, : Studies with
## NAs omitted from model fitting.
```

```
summary(mod_gnd_con_B0T2)
```

```
##
## Random-Effects Model (k = 5; tau^2 estimator: DL)
##
##   logLik  deviance       AIC       BIC      AICc
## -25.7039    5.8907   55.4079   54.6268   61.4079
##
## tau^2 (estimated amount of total heterogeneity): 515.7280 (SE = 1214.8656)
## tau (square root of estimated tau^2 value):      22.7096
## I^2 (total heterogeneity / total variability):   30.95%
## H^2 (total variability / sampling variability):  1.45
##
## Test for Heterogeneity:
## Q(df = 4) = 5.7933, p-val = 0.2151
##
```

```
## Model Results:
##
## estimate       se    zval    pval     ci.lb    ci.ub
##  21.4838  18.4062  1.1672  0.2431  -14.5917  57.5592
##
## ---
## Signif. codes:  0 '***' 0.001 '**' 0.01 '*' 0.05 '.' 0.1 ' ' 1
```

```
forest(mod_gnd_con_BOT2,main="Forest plot",header='Study',xlab='')
mtext(c("Favors males", "Favors females"), side=1, line=3, at=c(-20,20),adj=c(1,0))
arrows(x0=c(-20,20), y0=-3.8, x1=c(-200, 200), y1=-3.8, length=0.1, lwd=2, xpd=TRUE)
```

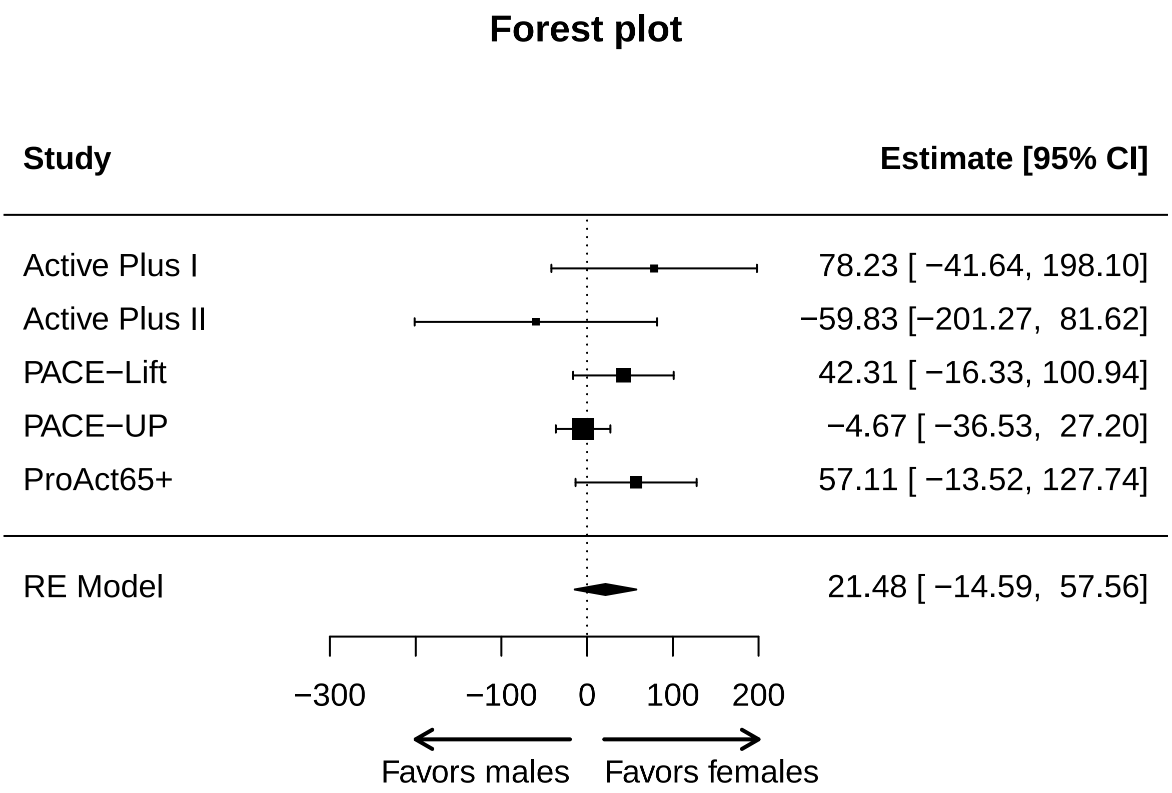


```
funnel(mod_gnd_con_BOT2,legend=TRUE,main='Funnel plot')
```

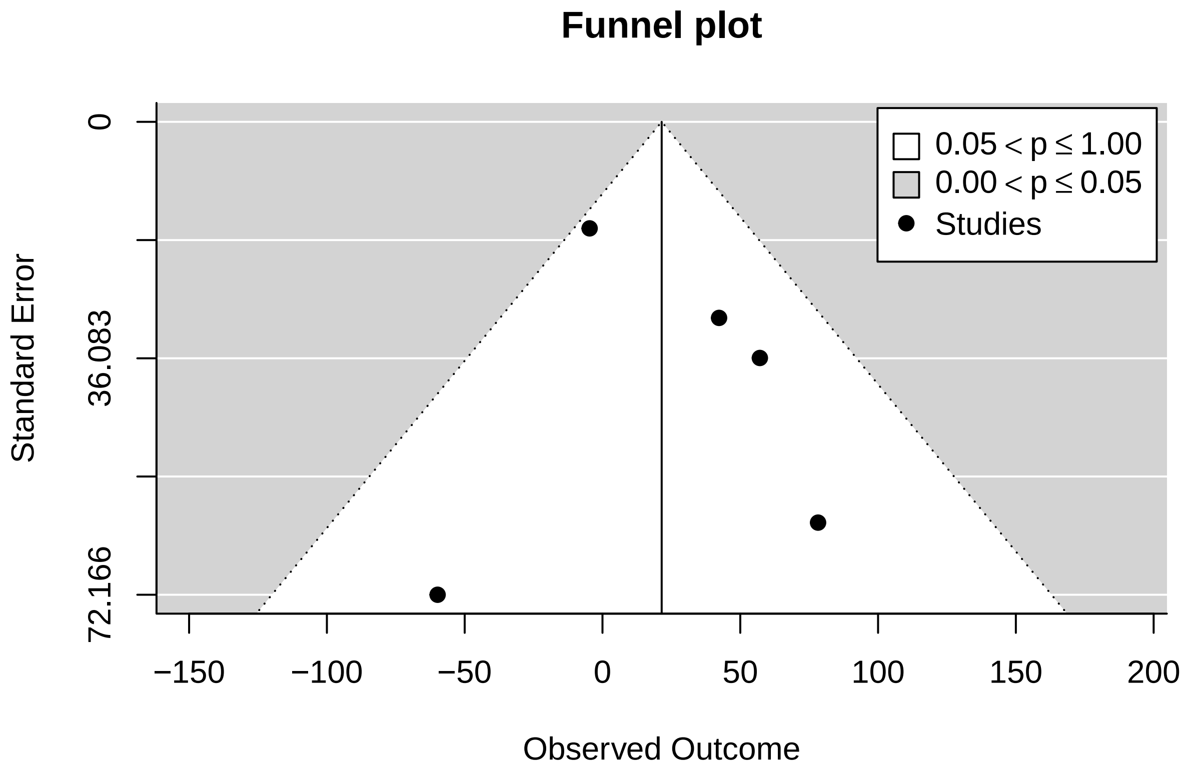

Model B2T2 (data from 5 studies; 5 randomized, 0 non-randomized; 2 with objective physical activity measure, 3 with subjective physical activity measure): The moderated effect of the intervention through gender (reference: male) at T2, adjusted for physical activity level at T0, age, education, and the condition x education interaction

```
mod_gnd_con_B2T2 = rma(yi=data_EQUAL_meta$X_GND_CON_B2T2,
                       sei=data_EQUAL_meta$X_GND_CON_B2T2/data_EQUAL_meta$t_X_GND_CON_B2T2,
                       method='DL',slab=Study)
```

```
## Warning in rma(yi = data_EQUAL_meta$X_GND_CON_B2T2, sei =
## data_EQUAL_meta$X_GND_CON_B2T2/data_EQUAL_meta$t_X_GND_CON_B2T2, : Studies with
## NAs omitted from model fitting.
```

```
summary(mod_gnd_con_B2T2)
```

```
##
## Random-Effects Model (k = 5; tau^2 estimator: DL)
##
##    logLik  deviance       AIC       BIC      AICc
## -25.3700    5.0487   54.7400   53.9589   60.7400
##
## tau^2 (estimated amount of total heterogeneity): 260.1575 (SE = 1031.7693)
## tau (square root of estimated tau^2 value):      16.1294
## I^2 (total heterogeneity / total variability):   18.02%
## H^2 (total variability / sampling variability):  1.22
##
## Test for Heterogeneity:
## Q(df = 4) = 4.8794, p-val = 0.2999
```

```
##
## Model Results:
##
## estimate       se     zval     pval      ci.lb    ci.ub
##  17.2465  16.2688  1.0601  0.2891  -14.6398  49.1327
##
## ---
## Signif. codes:  0 '***' 0.001 '**' 0.01 '*' 0.05 '.' 0.1 ' ' 1
```

```r
forest(mod_gnd_con_B2T2,main="Forest plot",header='Study',xlab='')
mtext(c("Favors males", "Favors females"), side=1, line=3, at=c(-20,20),adj=c(1,0))
arrows(x0=c(-20,20), y0=-3.8, x1=c(-200, 200), y1=-3.8, length=0.1, lwd=2, xpd=TRUE)
```

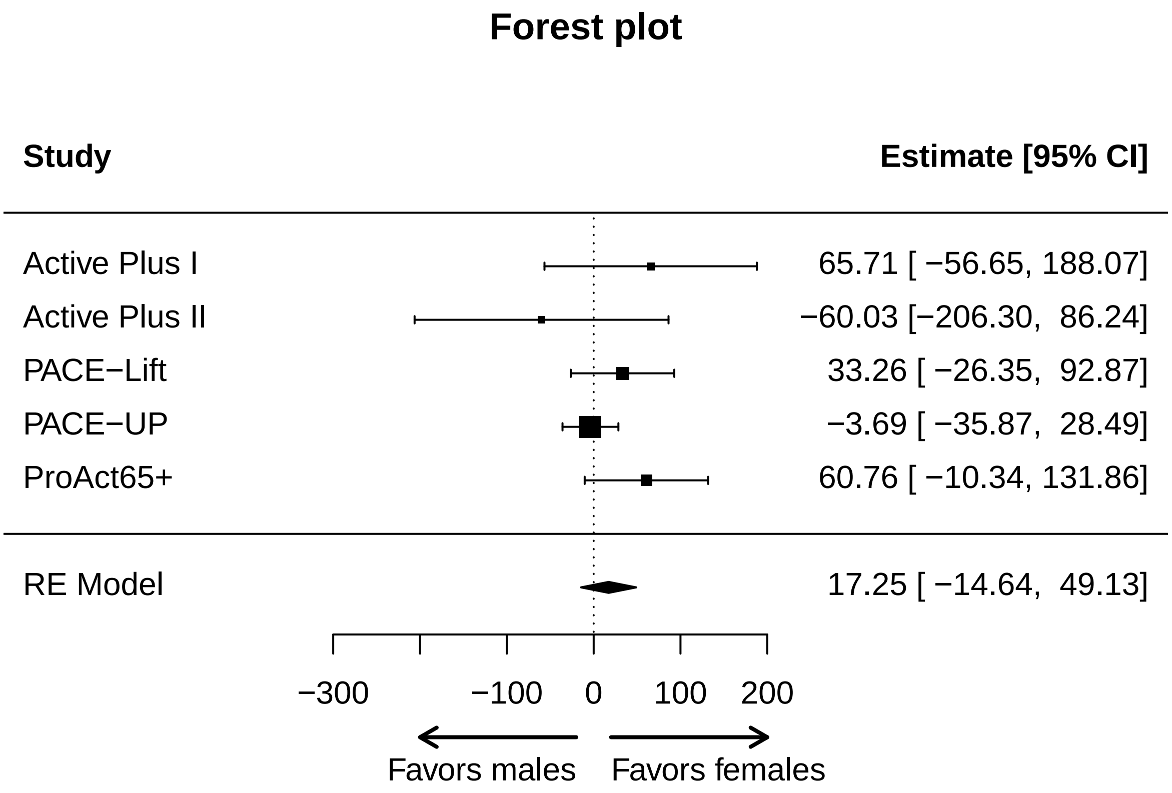


```r
funnel(mod_gnd_con_B2T2,legend=TRUE,main='Funnel plot')
```

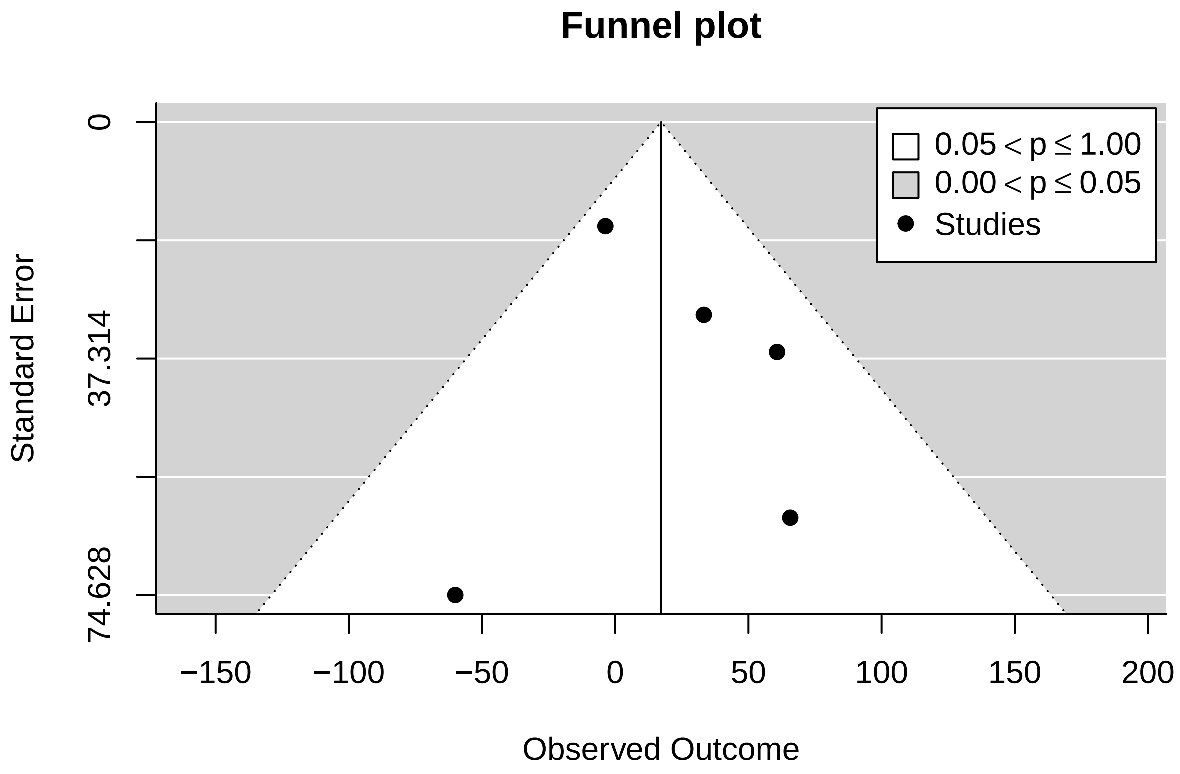

Education-specific intervention effects

Model C0T1 (data from 8 studies; 7 randomized, 1 non-randomized; 3 with objective physical activity measure, 5 with subjective physical activity measure): The moderated effect of the intervention through education (reference: medium/high) at T2, adjusted for physical activity level at T0 and age

```
mod_edu_con_C0T1 = rma(yi=data_EQUAL_meta$X_EDU_CON_C0T1,
                       sei=data_EQUAL_meta$X_EDU_CON_C0T1/data_EQUAL_meta$t_X_EDU_CON_C0T1,
                       method='DL',slab=Study)
summary(mod_edu_con_C0T1)
```

```
##
## Random-Effects Model (k = 8; tau^2 estimator: DL)
##
##   logLik  deviance       AIC       BIC      AICc
## -41.5474   11.7223   87.0949   87.2538   89.4949
##
## tau^2 (estimated amount of total heterogeneity): 481.7621 (SE = 686.0676)
## tau (square root of estimated tau^2 value):      21.9491
## I^2 (total heterogeneity / total variability):   41.05%
## H^2 (total variability / sampling variability):  1.70
##
## Test for Heterogeneity:
## Q(df = 7) = 11.8743, p-val = 0.1048
##
## Model Results:
```

```
##
## estimate       se     zval    pval     ci.lb    ci.ub
##   -0.6944  13.2842  -0.0523  0.9583  -26.7309  25.3422
##
## ---
## Signif. codes:  0 '***' 0.001 '**' 0.01 '*' 0.05 '.' 0.1 ' ' 1
```

```
forest(mod_edu_con_COT1,main="Forest plot",header='Study',xlab='')
mtext(c("Favors medium/high education", "Favors low education"),
      side=1, line=3, at=c(-20,20),adj=c(1,0))
arrows(x0=c(-20,20), y0=-4.4, x1=c(-200, 200), y1=-4.4, length=0.1, lwd=2, xpd=TRUE)
```

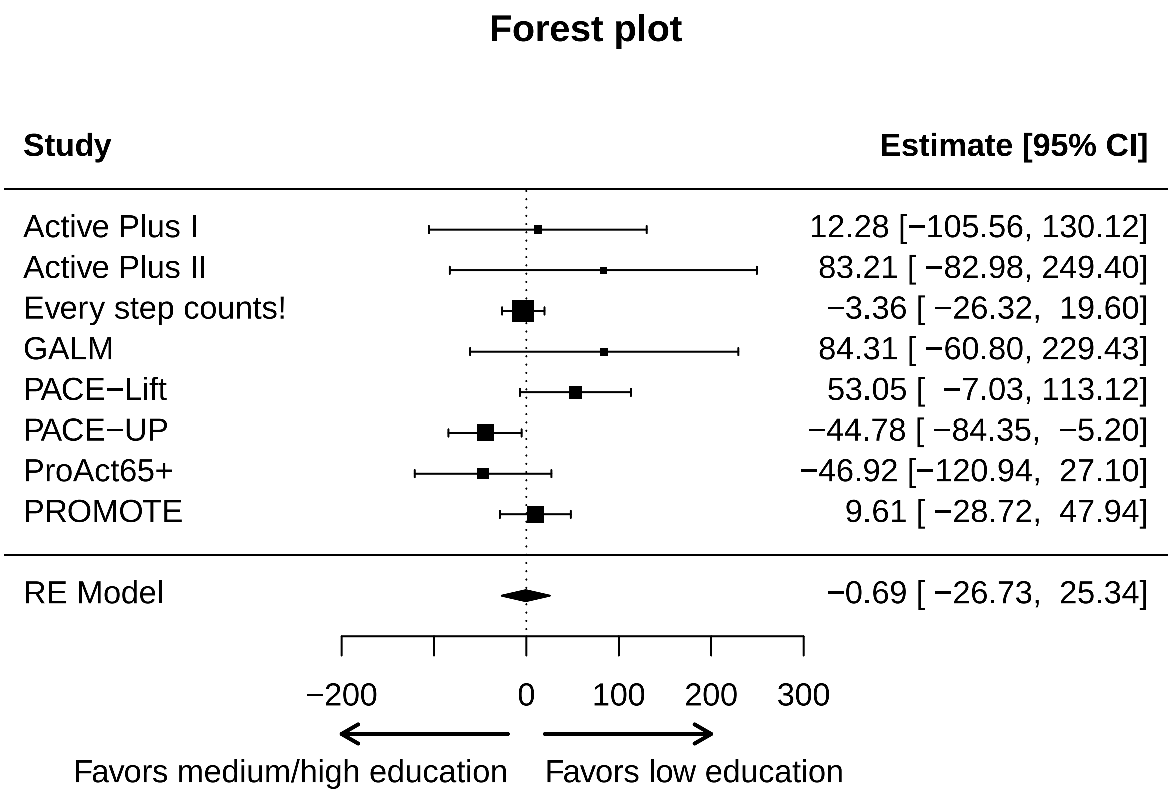


```
funnel(mod_edu_con_COT1,legend=TRUE,main='Funnel plot')
```

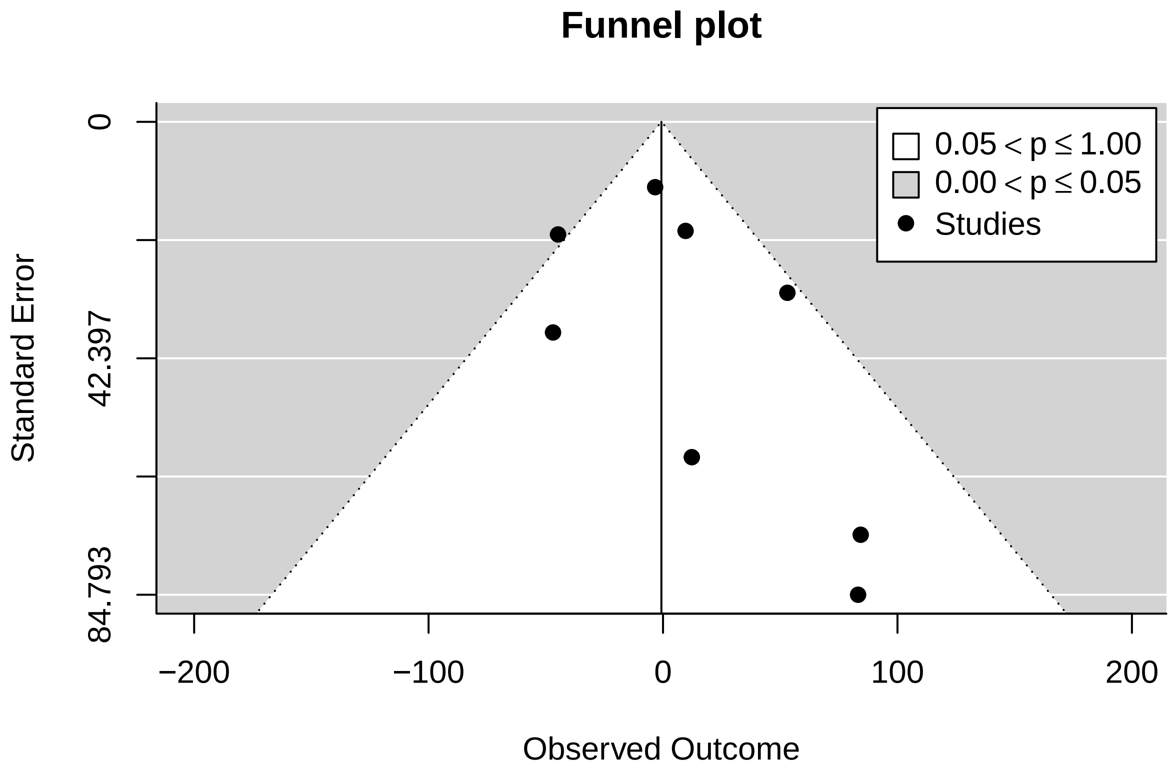

Model C2T1 (data from 8 studies; 7 randomized, 1 non-randomized; 3 with objective physical activity measure, 5 with subjective physical activity measure): The moderated effect of the intervention through education (reference: medium/high) at T1, adjusted for physical activity level at T0, age, gender, and the condition x gender interaction

```
mod_edu_con_C2T1 = rma(yi=data_EQUAL_meta$X_EDU_CON_C2T1,
                       sei=data_EQUAL_meta$X_EDU_CON_C2T1/data_EQUAL_meta$t_X_EDU_CON_C2T1,
                       method='DL',slab=Study)
summary(mod_edu_con_C2T1)
```

```
##
## Random-Effects Model (k = 8; tau^2 estimator: DL)
##
##   logLik  deviance       AIC       BIC      AICc
## -41.8717   12.2916   87.7433   87.9022   90.1433
##
## tau^2 (estimated amount of total heterogeneity): 579.8033 (SE = 759.2898)
## tau (square root of estimated tau^2 value):      24.0791
## I^2 (total heterogeneity / total variability):   45.40%
## H^2 (total variability / sampling variability):  1.83
##
## Test for Heterogeneity:
## Q(df = 7) = 12.8201, p-val = 0.0766
##
## Model Results:
##
## estimate       se     zval     pval     ci.lb    ci.ub
```

```
##  -1.5024  13.9724  -0.1075  0.9144  -28.8878  25.8831
##
## ---
## Signif. codes:  0 '***' 0.001 '**' 0.01 '*' 0.05 '.' 0.1 ' ' 1
```

```r
forest(mod_edu_con_C2T1,main="Forest plot",header='Study',xlab='')
mtext(c("Favors medium/high education", "Favors low education"),
      side=1, line=3, at=c(-20,20),adj=c(1,0))
arrows(x0=c(-20,20), y0=-4.4, x1=c(-200, 200), y1=-4.4, length=0.1, lwd=2, xpd=TRUE)
```

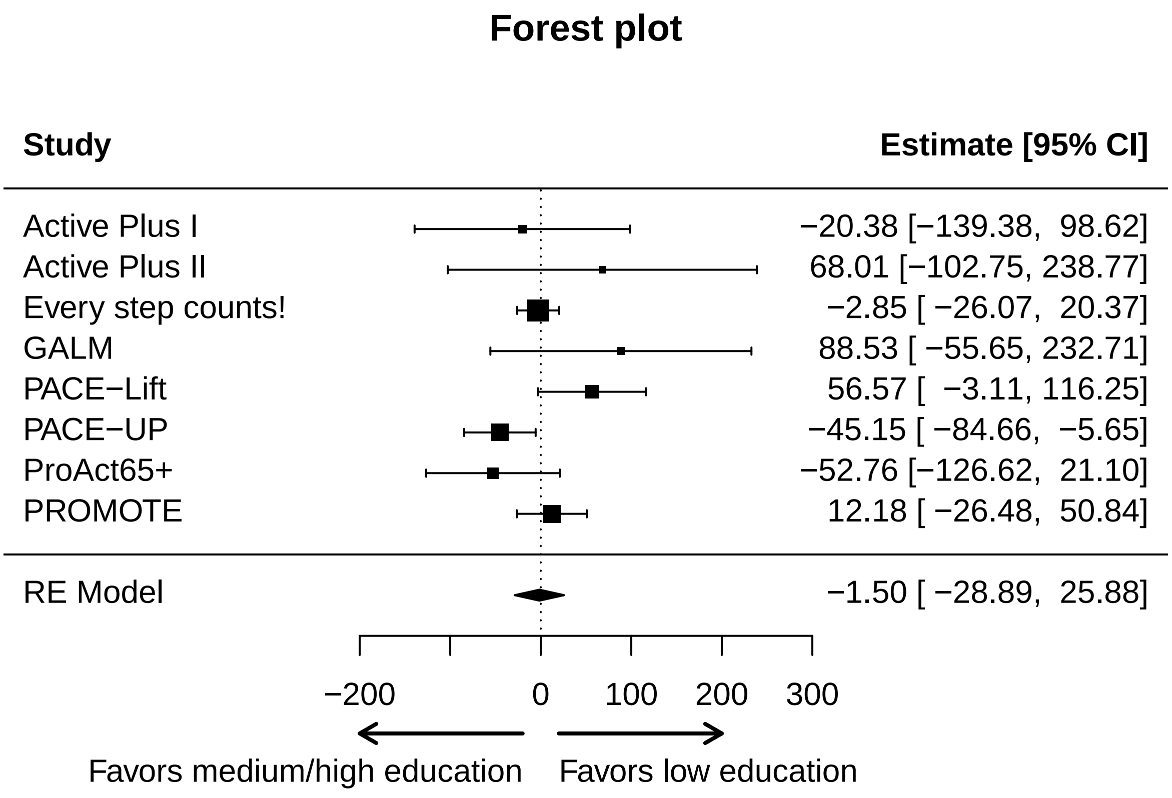


```r
funnel(mod_edu_con_C2T1,legend=TRUE,main='Funnel plot')
```

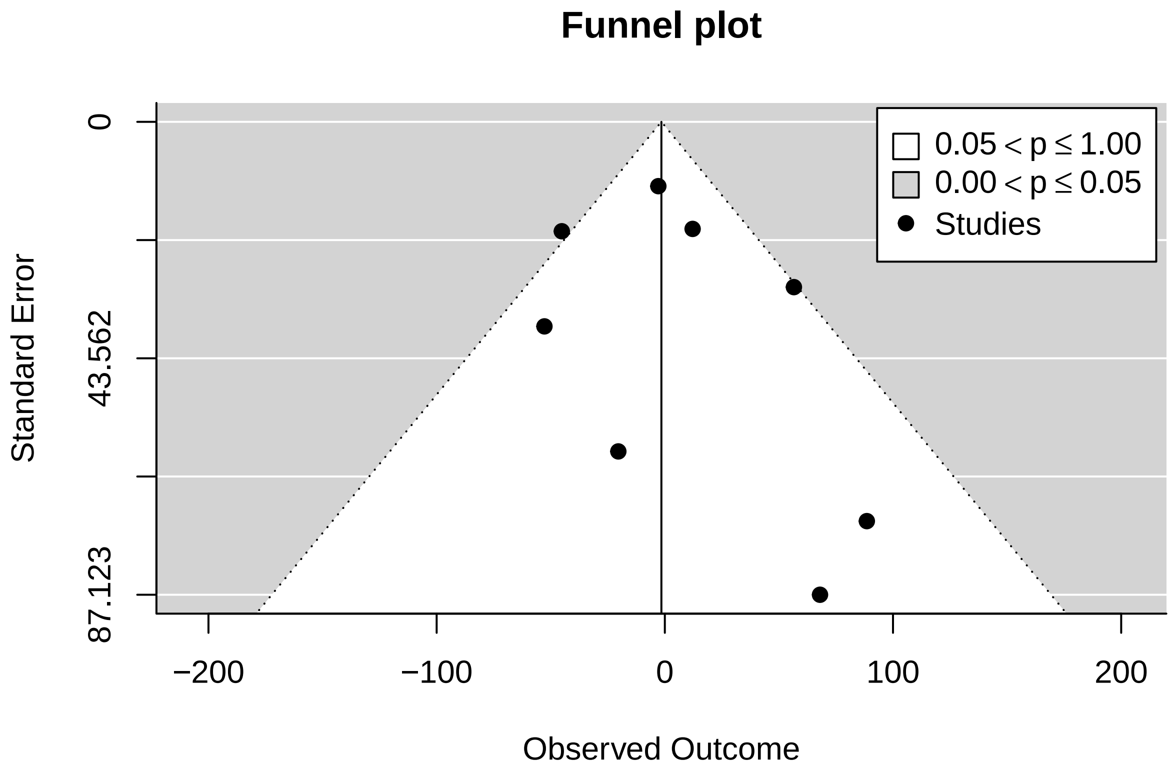

Model C0T2 (data from 5 studies; 5 randomized, 0 non-randomized; 2 with objective physical activity measure, 3 with subjective physical activity measure): The moderated effect of the intervention through education (reference: medium/high) at T2, adjusted for physical activity level at T0 and age

```
mod_edu_con_C0T2 = rma(yi=data_EQUAL_meta$X_EDU_CON_C0T2,
                       sei=data_EQUAL_meta$X_EDU_CON_C0T2/data_EQUAL_meta$t_X_EDU_CON_C0T2,
                       method='DL',slab=Study)
```

```
## Warning in rma(yi = data_EQUAL_meta$X_EDU_CON_C0T2, sei =
## data_EQUAL_meta$X_EDU_CON_C0T2/data_EQUAL_meta$t_X_EDU_CON_C0T2, : Studies with
## NAs omitted from model fitting.
```

```
summary(mod_edu_con_C0T2)
```

```
##
## Random-Effects Model (k = 5; tau^2 estimator: DL)
##
##   logLik  deviance       AIC       BIC      AICc
## -26.5228    7.0817   57.0455   56.2644   63.0455
##
## tau^2 (estimated amount of total heterogeneity): 716.6227 (SE = 1464.5754)
## tau (square root of estimated tau^2 value):      26.7698
## I^2 (total heterogeneity / total variability):   35.80%
## H^2 (total variability / sampling variability):  1.56
##
## Test for Heterogeneity:
## Q(df = 4) = 6.2305, p-val = 0.1826
##
```

```
## Model Results:
##
## estimate       se     zval    pval     ci.lb    ci.ub
## -11.6589  20.4349  -0.5705  0.5683  -51.7106  28.3927
##
## ---
## Signif. codes:  0 '***' 0.001 '**' 0.01 '*' 0.05 '.' 0.1 ' ' 1
```

```r
forest(mod_edu_con_COT2,main="Forest plot",header='Study',xlab='')
mtext(c("Favors medium/high education", "Favors low education"),
      side=1, line=3, at=c(-20,20),adj=c(1,0))
arrows(x0=c(-20,20), y0=-3.8, x1=c(-200, 200), y1=-3.8, length=0.1, lwd=2, xpd=TRUE)
```

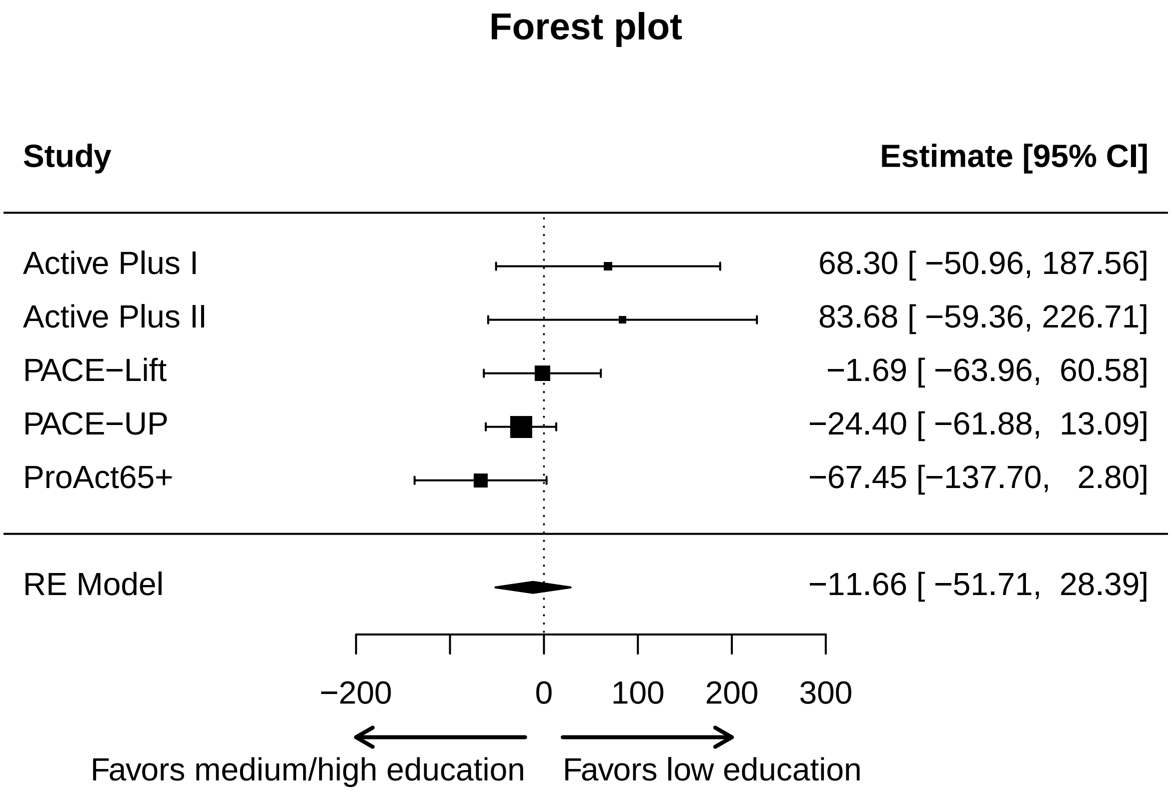


```r
funnel(mod_edu_con_COT2,legend=TRUE,main='Funnel plot')
```

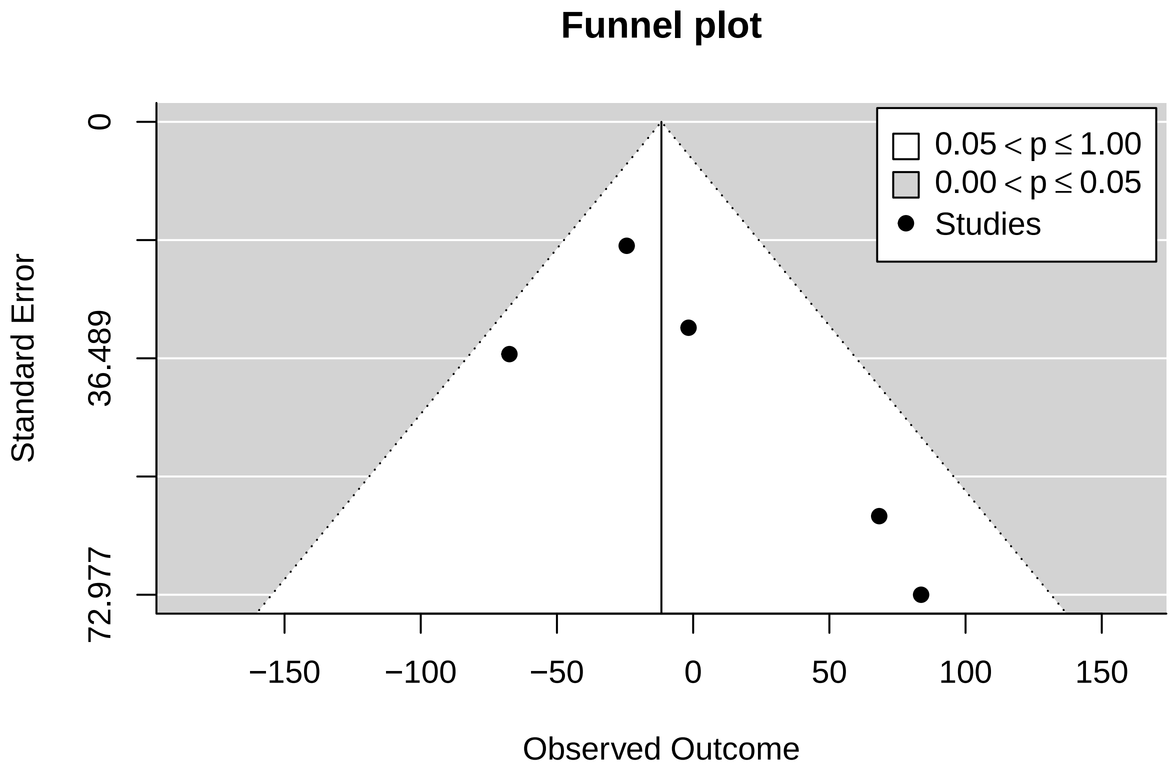

Model C2T2 (data from 5 studies; 5 randomized, 0 non-randomized; 2 with objective physical activity measure, 3 with subjective physical activity measure): The moderated effect of the intervention through education (reference: medium/high) at T2, adjusted for physical activity level at T0, age, gender, and the condition x gender interaction

```
mod_edu_con_C2T2 = rma(yi=data_EQUAL_meta$X_EDU_CON_C2T2,
                       sei=data_EQUAL_meta$X_EDU_CON_C2T2/data_EQUAL_meta$t_X_EDU_CON_C2T2,
                       method='DL',slab=Study)
```

```
## Warning in rma(yi = data_EQUAL_meta$X_EDU_CON_C2T2, sei =
## data_EQUAL_meta$X_EDU_CON_C2T2/data_EQUAL_meta$t_X_EDU_CON_C2T2, : Studies with
## NAs omitted from model fitting.
```

```
summary(mod_edu_con_C2T2)
```

```
##
## Random-Effects Model (k = 5; tau^2 estimator: DL)
##
##   logLik  deviance       AIC       BIC      AICc
## -26.6917    7.3435   57.3834   56.6022   63.3834
##
## tau^2 (estimated amount of total heterogeneity): 776.5484 (SE = 1520.0142)
## tau (square root of estimated tau^2 value):      27.8666
## I^2 (total heterogeneity / total variability):   37.53%
## H^2 (total variability / sampling variability):  1.60
##
## Test for Heterogeneity:
## Q(df = 4) = 6.4035, p-val = 0.1710
```

```
##
## Model Results:
##
## estimate       se     zval    pval     ci.lb    ci.ub
## -13.3837  20.8663  -0.6414  0.5213  -54.2809  27.5135
##
## ---
## Signif. codes:  0 '***' 0.001 '**' 0.01 '*' 0.05 '.' 0.1 ' ' 1
```

```r
forest(mod_edu_con_C2T2,main="Forest plot",header='Study',xlab='')
mtext(c("Favors medium/high education", "Favors low education"),
      side=1, line=3, at=c(-20,20),adj=c(1,0))
arrows(x0=c(-20,20), y0=-3.8, x1=c(-200, 200), y1=-3.8, length=0.1, lwd=2, xpd=TRUE)
```

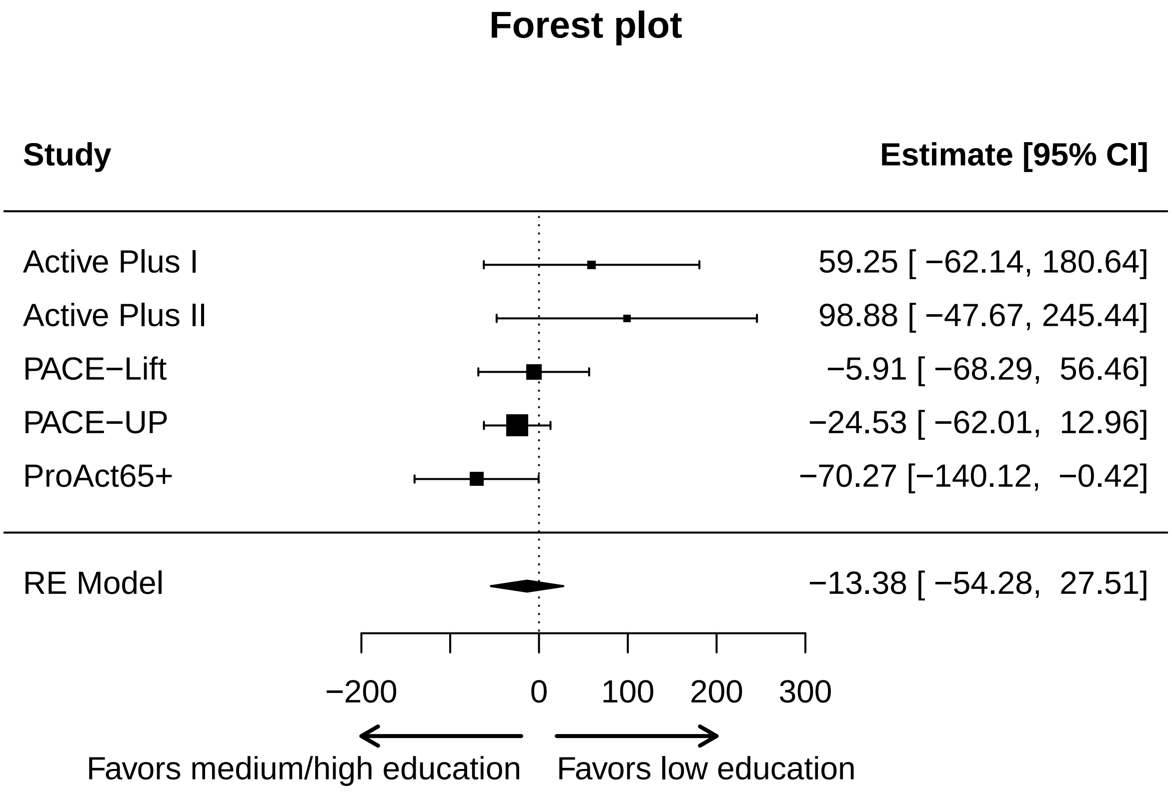


```r
funnel(mod_edu_con_C2T2,legend=TRUE,main='Funnel plot')
```

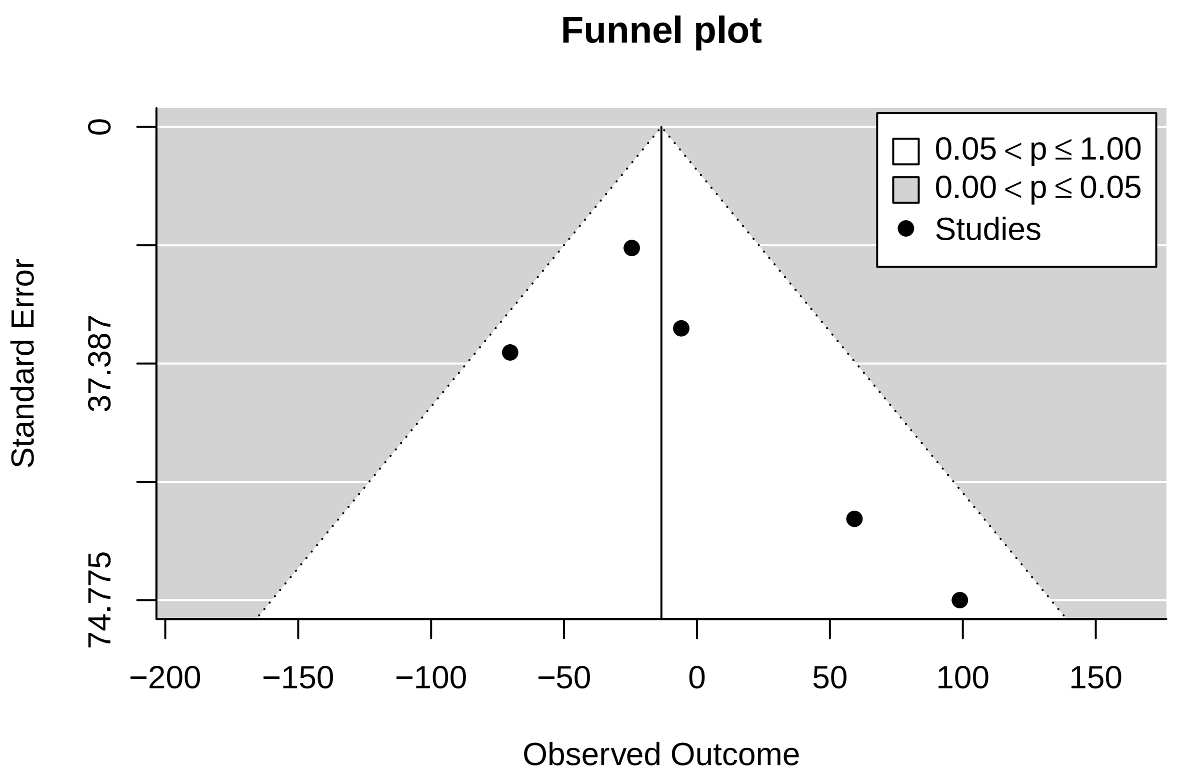

Marital status-specific intervention effects

Model D0T1 (data from 8 studies; 7 randomized, 1 non-randomized; 3 with objective physical activity measure, 5 with subjective physical activity measure): The moderated effect of the intervention through marital status (reference: with partner) at T1, adjusted for physical activity level at T0 and age

```
mod_mar_con_D0T1 = rma(yi=data_EQUAL_meta$X_MAR_CON_D0T1,
                       sei=data_EQUAL_meta$X_MAR_CON_D0T1/data_EQUAL_meta$t_X_MAR_CON_D0T1,
                       method='DL',slab=Study)
summary(mod_mar_con_D0T1)
```

```
##
## Random-Effects Model (k = 8; tau^2 estimator: DL)
##
##   logLik  deviance       AIC       BIC      AICc
## -37.8487    4.8342   79.6973   79.8562   82.0973
##
## tau^2 (estimated amount of total heterogeneity): 0 (SE = 216.7856)
## tau (square root of estimated tau^2 value):      0
## I^2 (total heterogeneity / total variability):   0.00%
## H^2 (total variability / sampling variability):  1.00
##
## Test for Heterogeneity:
## Q(df = 7) = 4.8342, p-val = 0.6802
##
## Model Results:
```

```
##
## estimate      se    zval    pval    ci.lb    ci.ub
##   5.5399  4.9296  1.1238  0.2611  -4.1220  15.2018
##
## ---
## Signif. codes:  0 '***' 0.001 '**' 0.01 '*' 0.05 '.' 0.1 ' ' 1
```

```
forest(mod_mar_con_DOT1,main="Forest plot",header='Study',xlab='')
mtext(c("Favors individuals with partner", "Favors individuals without partner"),
      side=1, line=3, at=c(-20,20),adj=c(1,0))
arrows(x0=c(-20,20), y0=-4.4, x1=c(-200, 200), y1=-4.4, length=0.1, lwd=2, xpd=TRUE)
```

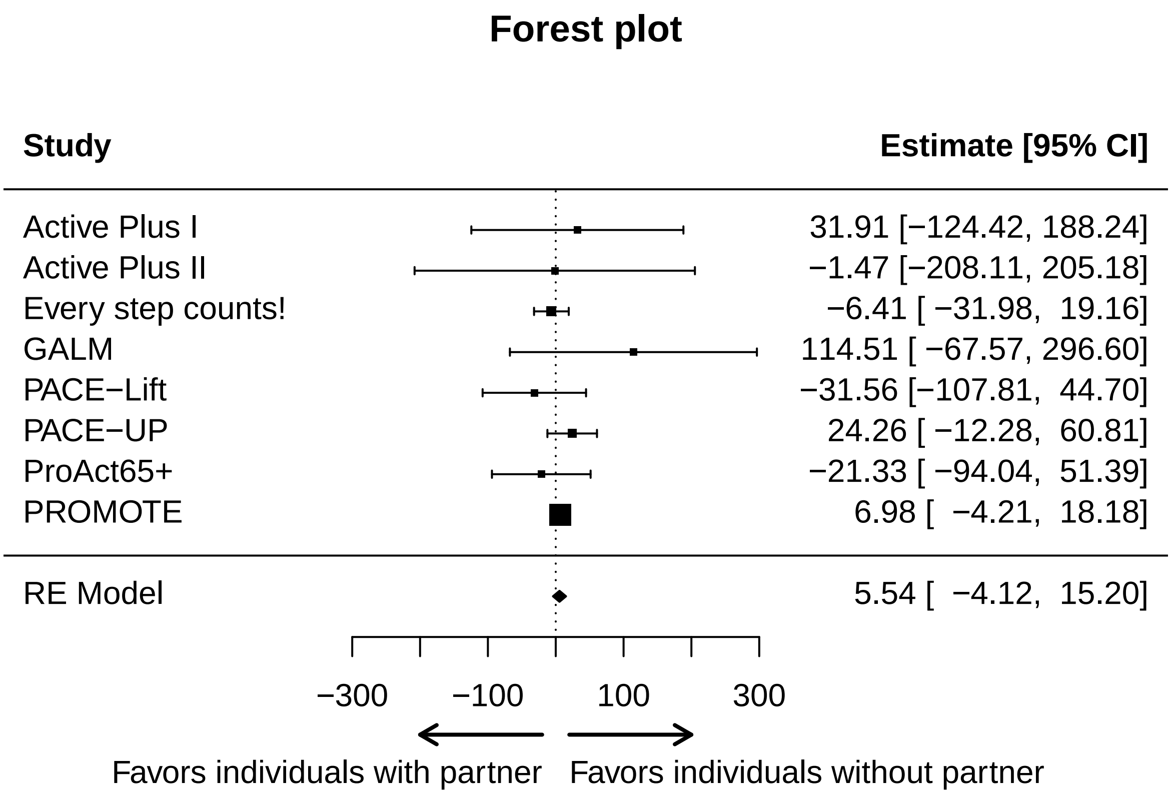


```
funnel(mod_mar_con_DOT1,legend=TRUE,main='Funnel plot')
```

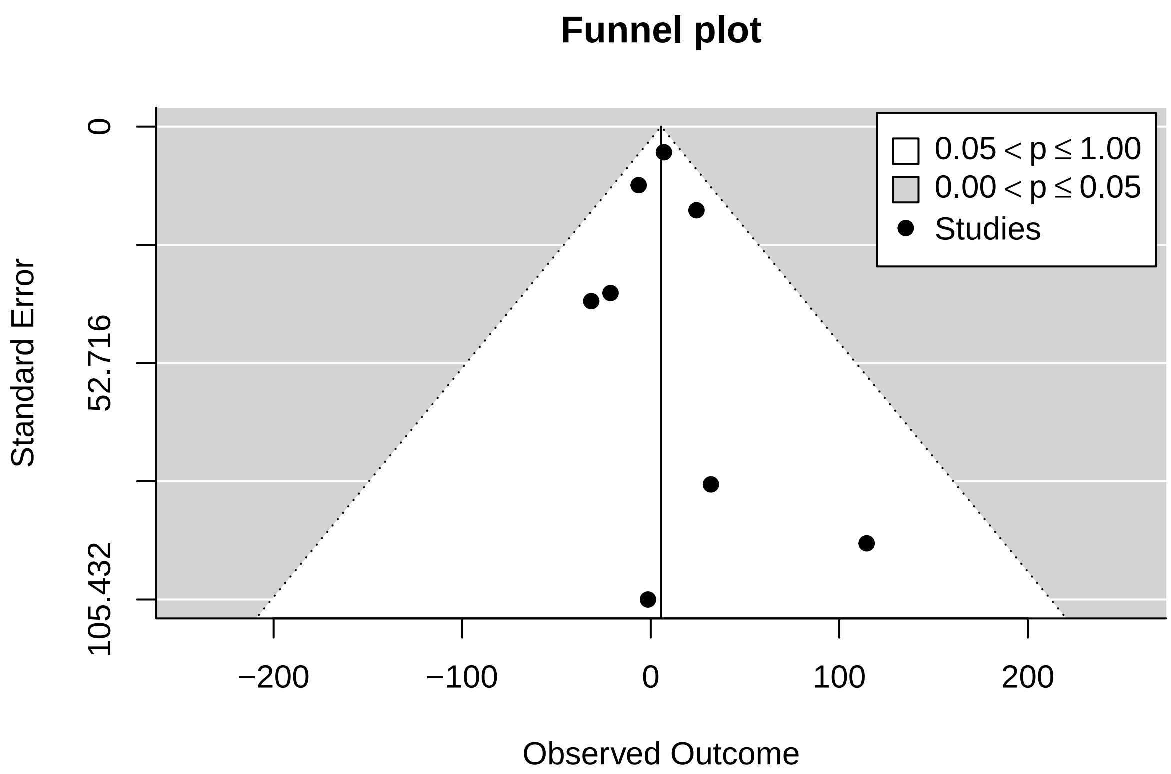

Model D2T1 (data from 8 studies; 7 randomized, 1 non-randomized; 3 with objective physical activity measure, 5 with subjective physical activity measure): The moderated effect of the intervention through marital status (reference: with partner) at T1, adjusted for physical activity level at T0, age, gender, and the condition x gender interaction

```
mod_mar_con_D2T1 = rma(yi=data_EQUAL_meta$X_MAR_CON_D2T1,
                       sei=data_EQUAL_meta$X_MAR_CON_D2T1/data_EQUAL_meta$t_X_MAR_CON_D2T1,
                       method='DL',slab=Study)
summary(mod_mar_con_D2T1)
```

```
##
## Random-Effects Model (k = 8; tau^2 estimator: DL)
##
##   logLik  deviance       AIC       BIC      AICc
## -38.9165    6.6169   81.8331   81.9919   84.2331
##
## tau^2 (estimated amount of total heterogeneity): 0 (SE = 234.9657)
## tau (square root of estimated tau^2 value):      0
## I^2 (total heterogeneity / total variability):   0.00%
## H^2 (total variability / sampling variability):  1.00
##
## Test for Heterogeneity:
## Q(df = 7) = 6.6169, p-val = 0.4698
##
## Model Results:
##
## estimate      se     zval     pval     ci.lb     ci.ub
```

```
##   6.8709  5.1968  1.3221  0.1861  -3.3147  17.0565
##
## ---
## Signif. codes:  0 '***' 0.001 '**' 0.01 '*' 0.05 '.' 0.1 ' ' 1
```

```r
forest(mod_mar_con_D2T1,main="Forest plot",header='Study',xlab='')
mtext(c("Favors individuals with partner", "Favors individuals without partner"),
      side=1, line=3, at=c(-20,20),adj=c(1,0))
arrows(x0=c(-20,20), y0=-4.4, x1=c(-200, 200), y1=-4.4, length=0.1, lwd=2, xpd=TRUE)
```

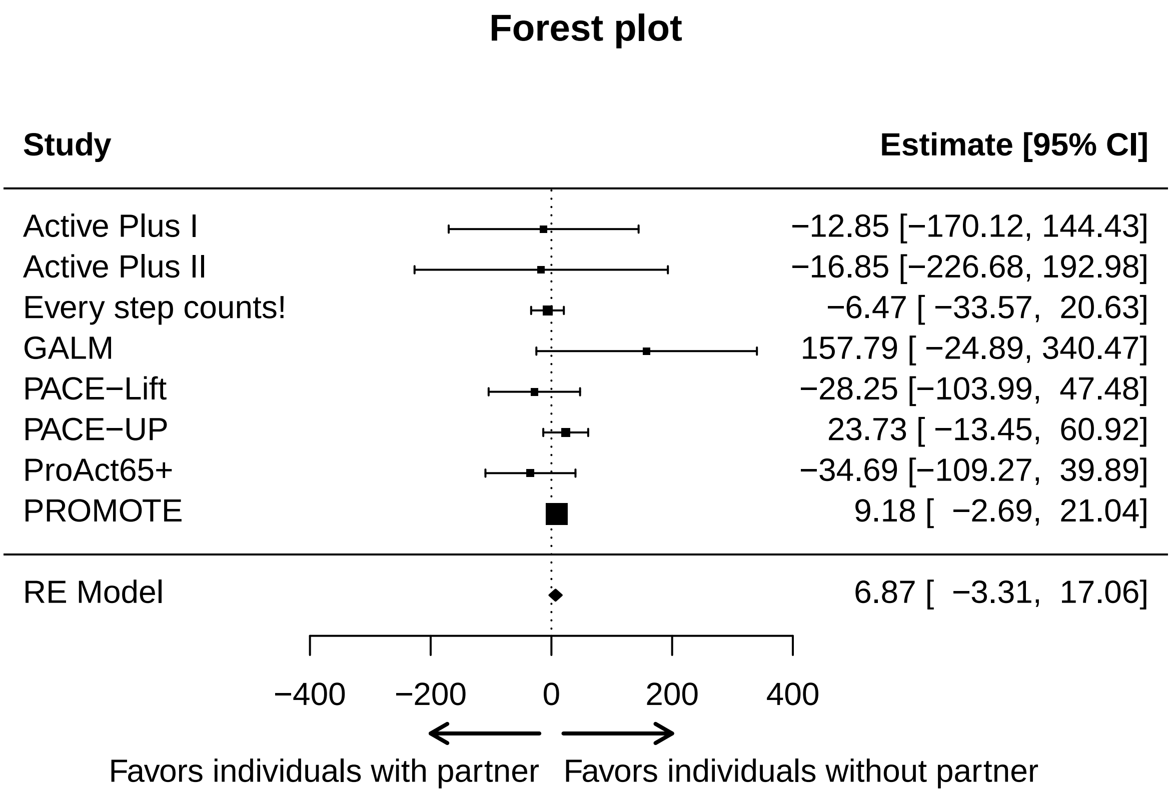


```r
funnel(mod_mar_con_D2T1,legend=TRUE,main='Funnel plot')
```

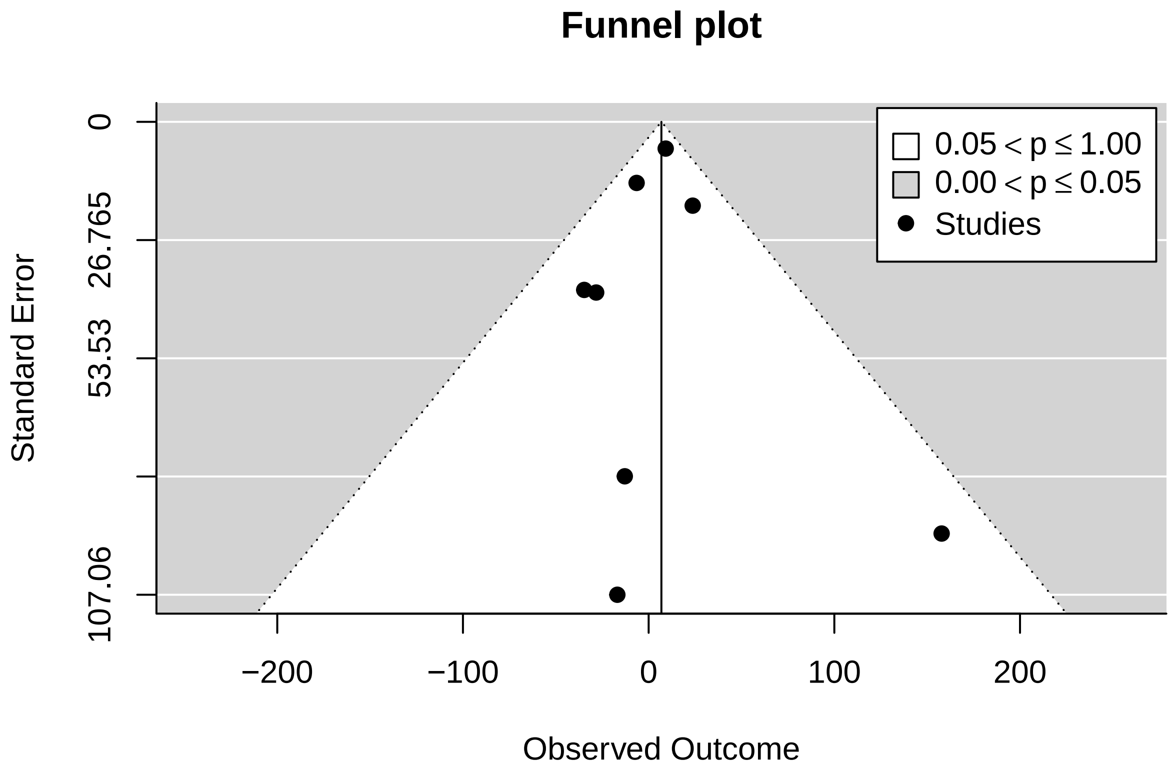

Model D0T2 (data from 5 studies; 5 randomized, 0 non-randomized; 2 with objective physical activity measure, 3 with subjective physical activity measure): The moderated effect of the intervention through marital status (reference: with partner) at T2, adjusted for physical activity level at T0 and age

```
mod_mar_con_D0T2 = rma(yi=data_EQUAL_meta$X_MAR_CON_D0T2,
                       sei=data_EQUAL_meta$X_MAR_CON_D0T2/data_EQUAL_meta$t_X_MAR_CON_D0T2,
                       method='DL',slab=Study)
```

```
## Warning in rma(yi = data_EQUAL_meta$X_MAR_CON_D0T2, sei =
## data_EQUAL_meta$X_MAR_CON_D0T2/data_EQUAL_meta$t_X_MAR_CON_D0T2, : Studies with
## NAs omitted from model fitting.
```

```
summary(mod_mar_con_D0T2)
```

```
##
## Random-Effects Model (k = 5; tau^2 estimator: DL)
##
##   logLik  deviance       AIC       BIC      AICc
## -25.4594    3.7005   54.9188   54.1377   60.9188
##
## tau^2 (estimated amount of total heterogeneity): 0 (SE = 1075.4791)
## tau (square root of estimated tau^2 value):      0
## I^2 (total heterogeneity / total variability):   0.00%
## H^2 (total variability / sampling variability):  1.00
##
## Test for Heterogeneity:
## Q(df = 4) = 3.7005, p-val = 0.4481
##
```

```
## Model Results:
##
## estimate       se    zval    pval     ci.lb    ci.ub
##   7.9573  14.1603  0.5619  0.5742  -19.7964  35.7111
##
## ---
## Signif. codes:  0 '***' 0.001 '**' 0.01 '*' 0.05 '.' 0.1 ' ' 1
```

```r
forest(mod_mar_con_DOT2,main="Forest plot",header='Study',xlab='')
mtext(c("Favors individuals with partner", "Favors individuals without partner"),
      side=1, line=3, at=c(-20,20),adj=c(1,0))
arrows(x0=c(-20,20), y0=-3.8, x1=c(-200, 200), y1=-3.8, length=0.1, lwd=2, xpd=TRUE)
```

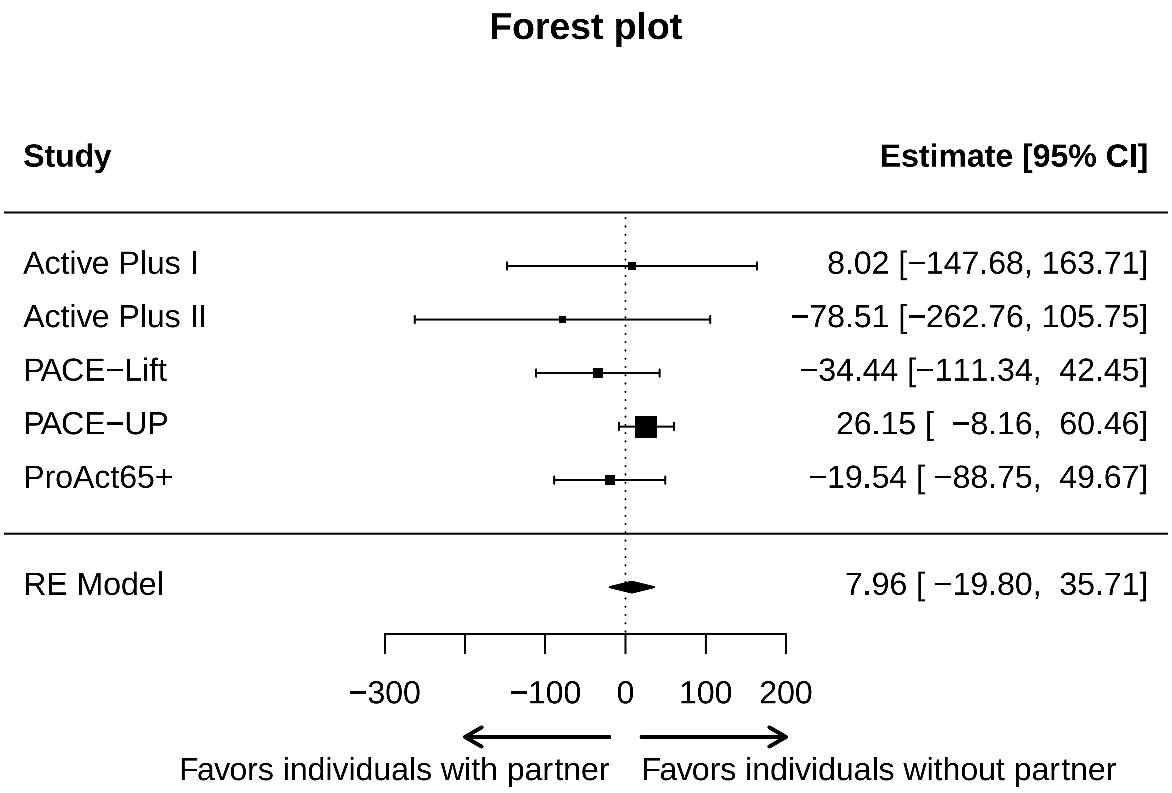


```r
funnel(mod_mar_con_DOT2,legend=TRUE,main='Funnel plot')
```

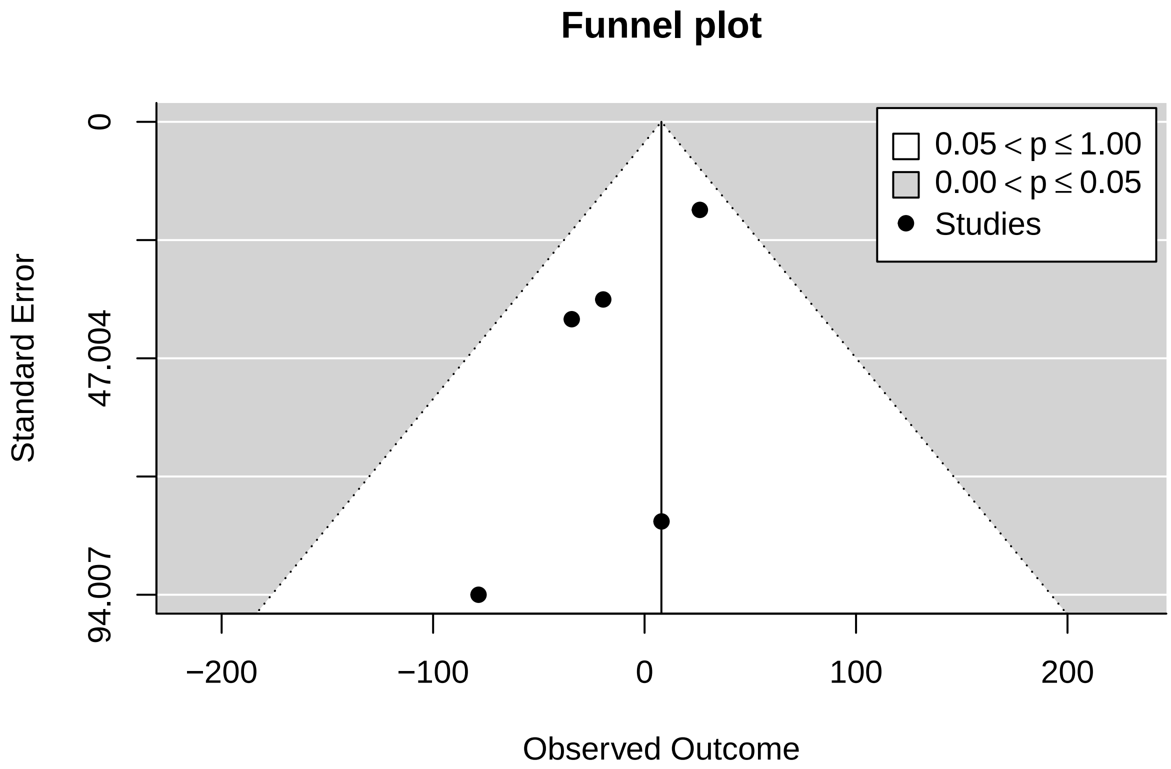

Model D2T2 (data from 5 studies; 5 randomized, 0 non-randomized; 2 with objective physical activity measure, 3 with subjective physical activity measure): The moderated effect of the intervention through marital status (reference: with partner) at T2, adjusted for physical activity level at T0, age, gender, and the condition x gender interaction

```
mod_mar_con_D2T2 = rma(yi=data_EQUAL_meta$X_MAR_CON_D2T2,
                       sei=data_EQUAL_meta$X_MAR_CON_D2T2/data_EQUAL_meta$t_X_MAR_CON_D2T2,
                       method='DL',slab=Study)
```

```
## Warning in rma(yi = data_EQUAL_meta$X_MAR_CON_D2T2, sei =
## data_EQUAL_meta$X_MAR_CON_D2T2/data_EQUAL_meta$t_X_MAR_CON_D2T2, : Studies with
## NAs omitted from model fitting.
```

```
summary(mod_mar_con_D2T2)
```

```
##
## Random-Effects Model (k = 5; tau^2 estimator: DL)
##
##   logLik  deviance       AIC       BIC      AICc
## -25.9086    4.4550   55.8172   55.0361   61.8172
##
## tau^2 (estimated amount of total heterogeneity): 280.1181 (SE = 1314.5422)
## tau (square root of estimated tau^2 value):      16.7367
## I^2 (total heterogeneity / total variability):   15.20%
## H^2 (total variability / sampling variability):  1.18
##
## Test for Heterogeneity:
## Q(df = 4) = 4.7170, p-val = 0.3176
```

```
##
## Model Results:
##
## estimate       se     zval    pval     ci.lb    ci.ub
##  -1.6785  17.9354  -0.0936  0.9254  -36.8312  33.4742
##
## ---
## Signif. codes:  0 '***' 0.001 '**' 0.01 '*' 0.05 '.' 0.1 ' ' 1
```

```
forest(mod_mar_con_D2T2,main="Forest plot",header='Study',xlab='')
mtext(c("Favors individuals with partner", "Favors individuals without partner"),
      side=1, line=3, at=c(-20,20),adj=c(1,0))
arrows(x0=c(-20,20), y0=-3.8, x1=c(-200, 200), y1=-3.8, length=0.1, lwd=2, xpd=TRUE)
```

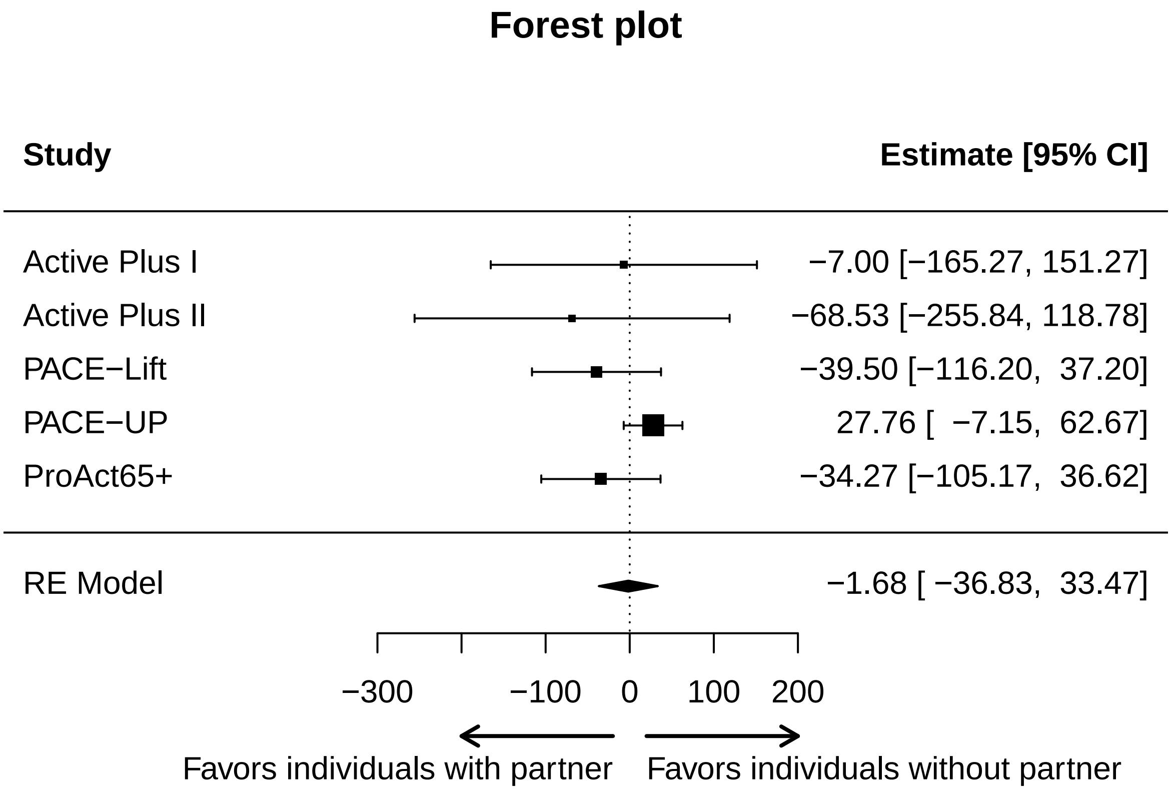


```
funnel(mod_mar_con_D2T2,legend=TRUE,main='Funnel plot')
```

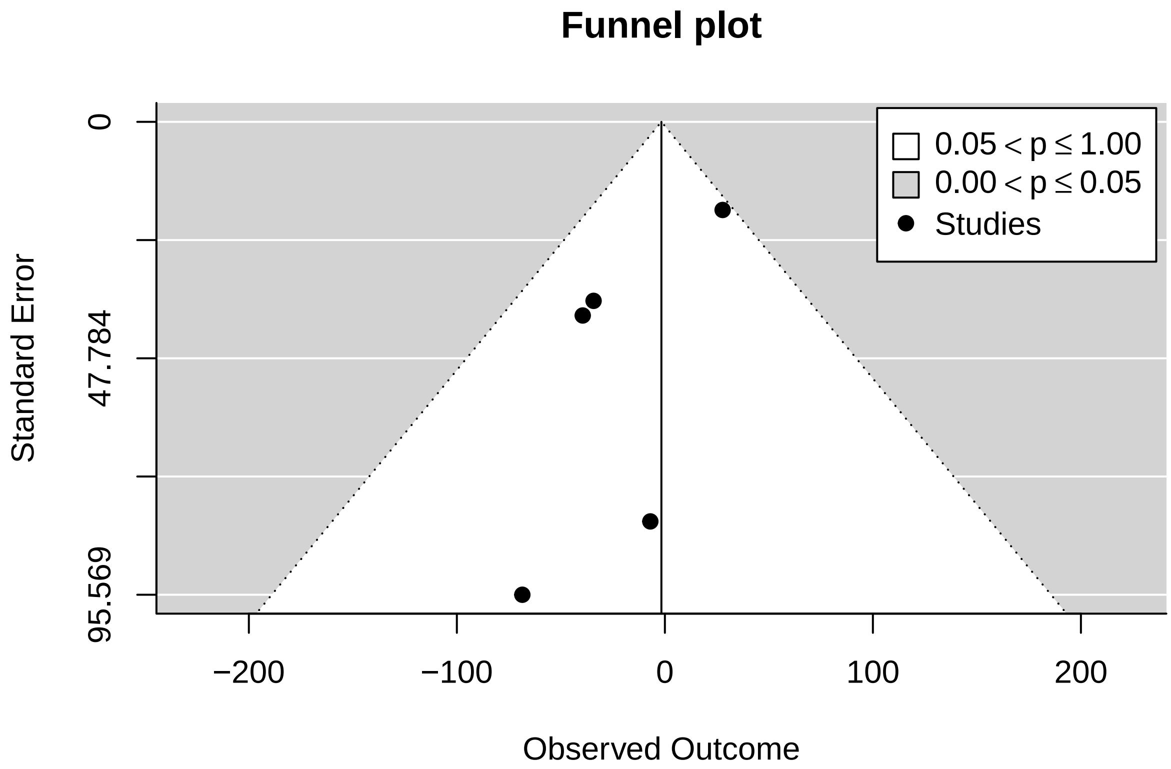

Income-specific intervention effects

Model E0T1 (data from 2 studies; 2 randomized, 0 non-randomized; 0 with objective physical activity measure, 2 with subjective physical activity measure): The moderated effect of the intervention through income (reference: medium/high) at T1, adjusted for physical activity level at T0 and age

```
mod_inc_con_E0T1 = rma(yi=data_EQUAL_meta$X_INC_CON_E0T1,
                       sei=data_EQUAL_meta$X_INC_CON_E0T1/data_EQUAL_meta$t_X_INC_CON_E0T1,
                       method='DL',slab=Study)
```

```
## Warning in rma(yi = data_EQUAL_meta$X_INC_CON_E0T1, sei =
## data_EQUAL_meta$X_INC_CON_E0T1/data_EQUAL_meta$t_X_INC_CON_E0T1, : Studies with
## NAs omitted from model fitting.
```

```
summary(mod_inc_con_E0T1)
```

```
##
## Random-Effects Model (k = 2; tau^2 estimator: DL)
##
##   logLik  deviance       AIC       BIC      AICc
##  -7.2803    0.0048   18.5606   15.9469   30.5606
##
## tau^2 (estimated amount of total heterogeneity): 0 (SE = 1210.2026)
## tau (square root of estimated tau^2 value):      0
## I^2 (total heterogeneity / total variability):   0.00%
## H^2 (total variability / sampling variability):  1.00
##
```

```
## Test for Heterogeneity:
## Q(df = 1) = 0.0048, p-val = 0.9450
##
## Model Results:
##
## estimate      se     zval    pval     ci.lb    ci.ub
##  -0.4257  5.5706  -0.0764  0.9391  -11.3440  10.4925
##
## ---
## Signif. codes:  0 '***' 0.001 '**' 0.01 '*' 0.05 '.' 0.1 ' ' 1
```

```
forest(mod_inc_con_EOT1,main="Forest plot",header='Study',xlab='')
mtext(c("Favors medium/high income", "Favors low income"),
      side=1, line=3, at=c(-10,10),adj=c(1,0))
arrows(x0=c(-10,10), y0=-3, x1=c(-100, 100), y1=-3, length=0.1, lwd=2, xpd=TRUE)
```

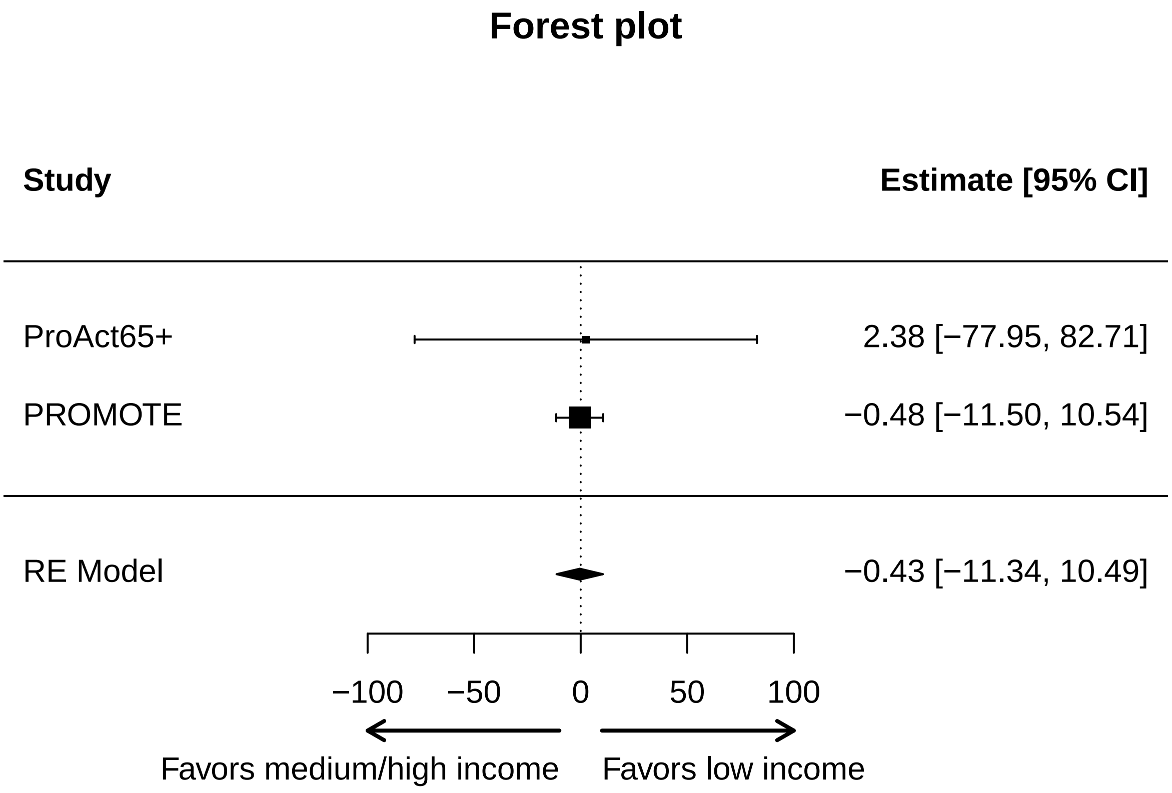


```
funnel(mod_inc_con_EOT1,legend=TRUE,main='Funnel plot')
```

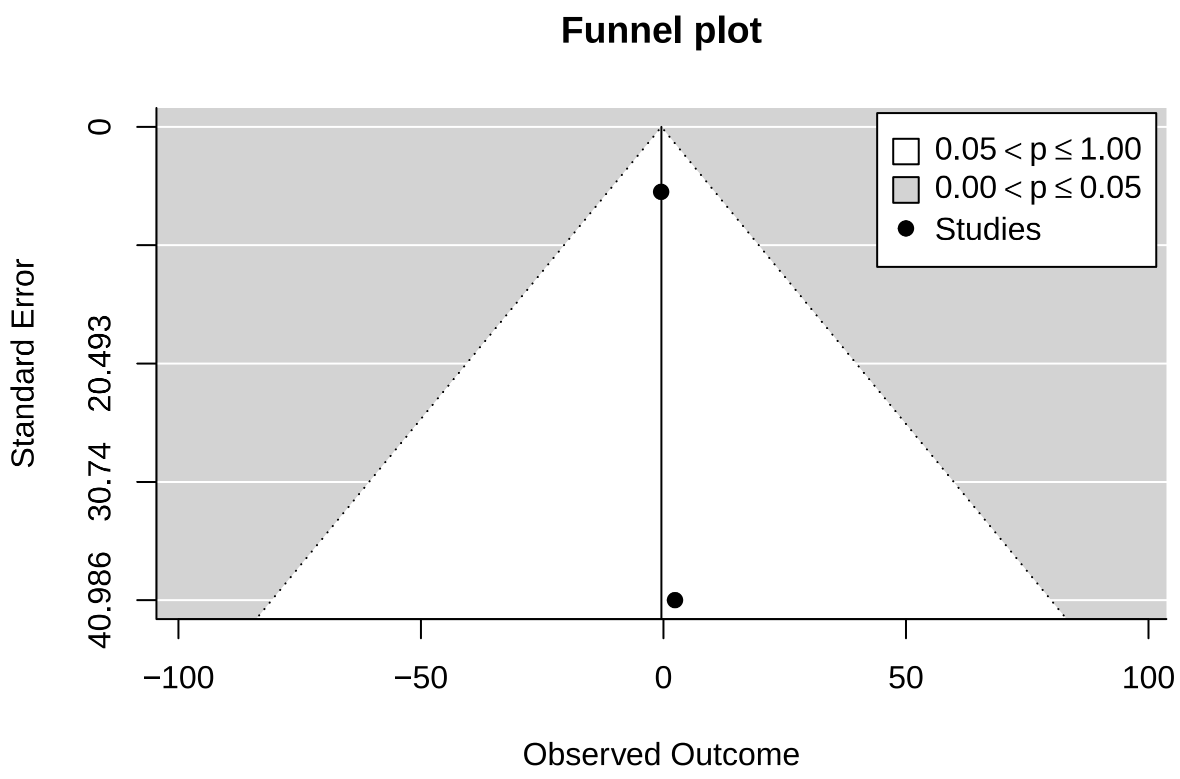

Model E2T1 (data from 2 studies; 2 randomized, 0 non-randomized; 0 with objective physical activity measure, 2 with subjective physical activity measure): The moderated effect of the intervention through income (reference: medium/high) at T1, adjusted for physical activity level at T0, age, gender, and the condition x gender interaction

```
mod_inc_con_E2T1 = rma(yi=data_EQUAL_meta$X_INC_CON_E2T1,
                       sei=data_EQUAL_meta$X_INC_CON_E2T1/data_EQUAL_meta$t_X_INC_CON_E2T1,
                       method='DL',slab=Study)
```

```
## Warning in rma(yi = data_EQUAL_meta$X_INC_CON_E2T1, sei =
## data_EQUAL_meta$X_INC_CON_E2T1/data_EQUAL_meta$t_X_INC_CON_E2T1, : Studies with
## NAs omitted from model fitting.
```

```
summary(mod_inc_con_E2T1)
```

```
##
## Random-Effects Model (k = 2; tau^2 estimator: DL)
##
##   logLik  deviance       AIC       BIC      AICc
##  -7.2955    0.0001   18.5910   15.9773   30.5910
##
## tau^2 (estimated amount of total heterogeneity): 0 (SE = 1214.0954)
## tau (square root of estimated tau^2 value):      0
## I^2 (total heterogeneity / total variability):   0.00%
## H^2 (total variability / sampling variability):  1.00
##
## Test for Heterogeneity:
## Q(df = 1) = 0.0001, p-val = 0.9942
```

```
##
## Model Results:
##
## estimate      se    zval    pval     ci.lb    ci.ub
##   0.5126  5.6602  0.0906  0.9278  -10.5811  11.6063
##
## ---
## Signif. codes:  0 '***' 0.001 '**' 0.01 '*' 0.05 '.' 0.1 ' ' 1
```

```
forest(mod_inc_con_E2T1,main="Forest plot",header='Study',xlab='')
mtext(c("Favors medium/high income", "Favors low income"),
      side=1, line=3, at=c(-10,10),adj=c(1,0))
arrows(x0=c(-10,10), y0=-3, x1=c(-100, 100), y1=-3, length=0.1, lwd=2, xpd=TRUE)
```

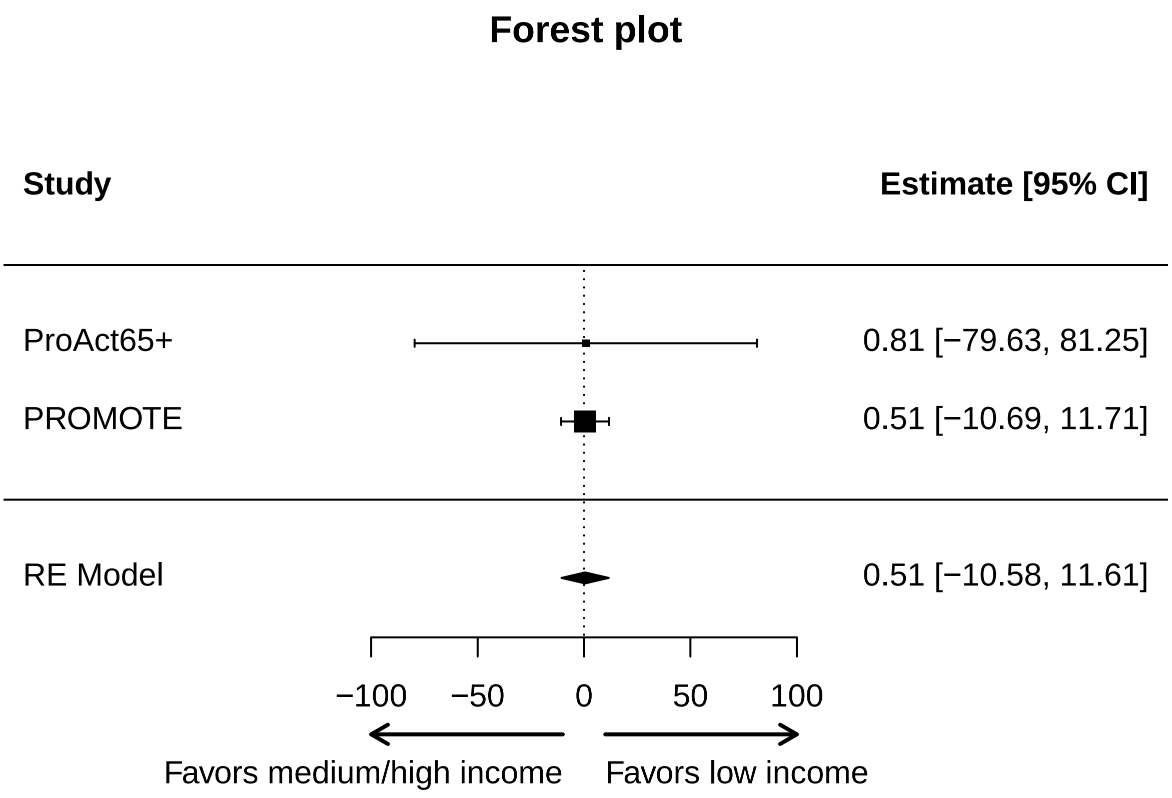


```
funnel(mod_inc_con_E2T1,legend=TRUE,main='Funnel plot')
```

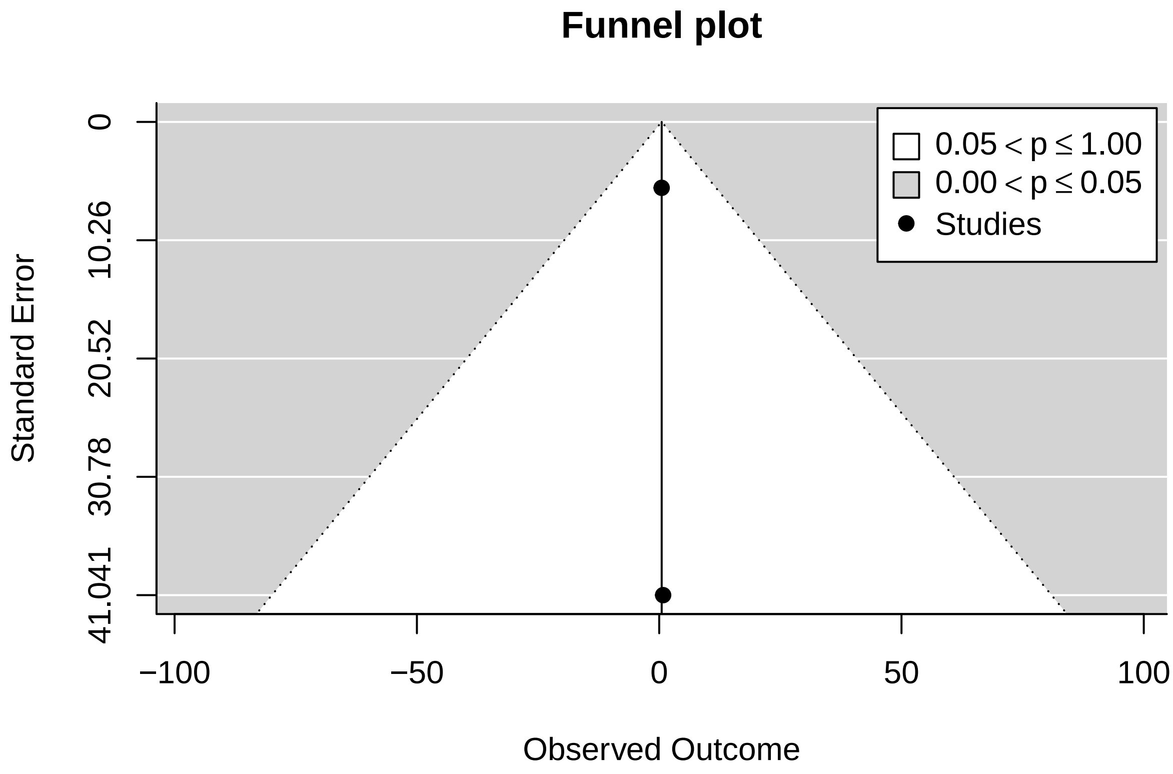

Area deprivation-specific intervention effects

Model F0T1 (data from 3 studies; 3 randomized, 0 non-randomized; 2 with objective physical activity measure, 1 with subjective physical activity measure): The moderated effect of the intervention through area deprivation (reference: medium/low) at T1, adjusted for physical activity level at T0 and age

```
mod_imd_con_F0T1 = rma(yi=data_EQUAL_meta$X_IMD_CON_F0T1,
                       sei=data_EQUAL_meta$X_IMD_CON_F0T1/data_EQUAL_meta$t_X_IMD_CON_F0T1,
                       method='DL',slab=Study)
```

```
## Warning in rma(yi = data_EQUAL_meta$X_IMD_CON_F0T1, sei =
## data_EQUAL_meta$X_IMD_CON_F0T1/data_EQUAL_meta$t_X_IMD_CON_F0T1, : Studies with
## NAs omitted from model fitting.
```

```
summary(mod_imd_con_F0T1)
```

```
##
## Random-Effects Model (k = 3; tau^2 estimator: DL)
##
##   logLik  deviance       AIC       BIC      AICc
## -13.0862    0.0079   30.1724   28.3696   42.1724
##
## tau^2 (estimated amount of total heterogeneity): 0 (SE = 998.3920)
## tau (square root of estimated tau^2 value):      0
## I^2 (total heterogeneity / total variability):   0.00%
## H^2 (total variability / sampling variability):  1.00
##
```

```
## Test for Heterogeneity:
## Q(df = 2) = 0.0079, p-val = 0.9961
##
## Model Results:
##
## estimate       se     zval    pval     ci.lb   ci.ub
## -27.9503  15.6696  -1.7837  0.0745  -58.6621  2.7615  .
##
## ---
## Signif. codes:  0 '***' 0.001 '**' 0.01 '*' 0.05 '.' 0.1 ' ' 1
```

```
forest(mod_imd_con_F0T1,main="Forest plot",header='Study',xlab='')
mtext(c("Favors medium/low area deprivation", "Favors high area deprivation"),
      side=1, line=3, at=c(-10,10),adj=c(1,0))
arrows(x0=c(-10,10), y0=-3.3, x1=c(-100, 100), y1=-3.3, length=0.1, lwd=2, xpd=TRUE)
```

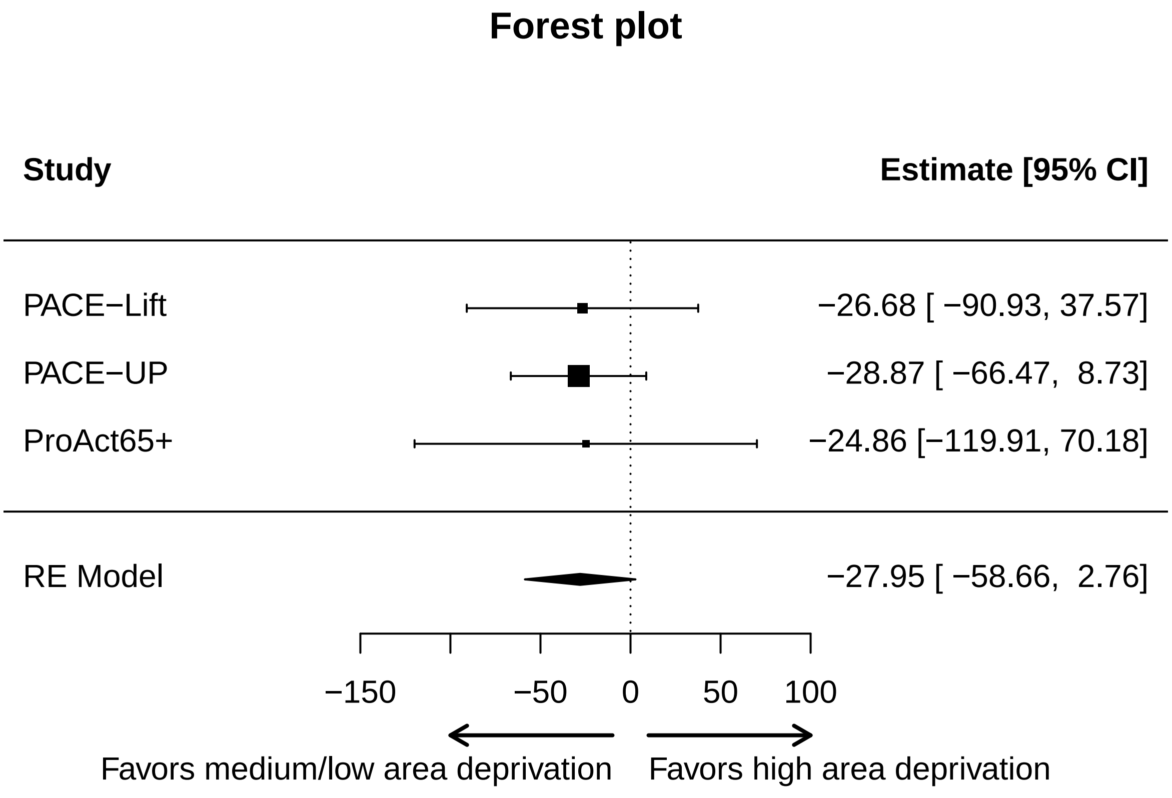


```
funnel(mod_imd_con_F0T1,legend=TRUE,main='Funnel plot')
```

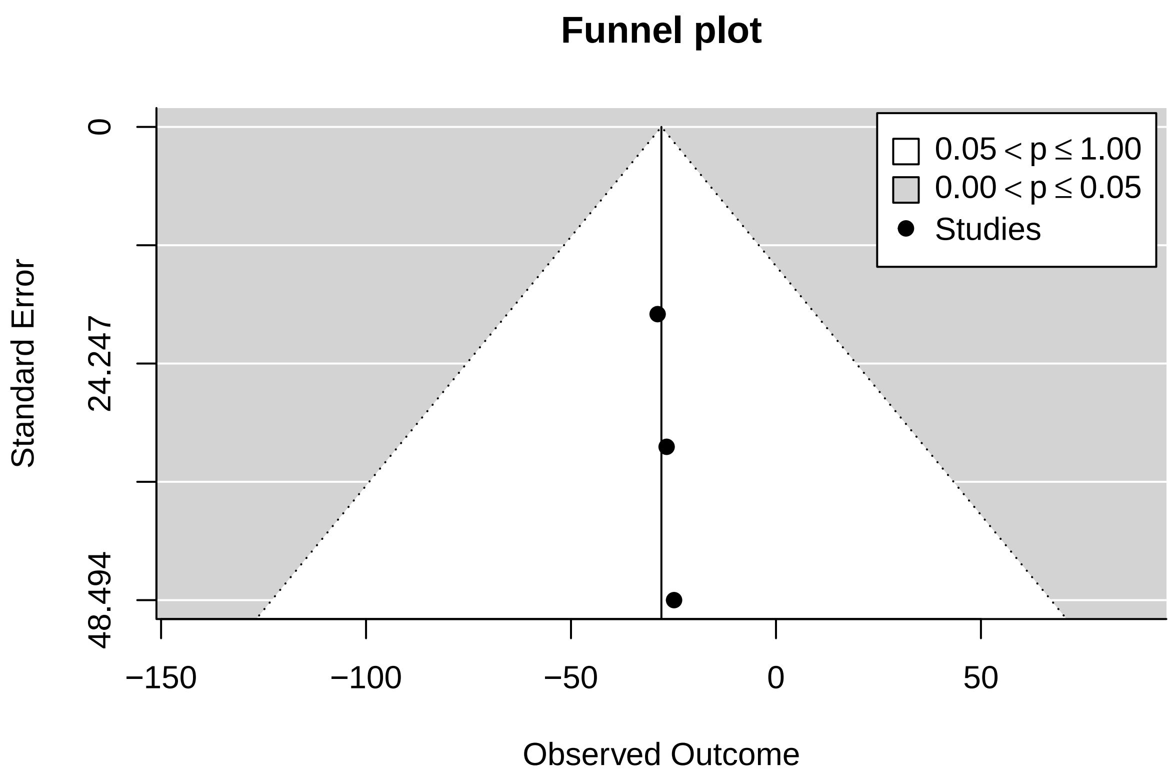

Model F2T1 (data from 3 studies; 3 randomized, 0 non-randomized; 2 with objective physical activity measure, 1 with subjective physical activity measure): The moderated effect of the intervention through area deprivation (reference: medium/low) at T1, adjusted for physical activity level at T0, age, gender, and the condition x gender interaction

```
mod_imd_con_F2T1 = rma(yi=data_EQUAL_meta$X_IMD_CON_F2T1,
                       sei=data_EQUAL_meta$X_IMD_CON_F2T1/data_EQUAL_meta$t_X_IMD_CON_F2T1,
                       method='DL',slab=Study)
```

```
## Warning in rma(yi = data_EQUAL_meta$X_IMD_CON_F2T1, sei =
## data_EQUAL_meta$X_IMD_CON_F2T1/data_EQUAL_meta$t_X_IMD_CON_F2T1, : Studies with
## NAs omitted from model fitting.
```

```
summary(mod_imd_con_F2T1)
```

```
##
## Random-Effects Model (k = 3; tau^2 estimator: DL)
##
##   logLik  deviance       AIC       BIC      AICc
## -13.0729    0.0122   30.1459   28.3431   42.1459
##
## tau^2 (estimated amount of total heterogeneity): 0 (SE = 985.3183)
## tau (square root of estimated tau^2 value):      0
## I^2 (total heterogeneity / total variability):   0.00%
## H^2 (total variability / sampling variability):  1.00
##
## Test for Heterogeneity:
## Q(df = 2) = 0.0122, p-val = 0.9939
```

```
##
## Model Results:
##
## estimate       se     zval    pval     ci.lb   ci.ub
## -27.9203  15.6111  -1.7885  0.0737  -58.5175  2.6769  .
##
## ---
## Signif. codes:  0 '***' 0.001 '**' 0.01 '*' 0.05 '.' 0.1 ' ' 1
```

```
forest(mod_imd_con_F2T1,main="Forest plot",header='Study',xlab='')
mtext(c("Favors medium/low area deprivation", "Favors high area deprivation"),
      side=1, line=3, at=c(-10,10),adj=c(1,0))
arrows(x0=c(-10,10), y0=-3.3, x1=c(-100, 100), y1=-3.3, length=0.1, lwd=2, xpd=TRUE)
```

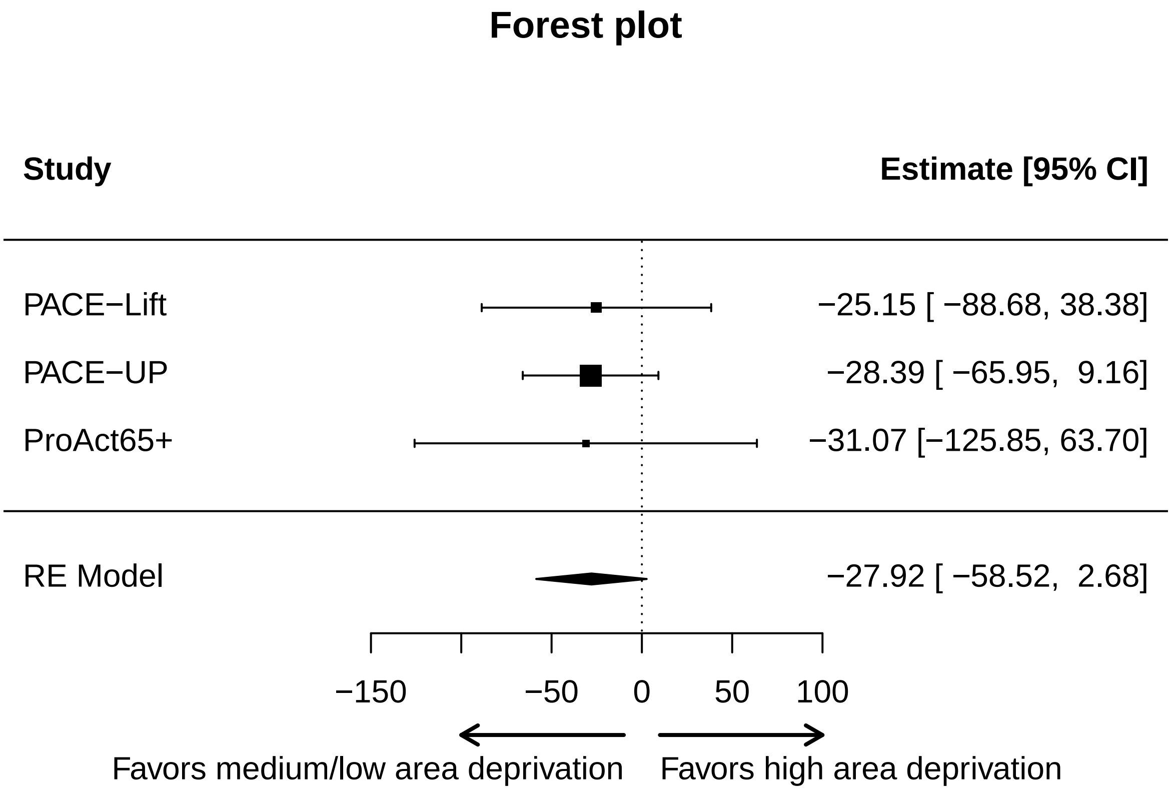


```
funnel(mod_imd_con_F2T1,legend=TRUE,main='Funnel plot')
```

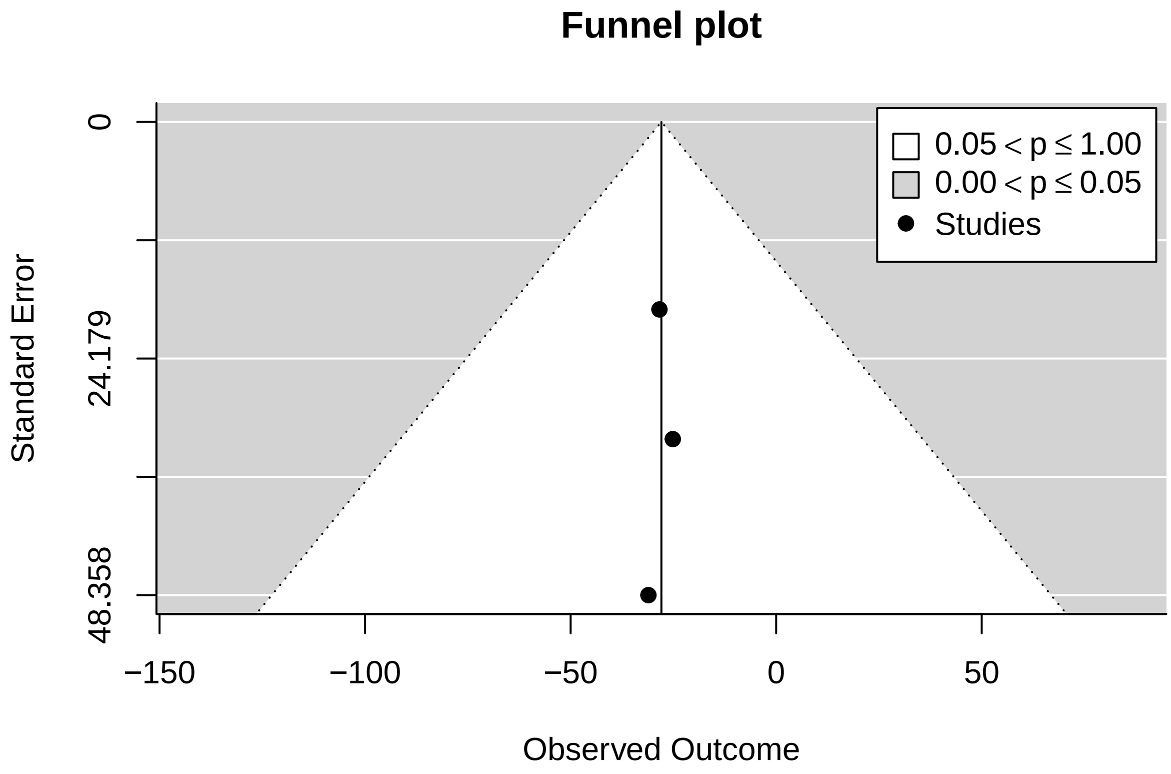

Model F0T2 (data from 3 studies; 3 randomized, 0 non-randomized; 2 with objective physical activity measure, 1 with subjective physical activity measure): The moderated effect of the intervention through area deprivation (reference: medium/low) at T2, adjusted for physical activity level at T0 and age

```
mod_imd_con_F0T2 = rma(yi=data_EQUAL_meta$X_IMD_CON_F0T2,
                       sei=data_EQUAL_meta$X_IMD_CON_F0T2/data_EQUAL_meta$t_X_IMD_CON_F0T2,
                       method='DL',slab=Study)
```

```
## Warning in rma(yi = data_EQUAL_meta$X_IMD_CON_F0T2, sei =
## data_EQUAL_meta$X_IMD_CON_F0T2/data_EQUAL_meta$t_X_IMD_CON_F0T2, : Studies with
## NAs omitted from model fitting.
```

```
summary(mod_imd_con_F0T2)
```

```
##
## Random-Effects Model (k = 3; tau^2 estimator: DL)
##
##   logLik  deviance       AIC       BIC      AICc
## -13.3949    1.2164   30.7897   28.9869   42.7897
##
## tau^2 (estimated amount of total heterogeneity): 0 (SE = 838.9142)
## tau (square root of estimated tau^2 value):      0
## I^2 (total heterogeneity / total variability):   0.00%
## H^2 (total variability / sampling variability):  1.00
##
## Test for Heterogeneity:
## Q(df = 2) = 1.2164, p-val = 0.5443
##
```

```
## Model Results:
##
## estimate       se     zval    pval     ci.lb   ci.ub
## -22.0596  14.6473  -1.5061  0.1321  -50.7678  6.6486
##
## ---
## Signif. codes:  0 '***' 0.001 '**' 0.01 '*' 0.05 '.' 0.1 ' ' 1
```

```
forest(mod_imd_con_F0T2,main="Forest plot",header='Study',xlab='')
mtext(c("Favors medium/low area deprivation", "Favors high area deprivation"),
      side=1, line=3, at=c(-10,10),adj=c(1,0))
arrows(x0=c(-10,10), y0=-3.3, x1=c(-100, 100), y1=-3.3, length=0.1, lwd=2, xpd=TRUE)
```

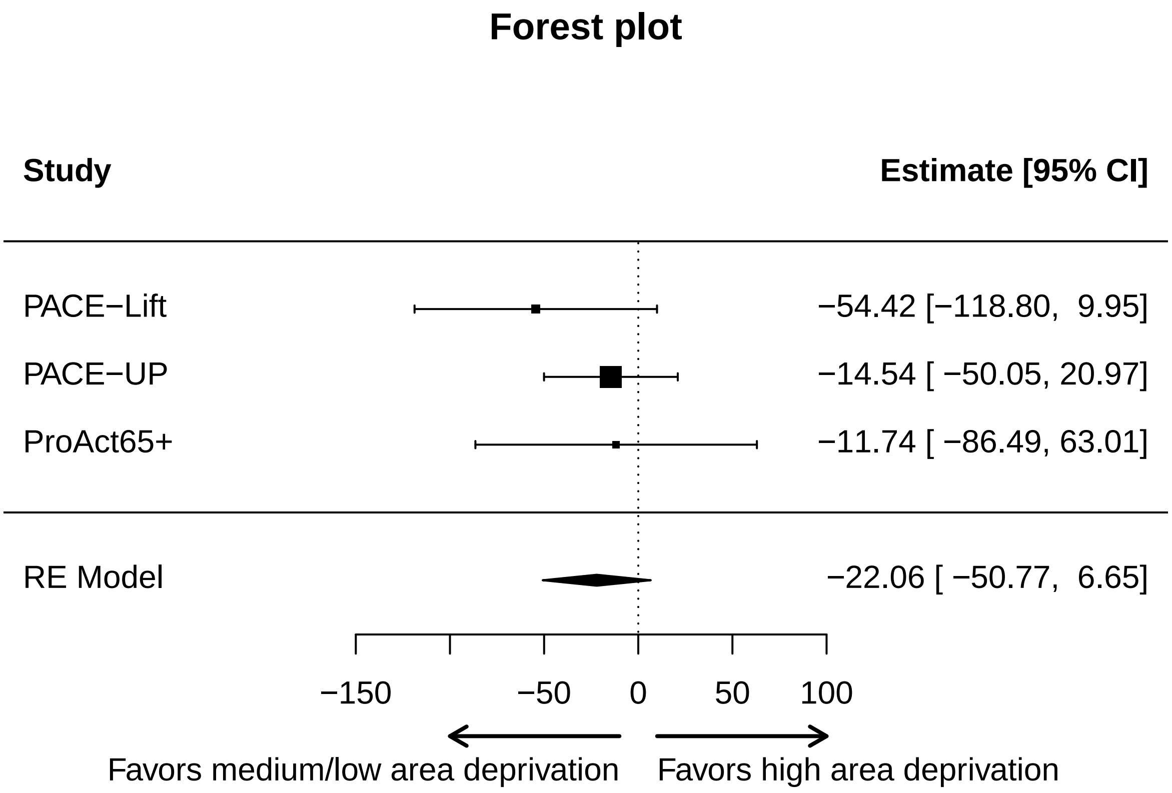


```
funnel(mod_imd_con_F0T2,legend=TRUE,main='Funnel plot')
```

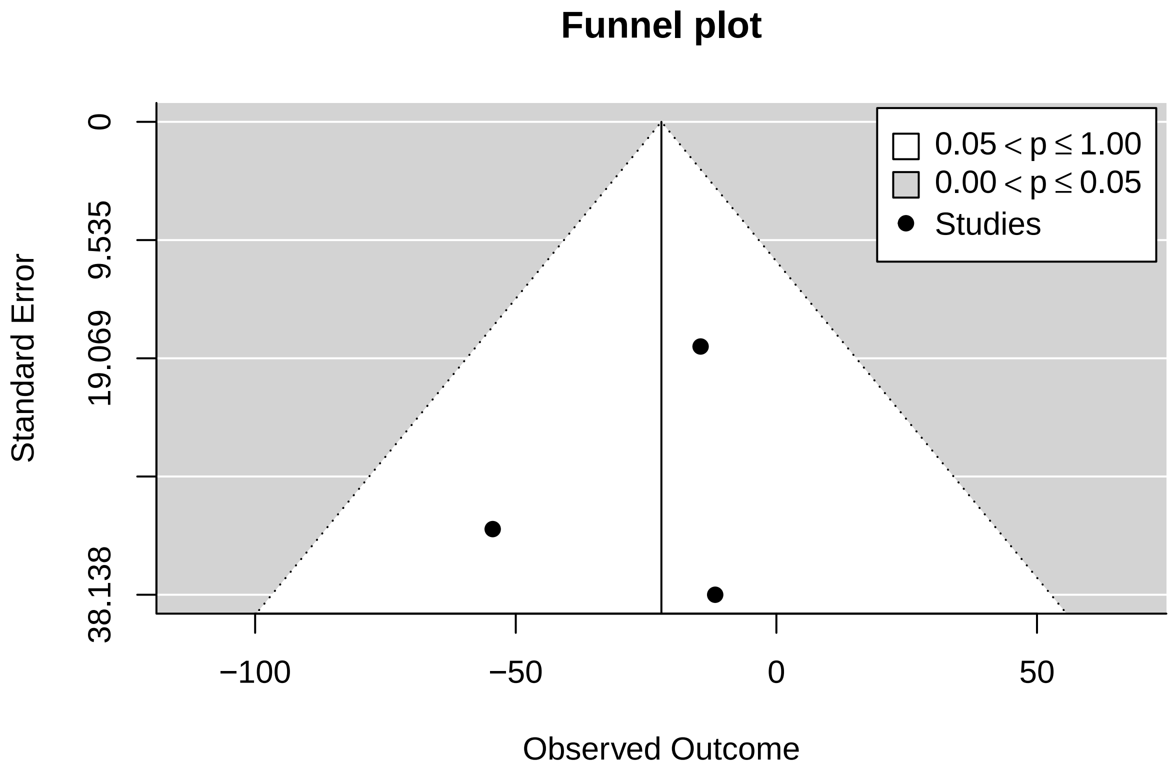

Model F2T2 (data from 3 studies; 3 randomized, 0 non-randomized; 2 with objective physical activity measure, 1 with subjective physical activity measure): The moderated effect of the intervention through area deprivation (reference: medium/low) at T2, adjusted for physical activity level at T0, age, gender, and the condition x gender interaction

```
mod_imd_con_F2T2 = rma(yi=data_EQUAL_meta$X_IMD_CON_F2T2,
                       sei=data_EQUAL_meta$X_IMD_CON_F2T2/data_EQUAL_meta$t_X_IMD_CON_F2T2,
                       method='DL',slab=Study)
```

```
## Warning in rma(yi = data_EQUAL_meta$X_IMD_CON_F2T2, sei =
## data_EQUAL_meta$X_IMD_CON_F2T2/data_EQUAL_meta$t_X_IMD_CON_F2T2, : Studies with
## NAs omitted from model fitting.
```

```
summary(mod_imd_con_F2T2)
```

```
##
## Random-Effects Model (k = 3; tau^2 estimator: DL)
##
##   logLik  deviance       AIC       BIC      AICc
## -13.2531    0.9462   30.5063   28.7035   42.5063
##
## tau^2 (estimated amount of total heterogeneity): 0 (SE = 835.0351)
## tau (square root of estimated tau^2 value):      0
## I^2 (total heterogeneity / total variability):   0.00%
## H^2 (total variability / sampling variability):  1.00
##
## Test for Heterogeneity:
## Q(df = 2) = 0.9462, p-val = 0.6231
```

```
##
## Model Results:
##
## estimate       se     zval    pval     ci.lb   ci.ub
## -21.7569  14.6297  -1.4872  0.1370  -50.4305  6.9168
##
## ---
## Signif. codes:  0 '***' 0.001 '**' 0.01 '*' 0.05 '.' 0.1 ' ' 1
```

```r
forest(mod_imd_con_F2T2,main="Forest plot",header='Study',xlab='')
mtext(c("Favors medium/low area deprivation", "Favors high area deprivation"),
      side=1, line=3, at=c(-10,10),adj=c(1,0))
arrows(x0=c(-10,10), y0=-3.3, x1=c(-100, 100), y1=-3.3, length=0.1, lwd=2, xpd=TRUE)
```

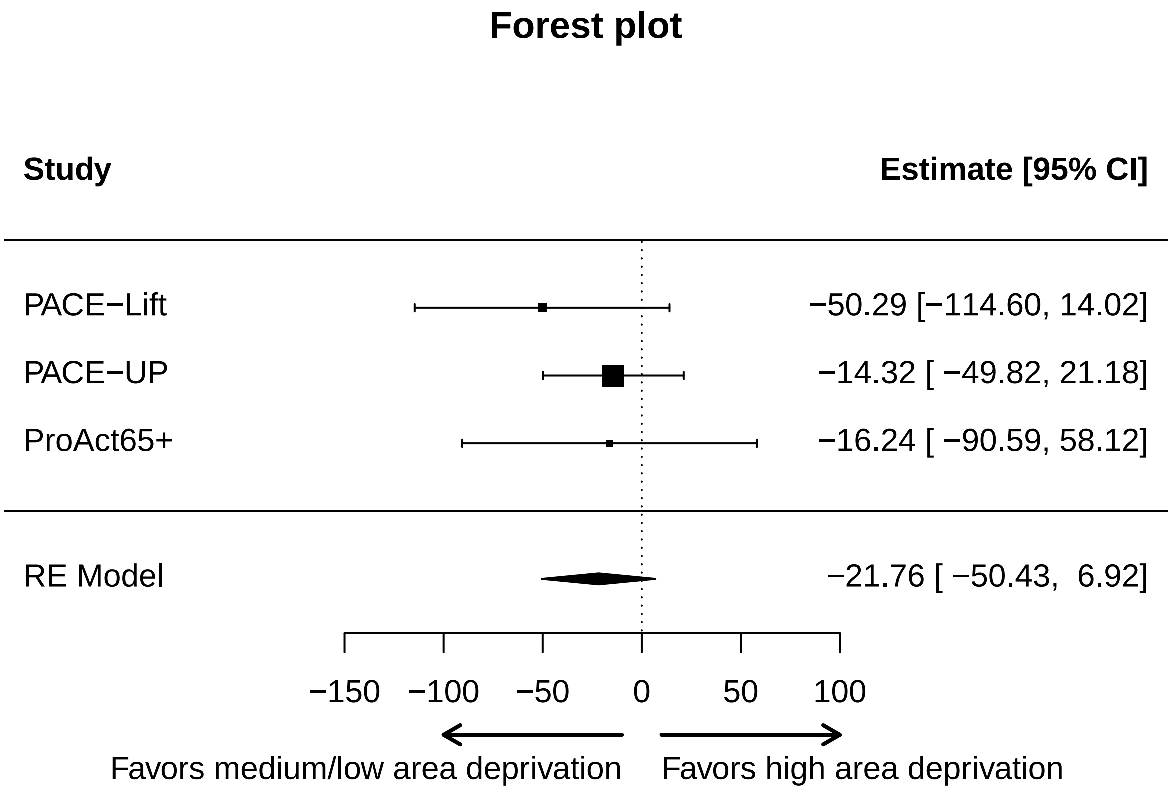


```r
funnel(mod_imd_con_F2T2,legend=TRUE,main='Funnel plot')
```

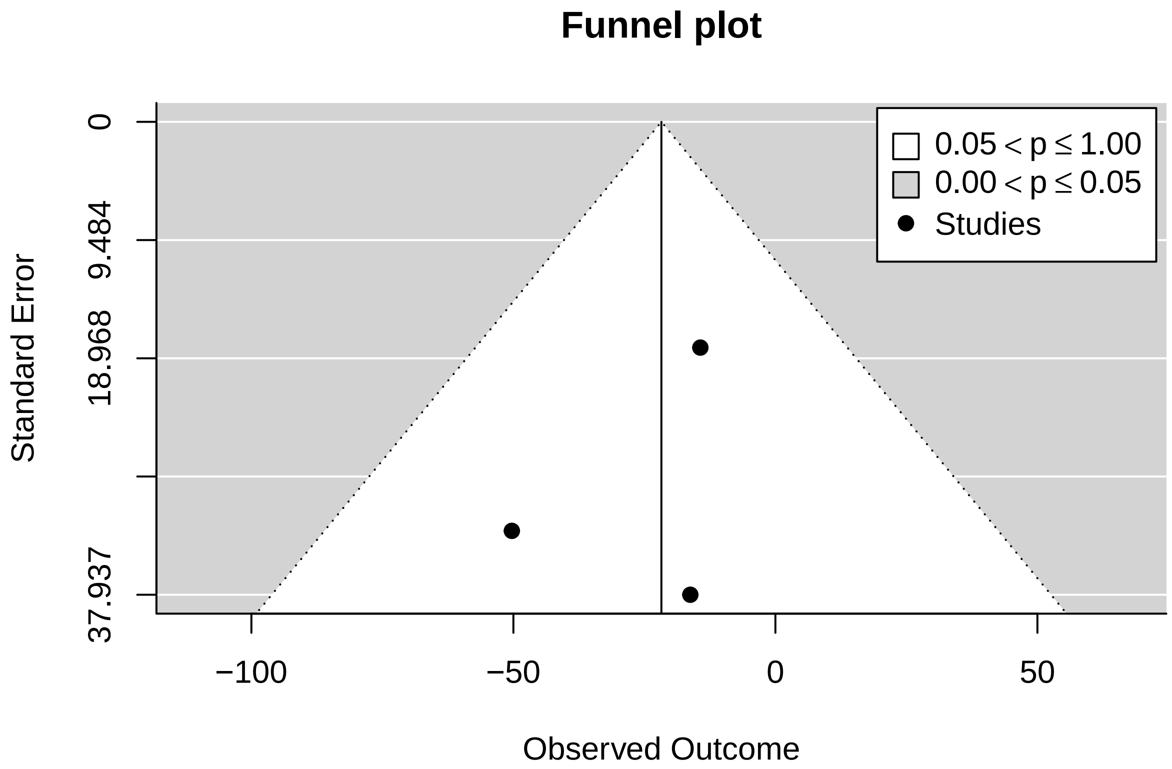
Funnel plot
0.05 < p ≤ 1.00
0.00 < p ≤ 0.05
Studies
Standard Error
0
9.484
18.968
37.937
−100
−50
0
50
Observed Outcome
