# Supplementary material for: Equity-specific effects of interventions to promote physical activity among middle-aged and older adults: results from applying a novel equity-specific re-analysis strategy
Source: Int J Behav Nutr Phys Act. 2021 May 17;18:65. doi: 10.1186/s12966-021-01131-w (PMC8130354; doi:10.1186/s12966-021-01131-w)
Supplement: Supplementary file 9 — Additional file 9. Detailed results of the meta-analyses using partial correlation coefficients. This file contains the complete output of the random-effects meta-analysis (main and secondary analysis) using partial correlation coefficients. [file 12966_2021_1131_MOESM9_ESM.pdf]

Random-effects meta-analysis of partial correlation coefficients

```
library(readxl)
library(metafor)
```

```
## Loading required package: Matrix
```

```
## Loading 'metafor' package (version 2.4-0). For an overview
## and introduction to the package please type: help(metafor).
```

```
data_EQUAL_meta = read_excel("data_EQUAL_meta.xlsx")
attach(data_EQUAL_meta)
```

Gender-specific intervention effects

Model B0T1 (data from 8 studies; 7 randomized, 1 non-randomized; 3 with objective physical activity measure, 5 with subjective physical activity measure): The moderated effect of the intervention through gender (reference: male) at T1 (follow-up time-point closest to intervention end point), adjusted for physical activity level at T0 (baseline) and age:

```
mod_gnd_con_B0T1 = rma(ti=data_EQUAL_meta$t_X_GND_CON_B0T1,
                       ni=data_EQUAL_meta$N_X_GND_CON_B0T1,
                       mi=data_EQUAL_meta$M_X_SOCIND_CON_ALL0T1T2,
                       measure="PCOR",slab=Study)
summary(mod_gnd_con_B0T1)
```

```
##
## Random-Effects Model (k = 8; tau^2 estimator: REML)
##
##   logLik  deviance       AIC       BIC      AICc
##   9.7524  -19.5049  -15.5049  -15.6131  -12.5049
##
## tau^2 (estimated amount of total heterogeneity): 0.0016 (SE = 0.0018)
## tau (square root of estimated tau^2 value):      0.0403
## I^2 (total heterogeneity / total variability):   50.65%
## H^2 (total variability / sampling variability):  2.03
##
## Test for Heterogeneity:
## Q(df = 7) = 14.1029, p-val = 0.0494
##
## Model Results:
##
## estimate      se    zval    pval    ci.lb   ci.ub
##   0.0152  0.0208  0.7311  0.4647  -0.0256  0.0560
##
## ---
## Signif. codes:  0 '***' 0.001 '**' 0.01 '*' 0.05 '.' 0.1 ' ' 1
```

```
forest(mod_gnd_con_B0T1,main="Forest plot",header='Study',xlab = '')
mtext(c("Favors males", "Favors females"), side=1, line=3, at=c(-.02,.02),adj=c(1,0))
```

```
arrows(x0=c(-.02,.02), y0=-4.4, x1=c(-.2, .2), y1=-4.4, length=0.1, lwd=2, xpd=TRUE)
```

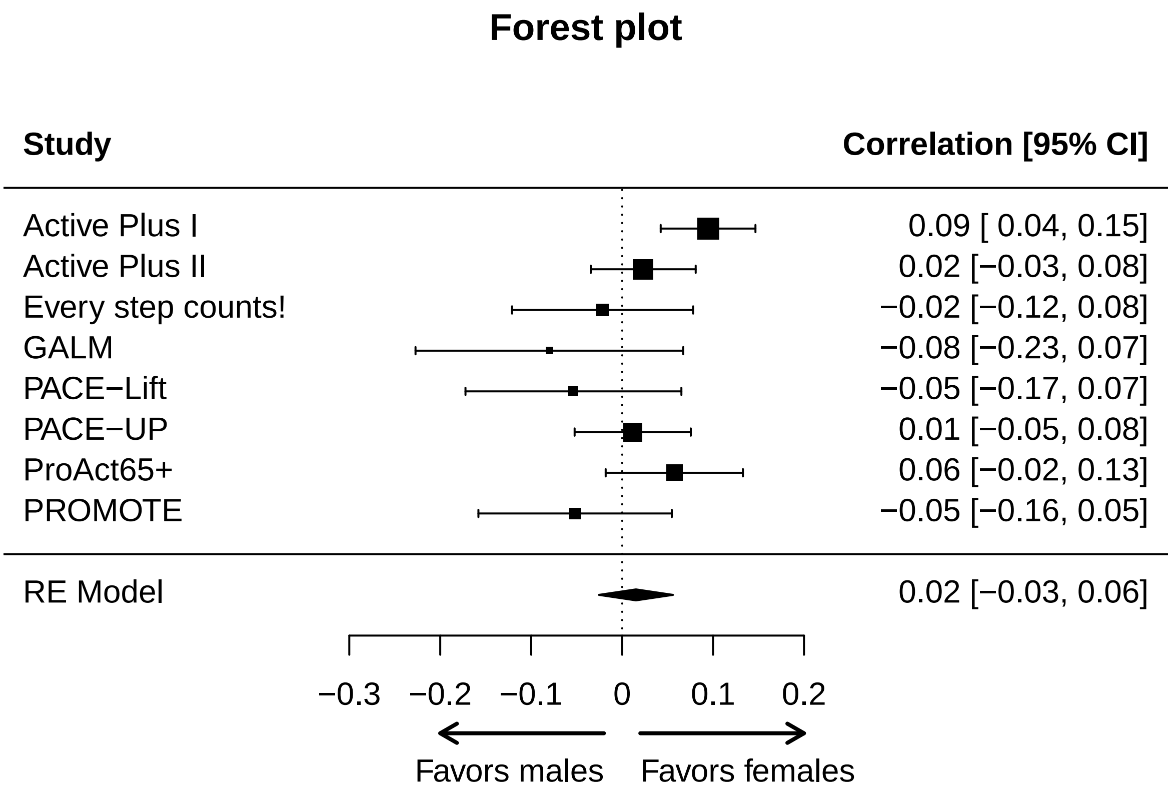


```
funnel(mod_gnd_con_BOT1,legend=TRUE,main='Funnel plot')
```

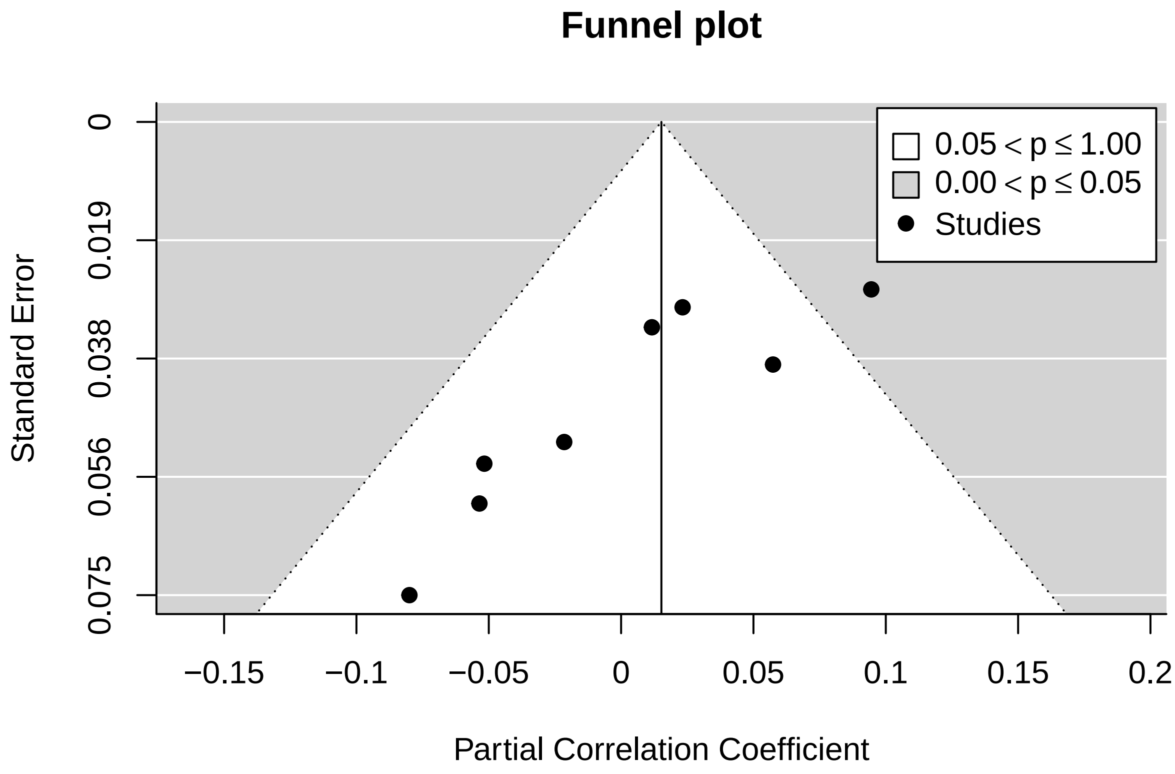

Model B2T1 (data from 8 studies; 7 randomized, 1 non-randomized; 3 with objective physical activity measure, 5 with subjective physical activity measure): The moderated effect of the intervention through gender (reference: male) at T1, adjusted for physical activity level at T0, age, education, and the condition x education interaction:

```
mod_gnd_con_B2T1 = rma(ti=data_EQUAL_meta$t_X_GND_CON_B2T1,
                       ni=data_EQUAL_meta$N_X_GNDEDU_CON_B2C2T1,
                       mi=data_EQUAL_meta$M_X_SOCIND_CON_ALL2T1T2,
                       measure="PCOR",slab=Study)
summary(mod_gnd_con_B2T1)
```

```
##
## Random-Effects Model (k = 8; tau^2 estimator: REML)
##
##   logLik  deviance       AIC       BIC      AICc
##   9.0134  -18.0267  -14.0267  -14.1349  -11.0267
##
## tau^2 (estimated amount of total heterogeneity): 0.0024 (SE = 0.0022)
## tau (square root of estimated tau^2 value):      0.0485
## I^2 (total heterogeneity / total variability):   59.45%
## H^2 (total variability / sampling variability):  2.47
##
## Test for Heterogeneity:
## Q(df = 7) = 17.1689, p-val = 0.0163
##
## Model Results:
##
```

```
## estimate      se    zval    pval    ci.lb   ci.ub
##   0.0072  0.0232  0.3098  0.7567  -0.0382  0.0526
##
## ---
## Signif. codes:  0 '***' 0.001 '**' 0.01 '*' 0.05 '.' 0.1 ' ' 1
```

```
forest(mod_gnd_con_B2T1,main="Forest plot",header='Study',xlab = '')
mtext(c("Favors males", "Favors females"), side=1, line=3, at=c(-.02,.02),adj=c(1,0))
arrows(x0=c(-.02,.02), y0=-4.4, x1=c(-.2, .2), y1=-4.4, length=0.1, lwd=2, xpd=TRUE)
```

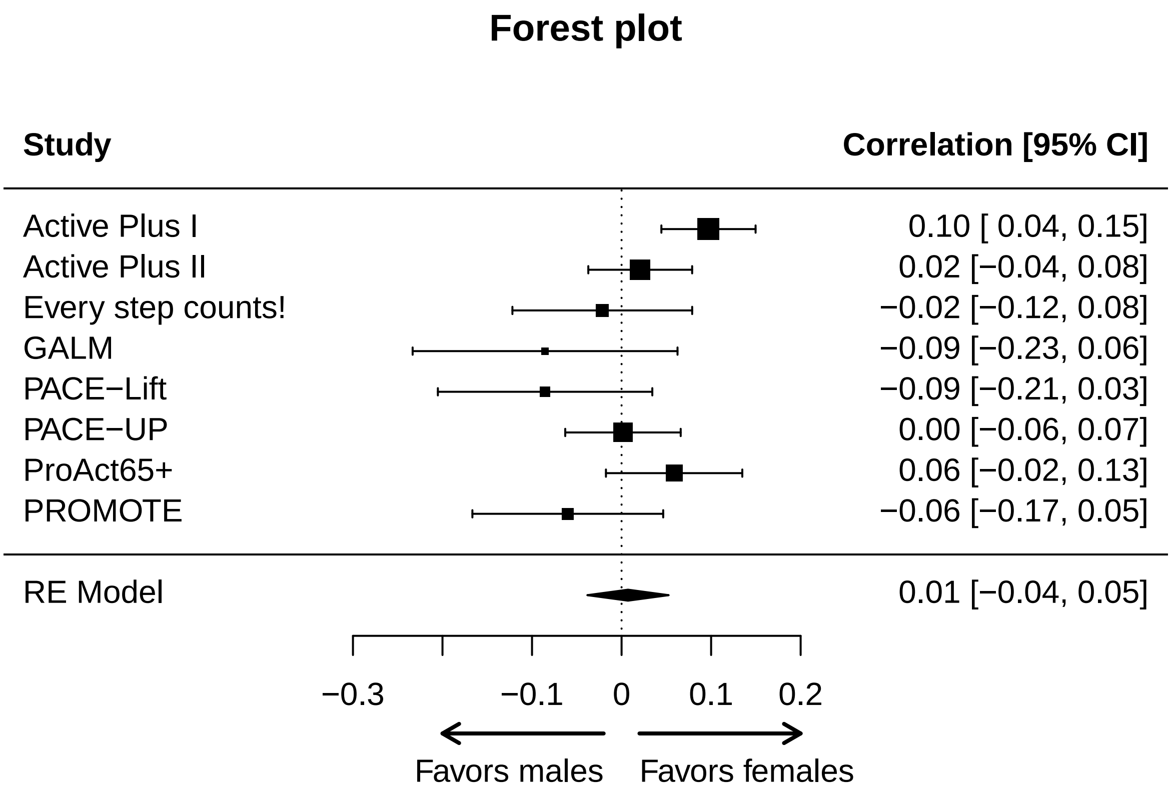


```
funnel(mod_gnd_con_B2T1,legend=TRUE,main='Funnel plot')
```

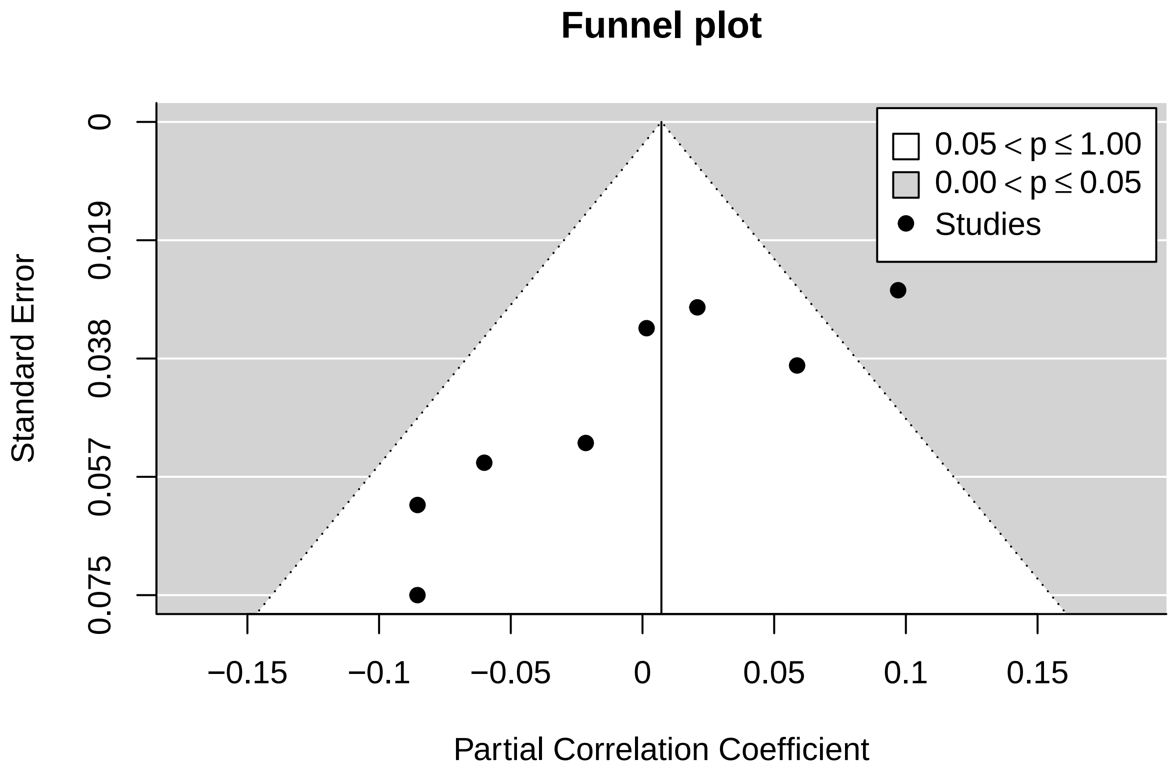

Model B0T2 (data from 5 studies; 5 randomized, 0 non-randomized; 2 with objective physical activity measure, 3 with subjective physical activity measure): The moderated effect of the intervention through gender (reference: male) at T2 (next follow-up assessment), adjusted for physical activity level at T0 and age

```
mod_gnd_con_B0T2 = rma(ti=data_EQUAL_meta$t_X_GND_CON_B0T2,
                       ni=data_EQUAL_meta$N_X_GND_CON_B0T2,
                       mi=data_EQUAL_meta$M_X_SOCIND_CON_ALL0T1T2,
                       measure="PCOR",slab=Study)
```

```
## Warning in rma(ti = data_EQUAL_meta$t_X_GND_CON_B0T2, ni =
## data_EQUAL_meta$N_X_GND_CON_B0T2, : Studies with NAs omitted from model fitting.
```

```
summary(mod_gnd_con_B0T2)
```

```
##
## Random-Effects Model (k = 5; tau^2 estimator: REML)
##
##   logLik  deviance       AIC       BIC      AICc
##   6.7570  -13.5140   -9.5140  -10.7414    2.4860
##
## tau^2 (estimated amount of total heterogeneity): 0.0005 (SE = 0.0012)
## tau (square root of estimated tau^2 value):      0.0225
## I^2 (total heterogeneity / total variability):   29.55%
## H^2 (total variability / sampling variability):  1.42
##
## Test for Heterogeneity:
## Q(df = 4) = 5.8000, p-val = 0.2146
##
```

```
## Model Results:
##
## estimate      se    zval    pval    ci.lb   ci.ub
##   0.0188  0.0186  1.0099  0.3125  -0.0177  0.0552
##
## ---
## Signif. codes:  0 '***' 0.001 '**' 0.01 '*' 0.05 '.' 0.1 ' ' 1
```

```r
forest(mod_gnd_con_BOT2,main="Forest plot",header='Study',xlab = '')
mtext(c("Favors males", "Favors females"), side=1, line=3, at=c(-.02,.02),adj=c(1,0))
arrows(x0=c(-.02,.02), y0=-3.8, x1=c(-.15, .15), y1=-3.8, length=0.1, lwd=2, xpd=TRUE)
```

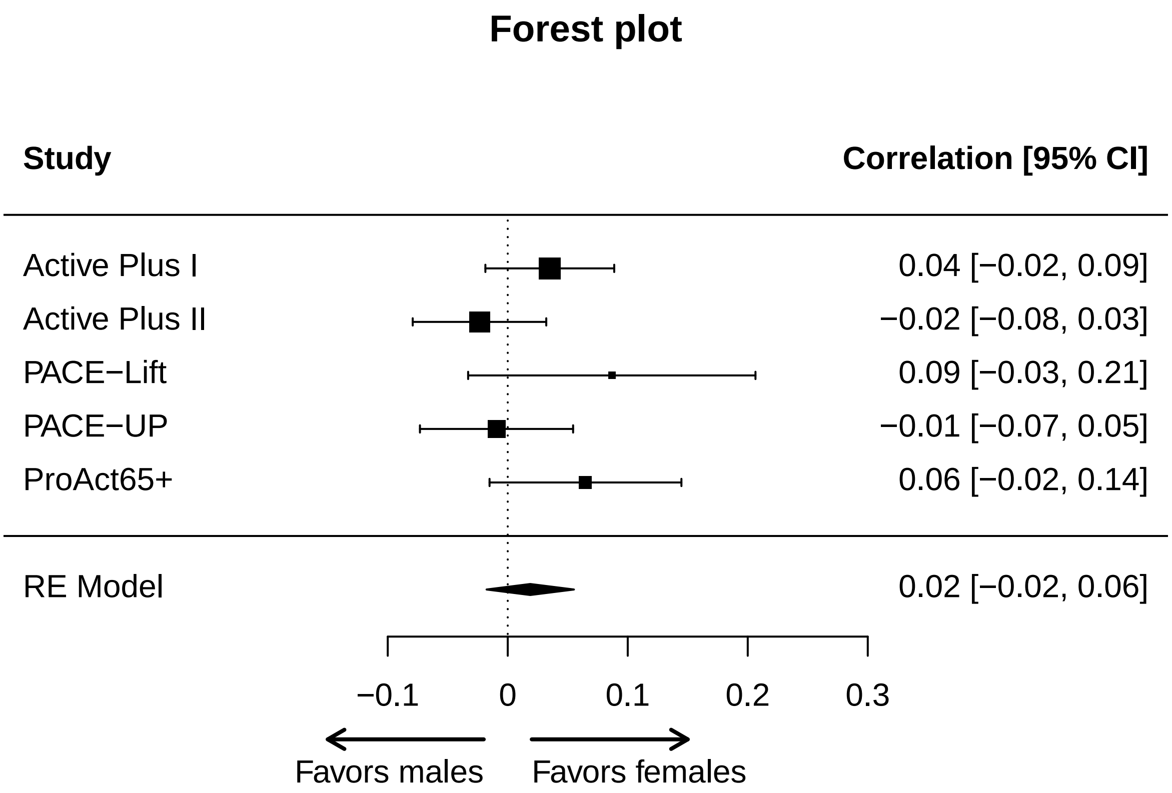


```r
funnel(mod_gnd_con_BOT2,legend=TRUE,main='Funnel plot')
```

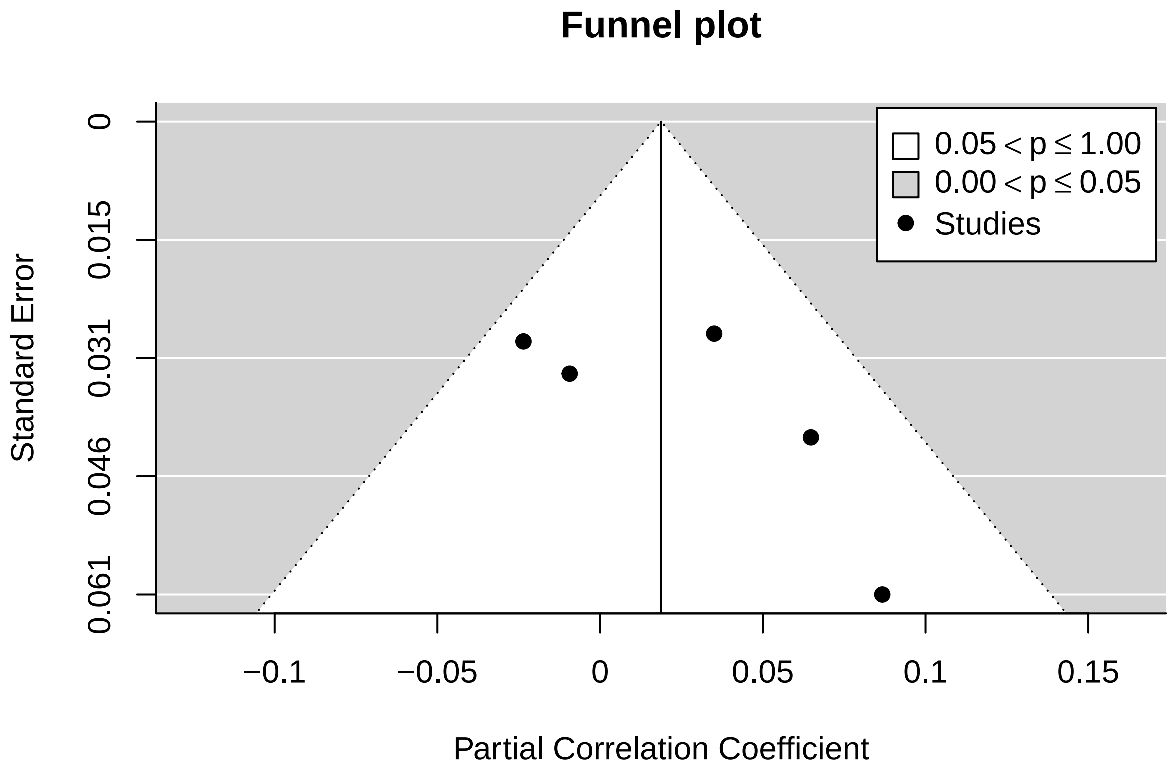

Model B2T2 (data from 5 studies; 5 randomized, 0 non-randomized; 2 with objective physical activity measure, 3 with subjective physical activity measure): The moderated effect of the intervention through gender (reference: male) at T2, adjusted for physical activity level at T0, age, education, and the condition x education interaction

```
mod_gnd_con_B2T2 = rma(ti=data_EQUAL_meta$t_X_GND_CON_B2T2,
                       ni=data_EQUAL_meta$N_X_GNDEDU_CON_B2C2T2,
                       mi=data_EQUAL_meta$M_X_SOCIND_CON_ALL2T1T2,
                       measure="PCOR",slab=Study)
```

```
## Warning in rma(ti = data_EQUAL_meta$t_X_GND_CON_B2T2, ni =
## data_EQUAL_meta$N_X_GNDEDU_CON_B2C2T2, : Studies with NAs omitted from model
## fitting.
```

```
summary(mod_gnd_con_B2T2)
```

```
##
## Random-Effects Model (k = 5; tau^2 estimator: REML)
##
##   logLik  deviance       AIC       BIC      AICc
##   7.0478  -14.0956  -10.0956  -11.3230    1.9044
##
## tau^2 (estimated amount of total heterogeneity): 0.0003 (SE = 0.0010)
## tau (square root of estimated tau^2 value):      0.0170
## I^2 (total heterogeneity / total variability):   19.17%
## H^2 (total variability / sampling variability):  1.24
##
## Test for Heterogeneity:
```

```
## Q(df = 4) = 4.9501, p-val = 0.2925
##
## Model Results:
##
## estimate      se    zval    pval    ci.lb   ci.ub
##   0.0158  0.0173  0.9129  0.3613  -0.0182  0.0498
##
## ---
## Signif. codes:  0 '***' 0.001 '**' 0.01 '*' 0.05 '.' 0.1 ' ' 1
```

```r
forest(mod_gnd_con_B2T2,main="Forest plot",header='Study',xlab = '')
mtext(c("Favors males", "Favors females"), side=1, line=3, at=c(-.02,.02),adj=c(1,0))
arrows(x0=c(-.02,.02), y0=-3.8, x1=c(-.15, .15), y1=-3.8, length=0.1, lwd=2, xpd=TRUE)
```

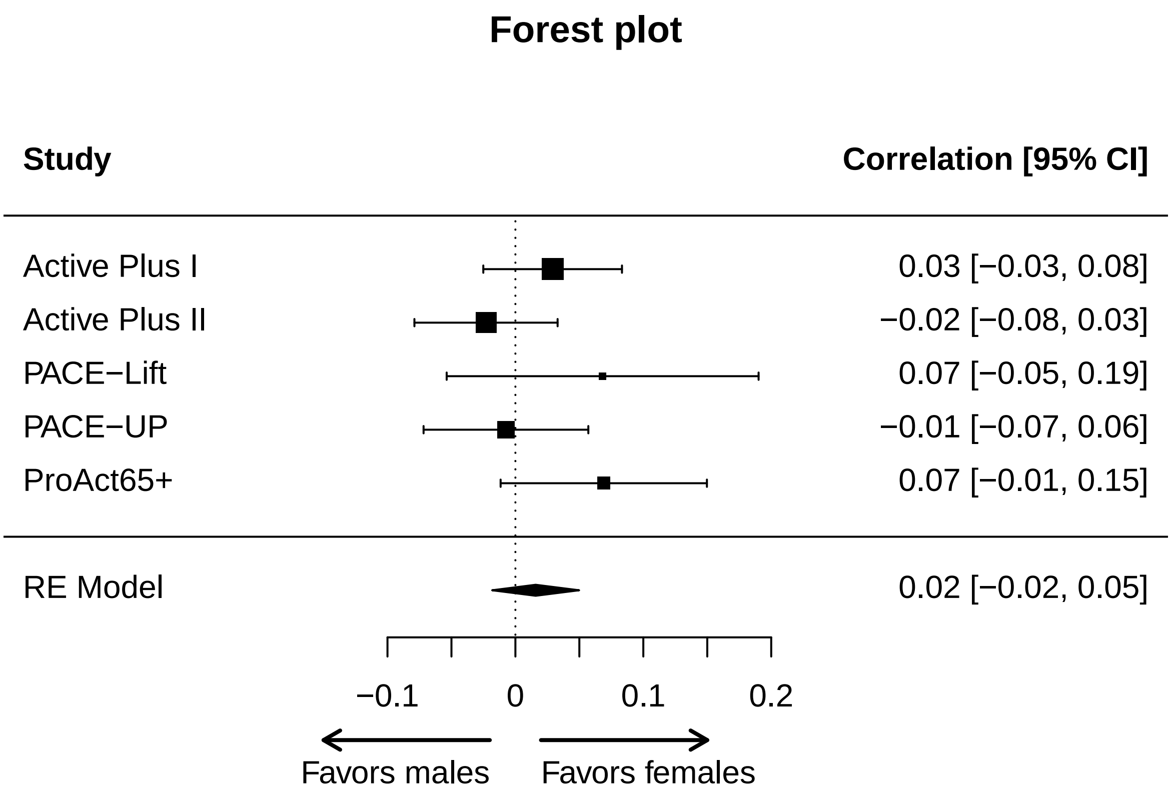


```r
funnel(mod_gnd_con_B2T2,legend=TRUE,main='Funnel plot')
```

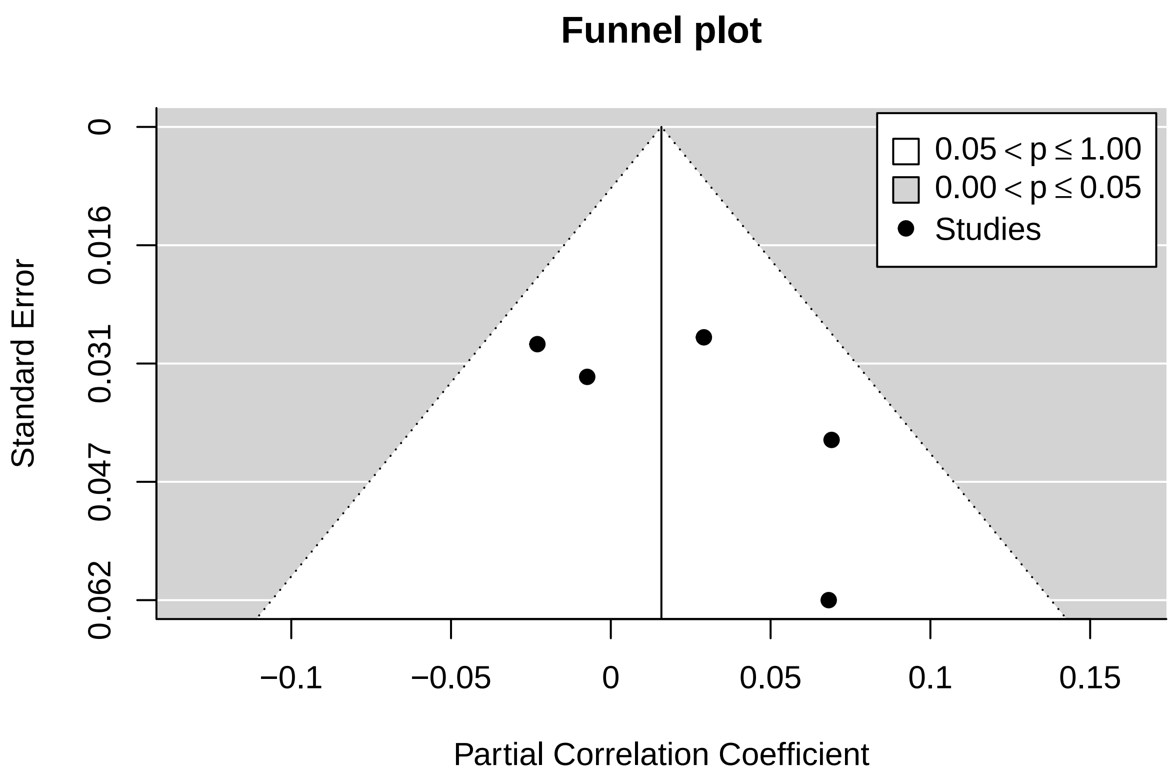

Education-specific intervention effects

Model C0T1 (data from 8 studies; 7 randomized, 1 non-randomized; 3 with objective physical activity measure, 5 with subjective physical activity measure): The moderated effect of the intervention through education (reference: medium/high) at T2, adjusted for physical activity level at T0 and age

```
mod_edu_con_C0T1 = rma(ti=data_EQUAL_meta$t_X_EDU_CON_C0T1,
                       ni=data_EQUAL_meta$N_X_EDU_CON_C0T1,
                       mi=data_EQUAL_meta$M_X_SOCIND_CON_ALL0T1T2,
                       measure="PCOR",slab=Study)
summary(mod_edu_con_C0T1)
```

```
##
## Random-Effects Model (k = 8; tau^2 estimator: REML)
##
##   logLik  deviance       AIC       BIC      AICc
##   9.9623  -19.9247  -15.9247  -16.0328  -12.9247
##
## tau^2 (estimated amount of total heterogeneity): 0.0012 (SE = 0.0015)
## tau (square root of estimated tau^2 value):      0.0340
## I^2 (total heterogeneity / total variability):   41.90%
## H^2 (total variability / sampling variability):  1.72
##
## Test for Heterogeneity:
## Q(df = 7) = 12.1293, p-val = 0.0964
##
```

```
## Model Results:
##
## estimate      se    zval    pval    ci.lb   ci.ub
##   0.0013  0.0192  0.0674  0.9463  -0.0364  0.0389
##
## ---
## Signif. codes:  0 '***' 0.001 '**' 0.01 '*' 0.05 '.' 0.1 ' ' 1
```

```
forest(mod_edu_con_COT1,main="Forest plot",header='Study',xlab = '')
mtext(c("Favors medium/high education", "Favors low education"),
      side=1, line=3, at=c(-.02,.02),adj=c(1,0))
arrows(x0=c(-.02,.02), y0=-4.4, x1=c(-.2, .2), y1=-4.4, length=0.1, lwd=2, xpd=TRUE)
```

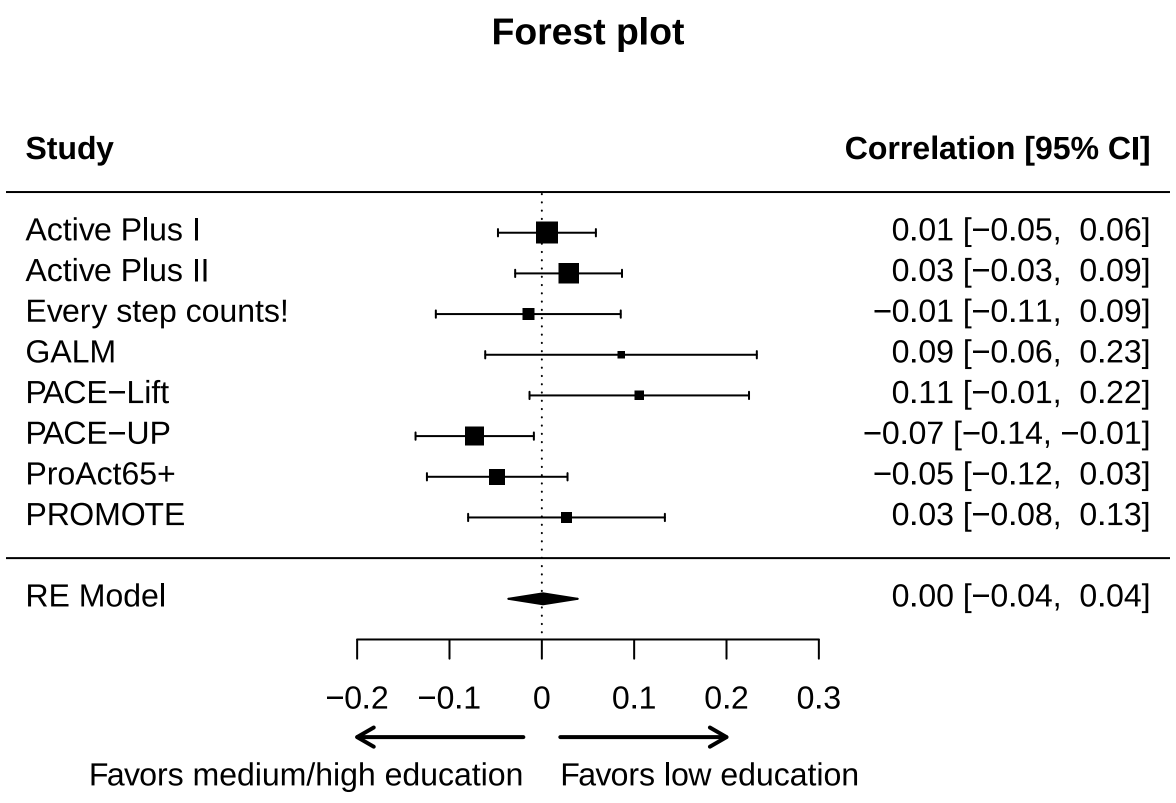


```
funnel(mod_edu_con_COT1,legend=TRUE,main='Funnel plot')
```

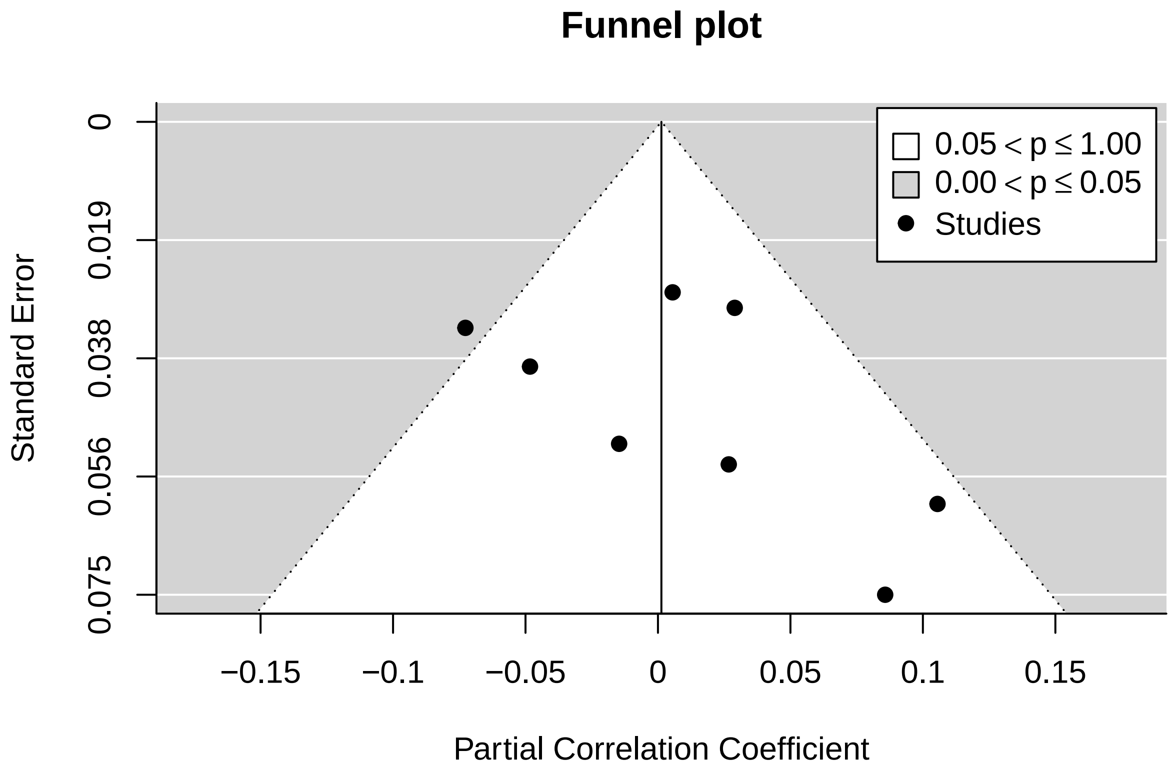

Model C2T1 (data from 8 studies; 7 randomized, 1 non-randomized; 3 with objective physical activity measure, 5 with subjective physical activity measure): The moderated effect of the intervention through education (reference: medium/high) at T1, adjusted for physical activity level at T0, age, gender, and the condition x gender interaction

```
mod_edu_con_C2T1 = rma(ti=data_EQUAL_meta$t_X_EDU_CON_C2T1,
                       ni=data_EQUAL_meta$N_X_GNDEDU_CON_B2C2T1,
                       mi=data_EQUAL_meta$M_X_SOCIND_CON_ALL2T1T2,
                       measure="PCOR",slab=Study)
summary(mod_edu_con_C2T1)
```

```
##
## Random-Effects Model (k = 8; tau^2 estimator: REML)
##
##   logLik  deviance       AIC       BIC      AICc
##   9.5899  -19.1799  -15.1799  -15.2881  -12.1799
##
## tau^2 (estimated amount of total heterogeneity): 0.0013 (SE = 0.0016)
## tau (square root of estimated tau^2 value):      0.0363
## I^2 (total heterogeneity / total variability):   44.95%
## H^2 (total variability / sampling variability):  1.82
##
## Test for Heterogeneity:
## Q(df = 7) = 12.8672, p-val = 0.0754
##
## Model Results:
##
```

```
## estimate      se      zval    pval    ci.lb   ci.ub
##  -0.0014  0.0198  -0.0704  0.9439  -0.0402  0.0374
##
## ---
## Signif. codes:  0 '***' 0.001 '**' 0.01 '*' 0.05 '.' 0.1 ' ' 1
```

```
forest(mod_edu_con_C2T1,main="Forest plot",header='Study',xlab = '')
mtext(c("Favors medium/high education", "Favors low education"),
      side=1, line=3, at=c(-.02,.02),adj=c(1,0))
arrows(x0=c(-.02,.02), y0=-4.4, x1=c(-.2, .2), y1=-4.4, length=0.1, lwd=2, xpd=TRUE)
```

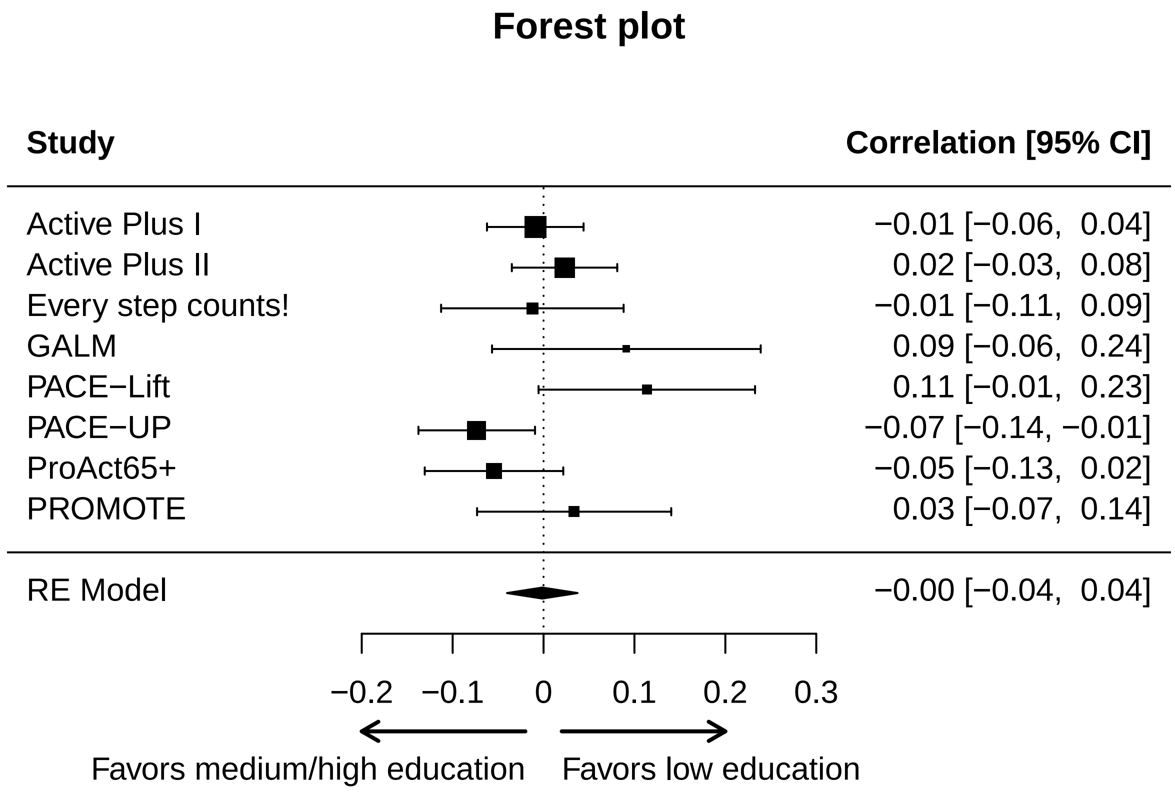


```
funnel(mod_edu_con_C2T1,legend=TRUE,main='Funnel plot')
```

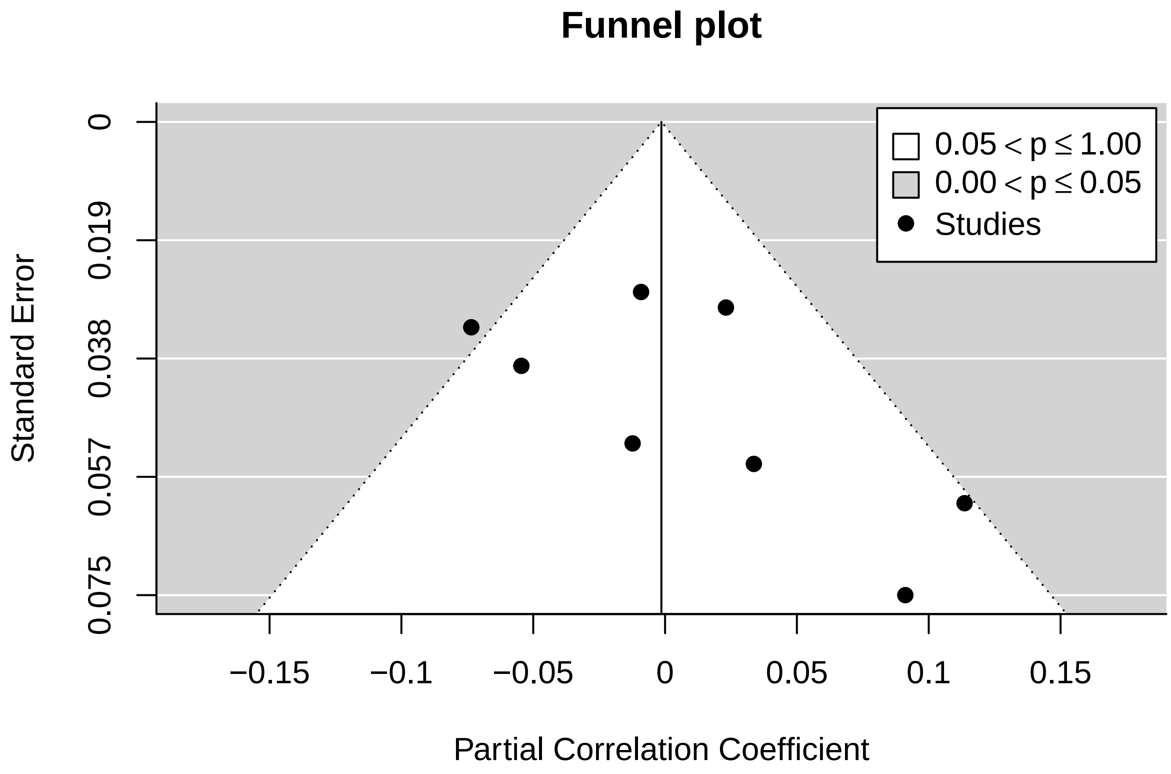

Model C0T2 (data from 5 studies; 5 randomized, 0 non-randomized; 2 with objective physical activity measure, 3 with subjective physical activity measure): The moderated effect of the intervention through education (reference: medium/high) at T2, adjusted for physical activity level at T0 and age

```
mod_edu_con_C0T2 = rma(ti=data_EQUAL_meta$t_X_EDU_CON_C0T2,
                       ni=data_EQUAL_meta$N_X_EDU_CON_C0T2,
                       mi=data_EQUAL_meta$M_X_SOCIND_CON_ALL0T1T2,
                       measure="PCOR",slab=Study)
```

```
## Warning in rma(ti = data_EQUAL_meta$t_X_EDU_CON_C0T2, ni =
## data_EQUAL_meta$N_X_EDU_CON_C0T2, : Studies with NAs omitted from model fitting.
```

```
summary(mod_edu_con_C0T2)
```

```
##
## Random-Effects Model (k = 5; tau^2 estimator: REML)
##
##   logLik  deviance       AIC       BIC      AICc
##   6.2737  -12.5474   -8.5474   -9.7748    3.4526
##
## tau^2 (estimated amount of total heterogeneity): 0.0013 (SE = 0.0018)
## tau (square root of estimated tau^2 value):      0.0354
## I^2 (total heterogeneity / total variability):   50.72%
## H^2 (total variability / sampling variability):  2.03
##
## Test for Heterogeneity:
## Q(df = 4) = 7.7678, p-val = 0.1005
##
```

```
## Model Results:
##
## estimate      se     zval    pval    ci.lb   ci.ub
##  -0.0071  0.0227  -0.3126  0.7546  -0.0515  0.0374
##
## ---
## Signif. codes:  0 '***' 0.001 '**' 0.01 '*' 0.05 '.' 0.1 ' ' 1
```

```r
forest(mod_edu_con_COT2,main="Forest plot",header='Study',xlab = '')
mtext(c("Favors medium/high education", "Favors low education"),
      side=1, line=3, at=c(-.02,.02),adj=c(1,0))
arrows(x0=c(-.02,.02), y0=-3.8, x1=c(-.15, .15), y1=-3.8, length=0.1, lwd=2, xpd=TRUE)
```

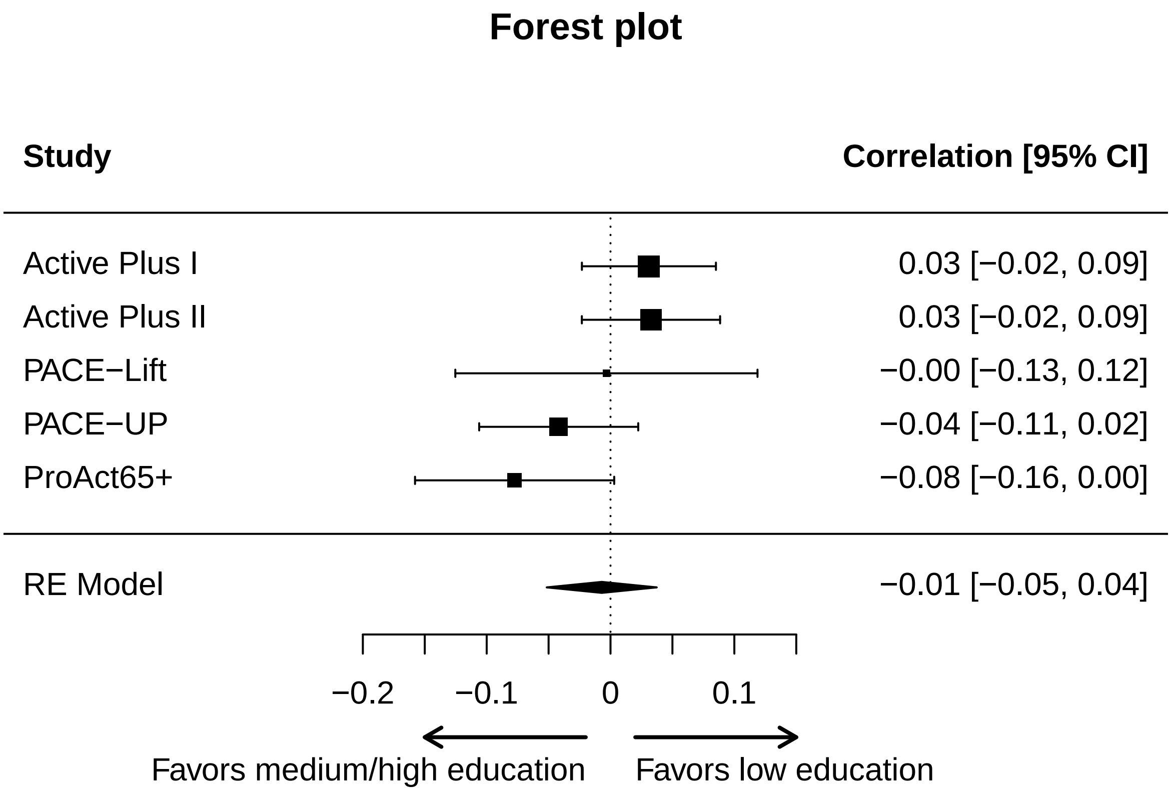


```r
funnel(mod_edu_con_COT2,legend=TRUE,main='Funnel plot')
```

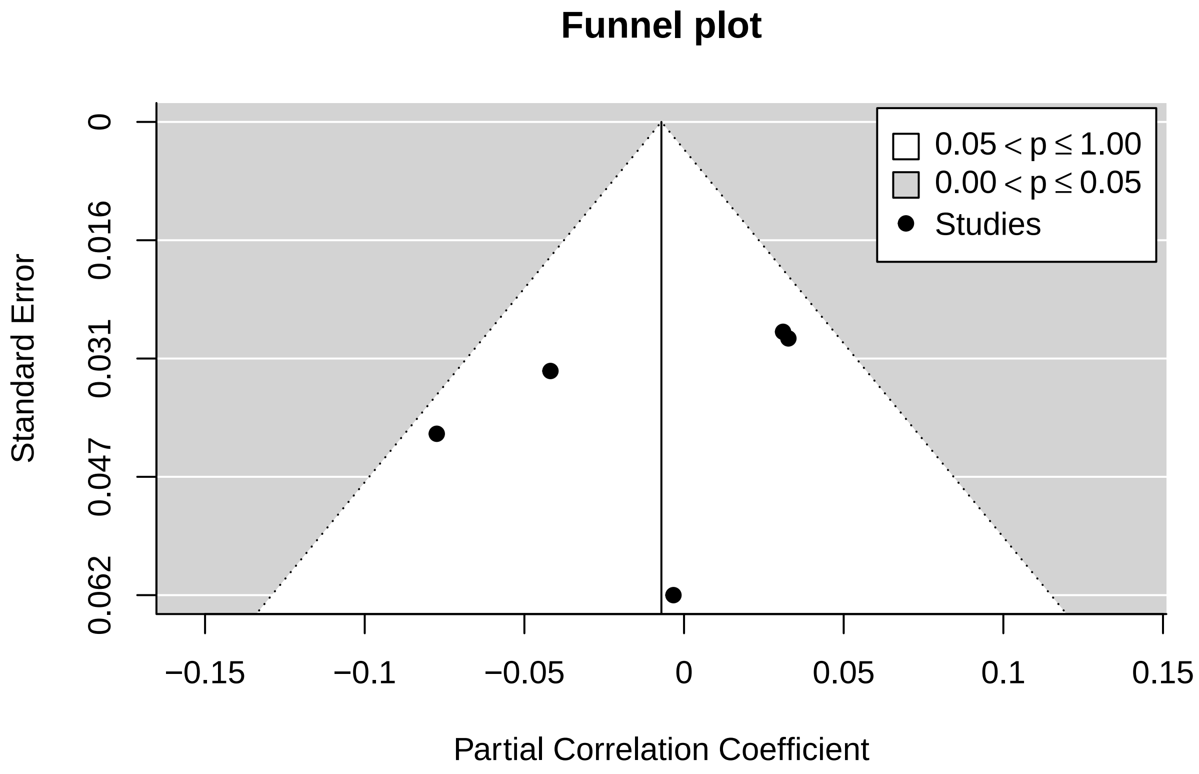

Model C2T2 (data from 5 studies; 5 randomized, 0 non-randomized; 2 with objective physical activity measure, 3 with subjective physical activity measure): The moderated effect of the intervention through education (reference: medium/high) at T2, adjusted for physical activity level at T0, age, gender, and the condition x gender interaction

```
mod_edu_con_C2T2 = rma(ti=data_EQUAL_meta$t_X_EDU_CON_C2T2,
                       ni=data_EQUAL_meta$N_X_GNDEDU_CON_B2C2T2,
                       mi=data_EQUAL_meta$M_X_SOCIND_CON_ALL2T1T2,
                       measure="PCOR",slab=Study)
```

```
## Warning in rma(ti = data_EQUAL_meta$t_X_EDU_CON_C2T2, ni =
## data_EQUAL_meta$N_X_GNDEDU_CON_B2C2T2, : Studies with NAs omitted from model
## fitting.
```

```
summary(mod_edu_con_C2T2)
```

```
##
## Random-Effects Model (k = 5; tau^2 estimator: REML)
##
##   logLik  deviance       AIC       BIC      AICc
##   6.1556  -12.3112   -8.3112   -9.5386    3.6888
##
## tau^2 (estimated amount of total heterogeneity): 0.0014 (SE = 0.0019)
## tau (square root of estimated tau^2 value):      0.0375
## I^2 (total heterogeneity / total variability):   53.51%
## H^2 (total variability / sampling variability):  2.15
##
## Test for Heterogeneity:
```

```
## Q(df = 4) = 8.2430, p-val = 0.0831
##
## Model Results:
##
## estimate      se     zval    pval    ci.lb   ci.ub
##  -0.0089  0.0234  -0.3797  0.7041  -0.0548  0.0370
##
## ---
## Signif. codes:  0 '***' 0.001 '**' 0.01 '*' 0.05 '.' 0.1 ' ' 1
```

```
forest(mod_edu_con_C2T2,main="Forest plot",header='Study',xlab = '')
mtext(c("Favors medium/high education", "Favors low education"),
      side=1, line=3, at=c(-.02,.02),adj=c(1,0))
arrows(x0=c(-.02,.02), y0=-3.8, x1=c(-.15, .15), y1=-3.8, length=0.1, lwd=2, xpd=TRUE)
```

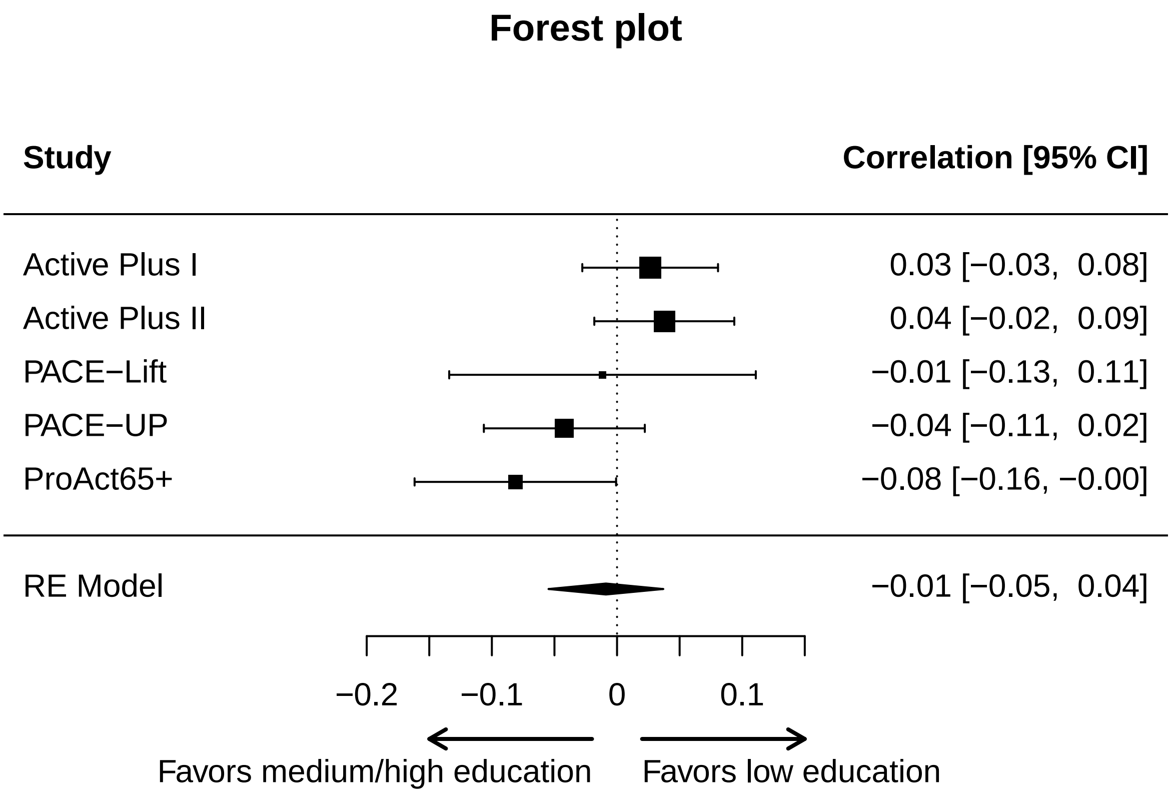


```
funnel(mod_edu_con_C2T2,legend=TRUE,main='Funnel plot')
```

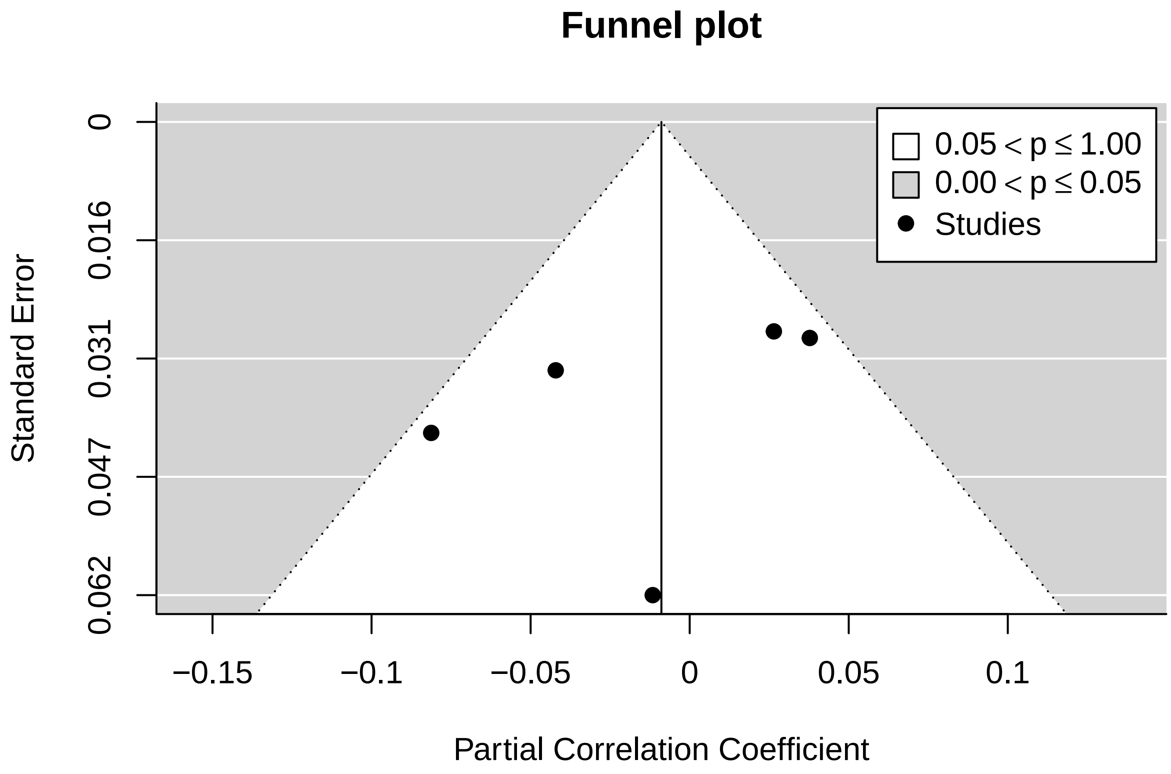

Marital status-specific intervention effects

Model D0T1 (data from 8 studies; 7 randomized, 1 non-randomized; 3 with objective physical activity measure, 5 with subjective physical activity measure): The moderated effect of the intervention through marital status (reference: with partner) at T1, adjusted for physical activity level at T0 and age

```
mod_mar_con_D0T1 = rma(ti=data_EQUAL_meta$t_X_MAR_CON_D0T1,
                       ni=data_EQUAL_meta$N_X_MAR_CON_D0T1,
                       mi=data_EQUAL_meta$M_X_SOCIND_CON_ALL0T1T2,
                       measure="PCOR",slab=Study)
summary(mod_mar_con_D0T1)
```

```
##
## Random-Effects Model (k = 8; tau^2 estimator: REML)
##
##   logLik  deviance       AIC       BIC      AICc
##  12.6290  -25.2581  -21.2581  -21.3663  -18.2581
##
## tau^2 (estimated amount of total heterogeneity): 0.0000 (SE = 0.0007)
## tau (square root of estimated tau^2 value):      0.0027
## I^2 (total heterogeneity / total variability):   0.46%
## H^2 (total variability / sampling variability):  1.00
##
## Test for Heterogeneity:
## Q(df = 7) = 5.5467, p-val = 0.5936
##
```

```
## Model Results:
##
## estimate      se    zval    pval    ci.lb   ci.ub
##   0.0104  0.0138  0.7560  0.4496  -0.0166  0.0375
##
## ---
## Signif. codes:  0 '***' 0.001 '**' 0.01 '*' 0.05 '.' 0.1 ' ' 1
```

```r
forest(mod_mar_con_DOT1,main="Forest plot",header='Study',xlab = '')
mtext(c("Favors individuals with partner", "Favors individuals without partner"),
      side=1, line=3, at=c(-.02,.02),adj=c(1,0))
arrows(x0=c(-.02,.02), y0=-4.4, x1=c(-.2, .2), y1=-4.4, length=0.1, lwd=2, xpd=TRUE)
```

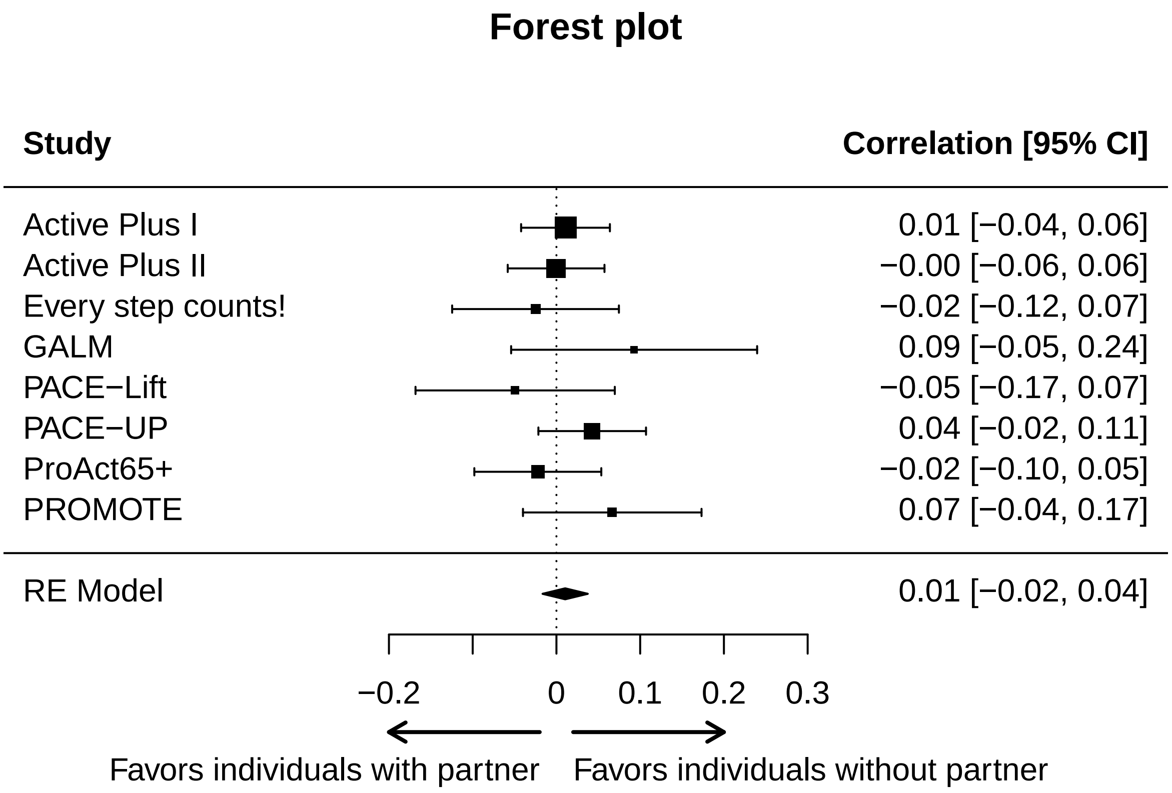


```r
funnel(mod_mar_con_DOT1,legend=TRUE,main='Funnel plot')
```

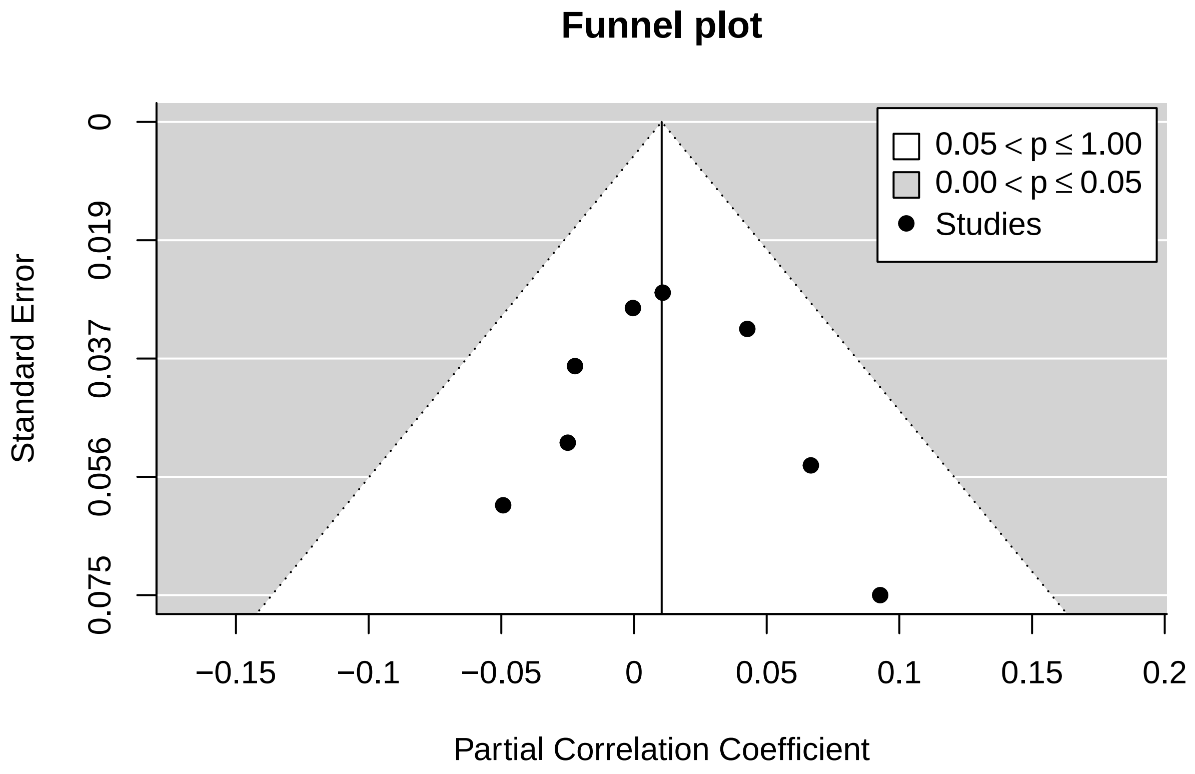

Model D2T1 (data from 8 studies; 7 randomized, 1 non-randomized; 3 with objective physical activity measure, 5 with subjective physical activity measure): The moderated effect of the intervention through marital status (reference: with partner) at T1, adjusted for physical activity level at T0, age, gender, and the condition x gender interaction

```
mod_mar_con_D2T1 = rma(ti=data_EQUAL_meta$t_X_MAR_CON_D2T1,
                       ni=data_EQUAL_meta$N_X_MAR_CON_D2T1,
                       mi=data_EQUAL_meta$M_X_SOCIND_CON_ALL2T1T2,
                       measure="PCOR",slab=Study)
summary(mod_mar_con_D2T1)
```

```
##
## Random-Effects Model (k = 8; tau^2 estimator: REML)
##
##   logLik  deviance       AIC       BIC      AICc
##  11.2849  -22.5697  -18.5697  -18.6779  -15.5697
##
## tau^2 (estimated amount of total heterogeneity): 0.0000 (SE = 0.0007)
## tau (square root of estimated tau^2 value):      0.0018
## I^2 (total heterogeneity / total variability):   0.21%
## H^2 (total variability / sampling variability):  1.00
##
## Test for Heterogeneity:
## Q(df = 7) = 8.2279, p-val = 0.3129
##
## Model Results:
##
```

```
## estimate      se    zval    pval    ci.lb   ci.ub
##   0.0062  0.0138  0.4531  0.6505  -0.0208  0.0333
##
## ---
## Signif. codes:  0 '***' 0.001 '**' 0.01 '*' 0.05 '.' 0.1 ' ' 1
```

```
forest(mod_mar_con_D2T1,main="Forest plot",header='Study',xlab = '')
mtext(c("Favors individuals with partner", "Favors individuals without partner"),
      side=1, line=3, at=c(-.02,.02),adj=c(1,0))
arrows(x0=c(-.02,.02), y0=-4.4, x1=c(-.2, .2), y1=-4.4, length=0.1, lwd=2, xpd=TRUE)
```

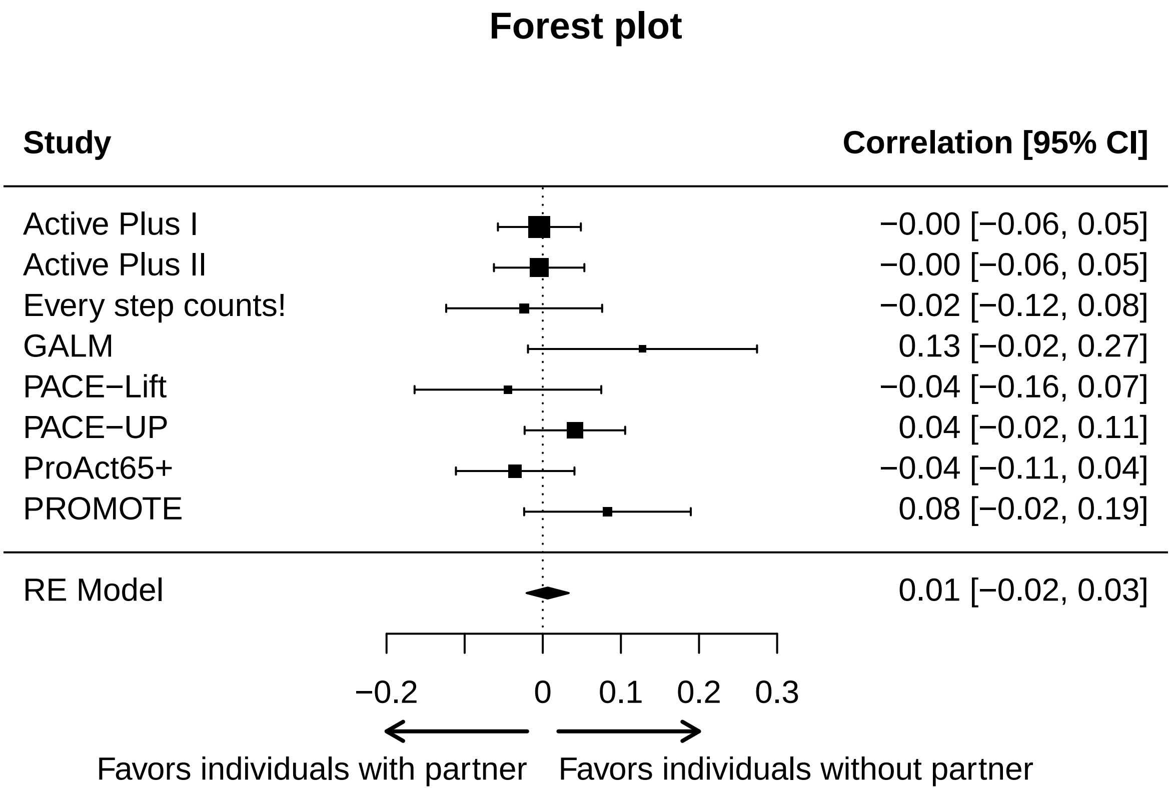


```
funnel(mod_mar_con_D2T1,legend=TRUE,main='Funnel plot')
```

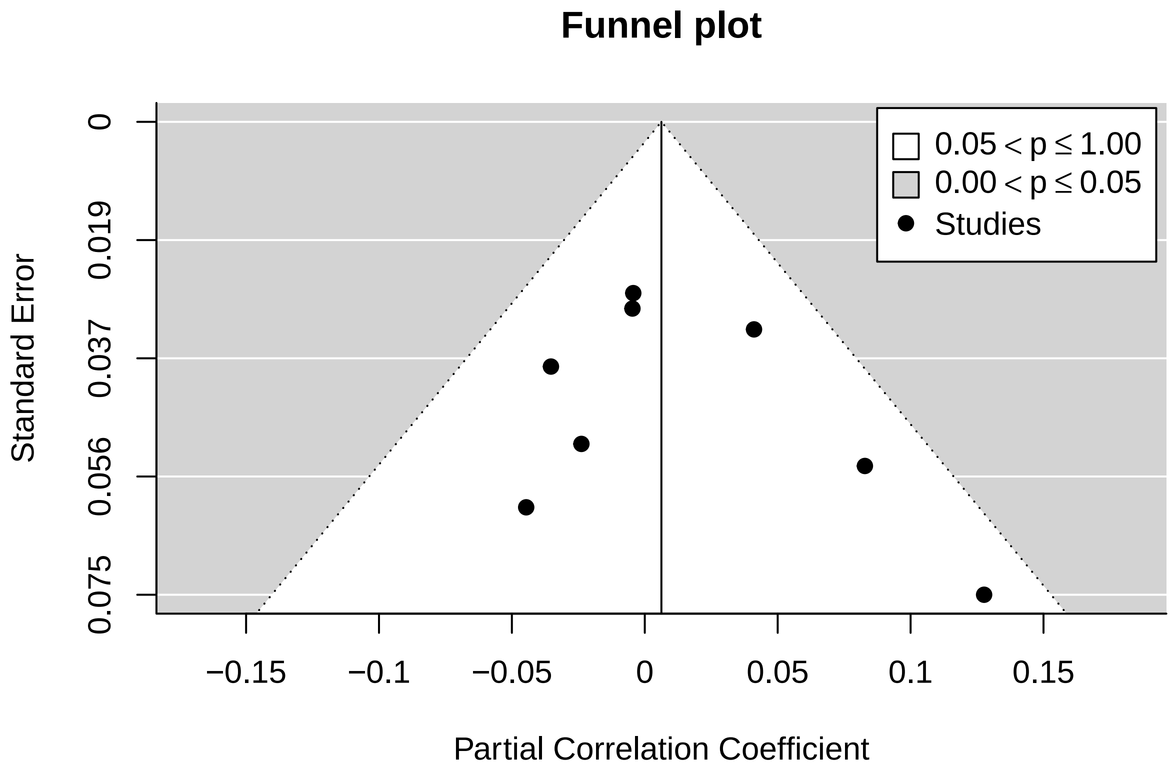

Model D0T2 (data from 5 studies; 5 randomized, 0 non-randomized; 2 with objective physical activity measure, 3 with subjective physical activity measure): The moderated effect of the intervention through marital status (reference: with partner) at T2, adjusted for physical activity level at T0 and age

```
mod_mar_con_D0T2 = rma(ti=data_EQUAL_meta$t_X_MAR_CON_D0T2,
                       ni=data_EQUAL_meta$N_X_MAR_CON_D0T2,
                       mi=data_EQUAL_meta$M_X_SOCIND_CON_ALL0T1T2,
                       measure="PCOR",slab=Study)
```

```
## Warning in rma(ti = data_EQUAL_meta$t_X_MAR_CON_D0T2, ni =
## data_EQUAL_meta$N_X_MAR_CON_D0T2, : Studies with NAs omitted from model fitting.
```

```
summary(mod_mar_con_D0T2)
```

```
##
## Random-Effects Model (k = 5; tau^2 estimator: REML)
##
##   logLik  deviance       AIC       BIC      AICc
##   7.4880  -14.9760  -10.9760  -12.2034    1.0240
##
## tau^2 (estimated amount of total heterogeneity): 0.0000 (SE = 0.0008)
## tau (square root of estimated tau^2 value):      0.0049
## I^2 (total heterogeneity / total variability):   1.91%
## H^2 (total variability / sampling variability):  1.02
##
## Test for Heterogeneity:
## Q(df = 4) = 4.0103, p-val = 0.4046
##
```

```
## Model Results:
##
## estimate      se     zval    pval    ci.lb   ci.ub
##  -0.0018  0.0154  -0.1202  0.9043  -0.0320  0.0283
##
## ---
## Signif. codes:  0 '***' 0.001 '**' 0.01 '*' 0.05 '.' 0.1 ' ' 1
```

```
forest(mod_mar_con_DOT2,main="Forest plot",header='Study',xlab = '')
mtext(c("Favors individuals with partner", "Favors individuals without partner"),
      side=1, line=3, at=c(-.02,.02),adj=c(1,0))
arrows(x0=c(-.02,.02), y0=-3.8, x1=c(-.15, .15), y1=-3.8, length=0.1, lwd=2, xpd=TRUE)
```

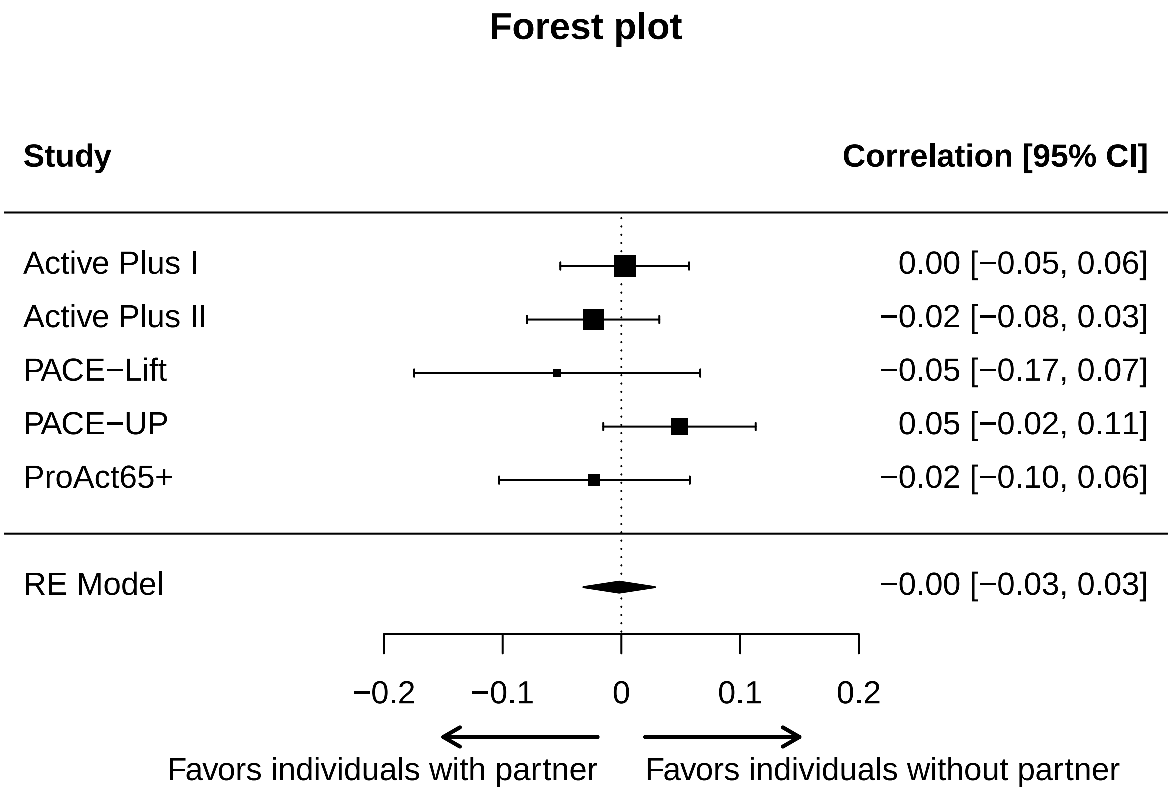


```
funnel(mod_mar_con_DOT2,legend=TRUE,main='Funnel plot')
```

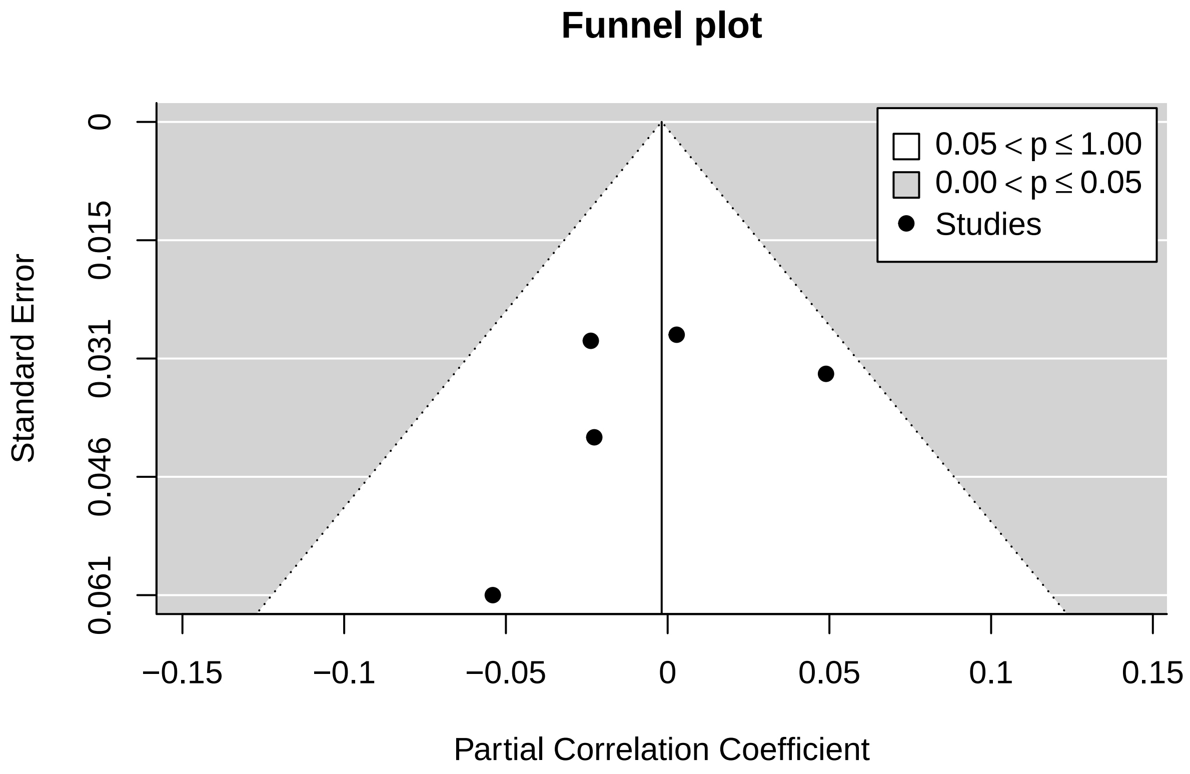

Model D2T2 (data from 5 studies; 5 randomized, 0 non-randomized; 2 with objective physical activity measure, 3 with subjective physical activity measure): The moderated effect of the intervention through marital status (reference: with partner) at T2, adjusted for physical activity level at T0, age, gender, and the condition x gender interaction

```
mod_mar_con_D2T2 = rma(ti=data_EQUAL_meta$t_X_MAR_CON_D2T2,
                       ni=data_EQUAL_meta$N_X_MAR_CON_D2T2,
                       mi=data_EQUAL_meta$M_X_SOCIND_CON_ALL2T1T2,
                       measure="PCOR",slab=Study)
```

```
## Warning in rma(ti = data_EQUAL_meta$t_X_MAR_CON_D2T2, ni =
## data_EQUAL_meta$N_X_MAR_CON_D2T2, : Studies with NAs omitted from model fitting.
```

```
summary(mod_mar_con_D2T2)
```

```
##
## Random-Effects Model (k = 5; tau^2 estimator: REML)
##
##   logLik  deviance       AIC       BIC      AICc
##   7.1122  -14.2243  -10.2243  -11.4517    1.7757
##
## tau^2 (estimated amount of total heterogeneity): 0.0002 (SE = 0.0009)
## tau (square root of estimated tau^2 value):      0.0135
## I^2 (total heterogeneity / total variability):   13.01%
## H^2 (total variability / sampling variability):  1.15
##
## Test for Heterogeneity:
## Q(df = 4) = 4.7848, p-val = 0.3101
```

```
##
## Model Results:
##
## estimate      se     zval    pval     ci.lb   ci.ub
##  -0.0052  0.0166  -0.3127  0.7545  -0.0377  0.0273
##
## ---
## Signif. codes:  0 '***' 0.001 '**' 0.01 '*' 0.05 '.' 0.1 ' ' 1
```

```
forest(mod_mar_con_D2T2,main="Forest plot",header='Study',xlab='')
mtext(c("Favors individuals with partner", "Favors individuals without partner"),
      side=1, line=3, at=c(-.02,.02),adj=c(1,0))
arrows(x0=c(-.02,.02), y0=-3.8, x1=c(-.15, .15), y1=-3.8, length=0.1, lwd=2, xpd=TRUE)
```

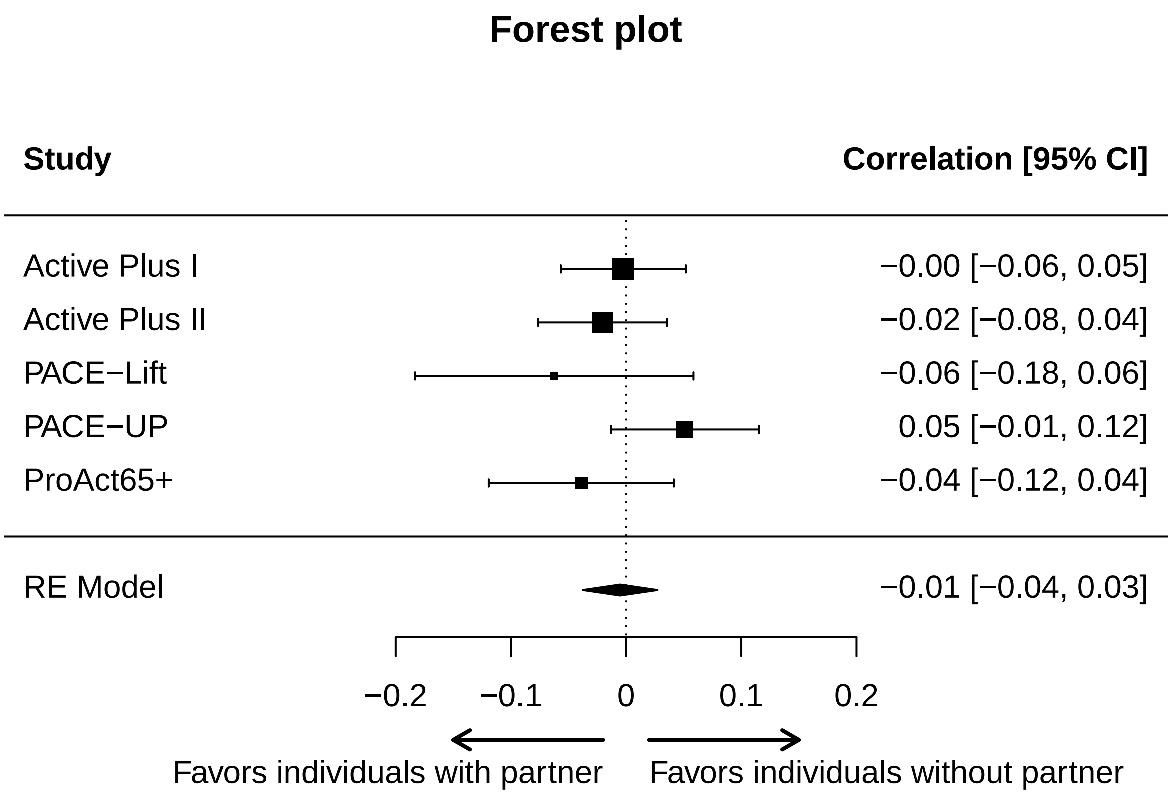


```
funnel(mod_mar_con_D2T2,legend=TRUE,main='Funnel plot')
```

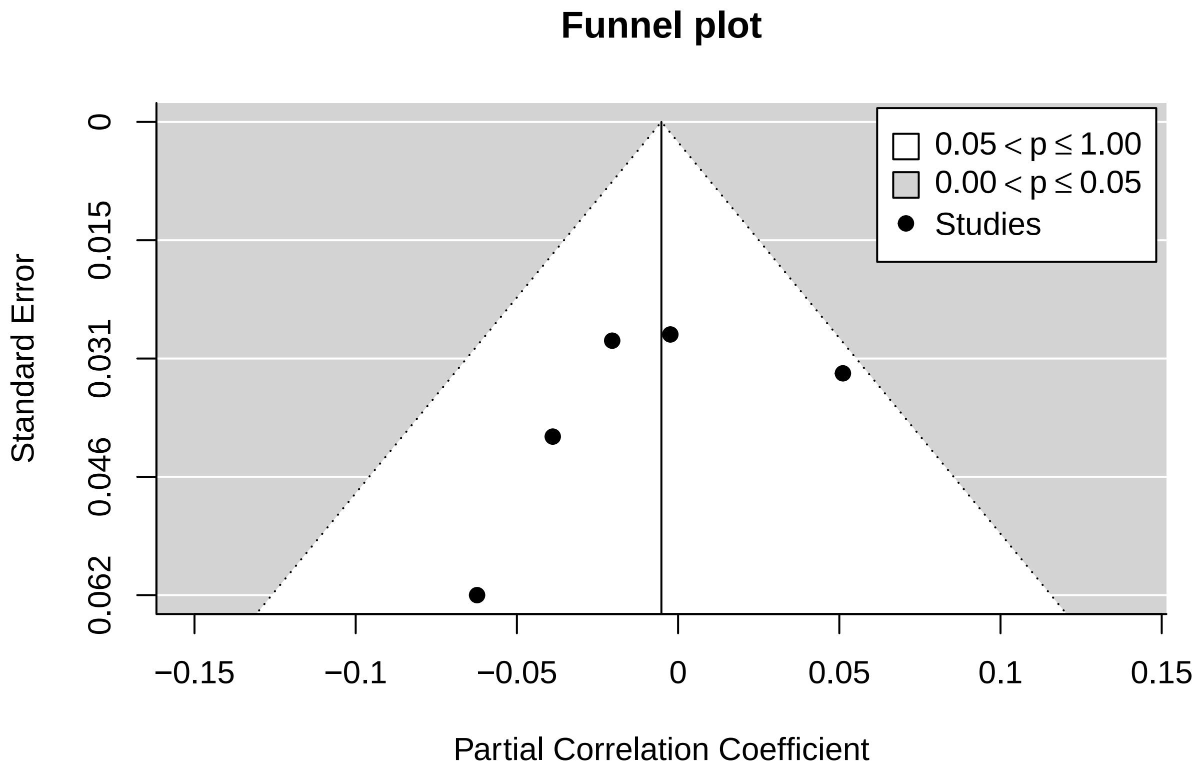

Income-specific intervention effects

Model E0T1 (data from 2 studies; 2 randomized, 0 non-randomized; 0 with objective physical activity measure, 2 with subjective physical activity measure): The moderated effect of the intervention through income (reference: medium/high) at T1, adjusted for physical activity level at T0 and age

```
mod_inc_con_E0T1 = rma(ti=data_EQUAL_meta$t_X_INC_CON_E0T1,
                       ni=data_EQUAL_meta$N_X_INC_CON_E0T1,
                       mi=data_EQUAL_meta$M_X_SOCIND_CON_ALL0T1T2,
                       measure="PCOR",slab=Study)
```

```
## Warning in rma(ti = data_EQUAL_meta$t_X_INC_CON_E0T1, ni =
## data_EQUAL_meta$N_X_INC_CON_E0T1, : Studies with NAs omitted from model fitting.
```

```
summary(mod_inc_con_E0T1)
```

```
##
## Random-Effects Model (k = 2; tau^2 estimator: REML)
##
##   logLik  deviance       AIC       BIC      AICc
##   2.0910   -4.1821   -0.1821   -4.1821   11.8179
##
## tau^2 (estimated amount of total heterogeneity): 0 (SE = 0.0034)
## tau (square root of estimated tau^2 value):      0
## I^2 (total heterogeneity / total variability):   0.00%
## H^2 (total variability / sampling variability):  1.00
##
```

```
## Test for Heterogeneity:
## Q(df = 1) = 0.0106, p-val = 0.9180
##
## Model Results:
##
## estimate      se     zval    pval    ci.lb   ci.ub
##  -0.0001  0.0331  -0.0036  0.9971  -0.0649  0.0647
##
## ---
## Signif. codes:  0 '***' 0.001 '**' 0.01 '*' 0.05 '.' 0.1 ' ' 1
```

```r
forest(mod_inc_con_EOT1,main="Forest plot",header='Study',xlab = '')
mtext(c("Favors medium/high income", "Favors low income"),
      side=1, line=3, at=c(-.02,.02),adj=c(1,0))
arrows(x0=c(-.02,.02), y0=-3, x1=c(-.12, .12), y1=-3, length=0.1, lwd=2, xpd=TRUE)
```

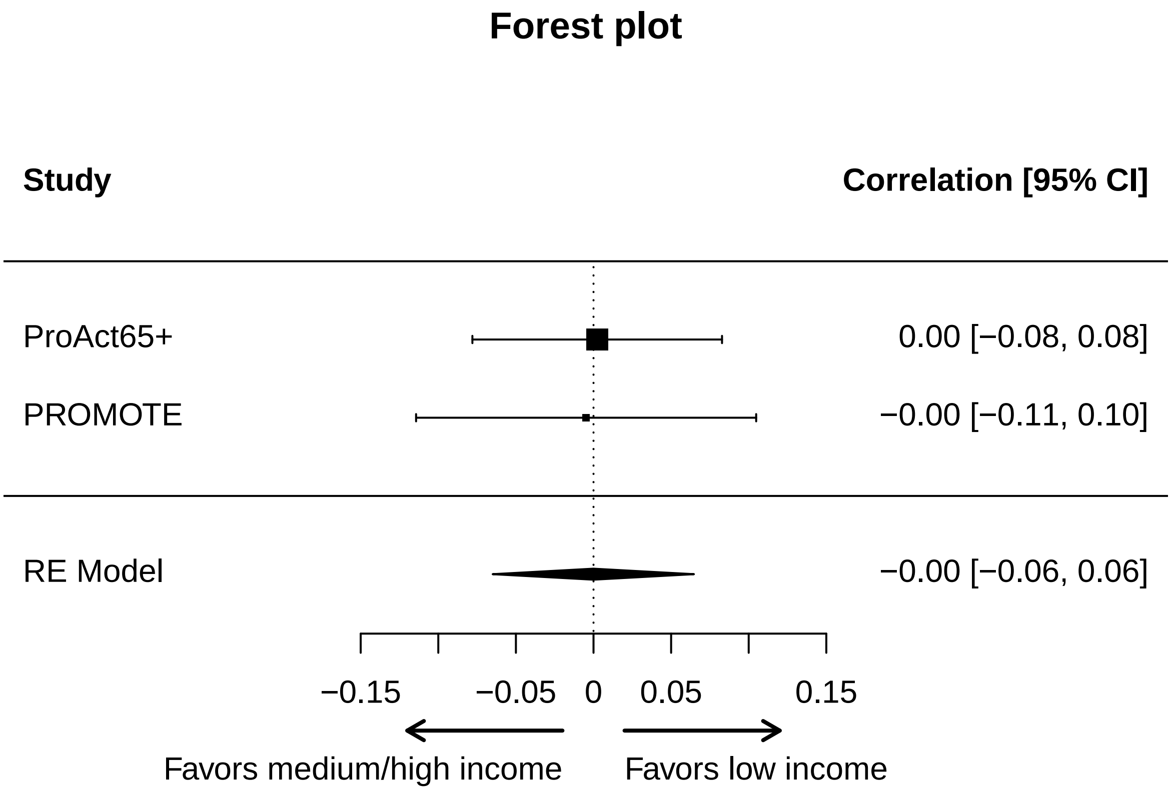


```r
funnel(mod_inc_con_EOT1,legend=TRUE,main='Funnel plot')
```

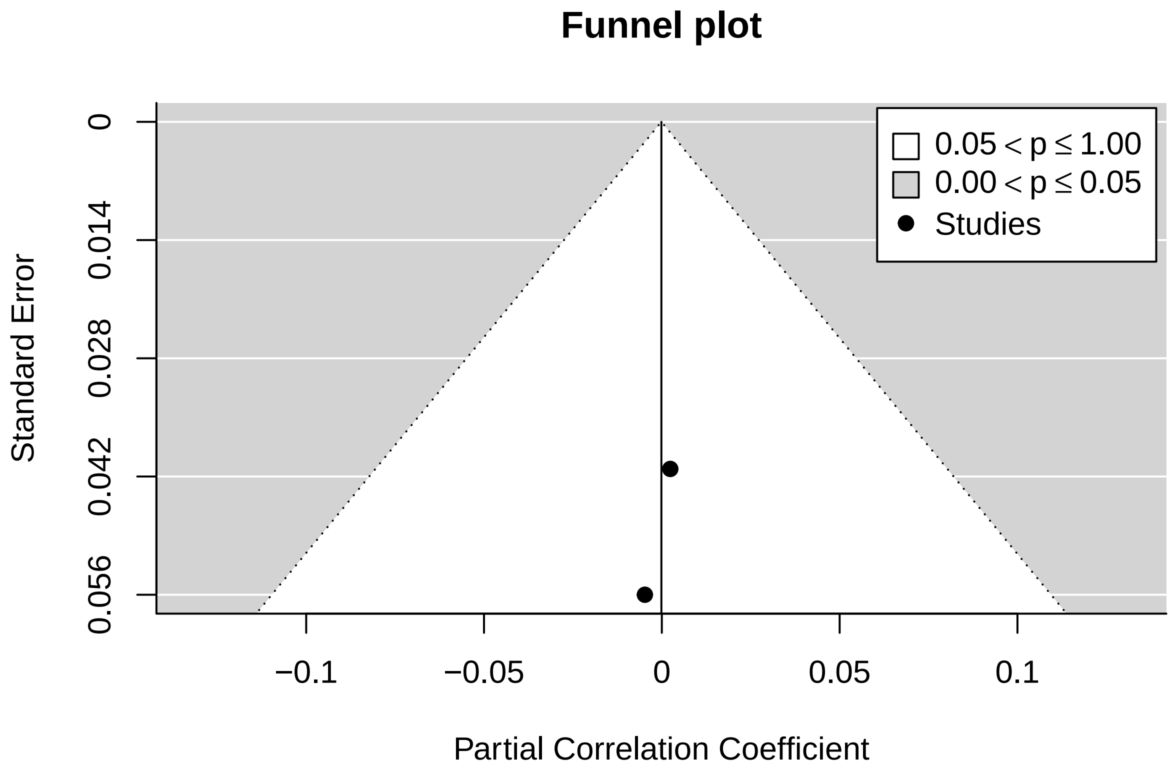

Model E2T1 (data from 2 studies; 2 randomized, 0 non-randomized; 0 with objective physical activity measure, 2 with subjective physical activity measure): The moderated effect of the intervention through income (reference: medium/high) at T1, adjusted for physical activity level at T0, age, gender, and the condition x gender interaction

```
mod_inc_con_E2T1 = rma(ti=data_EQUAL_meta$t_X_INC_CON_E2T1,
                       ni=data_EQUAL_meta$N_X_INC_CON_E2T1,
                       mi=data_EQUAL_meta$M_X_SOCIND_CON_ALL2T1T2,
                       measure="PCOR",slab=Study)
```

```
## Warning in rma(ti = data_EQUAL_meta$t_X_INC_CON_E2T1, ni =
## data_EQUAL_meta$N_X_INC_CON_E2T1, : Studies with NAs omitted from model fitting.
```

```
summary(mod_inc_con_E2T1)
```

```
##
## Random-Effects Model (k = 2; tau^2 estimator: REML)
##
##   logLik  deviance       AIC       BIC      AICc
##   2.0919   -4.1838   -0.1838   -4.1838   11.8162
##
## tau^2 (estimated amount of total heterogeneity): 0 (SE = 0.0034)
## tau (square root of estimated tau^2 value):      0
## I^2 (total heterogeneity / total variability):   0.00%
## H^2 (total variability / sampling variability):  1.00
##
## Test for Heterogeneity:
## Q(df = 1) = 0.0036, p-val = 0.9522
```

```
##
## Model Results:
##
## estimate      se    zval    pval    ci.lb   ci.ub
##   0.0023  0.0331  0.0683  0.9455  -0.0627  0.0672
##
## ---
## Signif. codes:  0 '***' 0.001 '**' 0.01 '*' 0.05 '.' 0.1 ' ' 1
```

```r
forest(mod_inc_con_E2T1,main="Forest plot",header='Study',xlab = '')
mtext(c("Favors medium/high income", "Favors low income"),
      side=1, line=3, at=c(-.02,.02),adj=c(1,0))
arrows(x0=c(-.02,.02), y0=-3, x1=c(-.12, .12), y1=-3, length=0.1, lwd=2, xpd=TRUE)
```

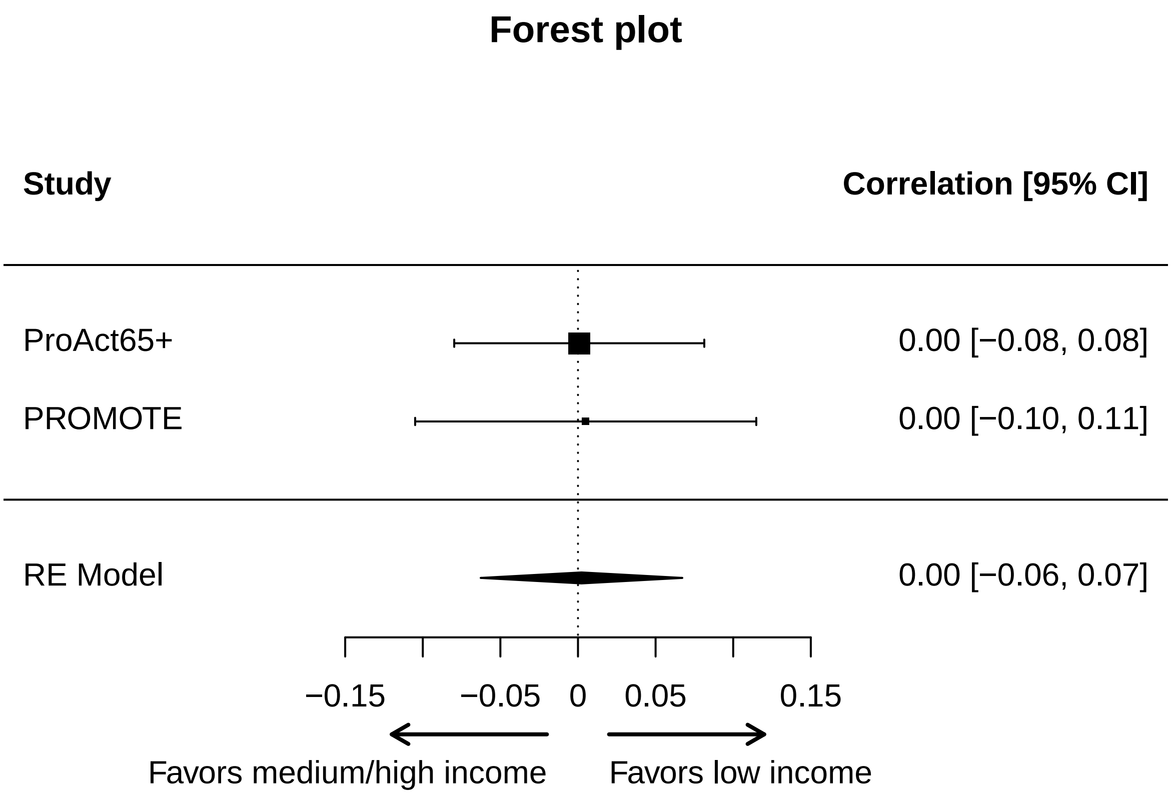


```r
funnel(mod_inc_con_E2T1,legend=TRUE,main='Funnel plot')
```

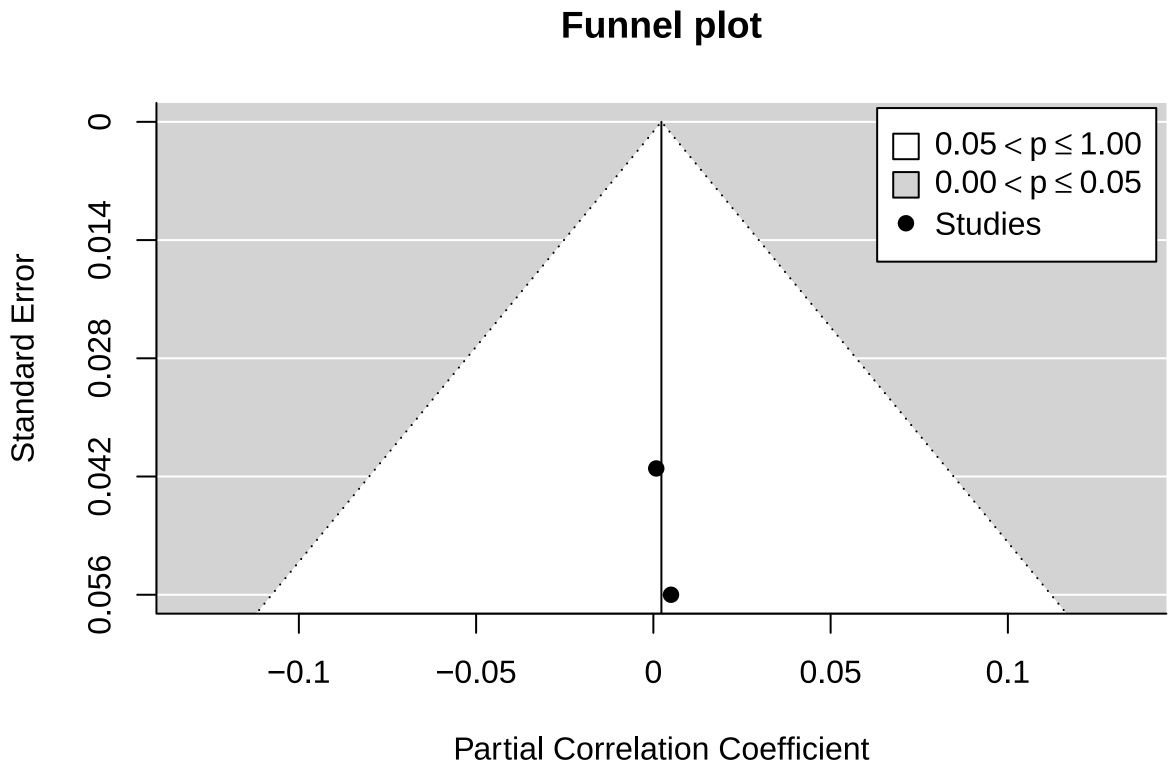

Area deprivation-specific intervention effects

Model F0T1 (data from 3 studies; 3 randomized, 0 non-randomized; 2 with objective physical activity measure, 1 with subjective physical activity measure): The moderated effect of the intervention through area deprivation (reference: medium/low) at T1, adjusted for physical activity level at T0 and age

```
mod_imd_con_F0T1 = rma(ti=data_EQUAL_meta$t_X_IMD_CON_F0T1,
                       ni=data_EQUAL_meta$N_X_IMD_CON_F0T1,
                       mi=data_EQUAL_meta$M_X_SOCIND_CON_ALL0T1T2,
                       measure="PCOR",slab=Study)
```

```
## Warning in rma(ti = data_EQUAL_meta$t_X_IMD_CON_F0T1, ni =
## data_EQUAL_meta$N_X_IMD_CON_F0T1, : Studies with NAs omitted from model fitting.
```

```
summary(mod_imd_con_F0T1)
```

```
##
## Random-Effects Model (k = 3; tau^2 estimator: REML)
##
##   logLik  deviance       AIC       BIC      AICc
##   4.2264   -8.4527   -4.4527   -7.0664    7.5473
##
## tau^2 (estimated amount of total heterogeneity): 0 (SE = 0.0017)
## tau (square root of estimated tau^2 value):      0
## I^2 (total heterogeneity / total variability):   0.00%
## H^2 (total variability / sampling variability):  1.00
##
```

```
## Test for Heterogeneity:
## Q(df = 2) = 0.3805, p-val = 0.8267
##
## Model Results:
##
## estimate      se     zval    pval    ci.lb   ci.ub
##  -0.0389  0.0232  -1.6782  0.0933  -0.0843  0.0065  .
##
## ---
## Signif. codes:  0 '***' 0.001 '**' 0.01 '*' 0.05 '.' 0.1 ' ' 1
```

```r
forest(mod_imd_con_F0T1,main="Forest plot",header='Study',xlab = '')
mtext(c("Favors medium/low area deprivation", "Favors high area deprivation"),
      side=1, line=3, at=c(-.02,.02),adj=c(1,0))
arrows(x0=c(-.02,.02), y0=-3.3, x1=c(-.12, .12), y1=-3.3, length=0.1, lwd=2, xpd=TRUE)
```

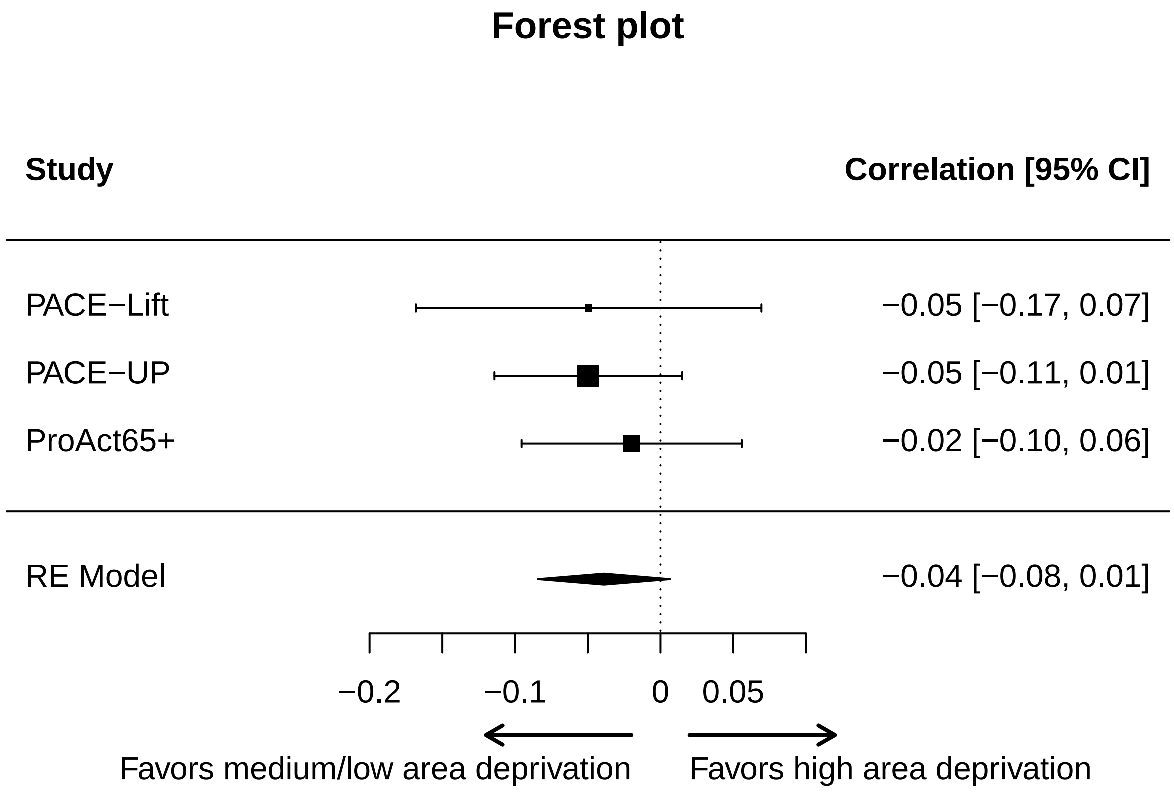


```r
funnel(mod_imd_con_F0T1,legend=TRUE,main='Funnel plot')
```

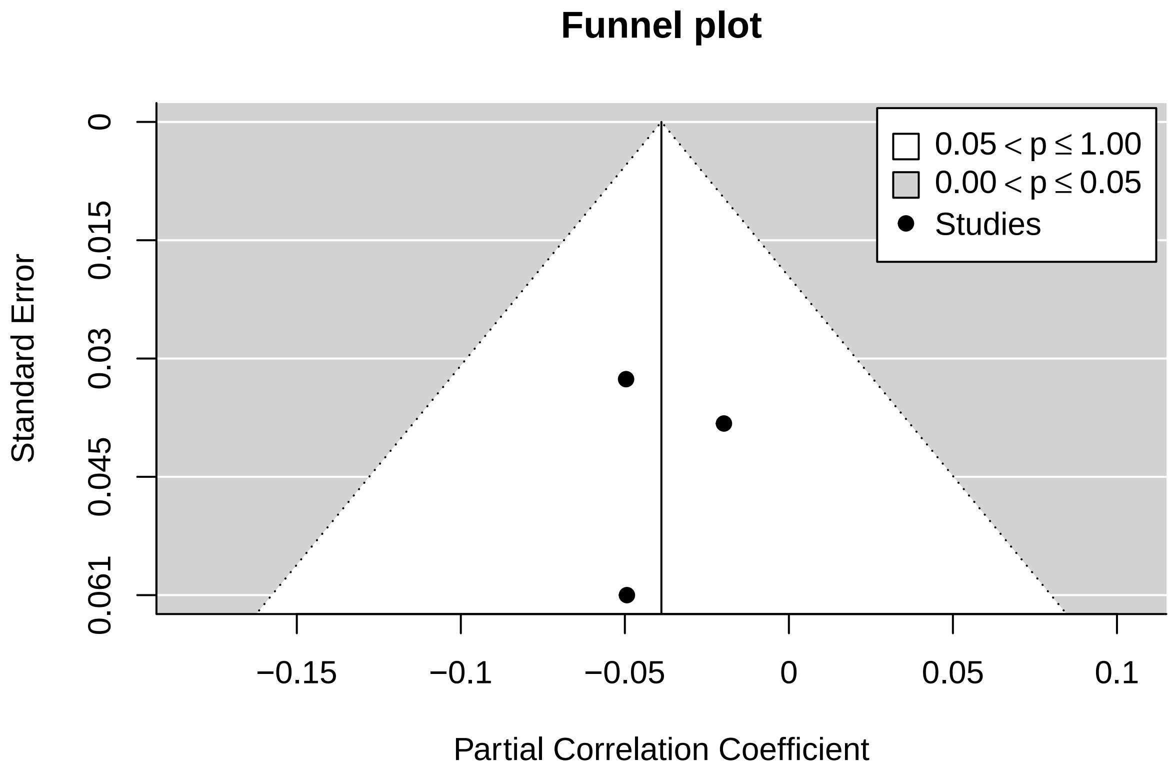

Model F2T1 (data from 3 studies; 3 randomized, 0 non-randomized; 2 with objective physical activity measure, 1 with subjective physical activity measure): The moderated effect of the intervention through area deprivation (reference: medium/low) at T1, adjusted for physical activity level at T0, age, gender, and the condition x gender interaction

```
mod_imd_con_F2T1 = rma(ti=data_EQUAL_meta$t_X_IMD_CON_F2T1,
                       ni=data_EQUAL_meta$N_X_IMD_CON_F2T1,
                       mi=data_EQUAL_meta$M_X_SOCIND_CON_ALL2T1T2,
                       measure="PCOR",slab=Study)
```

```
## Warning in rma(ti = data_EQUAL_meta$t_X_IMD_CON_F2T1, ni =
## data_EQUAL_meta$N_X_IMD_CON_F2T1, : Studies with NAs omitted from model fitting.
```

```
summary(mod_imd_con_F2T1)
```

```
##
## Random-Effects Model (k = 3; tau^2 estimator: REML)
##
##   logLik  deviance       AIC       BIC      AICc
##   4.2914   -8.5829   -4.5829   -7.1966    7.4171
##
## tau^2 (estimated amount of total heterogeneity): 0 (SE = 0.0017)
## tau (square root of estimated tau^2 value):      0
## I^2 (total heterogeneity / total variability):   0.00%
## H^2 (total variability / sampling variability):  1.00
##
## Test for Heterogeneity:
## Q(df = 2) = 0.2409, p-val = 0.8865
```

```
##
## Model Results:
##
## estimate      se     zval    pval    ci.lb   ci.ub
##  -0.0400  0.0232  -1.7253  0.0845  -0.0855  0.0054  .
##
## ---
## Signif. codes:  0 '***' 0.001 '**' 0.01 '*' 0.05 '.' 0.1 ' ' 1
```

```
forest(mod_imd_con_F2T1,main="Forest plot",header='Study',xlab = '')
mtext(c("Favors medium/low area deprivation", "Favors high area deprivation"),
      side=1, line=3, at=c(-.02,.02),adj=c(1,0))
arrows(x0=c(-.02,.02), y0=-3.3, x1=c(-.12, .12), y1=-3.3, length=0.1, lwd=2, xpd=TRUE)
```

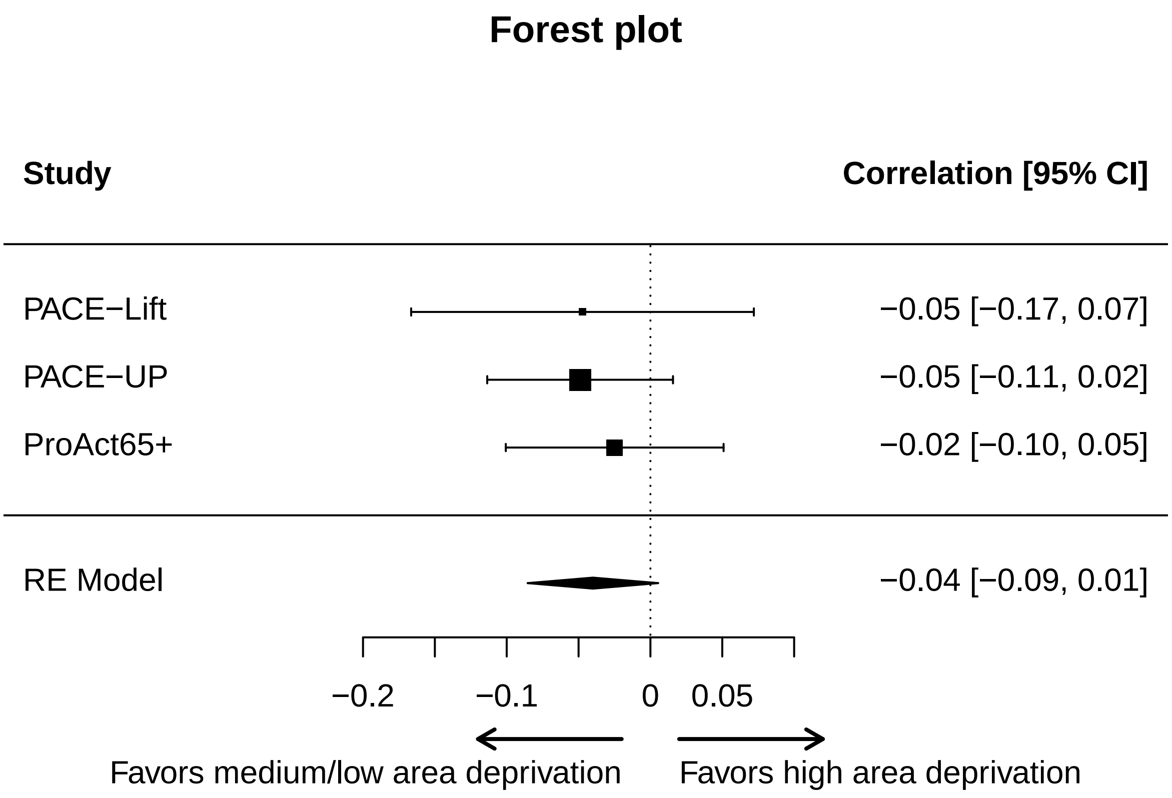


```
funnel(mod_imd_con_F2T1,legend=TRUE,main='Funnel plot')
```

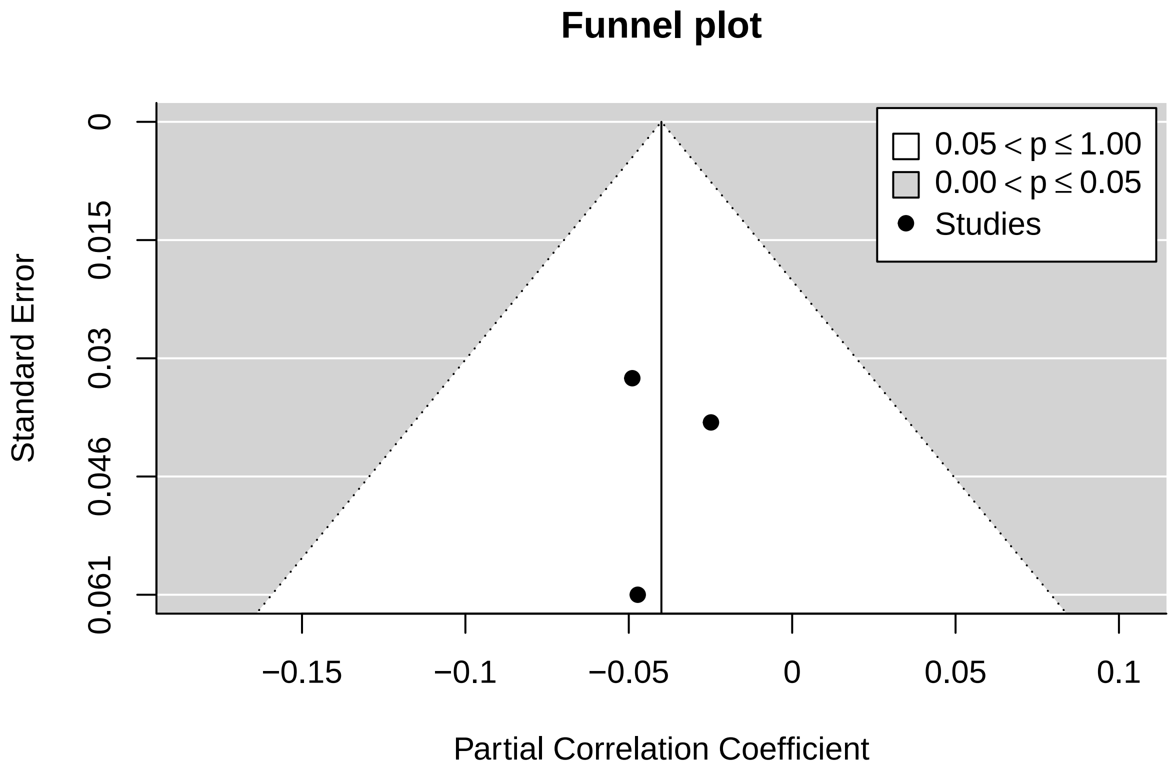

Model F0T2 (data from 3 studies; 3 randomized, 0 non-randomized; 2 with objective physical activity measure, 1 with subjective physical activity measure): The moderated effect of the intervention through area deprivation (reference: medium/low) at T2, adjusted for physical activity level at T0 and age

```
mod_imd_con_F0T2 = rma(ti=data_EQUAL_meta$t_X_IMD_CON_F0T2,
                       ni=data_EQUAL_meta$N_X_IMD_CON_F0T2,
                       mi=data_EQUAL_meta$M_X_SOCIND_CON_ALL0T1T2,
                       measure="PCOR",slab=Study)
```

```
## Warning in rma(ti = data_EQUAL_meta$t_X_IMD_CON_F0T2, ni =
## data_EQUAL_meta$N_X_IMD_CON_F0T2, : Studies with NAs omitted from model fitting.
```

```
summary(mod_imd_con_F0T2)
```

```
##
## Random-Effects Model (k = 3; tau^2 estimator: REML)
##
##   logLik  deviance       AIC       BIC      AICc
##   3.5972   -7.1943   -3.1943   -5.8080    8.8057
##
## tau^2 (estimated amount of total heterogeneity): 0.0000 (SE = 0.0018)
## tau (square root of estimated tau^2 value):      0.0011
## I^2 (total heterogeneity / total variability):   0.06%
## H^2 (total variability / sampling variability):  1.00
##
## Test for Heterogeneity:
## Q(df = 2) = 1.5489, p-val = 0.4610
##
```

```
## Model Results:
##
## estimate      se     zval    pval    ci.lb   ci.ub
##  -0.0332  0.0237  -1.4013  0.1611  -0.0797  0.0132
##
## ---
## Signif. codes:  0 '***' 0.001 '**' 0.01 '*' 0.05 '.' 0.1 ' ' 1
```

```
forest(mod_imd_con_F0T2,main="Forest plot",header='Study',xlab = '')
mtext(c("Favors medium/low area deprivation", "Favors high area deprivation"),
      side=1, line=3, at=c(-.02,.02),adj=c(1,0))
arrows(x0=c(-.02,.02), y0=-3.3, x1=c(-.12, .12), y1=-3.3, length=0.1, lwd=2, xpd=TRUE)
```

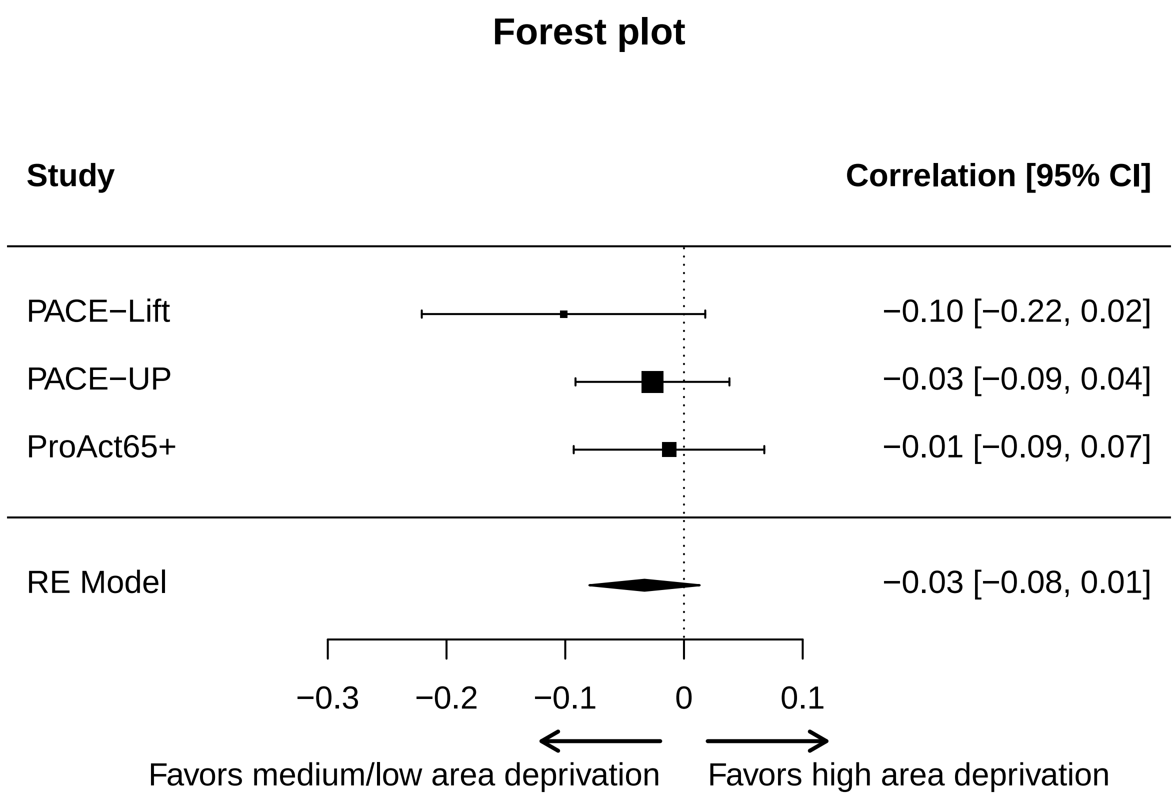


```
funnel(mod_imd_con_F0T2,legend=TRUE,main='Funnel plot')
```

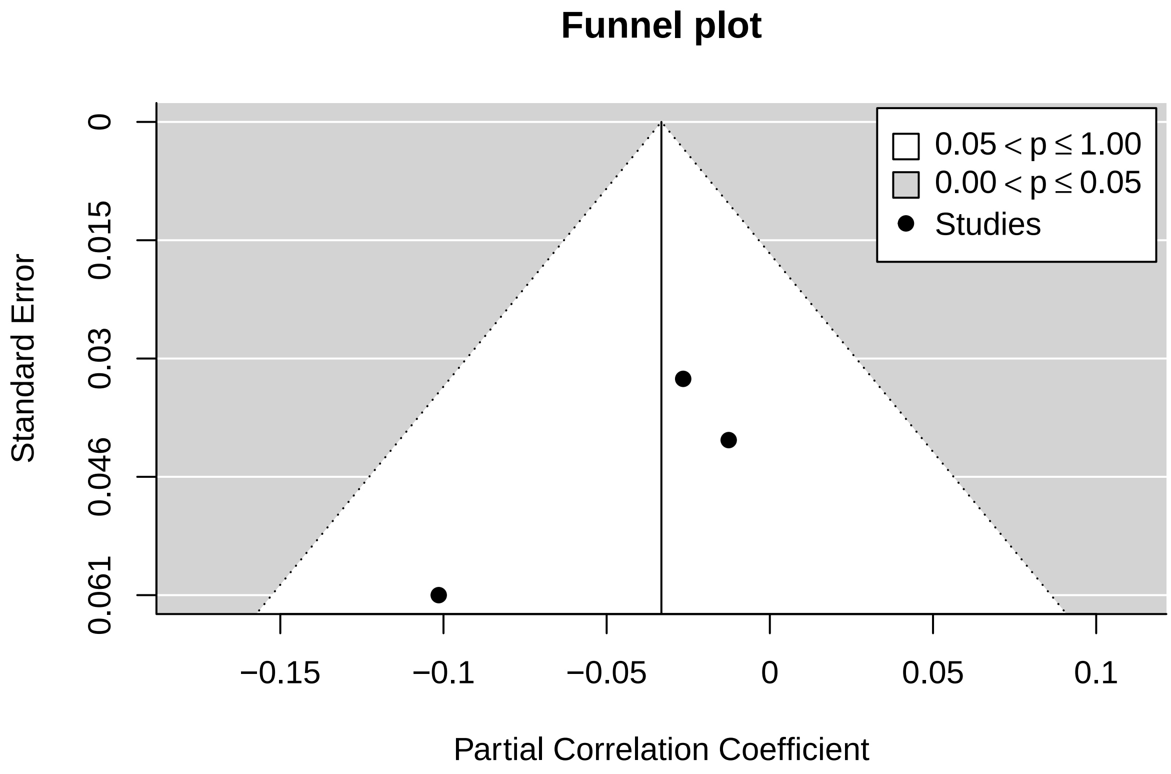

Model F2T2 (data from 3 studies; 3 randomized, 0 non-randomized; 2 with objective physical activity measure, 1 with subjective physical activity measure): The moderated effect of the intervention through area deprivation (reference: medium/low) at T2, adjusted for physical activity level at T0, age, gender, and the condition x gender interaction

```
mod_imd_con_F2T2 = rma(ti=data_EQUAL_meta$t_X_IMD_CON_F2T2,
                       ni=data_EQUAL_meta$N_X_IMD_CON_F2T2,
                       mi=data_EQUAL_meta$M_X_SOCIND_CON_ALL2T1T2,
                       measure="PCOR",slab=Study)
```

```
## Warning in rma(ti = data_EQUAL_meta$t_X_IMD_CON_F2T2, ni =
## data_EQUAL_meta$N_X_IMD_CON_F2T2, : Studies with NAs omitted from model fitting.
```

```
summary(mod_imd_con_F2T2)
```

```
##
## Random-Effects Model (k = 3; tau^2 estimator: REML)
##
##   logLik  deviance       AIC       BIC      AICc
##   3.7734   -7.5469   -3.5469   -6.1606    8.4531
##
## tau^2 (estimated amount of total heterogeneity): 0 (SE = 0.0018)
## tau (square root of estimated tau^2 value):      0
## I^2 (total heterogeneity / total variability):   0.00%
## H^2 (total variability / sampling variability):  1.00
##
## Test for Heterogeneity:
## Q(df = 2) = 1.1850, p-val = 0.5529
```

```
##
## Model Results:
##
## estimate      se     zval    pval    ci.lb   ci.ub
##  -0.0335  0.0237  -1.4122  0.1579  -0.0801  0.0130
##
## ---
## Signif. codes:  0 '***' 0.001 '**' 0.01 '*' 0.05 '.' 0.1 ' ' 1
```

```
forest(mod_imd_con_F2T2,main="Forest plot",header='Study',xlab = '')
mtext(c("Favors medium/low area deprivation", "Favors high area deprivation"),
      side=1, line=3, at=c(-.02,.02),adj=c(1,0))
arrows(x0=c(-.02,.02), y0=-3.3, x1=c(-.12, .12), y1=-3.3, length=0.1, lwd=2, xpd=TRUE)
```

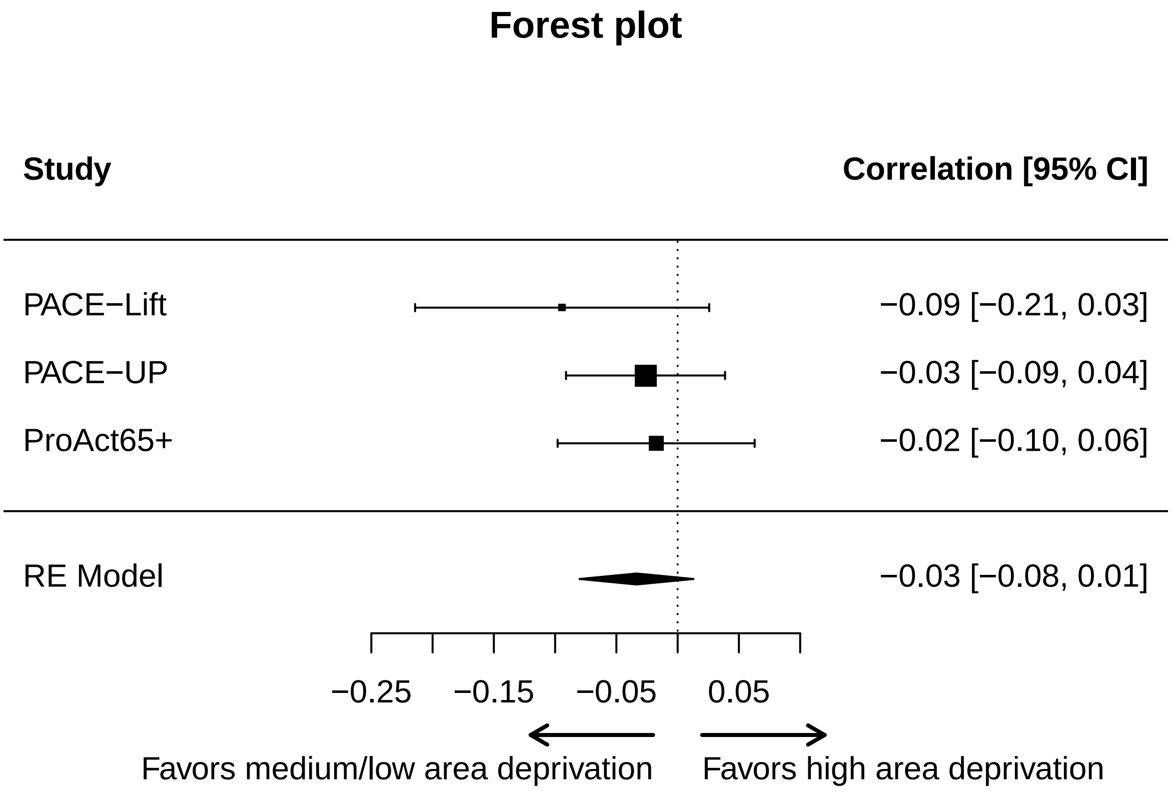


```
funnel(mod_imd_con_F2T2,legend=TRUE,main='Funnel plot')
```

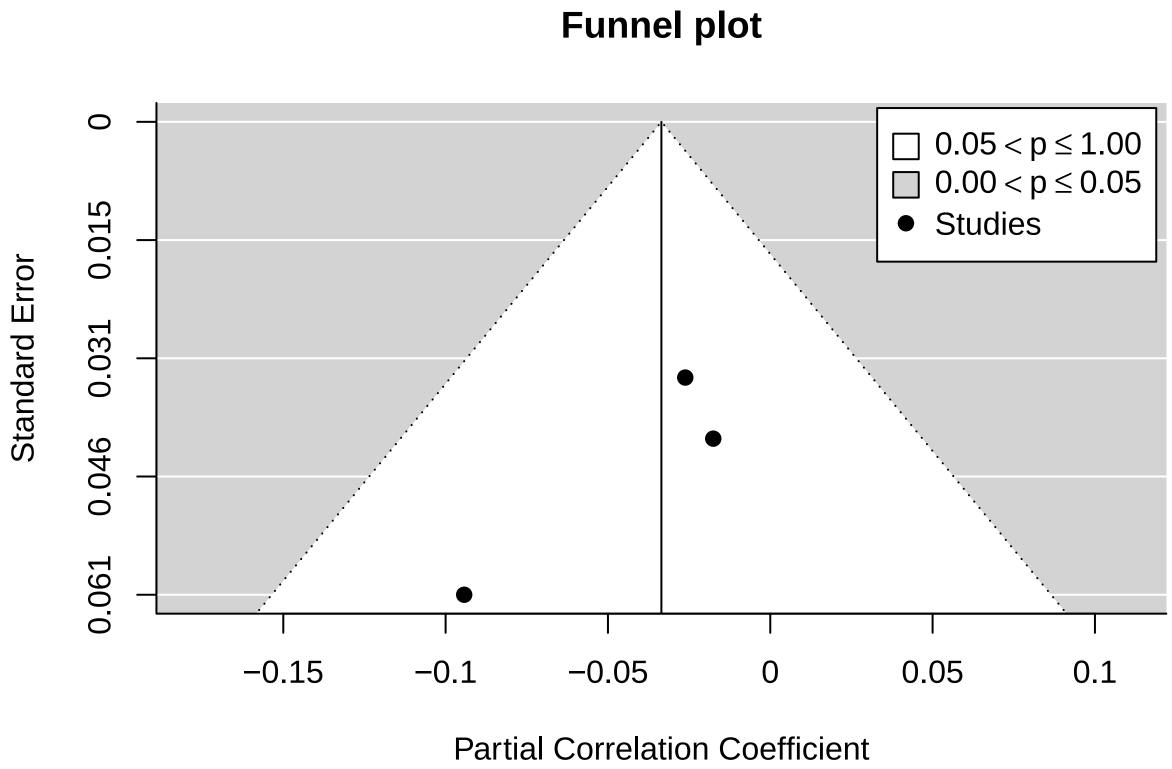
Funnel plot
0.05 < p ≤ 1.00
0.00 < p ≤ 0.05
Studies
Standard Error
0
0.015
0.031
0.046
0.061
−0.15
−0.1
−0.05
0
0.05
0.1
Partial Correlation Coefficient
